# Proteomic Characterization, Biodistribution, and Functional Studies of Immune-Therapeutic Exosomes: Implications for Inflammatory Lung Diseases

**DOI:** 10.3389/fimmu.2021.636222

**Published:** 2021-03-25

**Authors:** Mahmoud Elashiry, Ranya Elsayed, Mohamed M. Elashiry, Mohammad H. Rashid, Roxan Ara, Ali S. Arbab, Ahmed R. Elawady, Mark Hamrick, Yutao Liu, Wenbo Zhi, Rudolf Lucas, Jose Vazquez, Christopher W. Cutler

**Affiliations:** ^1^ Department of Periodontics, Dental College of Georgia at Augusta University, Augusta, GA, United States; ^2^ Department of Endodontics, College of Dentistry, Ainshams University, Cairo, Egypt; ^3^ Georgia Cancer Center, Department of Biochemistry and Molecular Biology, at Augusta University, Augusta, GA, United States; ^4^ Department of Cellular Biology and Anatomy, Medical College of Georgia at Augusta University, Augusta, GA, United States; ^5^ Center of Biotechnology and Genomic Medicine, at Augusta University, Augusta, GA, United States; ^6^ Vascular Biology Center, Medical College of Georgia at Augusta University, Augusta, GA, United States; ^7^ Division of Pulmonary and Critical Care Medicine, Medical College of Georgia at Augusta University, Augusta, GA, United States; ^8^ Division of Infectious Diseases, Department of Medicine, Medical College of Georgia at Augusta University, Augusta, GA, United States

**Keywords:** dendritic cells, exosomes, lung diseases, immune therapy, COVID-19

## Abstract

Dendritic cell (DC)-derived exosomes (DC EXO), natural nanoparticles of endosomal origin, are under intense scrutiny in clinical trials for various inflammatory diseases. DC EXO are eobiotic, meaning they are well-tolerated by the host; moreover, they can be custom-tailored for immune-regulatory or -stimulatory functions, thus presenting attractive opportunities for immune therapy. Previously we documented the efficacy of immunoregulatory DCs EXO (regDCs EXO) as immunotherapy for inflammatory bone disease, in an *in-vivo* model. We showed a key role for encapsulated TGFβ1 in promoting a bone sparing immune response. However, the on- and off-target effects of these therapeutic regDC EXO and how target signaling in acceptor cells is activated is unclear. In the present report, therapeutic regDC EXO were analyzed by high throughput proteomics, with non-therapeutic EXO from immature DCs and mature DCs as controls, to identify shared and distinct proteins and potential off-target proteins, as corroborated by immunoblot. The predominant expression in regDC EXO of immunoregulatory proteins as well as proteins involved in trafficking from the circulation to peripheral tissues, cell surface binding, and transmigration, prompted us to investigate how these DC EXO are biodistributed to major organs after intravenous injection. Live animal imaging showed preferential accumulation of regDCs EXO in the lungs, followed by spleen and liver tissue. In addition, TGFβ1 in regDCs EXO sustained downstream signaling in acceptor DCs. Blocking experiments suggested that sustaining TGFβ1 signaling require initial interaction of regDCs EXO with TGFβ1R followed by internalization of regDCs EXO with TGFβ1-TGFβ1R complex. Finally, these regDCs EXO that contain immunoregulatory cargo and showed biodistribution to lungs could downregulate the main severe acute respiratory syndrome coronavirus 2 (SARS-CoV-2) target receptor, ACE2 on recipient lung parenchymal cells *via* TGFβ1 *in-vitro.* In conclusion, these results in mice may have important immunotherapeutic implications for lung inflammatory disorders.

## Introduction

Exosomes (EXO) are nanoparticles of endosomal origin secreted by all cells, including dendritic cells (DCs), the most potent antigen presenting cells and “directors” of immune response (1). EXO contain proteins, nucleic acids and lipid cargo that mediate intercellular communication and signaling. They are secreted into tissues and body fluids and can act locally or from at a distance (2). In addition, EXO have desirable traits as a drug delivery system, which includes their small size (30-150 nm), their low clearance from target tissues and cargo preservation (3–6). DC-derived EXO are already being used to deliver molecular cargo to promote cytotoxic T cell responses for cancer (7) or inhibit effector T cell responses and hyperinflammation in autoimmune/inflammatory diseases (8).

We have previously reported important aspects of the immunobiology and functions of DC-derived EXO subtypes, isolated from DCs at distinct stages of maturation. Most notably among these subtypes are immune-regulatory (regDCs EXO), loaded with TGFβ1 and IL10 and deficient in costimulatory molecules. These have been shown to “reprogram” recipient DCs and CD4+ T cells towards an immune-regulatory response *in vitro* and *in vivo*. Immature or immune-null DC exo, (iDCs EXO) and EXO from matured DCs, called immune-stimulatory (stimDCs EXO), were also characterized for immune functions (5). The stability of these EXO, their ability to protect their cargo, and be retained at inflamed mucosal sites and inhibit inflammatory bone loss has also been documented. Other groups have had similar success with DC-derived EXO loaded with immune-regulatory cargo in various disease states such as inflammatory colitis and other inflammatory disease states (9–11). Currently lacking however, is a more in-depth characterization of the proteomic cargo of therapeutic DC EXO and their biodistribution to different body organs, needed to interpret the on-target and off-target effects of such immune regulatory nanoparticles. In addition, how regDCs EXO modulate cytokine signaling in recipient cells (5), needs further investigations.

TGFβ1 is a master regulator of the immune response (12, 13). TGFβ1 is a pleiotropic cytokine that, at high levels activates SMAD2/3, inhibits DC maturation, and suppresses effector Th1 and Th2, and Th17 cells, thereby promoting anti-inflammatory FoxP3+ T-regulatory cells. Moreover, TGFβ1 may have other therapeutic advantages in fatal infectious diseases such as COVID-19, by inhibiting one of the SARS-CoV-2 attachments and point of entry, like the ACE2 receptor (14–16).

SARS-CoV-2 is a coronavirus that gains entry primarily *via* the mucosal respiratory tract and is the etiologic agent of the COVID-19 pandemic (17). Symptoms of SARS-CoV-2 infection can vary greatly, depending on host factors, from asymptomatic infection to a severe and intense hyperinflammatory state creating multiorgan failure, especially in the respiratory tract (18). The severe inflammatory disease called acute respiratory distress syndrome (ARDS) is one of the leading causes of death in COVID-19 patients (19–21). An exaggerated immune/inflammatory response, due to release of pro-inflammatory cytokines, i.e. the “cytokine storm”, is a main characteristic of ARDS in COVID-19 patients, and is responsible for producing severe damage to numerous organs including the lung tissue and frequently death (22, 23). One of the main entry points for SARS-CoV-2 invasion is *via* its structural proteins such as spike (S) and others, *via* the ACE2 expressing cells (24), however the ability to regulate ACE2 using DC-derived EXO is unclear. Several immunotherapeutic approaches to regulate the inflammatory process or block ACE2 have been proposed, however the data on efficacy and the associated adverse effects are contradictory (25, 26).

The purpose of the present murine study was 4-fold: 1. To characterize, in-depth, the proteomic cargo of immune therapeutic and non-therapeutic DC EXO subtypes, to validate on-target functions and potential off-target effects; 2. To track, in live mice, the biodistribution patterns of intravenously injected therapeutic regDCs EXO into major organs, including the lungs *in vivo*; 3. To reveal how TGFβ1 in regDCs EXO activates target cell signaling; and 4. To test the ability of putative recipient cells of SARS-CoV-2 to uptake regDC EXO, and thus influence ACE2 expression.

## Methods

### Ethics Statement

The Institutional Animal Care and Use Committee (IACUC) of Augusta University (protocol # 2013-0586) approved all experimental procedures on C57BL/6 mice.

### Generation of Dendritic Cell Subsets

DC subsets including immature, immune-stimulatory (mature) and immune-regulatory were generated as we previously described (5, 27). Briefly, bone marrow was isolated from tibias and femurs of 6- to 8-week-old mice. Contaminating erythrocytes were lysed by ACK cell lysing buffer (Invitrogen, Thermofisher scientific, and Columbia, SC, USA). Cells were cultured in complete media (RPMI 1640 containing 10% FBS and 100 IU/mL penicillin/streptomycin) containing 20 ng/ml of murine GM-CSF and IL-4 (Peprotech, Rocky Hill, NJ, USA). Culture media was changed every 2 days and cells were re-incubated on day 6 in EXO depleted complete media to generate iDCs or in the presence of 1ug/ml LPS (Sigma, St. Louis, M.O., USA) to generate mature stimDCs for 48 hours. regDCs were generated by adding TGFβ1/IL10 recombinant cytokines on Day 5, in which 1 × 10^7^ DCs were incubated for 2 hours with 1ug/ml TGFβ1 (R&D Systems, Inc. Minneapolis, MN) and 1 μg/mL of the recombinant murine IL-10 (Cell Sciences, Canton, Massachusetts) in a total volume of 1 mL serum-free media, then diluted 1:10 in fresh complete media for 24 h. This is followed by harvesting, washing and culturing for 48 h in EXO depleted growth media. Culture supernatants were collected for EXO isolation on day 8.

Phenotypic characterization of DCs subsets were defined by expression level of differentiation and maturation markers including CD11c+ (N418) (Invitrogen), MHCII (M5/114.15.2) (Milteny biotech Auburn, CA,USA) and CD86 (GL1) (Invitrogen), (Milteny biotech) using flow cytometry and by expression level of pro/anti-inflammatory cytokine mRNA by PCR, including IL6 (Mm00446190_m1), IL12 (Mm01288989_m1), IL23 (Mm00518984_m1), TGFβ1: Mm01178820_m1 and TNF: Mm00443258_m1, (Thermofisher Scientific). Phenotypic profile was as follows: regDCs : CD11c +, low MHCII+, low CD86+, low CD80 + and low CD40+, iDCs: CD11c+, intermediate MHCII+, intermediate CD86+, intermediate CD80+, intermediate IL6+, intermediate IL12+ and intermediate IL23+ and stimDCs: CD11c+, high MHCII+, high CD86+, high CD80+, high IL6+, high IL12+ and high IL23 (5).

### Exosome Isolation and Purification

EXO isolation was performed as previously described (9). Briefly, culture supernatants were subjected to differential centrifugation (successive centrifugations at 500 g for (5 min), 2000g for (20 min), and 10,000 g for (30 min)) to eliminate cells and debris, followed by ultrafiltration 3x with 0.2 um and 3x with 100 kDA filters (to remove free proteins) and ultracentrifugation for 1.5 h at 120,000 g. To further remove excess free proteins, EXO pellets were washed with a large volume of PBS and ultra-centrifuged 2x at 120,000 g for 1.5 h, and finally re-suspended in 100 ul of PBS for further studies.

### Cytokine Loading of Immunoregulatory Dendritic Cell Exosomes

To increase the concentration of immunoregulatory factors like TGFβ1 and IL10, 1 x10^9^ particles of regDC EXO were actively loaded by sonication (3) with 5ug TGFβ1 and 5ug IL10 in 500 ul of PBS then filtered 3x by ultrafiltration with 100KDA filter to remove free proteins and washed 3x with large volume of PBS and ultra-centrifugation at 120,000 g for 1.5 h to further purify EXO from free molecules, and finally re-suspended in 100 ul of PBS. The supernatants of where regDCs EXO were incubated were isolated and checked for any contaminants of free TGFβ1 and IL10 by ELISA. It is important to mention that in our previous we have seen that TGFβ1 and IL10 were naturally loaded in regDCs EXO but their concentrations were very low. Thus, additional artificial loading was performed to achieve the desired immunoregulatory effect (5).

### Characterization of Dendritic Cell-Derived Exosomes

DCs EXO subsets were characterized for their size distribution, particle number and shape using nanotracking analysis and TEM respectively, and for exosomal markers using Western blot (WB) as we previously showed. In brief, nanoparticle tracking analysis (NTA) was used to visualize and quantitate size and count of nanoparticles in suspension using ZetaView PMX 110 (Particle Metrix, Meerbusch, Germany) and software (ZetaView 8.02.28). For TEM, EXO samples were loaded onto a copper grid. After precipitation of EXO, the sample liquid was isolated, and counter stained for 10 minutes with 2% phosphotungstic acid solution and then placed under an incandescent lamp for 5 min. EXO sample was then analyzed with TEM. For WB analysis, EXO lysates were isolated to confirm principal EXO proteins using anti-TSG101 (MA1-23296), anti-Alix (MA1-83977), anti-CD63 (10628D) and GRP94 (MA3-016) from (Invitrogen, Thermofisher scientific West Columbia, SC, USA) as we showed previously (5).

### Liquid Chromatography–Mass Spectrometry Analysis

Three biological replicates of regDCs, iDCs and stimDCs EXO samples were lyophilized to dryness. 100 ul of freshly prepared 50mM ammonium bicarbonate buffer with 0.1% acid labile detergent RapiGest SF Surfactant (Waters) was added to each sample to resuspend exosomes. This was followed by reduction with dithiothreitol, alkylation using iodoacetamide and digestion overnight using trypsin (Thermo Scientific #90057). Trifluoroacetic acid (TFA) was added to a final concentration of 0.1% to the digested sample, followed by incubation at 37°C for 40 minutes. Peptide digests were analyzed on an Orbitrap Fusion tribrid mass spectrometer (Thermo Scientific) coupled with an Ultimate 3000 nano-UPLC system (Thermo Scientific). Two microliters of reconstituted peptide were first trapped and washed on a Pepmap100 C18 trap (5um, 0.3X5mm) at 20ul/min using 2% acetonitrile in water (with 0.1% formic acid) for 10 minutes and then separated on a Pepman 100 RSLC C18 column (2.0 um, 75-μm × 150-mm) using a gradient of 2 to 40% acetonitrile with 0.1% formic acid over 40 min at a flow rate of 300nl/min and a column temperature of 40°C. Analysis of DCs EXO samples were then performed by data-dependent acquisition in positive mode using Orbitrap MS analyzer for precursor scan at 120,000 FWHM from 300 to 1500 m/z and ion-trap MS analyzer for MS/MS scans in top speed mode. Collision-induced dissociation (CID) was used as fragmentation method. Raw data were processed using Proteome Discoverer (v1.4, Thermo Scientific) and submitted for SequestHT search against database of Uniport. Fixed value Peptide spectrum matching (PSM) validator algorithm was used for peptide spectrum matching validation. SequestHT search parameters were 10 ppm precursor and 0.6 Da product ion tolerance, with static Carbamidomethylation (+57.021 Da).

### Bioinformatics Analysis

Clustered heat map of the expression profiles of the differentially expressed overlapped proteins in DCs EXO subtypes was conducted by ClastVist software (28). All proteins that showed a fold-change of at least 1.5 and satisfied *p* < 0.05 were differentially expressed. The database of Kyoto Encyclopedia of Genes and Genomes (KEGG) and Gene Ontology (GO) were performed using Web Gestalt software (29) to categorize exclusive, shared, differentially, and non-differentially expressed proteins in DCs EXO subsets. Functional categories and pathways with a corrected *p* < 0.05 were considered as significant. Percentage and significant level of DCs EXO subsets proteins that are related to the identified pathways were then identified.

### *In Vivo* Imaging of Immunoregulatory Dendritic Cell Exosomes and Biodistribution

#### *In Vivo* Imaging

1.5 to 2 mCi of Indium-111-Oxine (AnazaoHealth Corporation, Tampa, FL, USA) in PBS was incubated with 200 µl of exosomes particles (~2 x 10^9^ particles) at 37°C for 20 minutes. Free Indium-111-Oxine was removed by repeated PBS washes through an Amicon ultrafiltration device. Isolated Indium-111-Oxine-labeled EXO were diluted to 200 µCi of radioactivity per dose and injected intravenously *via* tail vein. Control animals received an injection of equivalent activity of free Indium-111-Oxine. Whole body and head single photon emission spectroscopy (SPECT) images were acquired by Mediso’s nanoScan microSPECT/CT system (Mediso, USA) at 3 and 24 h after injection. Images were reconstructed and imaging software was used to calculate radioactivity in the major organs and lymph nodes, as a percentage of total radioactivity (whole body). Afterwards, organs were excised and weighed after the last time point and *ex vivo* radioactivity measurements were performed by gamma counter (Perkin-Elmer Packard Cobra II Auto-Gamma) and expressed per mg wet weight (30).

### Exosome Uptake *In Vitro*

For EXO uptake study *in vitro*, EXO labeled with Dil (D282, Thermofisher Scientific) were co‐cultured with DCs or mouse primary tracheal/bronchial respiratory epithelium cells (PTBECs) (C57-6033, Cell Biologics, USA) for 24 h. Cells were fixed and stained on glass slides with Alex flour 647 phalloidin (A22287) and DAPI (D1306) (Invitrogen, Thermofisher scientific West Columbia, SC, USA). In some experiment’s cells were stained for TGFβR1 primary Antibody (PA5-32631) and labeled with Goat anti-Rabbit IgG Secondary Antibody, Alexa Fluor 488 conjugate (A27034) (Thermofisher Scientific, USA). The images were then acquired by scanning confocal fluorescence microscopy.

### Cell Culture and Reagents

Immature DCs or mouse primary tracheal/bronchial epithelium cells (PTBECs) were incubated with and without 10^8^/ml EXO regDCs EXO, iDCs EXO in the presence or absence of TGFβ1R blocker (SB 431542, R and D, USA, endocytosis inhibitor Cytochalasin D (C8273, Sigma Aldrich, USA) and free TGFβ1 (with dose approximately matching that in regDCs EXO) for 1 h and 24 hrs. Cells were harvested and ACE2 mRNA were measured by polymerase chain reaction (PCR) while ACE2 surface markers levels were measured by flow cytometry. Phosphorylation of TGβ1 transcription factors was assessed by western blot using anti PSMAD2/3 (D6G10), anti SMAD2/3 (D7G7), with anti-GAPDH (D16H11) or anti-Beta-actin (8H10D10) as loading control (Cell Signaling Technology, Danvers, MA, USA).

### Flow Cytometry and Antibodies

FACS Staining Buffer (Thermofisher scientific) was used to stain cells on ice. FC receptors (FcR) were blocked using mouse FcR blocking reagent (Miltenyi Biotec) for 15 minutes protected from light. Primary goat anti mouse ACE2 antibody (AF3437) at recommended concentration were added for 30 minutes followed by APC-conjugated Anti-Goat IgG Secondary Antibody (F0108) (R and D). Cells were washed, re-suspended in FACS buffer and data was acquired using Milteny biotech machine and software.

### Real-Time Polymerase Chain Reaction

Total RNA was isolated from DCs *in vitro* and from oral mucosal (gingiva) tissue of the experimental groups used for *in vivo* studies using QIAGEN RNeasy mini kit (Qiagen, Inc., Valencia, CA, and USA). RNA purity and concentration were analyzed with Nanodrop (NanoDrop 1000 UV-VIS Spectrophotometer Software Ver.3.8.1, Thermofisher Scientific). Ratio of 260/280 of 2.0 was considered adequate for analysis and was reverse transcribed to cDNA. Amplification by PCR was performed using the High-Capacity cDNA Reverse Transcription Kit and PCR in total reaction of 20 μL. Quantitative real-time PCR was performed using TaqMan gene expression primers specific for ACE 2 (Mm00446190_m1), and Beta Actin (Mm02619580_g1)(Thermofisher Scientific). RT-PCR was run in StepOnePlus Real-Time PCR System. Relative gene expression was determined using delta-delta CT and plotted as relative fold change.

### Western Blotting

Cells or EXO lysates were extracted by addition of RIPA buffer supplemented by protease/phosphatase inhibitor cocktail and incubated for 20 minutes on ice. Proteins (10 μg) were separated using 14% Mini-PROTEAN TGX Precast Protein Gel (Bio-Rad Laboratories, Hercules, CA), and transferred onto PVDF membranes (Sigma-Aldrich). After blocking with 5% nonfat dry milk in PBS, the membrane was incubated with primary antibodies, washed with TBST, and incubated with HRP-conjugated secondary antibodies for 1 h at room temperature. The membranes were developed by ECL kit and imaged with ChemiDoc MP Imaging Gel (Bio-Rad Laboratories, Hercules, CA).

### Statistical Analysis

Data analysis was performed by two-way or one ANOVA followed by Tukey’s multiple-comparisons test using GraphPad Prism 6 (GraphPad Software, La Jolla, CA). Values are expressed as mean ± standard deviation (SD) and experiments were done in triplicates.

## Results

### *Bona Fide* Dendritic Cell Exosome Subtypes for Proteomic, Functional Studies

**Figure 1:**
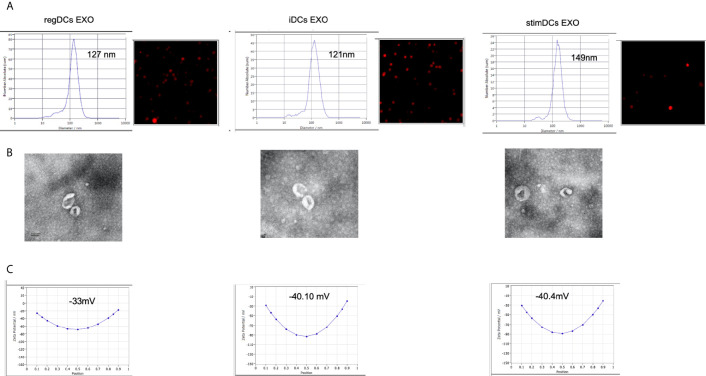
**(A)** Nano-tracking analysis to determine EXO number and size distribution in nm. **(B)** Transmission electron microscopy (TEM) to visualize EXO shape and size. **(C)** Zeta potential analysis to determine EXO surface charge. regDCs EXO (Left), iDCs EXO (middle) and stimDCs EXO (right).

Phenotypic analysis of bone marrow derived donor DC subtypes from C57BL/6 mice, as well as isolation, purification, and validation procedures of DC EXO from these subtypes are described in methods. Briefly, correct size distribution (30-150 nm) and shape of EXO were confirmed by nanoparticle tracking analysis (**Figure 1A**) and TEM (**Figure 1B**), while surface charge/colloidal stability was measured by the zeta potential (**Figure 1C**). Our published report (5) further validated bona fide EXO, based on their expression of CD63, CD81, Escort related proteins including ALIX and TSG101 and negative expression of GRP94.

### Liquid Chromatography–Mass Spectrometry Analysis of Dendritic Cell Exosome Proteins

**Figure 2:**
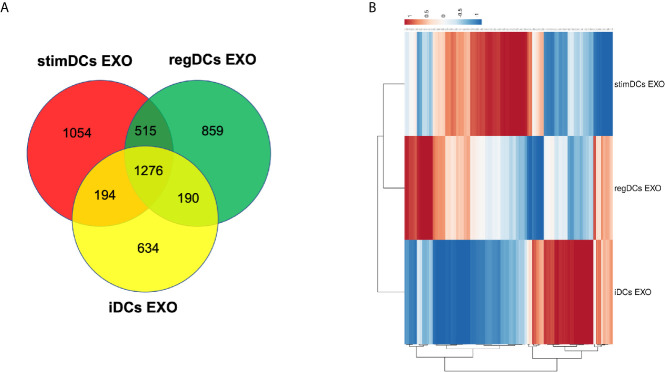
**(A)** Venn diagram showing the overlap of proteins between regDCs EXO, iDCs EXO and StimDCs EXO. 1278 proteins were overlapped, whereas 859, 1,054, and 634 proteins were unique to regDCs EXO, stimDCs EXO, and iDCs EXO, respectively. **(B)** Heat map showing differential expression in the overlapped proteins. The dots are color coded with red and blue indicating upregulation and downregulation, respectively.

DC EXO subtypes are complex nano-particles, with on-target functions typically consistent with the phenotype of source DCs. This was previously reported by our group and includes regulation by regDC EXO of inflammatory cytokines, and the reprogramming of DC maturation and Treg-Th17 cell effector differentiation (5). In this study, we focused on in-depth proteomic LC-MS/MS analysis, to identify both on- and off-target proteins that could lead to unintended consequences of DC EXO therapy. We were able to identify 1276 overlapping or shared proteins. Moreover, we identified 859,1054 and 634 proteins unique to regDCs EXO, stimDCs EXO and iDCs EXO, respectively. These are illustrated in a Venn diagram (**Figure 2A**). Details of the unique and overlapping proteins are listed in **Supplementary Tables 1–5**. Of the overlapped proteins, 235 showed significantly different expression levels. A Clustered Heatmap performed using ClustVist software shows the expression patterns of the differentially expressed proteins (DEPs) in DCs EXO subsets (**Figure 2B**). More details of DEPs showing PSM/sum of total PSM values in each DCs EXO subsets are listed in **Supporting Information Table 5**.

**Figure 3:**
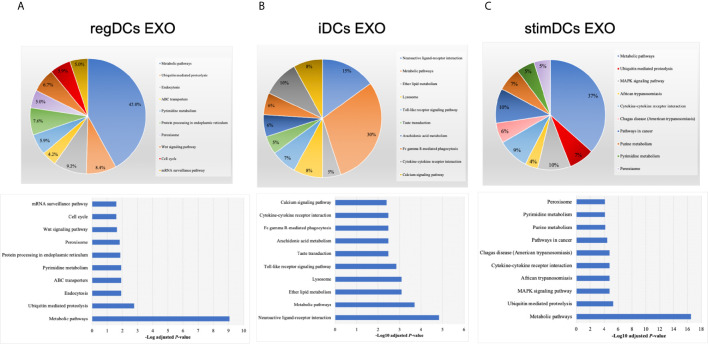
KEGG pathway enrichment analysis of the uniquely expressed proteins listed on their percentage (upper panel) and level of significance (lower panel) in **(A)** regDCs EXO, **(B)** iDCs EXO, and **(C)** stimDCs EXO.

**Figure 4:**
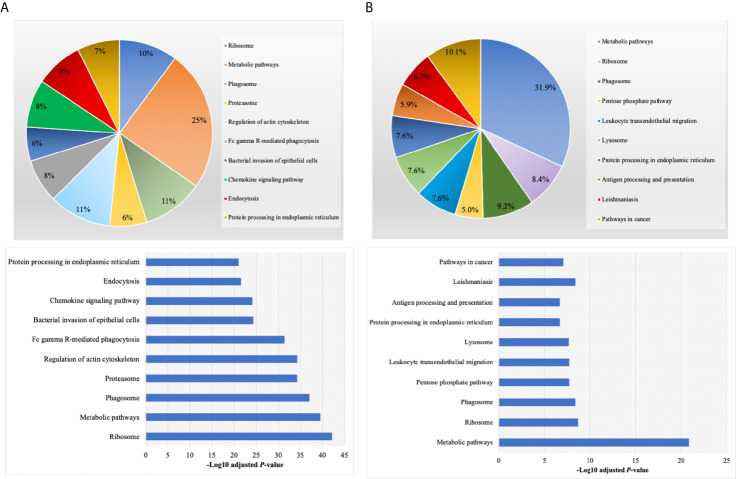
KEGG pathway enrichment analysis of **(A)** non-differentially expressed and **(B)** differentially expressed proteins listed on their percentage (upper panel) and level of significance (lower panel).

By annotating unique and overlapped expressed proteins into the Kyoto Encyclopedia of Genes and Genomes (KEGG) database and identifying the top ten pathways, we discovered that regDC EXO proteins are involved in: metabolic pathways, ubiquitin mediated proteolysis, endocytosis, ABC transporters, Pyrimidine metabolism, protein processing in the endoplasmic reticulum, peroxisome, Wnt signaling pathway, and in cell cycle and mRNA surveillance pathways. iDCs EXO proteins are involved in: neuroactive ligand receptor interactions, metabolic pathways, ether lipid metabolism, lysosome, Toll-like receptor signaling pathways, taste transduction, arachidonic acid metabolism, Fc gamma R-mediated phagocytosis, cytokine-cytokine receptor interaction and in the calcium signaling pathway. StimDCs EXO proteins are involved in: metabolic pathways, ubiquitin mediated proteolysis, MAPK signaling pathway, African trypanosomiasis, cytokine-cytokine receptor interaction, Chagas disease (American trypanosomiasis), pathways in cancer, purine metabolism, pyrimidine metabolism and peroxisome. **Figures 3A–C**. The non-DEPs were related to metabolic pathways, ribosome, phagosome, pentose phosphate pathway, leukocyte trans-endothelial migration, lysosome, protein processing in endoplasmic reticulum, antigen processing and presentation, Leishmaniasis and pathways in cancer (**Figure 4A**). DEPs were involved in ribosome, metabolic pathways, phagosome, proteasome, regulation of actin cytoskeleton, Fc gamma R-mediated phagocytosis, bacterial invasion of epithelial cells, chemokine signaling pathway, endocytosis, and protein processing in endoplasmic reticulum (**Figure 4B**). Collectively, these data showed that DC EXO subsets are mostly involved in pathways related to cellular immune function.

**Figure 5:**
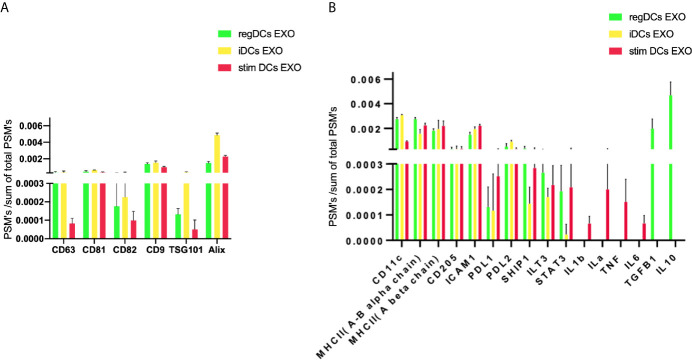
**(A)** Identification of exosomal markers tetraspanins and ESCRT complex related proteins and **(B)** DCs markers, immune-stimulatory/inhibitory molecules and pro/anti-inflammatory cytokines in DCs EXO.

**Figure 6:**
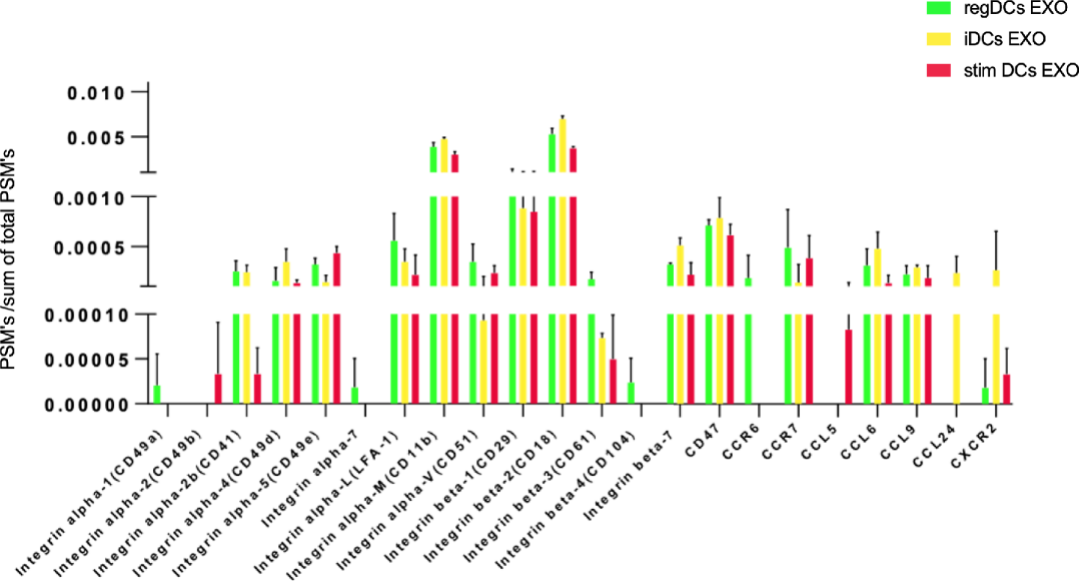
Identification of integrins and chemotactic factors in DCs EXO.

Further validation of high-throughput proteomic analysis was evidenced by expression in all DCs EXO subsets of EXO proteins CD63, CD81, CD82, C9, ALIX and TSG101 (**Figure 5A**), previously identified by WB and immunogold plating (5). DC markers and immunological/inflammatory molecules including CD11c, MHCII, CD205, ICAM1, SHIP1, LT3B, PDL1, PDL2, STAT3, IL1a, IL1β, TNF, IL6, IL10 and TGFβ1 were found in DC EXO. The negative regulators of inflammation included SHIP1, LT3B, and STAT3 and were expressed in both regDC EXO and stimDC EXO. In line with our published WB data, ELISA and immune gold plating (5), IL6, TNF, IL1β, and ILα were detected only in stimDCs EXO, while TGFβ1 and IL10 were exclusively detected in regDCs EXO (**Figure 5B**). In addition, a variety of integrins and chemotactic markers were differentially expressed in DCs EXO subsets (**Figure 6**). RegDCs EXO contain integrin alpha-1, integrin alpha-7, integrin beta-4 and CCR6, while stimDC EXO contain integrin alpha-2, and CCL5 and iDC EXO contain CCL24. DCs EXO subsets were also found to express integrin alpha-2b, integrin alpha-4, integrin alpha-5, integrin alpha-L (LFA-1), integrin alpha-M (CD11B), integrin alpha-V, integrin beta-1, integrin beta-2, integrin beta-3, integrin beta-7 and CD47, as well as other chemotactic factors including CCR7, CCL6, CCL9, and CXCR2. Collectively, these data suggest that DCs EXO subsets differentially express chemotactic and adhesion molecules that may have a role in homing, trafficking, cell adhesion and immune regulation. In addition, regDCs EXO are enriched with naturally as well as artificially loaded immunoregulatory/anti-inflammatory cargo.

**Figure 7:**
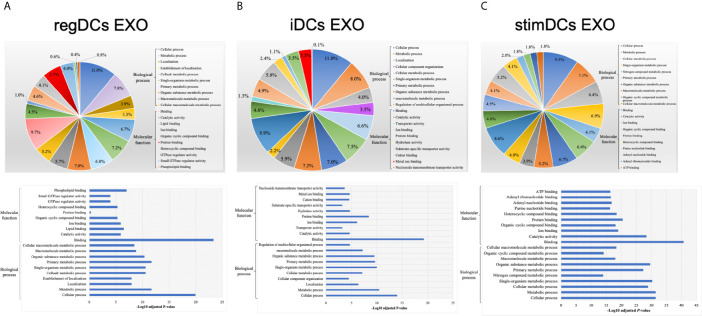
Gene ontology (GO) enrichment analysis of the uniquely expressed proteins listed on their percentage (upper panel) and level of significance (lower panel) in **(A)** regDCs EXO, **(B)** iDCs EXO, and **(C)** stimDCs EXO.

**Figure 8:**
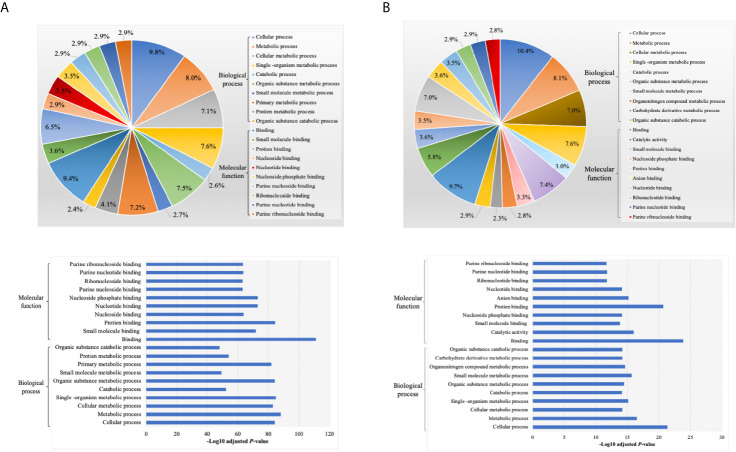
Gene ontology (GO) enrichment of proteins **(A)** non differentially expressed and **(B)** differentially expressed proteins between DCs exosomes subsets, listed on their percentage (upper panel) and level of significance (lower panel).

We next analyzed the putative protein functions in DCs EXO subtypes according to the biological process and molecular function by Gene Ontology (GO) analysis. These results are shown in detail in **Figures 7** and **8**. Briefly, while unique expression patterns in DCs EXO subtypes were observed, all are enriched in proteins of cellular localization, metabolism and protein and phospholipid binding.

### *In Vivo* Live Imaging and Anatomic Biodistribution of Radioisotope Indium-111-Oxine-Labeled Dendritic Cell Exosome

**Figure 9:**
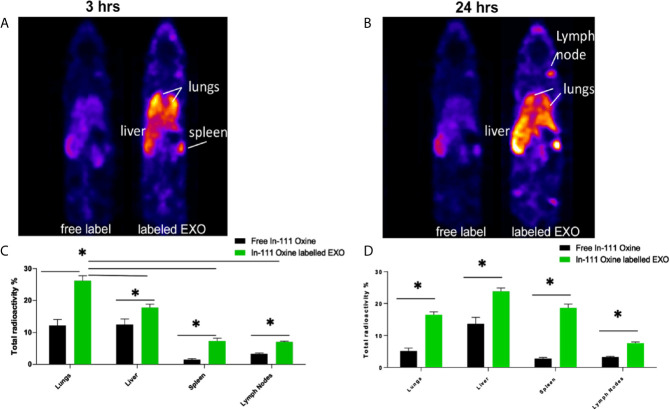
Biodistribution of IV administrated EXO at 3 h and 24 h time points. SPECT CT live animal in vivo imaging of free In-111 (left) or In-111-labeled exosomes (right) in mice after **(A)** 3 h and **(B)** 24 h of IV administration. Radioactivity in lung, liver, spleen, and lymph nodes, relative to total, when free radiolabels or bound to DC EXO, expressed as % determined using SPECT CT images after **(C)** 3 h and **(D)** 24** h** of EXO IV injection. N = 3; *P < 0.05 by two-way ANOVA, followed by Tukey’s multiple comparisons.

Based on proteomic analysis, we predicted that regDCs EXO would be particularly active in cellular trafficking and localization, as well as cell surface receptor binding and immune regulation. We therefore, conducted *in vivo* tracking of regDCs EXO after intravenous injection in live mice. Indium-111-Oxine-labeled-regDCs EXO or free label at equivalent radioactivity was injected *via* tail vein. Three and 24 h after administration, animals were scanned by SPECT/CT and reconstructed images were analyzed and a percentage of biodistribution to the lungs, liver, spleen and lymph nodes was assessed. At the 3hr time point, we observed the highest accumulation of labeled regDC EXO in the lungs (26 ± 2%), followed by the liver (16 ± 2%), spleen (7 ± 1%) and lymph nodes (6 ± .5%), while free label was rapidly dispersed to the liver (**Figures 9A, C**). After 24 h, regDCs EXO were found mainly in the liver, followed by the lungs, spleen and lymph nodes, respectively (**Figures 9B, D**). These data suggest a predilection of regDCs EXO for biodistribution to the lungs shortly after IV injection and they persisted in the lungs for at least 24 hrs. Afterwards, liver clearance appears to predominate.

To confirm these results, animals were euthanized, and organs were harvested and weighed. The emitted gamma activity from the harvested organs was measured to calculate radioactivity per milligram of tissue. Consistent with *in vivo* tracking, *ex-vivo* gamma activity measurements in animals treated with Indium-111-Oxine -labeled regDCs EXO showed gamma activity mostly in the lungs, liver and spleen. The gamma activity in these tissues was significantly higher for Indium-111-Oxine -labeled regDCs EXO in comparison to those treated with free In-111 (**Supplementary Figure 1**).

### Immunoregulatory Dendritic Cell Exosome Uptake Is Essential to Maintain Sustained TGFβ1R-Mediated Signaling in Recipient Cells

**Figure 10:**
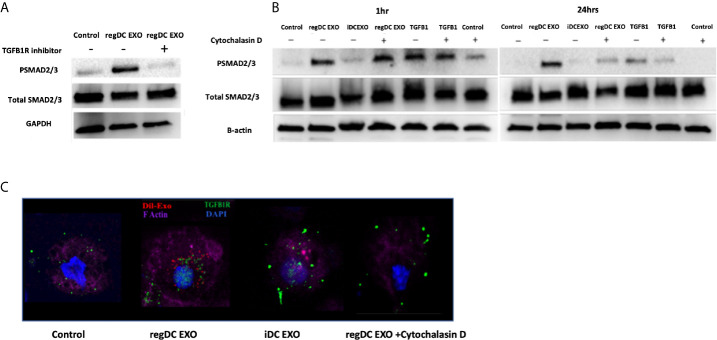
Early and sustained pSMAD2/3 signaling by uptake of regDC EXO with TGFβRI: **(A)** Immunoblot of Psmad2/3 and total smad2/3 in recipient DCs co-cultured for 24 h with reg DCS EXO +/- TGFβ1R inhibitor SB431542. Loading control was GAPDH **(B)** Immunoblot of Psmad2/3 and total smad2/3 in recipient DCs co-cultured for 1 and 24 h with reg DC EXO or iDC EXO +/- cytochalasin D. Loading control was B-actin. **(C)** Uptake of Dil labeled EXO (red) by recipient DCs, DAPI (blue), Alexa Fluor 680 phalloidin (violet) for FActin, Alexa flour 488 (green)-mouse anti-TGFβR1, visualized under confocal microscopy. Dil-DCs EXO or no EXO were added to recipient DCs at a 10:1 EXO : DC ratio (24 h shown). Results shown are representative of three independent experiments.

To further examine mechanisms of action of regDC EXO, we conducted coculture experiments with acceptor DCs using 10^8^/ml Dil-labeled regDCs EXO, iDCs EXO, free TGFβ1 (at an equivalent dosage to that contained within regDCs EXO by ELISA) or control culture media, in the presence or absence of TGFβ1R inhibitors or the uptake inhibitor cytochalasin D (CytoD). Blocking the TGFβ1 receptor with the specific inhibitor SB431542 in the recipient DCs prevented the regDCs EXO mediated activation/phosphorylation of TGFβ1 signaling transcription factor SMAD2/3 after 24 h (**Figure 10A**), suggesting its pivotal role in regDCs EXO mediated TGFβ1 signaling. Uptake of Dil-labeled regDC EXO, along with TGFβ1R, by recipient DCs was documented by immunofluorescence confocal microscopy. In the absence of regDCs EXO, TGFβ1R was extracellular (**Figure 10C**), with no phospho-SMAD2/3 evident at 1 or 24 h (**Figure 10B**). By contrast, upon regDC EXO treatment, TGFβ1R and regDCs EXO were internalized (**Figure 10C**). High phosphorylation of SMAD2/3 induced by regDCs EXO was observed at 1 h and was sustained at the 24 h timepoint (**Figure 10B**). CytoD prevented uptake of regDC EXO (**Figure 10C**) and prevented sustained phospho-SMAD2/3 signaling at 24 h, but not 1 h signaling (**Figure 10B**). Free TGFβ1 activated the 1 h signaling but did not sustain the 24h time point (**Figure 10B**). IDC EXO were internalized, but TGFβ1R was extracellular (**Figure 10C**), with almost no phosphorylation of SMAD2/3 (**Figure 10B**). Collectively, these data suggest optimum and sustained intracellular signaling by regDC EXO involves early binding to TGFβ1R, followed by internalization of TGFβ1R and sustained intercellular SMAD signaling.

### Uptake of Immunoregulatory Dendritic Cell Exosome by Recipient Primary Tracheal/Bronchial Epithelial Cells, Inhibiting ACE2 Expression, via a TGFβ1-Dependent Mechanism

**Figure 11:**
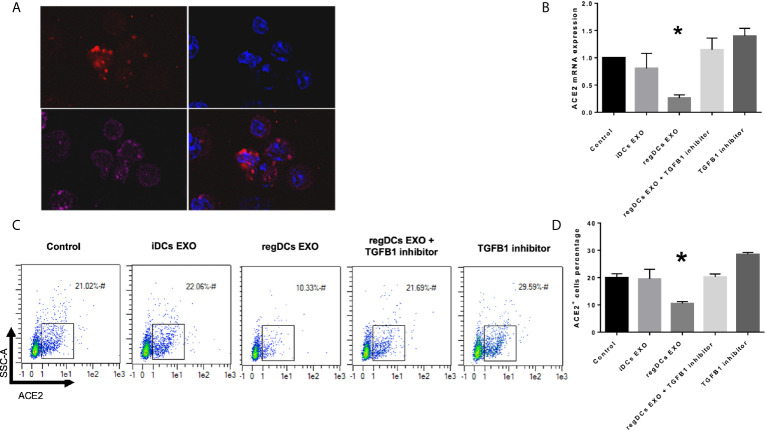
regDCs EXO are taken up by acceptor PBTECs, inhibiting ACE2 expression in vitro. **(A)** Uptake of Dil labeled EXO (red) by PBTECs, counterstained with nuclear stain DAPI (blue), phalloidin (Alex flour 647) for cell membrane and visualized under confocal microscopy. ACE2 mRNA expression **(B)** and flow cytometry scattergrams showing ACE2 positive cells percentage **(C)** in PBTECs treated or not treated with iDCs or regDCs EXO in the presence or absence of TGFβR1 inhibitor SB431542. **(D)** representative bar graph of **(C)**. Results shown are representative of three independent experiments (*P < 0.05 by one-way ANOVA followed by Tukey’s multiple comparisons).

In view of the predilection of regDC EXO for accumulation in lung tissue, and consistent with their protein content, we examined *in vitro* whether regDC EXO are taken up by putative recipient cells of SARS-CoV-2 and how expression of ACE2 (the main SARS-CoV-2 receptor), is influenced. PTBECs were co-cultured with or without 10^8^/ml dil-labeled regDCs EXO, in the presence or absence of TGFβ1R inhibitor for 24 h. PTBECs take up regDCs EXO (**Figure 11A**), commensurate with inhibition of ACE 2 expression (**Figures 11B–D**). Moreover, blocking the TGFβ1 receptor using specific inhibitor SB431542, reduced the inhibitory effect of regDCs EXO on ACE2 levels, suggesting a crucial role for TGFβ1 in regDC EXO-mediated inhibition of ACE 2 expression. This is consistent with previous reports of TGFβ1 cytokine induced inhibition of the ACE2 receptor, the SARS-CoV-2 point of entry (14–16).

## Discussion

Studies of EXO biology, especially of DC origin and their therapeutic applications have rapidly expanded over the last few years (31), revealing promising approaches to reprogram harmful and excessive immune responses (32, 33). Advancing such approaches into therapeutic applications requires more in-depth knowledge of the proteomic cargo, biodistribution and mechanism of action of DC EXO. This will enable investigators to optimize on-target effects and minimize off-target or unintended consequences of such therapy.

We describe the complexity of the protein cargo of DC EXO subtypes, which consist of 1278 shared proteins, and 859,1054 and 634 proteins unique to regDCs EXO, stimDCs EXO and iDCs EXO, respectively (**Figures 2** and **3**). Our study revealed that proteins associated with antigen presentation and processing, phagosome, leukocyte endothelial transmigration and chemokine signaling pathways were common to all DC EXO subtypes, suggesting putative roles in homing to and entry into peripheral tissues from circulation, uptake and modulation of immune responses (**Figure 4**). PDL1 and PDL2, important regulators of the immune response and targets for T cell-based immunotherapy, were identified in all three DCs EXO subtypes. Exosomal proteins including tetraspanins CD63, CD81, CD82, CD9 and those involved in ESCRT complex ALIX and TSG101 were commonly expressed (**Figure 5A**) and further validated by Western blotting and TEM analyses (5). DC markers including CD11c and MHCII, and CD205 were detected in all three EXO subsets, indicative of the parental cells of origin. ICAM1, a positive regulator of leukocyte transmigration* *across the endothelium and a promotor of naive T cells priming and activation (34) was also found to be expressed in all DC EXO.

Apart from TGFβ1 and IL-10, other proteins unique to regDCs EXO included those in the Wnt signaling pathway; namely, Wnt1 and Wnt9, both of which are involved in the regulation of immune tolerance and bone formation (35). Other negative regulators of the immune response were also identified in regDCs EXO such as SHIP1 (36), ILT3B (37), STAT3 (38), ostensibly resulting from parent DC treatment with TGFβ1 or IL10. These negative regulators were also found in stimDC EXO, possibly upregulated in parent mature DC, counter regulatory to overexpression of IL6, TNF, IL1β and IL1a. EXO proteins involved in Toll-like receptor signaling pathway were elevated in iDC EXO, consistent with antigen recognition functions of parent immature DCs (39). Cytokine-cytokine receptor interaction pathway proteins were common to iDCs EXO and stimDCs EXO (**Figure 3**). All DCs EXO subset proteins are detailed in **Supplementary Tables 1–5**.

The predominant expression in regDC EXO of immunoregulatory proteins, as well as proteins involved in trafficking, cell surface binding, and transmigration, sparked our interest in how these EXO might biodistribute to major organs. Many previous studies used either fluorescence imaging or bioluminescence imaging to track administered EXO. Our nuclear imaging approach, has many advantages over these technologies (5, 40–42), including a superior tissue penetration, a higher resolution, a higher signal to noise ratio and an improved sensitivity for deeper organs, thus enabling more accurate imaging (43–45). Blood levels of EXO after IV administration are a dynamic process, and decrease by greater than 50% within 30 minutes of administration (46), with complete elimination by 4 h (43). An initial phase of distribution to the lungs, spleen and liver within minutes, is followed by an elimination phase through the liver and kidney (42, 46, 47). In our study, a significant accumulation of regDC EXO occurred after 3 h in lung tissue, followed by liver, splenic tissue and lymph nodes (**Figures 9A, B**). Significant levels of regDC EXO persisted in lungs for up to 24 h, with a notable increase in hepatic and splenic tissue (**Figures 9C, D**). Ex-vivo gamma radiation measurements of postmortem tissue confirmed the *in vivo* SPECT/CT analysis (**SFig.1**).

Chemokine receptors and integrins and their binding partners shape the homing patterns of cells and ostensibly, EXO in the body (48). Of particular note are the chemotactic and adhesion factors identified in reg DCs EXO (**Figure 6**), including CCR6, CCR7, CXCR2, Integrin alpha-M (CD11b), Integrin beta-1 (CD29), CD47, Integrin alpha-1 (CD49a), ICAM-1(CD54), integrin beta-2, integrin alpha-5, and integrin alpha-L (LFA1) (49–60). CCR7 (50) mediates blood-derived lymphocyte trafficking to bronchial associated lymphoid tissue while CXCR2 directs neutrophil recruitment to the lungs (51). CD49a expression promotes selective trafficking and retention of lymphocytes into respiratory tissues (52–54). LFA-1 and ICAM-1 function by binding lymphocytes to bronchial endothelium cells (55, 56), while CCR6 and integrin beta-2 promote group 2 innate lymphoid cell migration to the lung (57, 58). Neutrophil trafficking in the lung is regulated by integrin beta-1 (CD29), CD47, integrin alpha-M (CD11b) and ICAM-1 (CD54) (59). Integrin beta-1 and integrin alpha-5 are involved in mesenchymal stem cell distribution in the lungs (60). Other investigators have observed a high retention of extracellular vesicles in the lungs 4 h after parenteral administration (42, 43). The accumulation of regDCs EXO observed here in splenic tissue and lymph nodes may be attributed to their interaction with abundant immune cells found in lymphoid tissue. EXO can also bind to lymphocytes, DCs and macrophages which circulate in the bloodstream and migrate to the spleen (61). CCR7 regulates cells or exosomes migration and homing to lymphoid organs and splenic tissue (62). CCR7 was found to be highly expressed in both regDCs EXO and stimDCs EXO. The liver is a large organ and contains a large population of macrophages (Kupffer cells) that can uptake a considerable amount of the injected regDCs EXO for clearance. DCs and hepatocytes can also uptake exosomes. Moreover, EXO express phosphatidylserine (PS) on their surfaces that could enable the recognition and uptake by hepatic phagocytes (42, 61).

Our previous work with these DC EXO subtypes, showed efficacy in regulation of the inflammatory bone disease and periodontitis, when administrated locally in gingival tissue. Locally injected EXO persisted at the site of inflammation and the adjacent lymph nodes, but were minimally cleared to distant tissues such as the lung, spleen and liver (5). The route of administration of EXO is also important to their biodistribution, especially to lymph nodes (63), brain (64) and retina (65). The cell source of EXO and their dose may also affect the biodistribution (66). Adhesion molecules like the tetraspanins, integrins, and chemotactic factors can direct EXO to immune cells found in inflammatory sites (10, 67).

Our previous work showed TGFβ1 in regDCs EXO as a key immunoregulatory factor that recruits T-regulatory cells to target inflamed tissue to deactivate the inflammatory process (5). Thus, we were interested to understand how TGFβ1 when in form of EXO activate the target signaling on acceptor cells. Examination of mechanism of action showed that initial binding of regDC EXO to TGFβR1, stimulated early SMAD2/3 phosphorylation, followed by internalization sustained SMAD2/3 phosphorylation in recipient DCs (**Figure 10**). An equivalent dose of free TGFβ1 could not maintain optimum TGFβ1 signaling. Several reports emphasize the role of endosomal translocation of EXO with TGFβ1-TGFβ1 receptor complex, in order to prevent the lysosomal degradation for prolonged internal SMAD2 signaling (68–70). Also, EXO internalization and TGFβ1 release inside the cell, can lead to TGFβ1 recycling to the cell membrane where it can be secreted and act on the cell surface receptor in an autocrine manner (71).

The present study demonstrated preferential biodistribution and retention of regDCs EXO into lung tissue. It is tempting to speculate that this supports a possible therapeutic implication of regDC EXO, or its cargo, in patients with COVID-19 infection. A significant amount of lung damage in COVID-19 patients is due to an exaggerated host immune response (72–74). In severe stages of COVID-19 infection, high levels of IL-1β, and IL-6, TNF along with decreased levels of antiviral factors (interferons-IFNs) are secreted from respiratory epithelial cells, DCs, macrophages and T cells. The resultant “cytokine storm” culminates in ARDS and multiorgan damage and eventually failure (75–78). Thus, reprogramming inflammatory cells such as DCs and T cells, with regDC EXO as we have described (5) may actually reverse the inflammatory response and attenuate the infectious process and thus diminish the severity of the infection (79). ARDS is an acute inflammatory response in lung tissue and frequently leads to severe damage of tissue and ultimately death in COVID-19 patients (19–23). The use of short-term TGFβ1-loaded regDCs EXO to attenuate the acute inflammation in the lung could prevent the severe lung damage seen during the acute phase and could also decrease the chronic consequences of severe pulmonary COVID-19 infection (80). TGFβ1 has been shown to inhibit early and acute responses upon intranasal lipopolysaccharide (LPS) challenge in an acute lung injury model (81). TGFβ1 suppressed neutrophils and induced Foxp3^+^r regulatory T cell responses needed to resolve acute inflammation in the lungs (79). Given the acute inflammatory nature of severe COVID-19 infection, regDCs EXO may therefore represent a natural nano-therapy modality. Furthermore, this may be also alleviate the need to use concurrent anti-inflammatory agents such as hydroxychloroquine and dexamethasone (26). Moreover, this may overcome limitations of immunoregulatory cell-based therapy such as phenotypic instability and low cell numbers (9). There are currently several clinical trials registered in clinicaltrials.gov using extracellular vesicles (EVs) as therapy for COVID-19. Extracellular vesicles from mesenchymal stem cells reportedly retain their ability to inhibit lung inflammation by shifting proinflammatory monocytes/macrophages toward the regulatory phenotype in an ARDS model. This is presumably done through reprograming the lung-infiltrating DCs and T cells toward a regulatory phenotype (82) as has been described in the oral mucosa (5).

In addition to immunoregulatory functions (5) and unique proteins expressed by regDC EXO, we investigated their ability to regulate expression of the SARS-COV-2 receptor, ACE2 (25, 26). This was particularly pertinent in view of recent findings linking TGFβ1 and ACE 2 to SARS-COV-2 internalization (14–16). In our study, regDCs EXO were shown to inhibit ACE2 expression in respiratory tract epithelial cells (PBTECs), which was abrogated by TGFβ1R blockers (**Figure 11**).

DCs EXO have many therapeutic advantages. These include protection of cargo against proteolytic degradation and damage by complement system, active migration, and localization to target lung tissue, affinity for interaction with immune cells and the capability to be loaded with therapeutic and diagnostic factors (5, 9–11, 83). Mention should be made of other EXO based approaches proposed for COVID-19, including EXO tailored to express decoy ACE2 (84), to encapsulate the S protein of the SARS-CoV-2, and used as a vaccine (85), or to encapsulate antiviral drugs (86).

In conclusion, our study shows that although regDC EXO contain a complex profile of proteins, their functions appear reflective of the dominant proteins in the parent donor DCs. These include proteins that mediate trafficking to inflamed tissue, cell binding and retention in lung tissue. In addition, the impact of regDCs EXO on regulating TGFβ1 cargo signaling and the main SARS-CoV-2 receptors expression such as ACE2 was shown. Overall, the capabilities of regDC EXO may suggest their future utility as an immunotherapeutic modality to improve the treatment of lung inflammatory diseases. Preclinical studies using ARDS animal models and early clinical trials are still necessary to evaluate the safety and efficacy of EXO from DCs.

## Data Availability Statement

The raw data supporting the conclusions of this article will be made available by the authors, without undue reservation.

## Ethics Statement

The animal study was reviewed and approved by the Institutional Animal Care and Use Committee (IACUC) of Augusta University (protocol # 2013-0586).

## Author Contributions

ME and RE conceived and designed the research, performed the experiments, analyzed the data, interpreted the results, prepared the figures, drafted the manuscript, and edited and revised the manuscript. MME, RA, MR, WZ, AE, and AA analyzed the data and interpreted the results. CC analyzed the data, interpreted the results, prepared the figures, drafted the manuscript, and edited and revised the manuscript. MH, YL, JV, and RL edited and revised the manuscript. All authors contributed to the article and approved the submitted version.

## Funding

This study was supported by the Carlos and Marguerite Mason Trust grant. RL was funded by NHLBI grant HL138410.

## Conflict of Interest

The authors declare that the research was conducted in the absence of any commercial or financial relationships that could be construed as a potential conflict of interest.
