# Supplementary material for: Proteomic Characterization, Biodistribution, and Functional Studies of Immune-Therapeutic Exosomes: Implications for Inflammatory Lung Diseases
Source: Front Immunol. 2021 Mar 25;12:636222. doi: 10.3389/fimmu.2021.636222 (PMC8027247; doi:10.3389/fimmu.2021.636222)
Supplement: Supplementary file 1 [file DataSheet_1.pdf]

Table 1. Proteins unique to regDCs EXO.

Accession	Entry name	Protein names
A0JP43	EFCB5_MOUSE	EF-hand calcium-binding domain-containing protein 5
A2A5R2	BIG2_MOUSE	Brefeldin A-inhibited guanine nucleotide-exchange protein 2 (Brefeldin A-inhibited GEP 2) (ADP-ribosylation factor guanine nucleotide-exchange factor 2)
A2A863	ITB4_MOUSE	Integrin beta-4 (CD antigen CD104)
A2AAY5	SPD2B_MOUSE	SH3 and PX domain-containing protein 2B (Factor for adipocyte differentiation 49) (Tyrosine kinase substrate with four SH3 domains)
A2AH22	AMRA1_MOUSE	Activating molecule in BECN1-regulated autophagy protein 1
A2AI05	NDOR1_MOUSE	NADPH-dependent diflavin oxidoreductase 1 (EC 1.18.1.-) (NADPH-dependent FMN and FAD-containing oxidoreductase)
A2AI08	TPRN_MOUSE	Taperin
A2AJA9	AJM1_MOUSE	Apical junction component 1 homolog
A2AMM0	CAVN4_MOUSE	Caveolae-associated protein 4 (Muscle-related coiled-coil protein) (Muscle-restricted coiled-coil protein)
A2APF3	CTCFL_MOUSE	Transcriptional repressor CTCFL (Brother of the regulator of imprinted sites) (CCCTC-binding factor) (CTCF paralog) (CTCF-like protein)
A2AWT3	AT7L3_MOUSE	Ataxin-7-like protein 3 (SAGA-associated factor 11 homolog)
A2BDP1	F155B_MOUSE	Transmembrane protein FAM155B (Protein TED) (Transmembrane protein 28)
A2BH40	ARI1A_MOUSE	AT-rich interactive domain-containing protein 1A (ARID domain-containing protein 1A) (BRG1-associated factor 250) (BAF250) (BRG1-associated factor 250a) (BAF250A) (Osa homolog 1) (SWI-like protein) (SWI/SNF complex protein p270) (SWI/SNF-related, matrix-associated, actin-dependent regulator of chromatin subfamily F member 1)
A2RSJ4	UH1BL_MOUSE	UHRF1-binding protein 1-like (Syntaxin-6 Habc-interacting protein of 164 kDa)
A2RTF1	CTSRB_MOUSE	Cation channel sperm-associated protein subunit beta (CatSper-beta)
A3KGB4	TBC8B_MOUSE	TBC1 domain family member 8B
A6H690	IQCAL_MOUSE	IQ and AAA domain-containing protein 1-like (Protein IQCA1P1)

A6H694	LRC63_MOUSE	Leucine-rich repeat-containing protein 63
A6X919	D19L1_MOUSE	Probable C-mannosyltransferase DPY19L1 (EC 2.4.1.-) (Dpy-19-like protein 1) (Protein dpy-19 homolog 1)
A7L9Z8	AT2C2_MOUSE	Calcium-transporting ATPase type 2C member 2 (ATPase 2C2) (EC 7.2.2.10) (Secretory pathway Ca(2+)-ATPase 2)
B0V2N1	PTPRS_MOUSE	Receptor-type tyrosine-protein phosphatase S (R-PTP-S) (EC 3.1.3.48) (PTPNU-3) (Receptor-type tyrosine-protein phosphatase sigma) (R-PTP-sigma)
B1AVH7	TBD2A_MOUSE	TBC1 domain family member 2A
B1AZP2	DLGP4_MOUSE	Disks large-associated protein 4 (DAP-4) (PSD-95/SAP90-binding protein 4) (SAP90/PSD-95-associated protein 4) (SAPAP-4)
B2RUP2	UN13D_MOUSE	Protein unc-13 homolog D (Munc13-4)
B2RUR8	OTU7B_MOUSE	OTU domain-containing protein 7B (EC 3.4.19.12) (Cellular zinc finger anti-NF-kappa-B protein) (Zinc finger A20 domain-containing protein 1) (Zinc finger protein Cezanne)
B7ZCC9	AGRG4_MOUSE	Adhesion G-protein coupled receptor G4 (G-protein coupled receptor 112)
B7ZNG4	TROAP_MOUSE	Tastin (Troap protein) (Trophinin-assisting protein) (Trophinin-associated protein)
B9EJ57	MTF1B_MOUSE	Transcription termination factor 1b, mitochondrial (Mitochondrial transcription termination factor 1b) (mTERF1b)
B9EJ86	OSBL8_MOUSE	Oxysterol-binding protein-related protein 8 (ORP-8) (OSBP-related protein 8)
B9EKR1	PTPRZ_MOUSE	Receptor-type tyrosine-protein phosphatase zeta (R-PTP-zeta) (EC 3.1.3.48)
E9PZM4	CHD2_MOUSE	Chromodomain-helicase-DNA-binding protein 2 (CHD-2) (EC 3.6.4.12) (ATP-dependent helicase CHD2)
E9Q173	BTBDG_MOUSE	BTB/POZ domain-containing protein 16
E9Q1U1	CC171_MOUSE	Coiled-coil domain-containing protein 171
E9Q735	UBE4A_MOUSE	Ubiquitin conjugation factor E4 A (EC 2.3.2.-)
E9Q7F2	RN169_MOUSE	E3 ubiquitin-protein ligase RNF169 (EC 2.3.2.27) (RING finger protein 169) (RING-type E3 ubiquitin transferase RNF169)
G5E8K5	ANK3_MOUSE	Ankyrin-3 (ANK-3) (Ankyrin-G)
O08582	GTPB1_MOUSE	GTP-binding protein 1 (G-protein 1) (GP-1) (GP1)
O08689	GDF8_MOUSE	Growth/differentiation factor 8 (GDF-8) (Myostatin)

O08858	SSR5_MOUSE	Somatostatin receptor type 5 (SS-5-R) (SS5-R) (SS5R)
O08915	AIP_MOUSE	AH receptor-interacting protein (AIP) (Aryl-hydrocarbon receptor-interacting protein)
O08999	LTBP2_MOUSE	Latent-transforming growth factor beta-binding protein 2 (LTBP-2)
O35085	ARX_MOUSE	Homeobox protein ARX (Aristaless-related homeobox)
O35098	DPYL4_MOUSE	Dihydropyrimidinase-related protein 4 (DRP-4) (Collapsin response mediator protein 3) (CRMP-3) (UNC33-like phosphoprotein 4) (ULIP-4)
O35134	RPA1_MOUSE	DNA-directed RNA polymerase I subunit RPA1 (RNA polymerase I subunit A1) (EC 2.7.7.6) (DNA-directed RNA polymerase I largest subunit) (DNA-directed RNA polymerase I subunit A) (RNA polymerase I 194 kDa subunit) (RPA194)
O35164	MCPT9_MOUSE	Mast cell protease 9 (mMCP-9) (EC 3.4.21.-)
O35177	CASL_MOUSE	Enhancer of filamentation 1 (mEF1) (CRK-associated substrate-related protein) (CAS-L) (Neural precursor cell expressed developmentally down-regulated protein 9) (NEDD-9) (p105)
O35181	NRG3_MOUSE	Pro-neuregulin-3, membrane-bound isoform (Pro-NRG3) [Cleaved into: Neuregulin-3 (NRG-3)]
O35206	COFA1_MOUSE	Collagen alpha-1(XV) chain [Cleaved into: Restin (Endostatin-XV)]
O35405	PLD3_MOUSE	5'-3' exonuclease PLD3 (EC 3.1.16.1) (Choline phosphatase 3) (Phosphatidylcholine-hydrolyzing phospholipase D3) (Phospholipase D3) (PLD 3) (Schwannoma-associated protein 9) (SAM-9)
O35409	FOLH1_MOUSE	Glutamate carboxypeptidase 2 (EC 3.4.17.21) (Folate hydrolase 1) (Folylpoly-gamma-glutamate carboxypeptidase) (FGCP) (Glutamate carboxypeptidase II) (GCPII) (Membrane glutamate carboxypeptidase) (mGCP) (N-acetylated-alpha-linked acidic dipeptidase I) (NAALADase I) (Prostate-specific membrane antigen homolog) (Pteroylpoly-gamma-glutamate carboxypeptidase)
O35454	CLCN6_MOUSE	Chloride transport protein 6 (Chloride channel protein 6) (ClC-6)
O35468	WNT9B_MOUSE	Protein Wnt-9b (Protein Wnt-14b) (Protein Wnt-15)
O35488	S27A2_MOUSE	Very long-chain acyl-CoA synthetase (VLACS) (VLCS) (EC 6.2.1.-) (Arachidonate--CoA ligase) (EC 6.2.1.15) (Fatty acid transport protein 2) (FATP-2) (Fatty-acid-coenzyme A ligase, very

		long-chain 1) (Long-chain-fatty-acid--CoA ligase) (EC 6.2.1.3) (Phytanate--CoA ligase) (EC 6.2.1.24) (Solute carrier family 27 member 2) (THCA-CoA ligase) (EC 6.2.1.7) (Very long-chain-fatty-acid-CoA ligase)
O35599	OPSG_MOUSE	Medium-wave-sensitive opsin 1 (Green cone photoreceptor pigment) (Green-sensitive opsin) (M opsin) (Medium wavelength-sensitive cone opsin)
O35602	RX_MOUSE	Retinal homeobox protein Rx (Retina and anterior neural fold homeobox protein)
O35655	PPE1_MOUSE	Serine/threonine-protein phosphatase with EF-hands 1 (PPEF-1) (EC 3.1.3.16) (DRES10) (Protein phosphatase with EF calcium-binding domain) (PPEF)
O35927	CTND2_MOUSE	Catenin delta-2 (Neural plakophilin-related ARM-repeat protein) (NPRAP) (Neurojungin)
O54689	CCR6_MOUSE	C-C chemokine receptor type 6 (C-C CKR-6) (CC-CKR-6) (CCR-6) (KY411) (CD antigen CD196)
O54788	DFFB_MOUSE	DNA fragmentation factor subunit beta (EC 3.-.-.-) (Caspase-activated deoxyribonuclease) (CAD) (Caspase-activated DNase) (DNA fragmentation factor 40 kDa subunit) (DFF-40)
O54826	AF10_MOUSE	Protein AF-10
O54834	RHG06_MOUSE	Rho GTPase-activating protein 6 (Rho-type GTPase-activating protein 6) (Rho-type GTPase-activating protein RhoGAPX-1)
O54863	TSSK2_MOUSE	Testis-specific serine/threonine-protein kinase 2 (TSK-2) (TSK2) (TSSK-2) (Testis-specific kinase 2) (EC 2.7.11.1) (Serine/threonine-protein kinase 22B)
O54943	PER2_MOUSE	Period circadian protein homolog 2 (mPER2) (Circadian clock protein PERIOD 2)
O54972	MTG16_MOUSE	Protein CBFA2T3 (Eight twenty one protein 2) (MTG8-related protein 2) (Protein ETO-2)
O54990	PROM1_MOUSE	Prominin-1 (Antigen AC133 homolog) (Prominin-like protein 1) (CD antigen CD133)
O55022	PGRC1_MOUSE	Membrane-associated progesterone receptor component 1 (mPR)
O55028	BCKD_MOUSE	[3-methyl-2-oxobutanoate dehydrogenase [lipoamide]] kinase, mitochondrial (EC 2.7.11.4) (Branched-chain alpha-ketoacid dehydrogenase kinase) (BCKD-kinase) (BCKDHKIN)
O55134	PCD12_MOUSE	Protocadherin-12 (Vascular cadherin-2) (Vascular endothelial cadherin-2) (VE-cad-2) (VE-cadherin-2) [Cleaved into: Protocadherin-12, secreted form]

O70167	P3C2G_MOUSE	Phosphatidylinositol 4-phosphate 3-kinase C2 domain-containing subunit gamma (PI3K-C2-gamma) (PtdIns-3-kinase C2 subunit gamma) (EC 2.7.1.154) (Phosphoinositide 3-kinase-C2-gamma)
O70306	TBX15_MOUSE	T-box transcription factor TBX15 (T-box protein 15) (MmTBx8) (T-box transcription factor TBX14) (T-box protein 14)
O70324	MOT8_MOUSE	Monocarboxylate transporter 8 (MCT 8) (Solute carrier family 16 member 2) (X-linked PEST-containing transporter)
O70410	V2R1_MOUSE	Vomeronasal type-2 receptor 1 (Putative pheromone receptor V2R1) (V2Rx)
O70493	SNX12_MOUSE	Sorting nexin-12 (SDP8 protein)
O70571	PDK4_MOUSE	[Pyruvate dehydrogenase (acetyl-transferring)] kinase isozyme 4, mitochondrial (EC 2.7.11.2) (Pyruvate dehydrogenase kinase isoform 4)
O70583	TRI18_MOUSE	E3 ubiquitin-protein ligase Midline-1 (EC 2.3.2.27) (Midin) (RING finger protein Midline-1) (RING-type E3 ubiquitin transferase Midline-1) (Tripartite motif-containing protein 18)
O88286	WIZ_MOUSE	Protein Wiz (Widely-interspaced zinc finger-containing protein)
O88324	CD83_MOUSE	CD83 antigen (mCD83) (CD antigen CD83)
O88338	CAD16_MOUSE	Cadherin-16 (Kidney-specific cadherin) (Ksp-cadherin)
O88533	DDC_MOUSE	Aromatic-L-amino-acid decarboxylase (AADC) (EC 4.1.1.28) (DOPA decarboxylase) (DDC)
O88811	STAM2_MOUSE	Signal transducing adapter molecule 2 (STAM-2) (Hrs-binding protein)
O88866	HUNK_MOUSE	Hormonally up-regulated neu tumor-associated kinase (EC 2.7.11.1) (Serine/threonine-protein kinase MAK-V)
O88875	BY55_MOUSE	CD160 antigen (Natural killer cell receptor BY55) (CD antigen CD160) [Cleaved into: CD160 antigen, soluble form]
O89016	ABCD4_MOUSE	ATP-binding cassette sub-family D member 4 (PMP70-related protein) (P70R) (Peroxisomal membrane protein 1-like) (PXMP1-L) (Peroxisomal membrane protein 69) (PMP69)
P00683	RNAS1_MOUSE	Ribonuclease pancreatic (EC 4.6.1.18) (RNase 1) (RNase A)
P01587	CSF2_MOUSE	Granulocyte-macrophage colony-stimulating factor (GM-CSF) (Colony-stimulating factor) (CSF)
P01592	IGJ_MOUSE	Immunoglobulin J chain

P03940	CP21A_MOUSE	Steroid 21-hydroxylase (EC 1.14.14.16) (21-OHase) (Cytochrome P-450c21) (Cytochrome P450 21) (Cytochrome P450 XXI) (Cytochrome P450-C21)
P04202	TGFB1_MOUSE	Transforming growth factor beta-1 proprotein [Cleaved into: Latency-associated peptide (LAP); Transforming growth factor beta-1 (TGF-beta-1)]
P04426	WNT1_MOUSE	Proto-oncogene Wnt-1 (Proto-oncogene Int-1)
P04444	HBBZ_MOUSE	Hemoglobin subunit beta-H1 (Beta-H1-globin) (Hemoglobin beta-H1 chain) (Protein Z)
P06240	LCK_MOUSE	Proto-oncogene tyrosine-protein kinase LCK (EC 2.7.10.2) (Leukocyte C-terminal Src kinase) (LSK) (Lymphocyte cell-specific protein-tyrosine kinase) (p56-LCK)
P06683	CO9_MOUSE	Complement component C9
P07750	IL4_MOUSE	Interleukin-4 (IL-4) (B-cell IgG differentiation factor) (B-cell growth factor 1) (B-cell stimulatory factor 1) (BSF-1) (IGG1 induction factor) (Lymphocyte stimulatory factor 1)
P07759	SPA3K_MOUSE	Serine protease inhibitor A3K (Serpin A3K) (Contrapsin) (SPI-2)
P08775	RPB1_MOUSE	DNA-directed RNA polymerase II subunit RPB1 (RNA polymerase II subunit B1) (EC 2.7.7.6) (DNA-directed RNA polymerase II subunit A) (DNA-directed RNA polymerase III largest subunit)
P10711	TCEA1_MOUSE	Transcription elongation factor A protein 1 (Transcription elongation factor S-II protein 1) (Transcription elongation factor TFIIS.o)
P11214	TPA_MOUSE	Tissue-type plasminogen activator (t-PA) (t-plasminogen activator) (tPA) (EC 3.4.21.68) [Cleaved into: Tissue-type plasminogen activator chain A; Tissue-type plasminogen activator chain B]
P11440	CDK1_MOUSE	Cyclin-dependent kinase 1 (CDK1) (EC 2.7.11.22) (EC 2.7.11.23) (Cell division control protein 2 homolog) (Cell division protein kinase 1) (p34 protein kinase)
P11609	CD1D1_MOUSE	Antigen-presenting glycoprotein CD1d1 (CD antigen CD1d.1)
P11627	L1CAM_MOUSE	Neural cell adhesion molecule L1 (N-CAM-L1) (NCAM-L1) (CD antigen CD171)
P11679	K2C8_MOUSE	Keratin, type II cytoskeletal 8 (Cytokeratin endo A) (Cytokeratin-8) (CK-8) (Keratin-8) (K8) (Type-II keratin Kb8)

P13439	UMPS_MOUSE	Uridine 5'-monophosphate synthase (UMP synthase) [Includes: Orotate phosphoribosyltransferase (OPRTase) (EC 2.4.2.10); Orotidine 5'-phosphate decarboxylase (EC 4.1.1.23) (OMPdecase)]
P13595	NCAM1_MOUSE	Neural cell adhesion molecule 1 (N-CAM-1) (NCAM-1) (CD antigen CD56)
P15037	ETS2_MOUSE	Protein C-ets-2
P15392	CP2A4_MOUSE	Cytochrome P450 2A4 (EC 1.14.14.1) (CYPIIA4) (Cytochrome P450-15-alpha) (Cytochrome P450-IIA3.1) (Testosterone 15-alpha-hydroxylase)
P16305	GBRA6_MOUSE	Gamma-aminobutyric acid receptor subunit alpha-6 (GABA(A) receptor subunit alpha-6)
P17532	TPH1_MOUSE	Tryptophan 5-hydroxylase 1 (EC 1.14.16.4) (Tryptophan 5-monooxygenase 1)
P18893	IL10_MOUSE	Interleukin-10 (IL-10) (Cytokine synthesis inhibitory factor) (CSIF)
P20108	PRDX3_MOUSE	Thioredoxin-dependent peroxide reductase, mitochondrial (EC 1.11.1.15) (Antioxidant protein 1) (AOP-1) (PRX III) (Perioredoxin-3) (Protein MER5)
P20863	GDF1_MOUSE	Embryonic growth/differentiation factor 1 (GDF-1)
P22091	TAL1_MOUSE	T-cell acute lymphocytic leukemia protein 1 homolog (TAL-1) (Stem cell protein)
P22858	PTHR_MOUSE	Parathyroid hormone-related protein (PTH-rP) (PTHrP) (Parathyroid hormone-like protein) (PLP) [Cleaved into: Osteostatin]
P24788	CD11B_MOUSE	Cyclin-dependent kinase 11B (Cell division cycle 2-like protein kinase 1) (Cell division protein kinase 11) (Cyclin-dependent kinase 11) (EC 2.7.11.22) (Galactosyltransferase-associated protein kinase p58/GTA) (PITSLRE serine/threonine-protein kinase CDC2L1)
P25233	NECD_MOUSE	Necdin
P27046	MA2A1_MOUSE	Alpha-mannosidase 2 (EC 3.2.1.114) (Golgi alpha-mannosidase II) (AMan II) (Man II) (Mannosidase alpha class 2A member 1) (Mannosyl-oligosaccharide 1,3-1,6-alpha-mannosidase)
P27664	PDE6A_MOUSE	Rod cGMP-specific 3',5'-cyclic phosphodiesterase subunit alpha (GMP-PDE alpha) (EC 3.1.4.35)
P28230	CXB1_MOUSE	Gap junction beta-1 protein (Connexin-32) (Cx32)
P28301	LYOX_MOUSE	Protein-lysine 6-oxidase (EC 1.4.3.13) (Lysyl oxidase) (Ras excision protein) [Cleaved into: Protein-lysine 6-oxidase, long form; Protein-lysine 6-oxidase, short form]

P28575	IPP_MOUSE	Actin-binding protein IPP (Intracisternal A particle-promoted polypeptide) (IPP) (Murine IAP-promoted placenta-expressed protein) (Protein MIPP)
P28665	MUG1_MOUSE	Murinoglobulin-1 (MuG1)
P30276	CCNB2_MOUSE	G2/mitotic-specific cyclin-B2
P30545	ADA2B_MOUSE	Alpha-2B adrenergic receptor (Alpha-2B adrenoreceptor) (Alpha-2B adrenoceptor) (Alpha-2BAR)
P31266	SUH_MOUSE	Recombining binding protein suppressor of hairless (J kappa-recombination signal-binding protein) (RBP-J kappa)
P33611	DPOA2_MOUSE	DNA polymerase alpha subunit B (DNA polymerase alpha 70 kDa subunit)
P35235	PTN11_MOUSE	Tyrosine-protein phosphatase non-receptor type 11 (EC 3.1.3.48) (Protein-tyrosine phosphatase SYP) (SH-PTP2) (SHP-2) (Shp2)
P35436	NMDE1_MOUSE	Glutamate receptor ionotropic, NMDA 2A (GluN2A) (Glutamate [NMDA] receptor subunit epsilon-1) (N-methyl D-aspartate receptor subtype 2A) (NMDAR2A) (NR2A)
P35550	FBRL_MOUSE	rRNA 2'-O-methyltransferase fibrillarin (EC 2.1.1.-) (Histone-glutamine methyltransferase) (Nucleolar protein 1)
P35689	ERCC5_MOUSE	DNA repair protein complementing XP-G cells homolog (EC 3.1.-.-) (DNA excision repair protein ERCC-5) (Xeroderma pigmentosum group G-complementing protein homolog)
P35761	TTK_MOUSE	Dual specificity protein kinase TTK (EC 2.7.12.1) (ESK) (PYT)
P37889	FBLN2_MOUSE	Fibulin-2 (FIBL-2)
P39061	COIA1_MOUSE	Collagen alpha-1(XVIII) chain [Cleaved into: Endostatin; Non-collagenous domain 1 (NC1)]
P41969	ELK1_MOUSE	ETS domain-containing protein Elk-1
P43006	EAA2_MOUSE	Excitatory amino acid transporter 2 (GLT-1) (Sodium-dependent glutamate/aspartate transporter 2) (Solute carrier family 1 member 2)
P43252	PI2R_MOUSE	Prostacyclin receptor (Prostaglandin I2 receptor) (PGI receptor) (PGI2 receptor) (Prostanoid IP receptor)
P46096	SYT1_MOUSE	Synaptotagmin-1 (Synaptotagmin I) (SytI) (p65)
P46935	NEDD4_MOUSE	E3 ubiquitin-protein ligase NEDD4 (EC 2.3.2.26) (HECT-type E3 ubiquitin transferase NEDD4) (Neural precursor cell expressed developmentally down-regulated protein 4) (NEDD-4)

P46978	STT3A_MOUSE	Dolichyl-diphosphooligosaccharide--protein glycosyltransferase subunit STT3A (Oligosaccharyl transferase subunit STT3A) (STT3-A) (EC 2.4.99.18) (B5) (Integral membrane protein 1)
P47867	SCG3_MOUSE	Secretogranin-3 (Secretogranin III) (SgIII)
P47968	RPIA_MOUSE	Ribose-5-phosphate isomerase (EC 5.3.1.6) (Phosphoriboisomerase)
P48299	EDN3_MOUSE	Endothelin-3 (ET-3) (Preproendothelin-3) (PPET3)
P48377	RFX1_MOUSE	MHC class II regulatory factor RFX1 (Regulatory factor X 1) (Transcription factor RFX1)
P48678	LMNA_MOUSE	Prelamin-A/C [Cleaved into: Lamin-A/C]
P50462	CSRP3_MOUSE	Cysteine and glycine-rich protein 3 (Cysteine-rich protein 3) (CRP3) (LIM domain protein, cardiac) (Muscle LIM protein)
P51829	ADCY7_MOUSE	Adenylate cyclase type 7 (EC 4.6.1.1) (ATP pyrophosphate-lyase 7) (Adenylate cyclase type VII) (Adenylyl cyclase 7)
P52196	THTR_MOUSE	Thiosulfate sulfurtransferase (EC 2.8.1.1) (Rhodanese)
P52293	IMA1_MOUSE	Importin subunit alpha-1 (Importin alpha P1) (Karyopherin subunit alpha-2) (Pendulin) (Pore targeting complex 58 kDa subunit) (PTAC58) (RAG cohort protein 1) (SRP1-alpha)
P52734	FGD1_MOUSE	FYVE, RhoGEF and PH domain-containing protein 1 (Faciogenital dysplasia 1 protein homolog) (Rho/Rac guanine nucleotide exchange factor FGD1) (Rho/Rac GEF) (Zinc finger FYVE domain-containing protein 3)
P52850	NDST2_MOUSE	Bifunctional heparan sulfate N-deacetylase/N-sulfotransferase 2 (EC 2.8.2.8) (Glucosaminyl N-deacetylase/N-sulfotransferase 2) (NDST-2) (Mndns) (N-heparan sulfate sulfotransferase 2) (N-HSST 2) [Includes: Heparan sulfate N-deacetylase 2 (EC 3.-.-.-); Heparan sulfate N-sulfotransferase 2 (EC 2.8.2.-)]
P53798	FDFT_MOUSE	Squalene synthase (SQS) (SS) (EC 2.5.1.21) (FPP:FPP farnesyltransferase) (Farnesyl-diphosphate farnesyltransferase)
P54818	GALC_MOUSE	Galactocerebrosidase (GALCERase) (EC 3.2.1.46) (Galactocerebroside beta-galactosidase) (Galactosylceramidase) (Galactosylceramide beta-galactosidase)
P54841	MAFB_MOUSE	Transcription factor MafB (Maf-B) (Kreisler) (Segmentation protein Kr) (Transcription factor Maf-1) (V-maf musculoaponeurotic fibrosarcoma oncogene homolog B)

P55014	S12A1_MOUSE	Solute carrier family 12 member 1 (BSC1) (Bumetanide-sensitive sodium-(potassium)-chloride cotransporter 2) (Kidney-specific Na-K-Cl symporter)
P56198	CIDEC_MOUSE	Cell death activator CIDE-3 (Cell death-inducing DFFA-like effector protein C) (Fat-specific protein FSP27)
P56469	CORT_MOUSE	Cortistatin [Cleaved into: Cortistatin-14]
P57725	SAMN1_MOUSE	SAM domain-containing protein SAMSN-1 (SAM domain, SH3 domain and nuclear localization signals protein 1) (SH3 protein expressed in lymphocytes 2) (SH3-lymphocyte protein 2) (SLy2)
P57748	MMP20_MOUSE	Matrix metalloproteinase-20 (MMP-20) (EC 3.4.24.-) (Enamel metalloproteinase) (Enamelysin)
P58137	ACOT8_MOUSE	Acyl-coenzyme A thioesterase 8 (Acyl-CoA thioesterase 8) (EC 3.1.2.1) (EC 3.1.2.11) (EC 3.1.2.2) (EC 3.1.2.3) (EC 3.1.2.5) (Choloyl-coenzyme A thioesterase) (EC 3.1.2.27) (Peroxisomal acyl-CoA thioesterase 2) (PTE-2) (Peroxisomal acyl-coenzyme A thioester hydrolase 1) (PTE-1) (Peroxisomal long-chain acyl-CoA thioesterase 1)
P58307	OX1R_MOUSE	Orexin receptor type 1 (Ox-1-R) (Ox1-R) (Ox1R) (Hypocretin receptor type 1)
P58404	STRN4_MOUSE	Striatin-4 (Zinedin)
P58466	CTDS1_MOUSE	Carboxy-terminal domain RNA polymerase II polypeptide A small phosphatase 1 (EC 3.1.3.16) (Golli-interacting protein) (GIP) (Nuclear LIM interactor-interacting factor 3) (NLI-interacting factor 3) (Small C-terminal domain phosphatase 1) (SCP1) (Small CTD phosphatase 1)
P58545	BTBD3_MOUSE	BTB/POZ domain-containing protein 3
P58660	CAR10_MOUSE	Caspase recruitment domain-containing protein 10 (Bcl10-interacting MAGUK protein 1) (Bimp1)
P59111	KCNH8_MOUSE	Potassium voltage-gated channel subfamily H member 8 (Ether-a-go-go-like potassium channel 3) (ELK channel 3) (ELK3) (Voltage-gated potassium channel subunit Kv12.1)
P59759	MRTFB_MOUSE	Myocardin-related transcription factor B (MRTF-B) (MKL/myocardin-like protein 2)
P59900	EMIL3_MOUSE	EMILIN-3 (EMILIN-5) (Elastin microfibril interface located protein 5) (Elastin microfibril interfacer 5) (Elastin microfibril interface-located protein 3) (Elastin microfibril interfacer 3)

P61222	ABCE1_MOUSE	ATP-binding cassette sub-family E member 1 (RNase L inhibitor) (Ribonuclease 4 inhibitor) (RNS4I)
P61620	S61A1_MOUSE	Protein transport protein Sec61 subunit alpha isoform 1 (Sec61 alpha-1)
P61963	DCAF7_MOUSE	DDB1- and CUL4-associated factor 7 (WD repeat-containing protein 68) (WD repeat-containing protein An11 homolog)
P62500	T22D1_MOUSE	TSC22 domain family protein 1 (Regulatory protein TSC-22) (TGFB-stimulated clone 22 homolog) (TSC22-related inducible leucine zipper 1b) (Transforming growth factor beta-1-induced transcript 4 protein)
P62509	ERR3_MOUSE	Estrogen-related receptor gamma (Estrogen receptor-related protein 3) (Nuclear receptor subfamily 3 group B member 3)
P62911	RL32_MOUSE	60S ribosomal protein L32
P67778	PHB_MOUSE	Prohibitin (B-cell receptor-associated protein 32) (BAP 32)
P70170	ABCC9_MOUSE	ATP-binding cassette sub-family C member 9 (Sulfonylurea receptor 2)
P70193	LRIG1_MOUSE	Leucine-rich repeats and immunoglobulin-like domains protein 1 (LIG-1)
P70227	ITPR3_MOUSE	Inositol 1,4,5-trisphosphate receptor type 3 (IP3 receptor isoform 3) (IP3R 3) (InsP3R3) (Type 3 inositol 1,4,5-trisphosphate receptor) (Type 3 InsP3 receptor)
P70335	ROCK1_MOUSE	Rho-associated protein kinase 1 (EC 2.7.11.1) (Rho-associated, coiled-coil-containing protein kinase 1) (Rho-associated, coiled-coil-containing protein kinase I) (ROCK-I) (p160 ROCK-1) (p160ROCK)
P70403	CASP_MOUSE	Protein CASP
P70458	IPSP_MOUSE	Plasma serine protease inhibitor (Plasminogen activator inhibitor 3) (PAI-3) (PAI3) (Protein C inhibitor) (PCI) (Serpin A5)
P70669	PHEX_MOUSE	Phosphate-regulating neutral endopeptidase PHEX (Metalloendopeptidase homolog PEX) (EC 3.4.24.-) (Phosphate regulating neutral endopeptidase) (Vitamin D-resistant hypophosphatemic rickets protein) (X-linked hypophosphatemia protein) (HYP)
P70700	RPA2_MOUSE	DNA-directed RNA polymerase I subunit RPA2 (RNA polymerase I subunit 2) (EC 2.7.7.6) (DNA-directed RNA polymerase I 135 kDa polypeptide) (RPA135)

P83626	TNR26_MOUSE	Tumor necrosis factor receptor superfamily member 26 (TNF receptor homolog 3)
P86174	BEND4_MOUSE	BEN domain-containing protein 4
P86448	TR43B_MOUSE	Tripartite motif-containing protein 43B
P97300	NPTN_MOUSE	Neuroplastin (Stromal cell-derived receptor 1) (SDR-1)
P97326	CADH6_MOUSE	Cadherin-6 (Kidney cadherin) (K-cadherin)
P97434	MPRIP_MOUSE	Myosin phosphatase Rho-interacting protein (Rho-interacting protein 3) (RIP3) (p116Rip)
P97452	BOP1_MOUSE	Ribosome biogenesis protein BOP1 (Block of proliferation 1 protein)
P97468	CML1_MOUSE	Chemokine-like receptor 1 (G-protein coupled receptor DEZ)
P97825	JUPI1_MOUSE	Jupiter microtubule associated homolog 1 (Hematological and neurological expressed 1 protein) [Cleaved into: Jupiter microtubule associated homolog 1, N-terminally processed]
P97930	KTHY_MOUSE	Thymidylate kinase (EC 2.7.4.9) (dTMP kinase)
P98083	SHC1_MOUSE	SHC-transforming protein 1 (SHC-transforming protein A) (Src homology 2 domain-containing-transforming protein C1) (SH2 domain protein C1)
P98154	IDD_MOUSE	Integral membrane protein DGCR2/IDD (Seizure-related membrane-bound adhesion protein)
Q00262	STX2_MOUSE	Syntaxin-2 (Epimorphin)
Q00417	TCF7_MOUSE	Transcription factor 7 (TCF-7) (T-cell-specific transcription factor 1) (T-cell factor 1) (TCF-1)
Q00420	GABP1_MOUSE	GA-binding protein subunit beta-1 (GABP subunit beta-1) (GABPB-1) (GABP subunit beta-2) (GABPB-2)
Q00898	A1AT5_MOUSE	Alpha-1-antitrypsin 1-5 (Alpha-1 protease inhibitor 5) (Serine protease inhibitor 1-5) (Serine protease inhibitor A1e) (Serpin A1e)
Q03172	ZEP1_MOUSE	Zinc finger protein 40 (Alpha A-crystallin-binding protein 1) (Alpha A-CRYBP1) (Alpha A-crystallin-binding protein I) (Transcription factor alphaA-CRYBP1)
Q04692	SMRCD_MOUSE	SWI/SNF-related matrix-associated actin-dependent regulator of chromatin subfamily A containing DEAD/H box 1 (EC 3.6.4.12) (ATP-dependent helicase SMARCAD1) (Enhancer trap locus homolog 1) (Etl-1)
Q05AA6	DRP2_MOUSE	Dystrophin-related protein 2 (DRP-2)
Q07643	CO9A2_MOUSE	Collagen alpha-2(IX) chain
Q07802	COE1_MOUSE	Transcription factor COE1 (O/E-1) (OE-1) (Early B-cell factor)

Q08639	TFDP1_MOUSE	Transcription factor Dp-1 (DRTF1-polypeptide 1) (E2F dimerization partner 1)
Q0GNC1	INF2_MOUSE	Inverted formin-2
Q0KK55	KNDC1_MOUSE	Kinase non-catalytic C-lobe domain-containing protein 1 (KIND domain-containing protein 1) (Protein very KIND) (v-KIND) (Ras-GEF domain-containing family member 2)
Q0V8T8	CTP5B_MOUSE	Contactin-associated protein like 5-2 (Cell recognition molecule Caspr5-2) (Cell recognition molecule Caspr5b) (Contactin-associated protein-like 5b)
Q149B8	PERM1_MOUSE	PGC-1 and ERR-induced regulator in muscle protein 1 (PPARGC1 and ESRR-induced regulator in muscle 1) (Peroxisome proliferator-activated receptor gamma coactivator 1 and estrogen-related receptor-induced regulator in muscle 1)
Q149C3	LIGO4_MOUSE	Leucine-rich repeat and immunoglobulin-like domain containing-NOGO receptor-interacting protein 4 (Leucine-rich repeat neuronal protein 6D)
Q14B71	CDCA2_MOUSE	Cell division cycle-associated protein 2
Q14BI2	GRM2_MOUSE	Metabotropic glutamate receptor 2 (mGluR2)
Q14BP6	LR74B_MOUSE	Leucine-rich repeat-containing protein 74B
Q19LI2	A1BG_MOUSE	Alpha-1B-glycoprotein (Alpha-1-B glycoprotein)
Q1XH17	TRI72_MOUSE	Tripartite motif-containing protein 72 (Mitsugumin-53) (Mg53)
Q2TPA8	HSDL2_MOUSE	Hydroxysteroid dehydrogenase-like protein 2 (EC 1.-.-.-)
Q2VPD4	BMAL2_MOUSE	Aryl hydrocarbon receptor nuclear translocator-like protein 2 (Brain and muscle ARNT-like 2)
Q32P12	CQ053_MOUSE	Uncharacterized protein C17orf53 homolog
Q3SYK4	VRTN_MOUSE	Vertnin
Q3TCH7	CUL4A_MOUSE	Cullin-4A (CUL-4A)
Q3TD16	PACER_MOUSE	Protein associated with UVRAG as autophagy enhancer (Pacer) (Protein Rubicon-like)
Q3TRM4	PLPL6_MOUSE	Neuropathy target esterase (EC 3.1.1.5) (Patatin-like phospholipase domain-containing protein 6)
Q3TWI9	CSCL2_MOUSE	CSC1-like protein 2 (Transmembrane protein 63B)
Q3TXU5	DHYS_MOUSE	Deoxyhypusine synthase (DHS) (EC 2.5.1.46)
Q3U0V2	TRADD_MOUSE	Tumor necrosis factor receptor type 1-associated DEATH domain protein (TNFR1-associated DEATH domain protein) (TNFRSF1A-associated via death domain)
Q3U129	B3GNL_MOUSE	UDP-GlcNAc:betaGal beta-1,3-N-acetylglucosaminyltransferase-like protein 1 (BGnT-like protein 1) (Beta1,3-N-

		acetylglucosaminyltransferase-like protein 1) (Beta3Gn-T-like protein 1) (Beta3GnTL1) (EC 2.4.1.-)
Q3U1V6	UEVLD_MOUSE	Ubiquitin-conjugating enzyme E2 variant 3 (UEV-3) (EV and lactate/malate dehydrogenase domain-containing protein)
Q3U319	BRE1B_MOUSE	E3 ubiquitin-protein ligase BRE1B (BRE1-B) (EC 2.3.2.27) (RING finger protein 40) (RING-type E3 ubiquitin transferase BRE1B)
Q3U410	KLH21_MOUSE	Kelch-like protein 21
Q3U435	MMP25_MOUSE	Matrix metalloproteinase-25 (MMP-25) (EC 3.4.24.-)
Q3U4B4	LIPN_MOUSE	Lipase member N (EC 3.1.1.-) (Lipase-like abhydrolase domain-containing protein 4)
Q3U6B2	GP183_MOUSE	G-protein coupled receptor 183 (Epstein-Barr virus-induced G-protein coupled receptor 2 homolog) (EBI2) (EBV-induced G-protein coupled receptor 2 homolog)
Q3U827	RN180_MOUSE	E3 ubiquitin-protein ligase RNF180 (EC 2.3.2.27) (RING finger protein 180) (RING-type E3 ubiquitin transferase RNF180)
Q3U829	AP5Z1_MOUSE	AP-5 complex subunit zeta-1 (Adaptor-related protein complex 5 zeta subunit) (Zeta5)
Q3UBG2	PCLI1_MOUSE	PTB-containing, cubilin and LRP1-interacting protein (P-CLI1) (Phosphotyrosine interaction domain-containing protein 1)
Q3UEI1	PDE4C_MOUSE	cAMP-specific 3',5'-cyclic phosphodiesterase 4C (EC 3.1.4.53)
Q3UFK8	FRMD8_MOUSE	FERM domain-containing protein 8 (iRhom tail-associated protein) (iTAP)
Q3UFT3	GARE1_MOUSE	GRB2-associated and regulator of MAPK protein (GRB2-associated and regulator of MAPK1)
Q3UH53	SDK1_MOUSE	Protein sidekick-1
Q3UHH2	S22AN_MOUSE	Solute carrier family 22 member 23
Q3UHN9	NDST1_MOUSE	Bifunctional heparan sulfate N-deacetylase/N-sulfotransferase 1 (EC 2.8.2.8) (Glucosaminyl N-deacetylase/N-sulfotransferase 1) (NDST-1) (N-heparan sulfate sulfotransferase 1) (N-HSST 1) ([Heparan sulfate]-glucosamine N-sulfotransferase 1) (HSNST 1) [Includes: Heparan sulfate N-deacetylase 1 (EC 3.-.-.-); Heparan sulfate N-sulfotransferase 1 (EC 2.8.2.-)]
Q3UHV4	TR5OS_MOUSE	Putative uncharacterized protein TRPC5OS homolog
Q3UIW5	RNF10_MOUSE	RING finger protein 10 (Sid 2705)

Q3UJ81	NEPR1_MOUSE	Nuclear envelope phosphatase-regulatory subunit 1 (NEP1-R1) (Transmembrane protein 188)
Q3UMF0	COBL1_MOUSE	Cordon-bleu protein-like 1 (Cobl-related protein 1)
Q3UND0	SKAP2_MOUSE	Src kinase-associated phosphoprotein 2 (Pyk2/RAFTK-associated protein) (SKAP55 homolog) (SKAP-HOM) (Src family-associated phosphoprotein 2) (Src kinase-associated phosphoprotein 55-related protein) (Src-associated adapter protein with PH and SH3 domains)
Q3UNH4	GRIN1_MOUSE	G protein-regulated inducer of neurite outgrowth 1 (GRIN1)
Q3US16	TEANC_MOUSE	Transcription elongation factor A N-terminal and central domain-containing protein (TFIIS central domain-containing protein 1)
Q3UU35	OVOS_MOUSE	Ovostatin homolog
Q3UUX7	CD046_MOUSE	Uncharacterized protein C4orf46 homolog
Q3UV17	K22O_MOUSE	Keratin, type II cytoskeletal 2 oral (Keratin-76) (K76) (Type-II keratin Kb9)
Q3UV55	NR1D1_MOUSE	Nuclear receptor subfamily 1 group D member 1 (Rev-erbA-alpha) (V-erbA-related protein 1) (EAR-1)
Q3UVK0	ERMP1_MOUSE	Endoplasmic reticulum metallopeptidase 1 (EC 3.4.-.-) (Felix-ina)
Q3UX62	CC114_MOUSE	Coiled-coil domain-containing protein 114
Q3UYK3	TBCD9_MOUSE	TBC1 domain family member 9
Q3UZ39	LRRF1_MOUSE	Leucine-rich repeat flightless-interacting protein 1 (LRR FLII-interacting protein 1) (FLI-LRR-associated protein 1) (Flap-1) (H186 FLAP)
Q3UZY0	SFI1_MOUSE	Protein SFI1 homolog
Q3V3Q7	PACS2_MOUSE	Phosphofurin acidic cluster sorting protein 2 (PACS-2) (PACS1-like protein)
Q3V3R4	ITA1_MOUSE	Integrin alpha-1 (CD49 antigen-like family member A) (Laminin and collagen receptor) (VLA-1) (CD antigen CD49a)
Q45VK7	DYHC2_MOUSE	Cytoplasmic dynein 2 heavy chain 1 (Cytoplasmic dynein 2 heavy chain) (Dynein cytoplasmic heavy chain 2) (Dynein heavy chain 11) (mDHC11) (Dynein heavy chain isotype 1B)
Q497N7	CF201_MOUSE	Uncharacterized protein C6orf201 homolog
Q499M4	TIGD5_MOUSE	Tigger transposable element derived 5
Q4KL25	LHPL5_MOUSE	LHFPL tetraspan subfamily member 5 protein (Lipoma HMGIC fusion partner-like 5 protein) (Tetraspan membrane protein of hair cell stereocilia)

Q4VA61	DSCL1_MOUSE	Down syndrome cell adhesion molecule-like protein 1 homolog
Q4VGL6	RC3H1_MOUSE	Roquin-1 (Roquin) (EC 2.3.2.27) (Protein Sanroque) (RING finger and C3H zinc finger protein 1) (RING finger and CCCH-type zinc finger domain-containing protein 1)
Q50H33	KCTD8_MOUSE	BTB/POZ domain-containing protein KCTD8
Q571C7	BDP1_MOUSE	Transcription factor TFIIIB component B" homolog (Transcription factor IIIB 150) (TFIIIB150) (Transcription factor-like nuclear regulator)
Q571G4	LIN54_MOUSE	Protein lin-54 homolog
Q5BJ29	FBXL7_MOUSE	F-box/LRR-repeat protein 7 (F-box and leucine-rich repeat protein 7)
Q5DTJ9	MYPN_MOUSE	Myopalladin
Q5DTT2	PSD1_MOUSE	PH and SEC7 domain-containing protein 1 (Exchange factor for ADP-ribosylation factor guanine nucleotide factor 6) (Exchange factor for ARF6) (Exchange factor for ARF6 A) (Pleckstrin homology and SEC7 domain-containing protein 1)
Q5DTV4	ZC12C_MOUSE	Probable ribonuclease ZC3H12C (EC 3.1.-.-) (Zinc finger CCCH domain-containing protein 12C)
Q5DU25	IQEC2_MOUSE	IQ motif and SEC7 domain-containing protein 2
Q5DU28	PCX2_MOUSE	Pecanex-like protein 2 (Pecanex homolog protein 2)
Q5EBG6	HSPB6_MOUSE	Heat shock protein beta-6 (HspB6)
Q5FWH6	ARHGF_MOUSE	Rho guanine nucleotide exchange factor 15 (Ephexin-5) (E5)
Q5GAM9	RNS11_MOUSE	Putative inactive ribonuclease 11 (RNase 11)
Q5GH62	XKR9_MOUSE	XK-related protein 9
Q5HZH2	TSR3_MOUSE	Ribosome biogenesis protein TSR3 homolog
Q5HZI9	S2551_MOUSE	Solute carrier family 25 member 51 (Mitochondrial carrier triple repeat protein 1)
Q5IRJ6	ZNT9_MOUSE	Zinc transporter 9 (ZnT-9) (GRIP1-associated coactivator 63) (GAC63) (Solute carrier family 30 member 9)
Q5NCY0	KDM6B_MOUSE	Lysine-specific demethylase 6B (EC 1.14.11.-) (JmjC domain-containing protein 3) (Jumonji domain-containing protein 3)
Q5PRE5	PRSR1_MOUSE	Proline and serine-rich protein 1
Q5SPW0	VPS54_MOUSE	Vacuolar protein sorting-associated protein 54 (Tumor antigen SLP-8p homolog)
Q5SPX1	CC157_MOUSE	Coiled-coil domain-containing protein 157
Q5SRT8	CCNJL_MOUSE	Cyclin-J-like protein
Q5SUV2	MYBPP_MOUSE	MYCBP-associated protein (AMAM-1) (AMY-1-binding protein 1) (AMAP-1)

Q5SVQ0	KAT7_MOUSE	Histone acetyltransferase KAT7 (EC 2.3.1.48) (Histone acetyltransferase binding to ORC1) (Lysine acetyltransferase 7) (MOZ, YBF2/SAS3, SAS2 and TIP60 protein 2) (MYST-2)
Q5SVT3	ETAA1_MOUSE	Ewing's tumor-associated antigen 1 homolog
Q5SWW4	MED13_MOUSE	Mediator of RNA polymerase II transcription subunit 13 (Thyroid hormone receptor-associated protein 1) (Thyroid hormone receptor-associated protein complex 240 kDa component) (Trap240)
Q5SX39	MYH4_MOUSE	Myosin-4 (Myosin heavy chain 2b) (MyHC-2b) (Myosin heavy chain 4)
Q5XF90	AT134_MOUSE	Probable cation-transporting ATPase 13A4 (EC 7.2.2.-) (P5-ATPase isoform 4)
Q60603	KCNH1_MOUSE	Potassium voltage-gated channel subfamily H member 1 (Ether-a-go-go potassium channel 1) (EAG channel 1) (EAG1) (m-eag) (Voltage-gated potassium channel subunit Kv10.1)
Q60641	NR1H4_MOUSE	Bile acid receptor (Farnesoid X-activated receptor) (Farnesol receptor HRR-1) (Nuclear receptor subfamily 1 group H member 4) (Retinoid X receptor-interacting protein 14) (RXR-interacting protein 14)
Q60707	TBX2_MOUSE	T-box transcription factor TBX2 (T-box protein 2)
Q60715	P4HA1_MOUSE	Prolyl 4-hydroxylase subunit alpha-1 (4-PH alpha-1) (EC 1.14.11.2) (Procollagen-proline,2-oxoglutarate-4-dioxygenase subunit alpha-1)
Q60722	ITF2_MOUSE	Transcription factor 4 (TCF-4) (Class A helix-loop-helix transcription factor ME2) (Immunoglobulin transcription factor 2) (ITF-2) (MITF-2) (SL3-3 enhancer factor 2) (SEF-2)
Q60736	ZP3R_MOUSE	Zona pellucida sperm-binding protein 3 receptor (Sperm fertilization protein 56) (sp56)
Q60760	GRB10_MOUSE	Growth factor receptor-bound protein 10 (GRB10 adapter protein) (Maternally expressed gene 1 protein)
Q60790	RASA3_MOUSE	Ras GTPase-activating protein 3 (GAP1(IP4BP)) (GapIII) (Ins P4-binding protein)
Q60793	KLF4_MOUSE	Krueppel-like factor 4 (Epithelial zinc finger protein EZF) (Gut-enriched krueppel-like factor)
Q60855	RIPK1_MOUSE	Receptor-interacting serine/threonine-protein kinase 1 (EC 2.7.11.1) (Cell death protein RIP) (Receptor-interacting protein 1) (RIP-1)
Q60886	OL147_MOUSE	Olfactory receptor 147 (Odorant receptor M3) (Olfactory receptor 164-1) (Olfactory receptor 7E)

Q61083	M3K2_MOUSE	Mitogen-activated protein kinase kinase kinase 2 (EC 2.7.11.25) (MAPK/ERK kinase kinase 2) (MEK kinase 2) (MEKK 2)
Q61084	M3K3_MOUSE	Mitogen-activated protein kinase kinase kinase 3 (EC 2.7.11.25) (MAPK/ERK kinase kinase 3) (MEK kinase 3) (MEKK 3)
Q61091	FZD8_MOUSE	Frizzled-8 (Fz-8) (mFz8)
Q61102	ABCB7_MOUSE	ATP-binding cassette sub-family B member 7, mitochondrial (ATP-binding cassette transporter 7) (ABC transporter 7 protein)
Q61121	GPR19_MOUSE	Probable G-protein coupled receptor 19
Q61211	EIF2D_MOUSE	Eukaryotic translation initiation factor 2D (eIF2D) (Ligatin)
Q61398	PCOC1_MOUSE	Procollagen C-endopeptidase enhancer 1 (P14) (Procollagen COOH-terminal proteinase enhancer 1) (PCPE-1) (Procollagen C-proteinase enhancer 1) (Type 1 procollagen C-proteinase enhancer protein) (Type I procollagen COOH-terminal proteinase enhancer)
Q61418	CLCN4_MOUSE	H(+)/Cl(-) exchange transporter 4 (Chloride channel protein 4) (ClC-4) (Chloride transporter ClC-4)
Q61501	E2F1_MOUSE	Transcription factor E2F1 (E2F-1)
Q61503	5NTD_MOUSE	5'-nucleotidase (5'-NT) (EC 3.1.3.5) (Ecto-5'-nucleotidase) (CD antigen CD73)
Q61527	ERBB4_MOUSE	Receptor tyrosine-protein kinase erbB-4 (EC 2.7.10.1) (Proto-oncogene-like protein c-ErbB-4) [Cleaved into: ERBB4 intracellular domain (4ICD) (E4ICD) (s80HER4)]
Q61543	GSLG1_MOUSE	Golgi apparatus protein 1 (E-selectin ligand 1) (ESL-1) (Selel) (Golgi sialoglycoprotein MG-160)
Q61609	S20A1_MOUSE	Sodium-dependent phosphate transporter 1 (Gibbon ape leukemia virus receptor 1) (GLVR-1) (Leukemia virus receptor 1 homolog) (Phosphate transporter 1) (PiT-1) (Solute carrier family 20 member 1)
Q61730	IL1AP_MOUSE	Interleukin-1 receptor accessory protein (IL-1 receptor accessory protein) (IL-1RAcP) (Interleukin-33 receptot beta chain)
Q61738	ITA7_MOUSE	Integrin alpha-7 [Cleaved into: Integrin alpha-7 heavy chain; Integrin alpha-7 light chain]
Q61765	K1H1_MOUSE	Keratin, type I cuticular Ha1 (HKA-1) (Hair keratin, type I Ha1) (Keratin-31) (K31)
Q61817	CREB3_MOUSE	Cyclic AMP-responsive element-binding protein 3 (CREB-3) (cAMP-responsive element-binding protein 3) (Transcription factor LZIP) [Cleaved

		into: Processed cyclic AMP-responsive element-binding protein 3]
Q61823	PDCD4_MOUSE	Programmed cell death protein 4 (Protein MA-3) (Topoisomerase-inhibitor suppressed protein)
Q61847	MEP1B_MOUSE	Meprin A subunit beta (EC 3.4.24.63) (Endopeptidase-2) (Meprin B)
Q61884	MNS1_MOUSE	Meiosis-specific nuclear structural protein 1
Q62036	CP131_MOUSE	Centrosomal protein of 131 kDa (5-azacytidine-induced protein 1) (Pre-acrosome localization protein 1)
Q62132	PTPRR_MOUSE	Receptor-type tyrosine-protein phosphatase R (R-PTP-R) (EC 3.1.3.48) (Phosphotyrosine phosphatase 13) (Protein-tyrosine-phosphatase SL)
Q62177	SEM3B_MOUSE	Semaphorin-3B (Semaphorin-A) (Sema A)
Q62187	TTF1_MOUSE	Transcription termination factor 1 (TTF-1) (RNA polymerase I termination factor) (Transcription termination factor I) (TTF-I) (mTFF-I)
Q62230	SN_MOUSE	Sialoadhesin (Sheep erythrocyte receptor) (SER) (Sialic acid-binding Ig-like lectin 1) (Siglec-1) (CD antigen CD169)
Q62315	JARD2_MOUSE	Protein Jumonji (Jumonji/ARID domain-containing protein 2)
Q62384	ZPR1_MOUSE	Zinc finger protein ZPR1 (Zinc finger protein 259)
Q62419	SH3G1_MOUSE	Endophilin-A2 (Endophilin-2) (SH3 domain protein 2B) (SH3 domain-containing GRB2-like protein 1) (SH3p8)
Q63829	COMD3_MOUSE	COMM domain-containing protein 3 (Bmi-1 upstream gene protein) (Bup protein)
Q64012	RALY_MOUSE	RNA-binding protein Raly (Maternally-expressed hnRNP C-related protein) (hnRNP associated with lethal yellow protein)
Q640M6	GDPD5_MOUSE	Glycerophosphodiester phosphodiesterase domain-containing protein 5 (EC 3.1.4.11) (Glycerophosphodiester phosphodiesterase 2)
Q64104	NR2E1_MOUSE	Nuclear receptor subfamily 2 group E member 1 (Nuclear receptor TLX) (Protein tailless homolog) (Tll) (mTll)
Q64288	OMP_MOUSE	Olfactory marker protein
Q64331	MYO6_MOUSE	Unconventional myosin-VI (Unconventional myosin-6)
Q64347	CLCN1_MOUSE	Chloride channel protein 1 (ClC-1) (Chloride channel protein, skeletal muscle)
Q64441	CP24A_MOUSE	1,25-dihydroxyvitamin D(3) 24-hydroxylase, mitochondrial (24-OHase) (Vitamin D(3) 24-

		hydroxylase) (EC 1.14.15.16) (Cytochrome P450 24A1) (Cytochrome P450-CC24)
Q64442	DHSO_MOUSE	Sorbitol dehydrogenase (SDH) (SORD) (EC 1.1.1.-) (L-iditol 2-dehydrogenase) (EC 1.1.1.14) (Polyol dehydrogenase) (Xylitol dehydrogenase) (XDH) (EC 1.1.1.9)
Q64519	SDC3_MOUSE	Syndecan-3 (SYND3)
Q64701	RBL1_MOUSE	Retinoblastoma-like protein 1 (107 kDa retinoblastoma-associated protein) (p107) (pRb1)
Q64704	STX3_MOUSE	Syntaxin-3
Q66JY6	ARG39_MOUSE	Rho guanine nucleotide exchange factor 39
Q66T02	PKHG5_MOUSE	Pleckstrin homology domain-containing family G member 5 (PH domain-containing family G member 5) (Synectin-binding RhoA exchange factor) (SYX)
Q66X19	NAL4E_MOUSE	NACHT, LRR and PYD domains-containing protein 4E (NALP-epsilon)
Q68ED3	PAPD5_MOUSE	Terminal nucleotidyltransferase 4B (Non-canonical poly(A) RNA polymerase PAPD5) (EC 2.7.7.19) (PAP-associated domain-containing protein 5) (Terminal guanylyltransferase) (EC 2.7.7.-) (Terminal uridylyltransferase 3) (TUTase 3) (Topoisomerase-related function protein 4-2) (TRF4-2)
Q68EF0	RAB3I_MOUSE	Rab-3A-interacting protein (Rab3A-interacting protein) (Rabin-3) (SSX2-interacting protein)
Q68FE9	TSH2_MOUSE	Teashirt homolog 2 (SDCCAG33-like protein) (Zinc finger protein 218)
Q68FF6	GIT1_MOUSE	ARF GTPase-activating protein GIT1 (ARF GAP GIT1) (G protein-coupled receptor kinase-interactor 1) (GRK-interacting protein 1)
Q68FF7	SLAI1_MOUSE	SLAIN motif-containing protein 1
Q69ZR9	TASOR_MOUSE	Protein TASOR (Transgene activation suppressor protein)
Q69ZS7	HBS1L_MOUSE	HBS1-like protein
Q6DFV3	RHG21_MOUSE	Rho GTPase-activating protein 21 (Rho GTPase-activating protein 10) (Rho-type GTPase-activating protein 21)
Q6H1V1	BEST3_MOUSE	Bestrophin-3 (Vitelliform macular dystrophy 2-like protein 3)
Q6HA09	ASTL_MOUSE	Astacin-like metalloendopeptidase (EC 3.4.-.-) (Oocyte astacin) (Ovastacin) (Sperm acrosomal SLLP1-binding protein)
Q6IMB1	RSLBA_MOUSE	Ras-like protein family member 11A
Q6IMH0	TEPP_MOUSE	Testis, prostate and placenta-expressed protein

Q6IR34	GPSM1_MOUSE	G-protein-signaling modulator 1 (Activator of G-protein signaling 3)
Q6IRU7	CEP78_MOUSE	Centrosomal protein of 78 kDa (Cep78)
Q6JPI3	MD13L_MOUSE	Mediator of RNA polymerase II transcription subunit 13-like (Mediator complex subunit 13-like) (Thyroid hormone receptor-associated protein 2) (Thyroid hormone receptor-associated protein complex 240 kDa component-like)
Q6KAR6	EXOC3_MOUSE	Exocyst complex component 3 (Exocyst complex component Sec6)
Q6KAU8	A16L2_MOUSE	Autophagy-related protein 16-2 (APG16-like 2)
Q6NS46	RRP5_MOUSE	Protein RRP5 homolog (Apoptosis-linked gene 4 protein) (Programmed cell death protein 11)
Q6NVD9	BFSP2_MOUSE	Phakinin (49 kDa cytoskeletal protein) (Beaded filament structural protein 2) (Lens fiber cell beaded filament protein CP 47) (CP47) (Lens fiber cell beaded filament protein CP 49) (CP49) (Lens intermediate filament-like light) (LIFL-L)
Q6NVG5	MREG_MOUSE	Melanoregulin (Dilute suppressor protein) (Whn-dependent transcript 2)
Q6NWW9	FND3B_MOUSE	Fibronectin type III domain-containing protein 3B (Factor for adipocyte differentiation 104) (HCV NS5A-binding protein 37)
Q6NXH8	MET25_MOUSE	Methyltransferase-like protein 25 (EC 2.1.1.-)
Q6P2B1	TNPO3_MOUSE	Transportin-3
Q6P8J7	KCRS_MOUSE	Creatine kinase S-type, mitochondrial (EC 2.7.3.2) (Basic-type mitochondrial creatine kinase) (Mib-CK) (Sarcomeric mitochondrial creatine kinase) (S-MtCK)
Q6P902	TXND2_MOUSE	Thioredoxin domain-containing protein 2 (Spermatid-specific thioredoxin-1) (Sptrx-1) (Thioredoxin-4)
Q6P9S0	MTSS2_MOUSE	Protein MTSS 2 (MTSS1-like protein)
Q6PAL8	DEN5A_MOUSE	DENN domain-containing protein 5A (Rab6-interacting protein 1) (Rab6IP1)
Q6PCM2	INT6_MOUSE	Integrator complex subunit 6 (Int6) (DBI-1) (Protein DDX26)
Q6PDX6	RN220_MOUSE	E3 ubiquitin-protein ligase Rnf220 (EC 2.3.2.27) (RING finger protein 220) (RING-type E3 ubiquitin transferase Rnf220)
Q6PDY2	AEDO_MOUSE	2-aminoethanethiol dioxygenase (EC 1.13.11.19) (Cysteamine dioxygenase)
Q6PE01	SNR40_MOUSE	U5 small nuclear ribonucleoprotein 40 kDa protein (U5 snRNP 40 kDa protein) (WD repeat-containing protein 57)

Q6PE84	STML3_MOUSE	Stomatin-like protein 3 (SLP-3) (Stomatin-related olfactory protein)
Q6PF93	PK3C3_MOUSE	Phosphatidylinositol 3-kinase catalytic subunit type 3 (PI3-kinase type 3) (PI3K type 3) (PtdIns-3-kinase type 3) (EC 2.7.1.137) (Phosphoinositide-3-kinase class 3)
Q6PFX2	BEND6_MOUSE	BEN domain-containing protein 6
Q6PFY8	TRI45_MOUSE	Tripartite motif-containing protein 45
Q6PGB8	SMCA1_MOUSE	Probable global transcription activator SNF2L1 (EC 3.6.4.-) (ATP-dependent helicase SMARCA1) (DNA-dependent ATPase SNF2L) (Nucleosome-remodeling factor subunit SNF2L) (SWI/SNF-related matrix-associated actin-dependent regulator of chromatin subfamily A member 1)
Q6PGL7	WASC2_MOUSE	WASH complex subunit 2
Q6PHZ5	RB15B_MOUSE	Putative RNA-binding protein 15B (RNA-binding motif protein 15B)
Q6QNU9	TLR12_MOUSE	Toll-like receptor 12 (Toll-like receptor 11)
Q6RT24	CENPE_MOUSE	Centromere-associated protein E (Centromere protein E) (CENP-E) (Kinesin superfamily protein 10) (KIF10) (Motor domain of KIF10)
Q6VZZ7	OPN5_MOUSE	Opsin-5 (G-protein coupled receptor 136) (G-protein coupled receptor PGR12) (Neuropsin)
Q6XBJ3	ONEC2_MOUSE	One cut domain family member 2 (One cut homeobox 2) (Transcription factor ONECUT-2) (OC-2)
Q6YCH2	TDPZ4_MOUSE	TD and POZ domain-containing protein 4
Q6ZPF3	TIAM2_MOUSE	T-lymphoma invasion and metastasis-inducing protein 2 (TIAM-2) (SIF and TIAM1-like exchange factor)
Q6ZPK0	PF21A_MOUSE	PHD finger protein 21A (BHC80a) (BRAF35-HDAC complex protein BHC80) (mBHC80)
Q6ZPR6	IBTK_MOUSE	Inhibitor of Bruton tyrosine kinase (IBtk)
Q6ZPY2	SMG5_MOUSE	Protein SMG5 (EST1-like protein B) (SMG-5 homolog)
Q6ZPY7	KDM3B_MOUSE	Lysine-specific demethylase 3B (EC 1.14.11.-) (JmjC domain-containing histone demethylation protein 2B) (Jumonji domain-containing protein 1B)
Q6ZQ82	RHG26_MOUSE	Rho GTPase-activating protein 26 (Rho-type GTPase-activating protein 26)
Q6ZQI3	MLEC_MOUSE	Malectin
Q6ZQM8	UD17C_MOUSE	UDP-glucuronosyltransferase 1-7C (UDPGT 1-7C) (UGT1*7C) (UGT1-07C) (UGT1.7C) (EC

		2.4.1.17) (UDP-glucuronosyltransferase 1A7C) (UGT1A10)
Q6ZWZ2	UB2R2_MOUSE	Ubiquitin-conjugating enzyme E2 R2 (EC 2.3.2.23) (E2 ubiquitin-conjugating enzyme R2) (Ubiquitin carrier protein R2) (Ubiquitin-conjugating enzyme E2-CDC34B) (Ubiquitin-protein ligase R2)
Q70FJ1	AKAP9_MOUSE	A-kinase anchor protein 9 (AKAP-9) (Protein kinase A-anchoring protein 9) (PRKA9)
Q75N73	S39AE_MOUSE	Zinc transporter ZIP14 (Factor for adipocyte differentiation 123) (FAD-123) (Solute carrier family 39 member 14) (Zrt- and Irt-like protein 14) (ZIP-14)
Q762D5	S35D2_MOUSE	UDP-N-acetylglucosamine/UDP-glucose/GDP-mannose transporter (Solute carrier family 35 member D2) (UDP-galactose transporter-related protein 8) (UGTrel8)
Q76LS9	MINY1_MOUSE	Ubiquitin carboxyl-terminal hydrolase MINDY-1 (EC 3.4.19.12) (Deubiquitinating enzyme MINDY-1) (NF-E2 inducible protein) (Protein FAM63A)
Q78IS1	TMED3_MOUSE	Transmembrane emp24 domain-containing protein 3 (p24 family protein gamma-4) (p24gamma4)
Q791V5	MTCH2_MOUSE	Mitochondrial carrier homolog 2
Q7M6U3	TEX14_MOUSE	Inactive serine/threonine-protein kinase TEX14 (Testis-expressed sequence 14) (Testis-expressed sequence 14 protein)
Q7M715	TR117_MOUSE	Taste receptor type 2 member 117 (T2R117) (mT2R54)
Q7M750	OPALI_MOUSE	Opalin (Oligodendrocytic myelin paranodal and inner loop protein) (Transmembrane protein 10)
Q7M761	OVCH2_MOUSE	Ovochymase-2 (EC 3.4.21.-) (Oviductin)
Q7TMI3	UHRF2_MOUSE	E3 ubiquitin-protein ligase UHRF2 (EC 2.3.2.27) (NIRF) (Np95-like ring finger protein) (Nuclear protein 97) (Nuclear zinc finger protein Np97) (RING-type E3 ubiquitin transferase UHRF2) (Ubiquitin-like PHD and RING finger domain-containing protein 2) (Ubiquitin-like-containing PHD and RING finger domains protein 2)
Q7TMM8	PAR16_MOUSE	Protein mono-ADP-ribosyltransferase PARP16 (EC 2.4.2.-) (ADP-ribosyltransferase diphtheria toxin-like 15) (Poly [ADP-ribose] polymerase 16) (PARP-16)
Q7TMY4	THOC7_MOUSE	THO complex subunit 7 homolog (Ngg1-interacting factor 3-like protein 1-binding protein 1)
Q7TN73	CASD1_MOUSE	N-acetylneuraminate 9-O-acetyltransferase (EC 2.3.1.45) (CAS1 domain-containing protein 1) (Sialate O-acetyltransferase) (SOAT)

Q7TNV0	DEK_MOUSE	Protein DEK
Q7TPD3	ROBO2_MOUSE	Roundabout homolog 2
Q7TPN9	PRR14_MOUSE	Proline-rich protein 14
Q7TPX9	LEGLB_MOUSE	Galectin-related protein B (Lectin galactoside-binding-like protein B)
Q7TR96	O1013_MOUSE	Olfactory receptor 1013 (Olfactory receptor 213-2)
Q7TS99	HELT_MOUSE	Hairy and enhancer of split-related protein HELT (HES/HEY-like transcription factor) (Protein Hes-like) (Protein megane)
Q7TSF0	DSG1C_MOUSE	Desmoglein-1-gamma (Dsg1-gamma) (Desmoglein-6)
Q7TSF4	LR75A_MOUSE	Leucine-rich repeat-containing protein 75A (Leucine-rich repeat-containing protein FAM211A)
Q7TSJ6	LATS2_MOUSE	Serine/threonine-protein kinase LATS2 (EC 2.7.11.1) (Kinase phosphorylated during mitosis protein) (Large tumor suppressor homolog 2) (Serine/threonine-protein kinase kpm)
Q7TSZ8	NACC1_MOUSE	Nucleus accumbens-associated protein 1 (NAC-1) (BTB/POZ domain-containing protein 14B)
Q7TT79	MCPH1_MOUSE	Microcephalin
Q80SY3	VA0D2_MOUSE	V-type proton ATPase subunit d 2 (V-ATPase subunit d 2) (Osteoclast-specific vacuolar ATP synthase) (Vacuolar proton pump subunit d 2)
Q80T41	GABR2_MOUSE	Gamma-aminobutyric acid type B receptor subunit 2 (GABA-B receptor 2) (GABA-B-R2) (GABA-BR2) (GABABR2) (Gb2) (G-protein coupled receptor 51)
Q80T79	CSMD3_MOUSE	CUB and sushi domain-containing protein 3 (CUB and sushi multiple domains protein 3)
Q80TI1	PKHH1_MOUSE	Pleckstrin homology domain-containing family H member 1 (PH domain-containing family H member 1)
Q80TN7	NAV3_MOUSE	Neuron navigator 3 (Pore membrane and/or filament-interacting-like protein 1)
Q80TR1	AGRL1_MOUSE	Adhesion G protein-coupled receptor L1 (Calcium-independent alpha-latrotoxin receptor 1) (CIRL-1) (Latrophilin-1) (Lectomedin-2)
Q80TR4	SLIT1_MOUSE	Slit homolog 1 protein (Slit-1)
Q80TS3	AGRL3_MOUSE	Adhesion G protein-coupled receptor L3 (Latrophilin-3) (Lectomedin-3)
Q80TV8	CLAP1_MOUSE	CLIP-associating protein 1 (Cytoplasmic linker-associated protein 1)
Q80U22	RUSC2_MOUSE	Iporin (Interacting protein of Rab1) (RUN and SH3 domain-containing protein 2)

Q80U49	C170B_MOUSE	Centrosomal protein of 170 kDa protein B (Centrosomal protein 170B) (Cep170B)
Q80U63	MFN2_MOUSE	Mitofusin-2 (EC 3.6.5.-) (Hypertension-related protein 1) (Mitochondrial assembly regulatory factor) (HSG protein) (Transmembrane GTPase MFN2)
Q80V03	ADCK5_MOUSE	Uncharacterized aarF domain-containing protein kinase 5 (EC 2.7.11.-)
Q80WJ7	LYRIC_MOUSE	Protein LYRIC (3D3/LYRIC) (Lysine-rich CEACAM1 co-isolated protein) (Metadherin) (Metastasis adhesion protein)
Q80XI6	M3K11_MOUSE	Mitogen-activated protein kinase kinase kinase 11 (EC 2.7.11.25) (Mixed lineage kinase 3)
Q80Y24	PRIC2_MOUSE	Prickle-like protein 2
Q80YA3	DDHD1_MOUSE	Phospholipase DDHD1 (EC 3.1.1.-) (DDHD domain-containing protein 1) (Phosphatidic acid-preferring phospholipase A1 homolog) (PA-PLA1)
Q80YF9	RHG33_MOUSE	Rho GTPase-activating protein 33 (Rho-type GTPase-activating protein 33) (Sorting nexin-26) (Tc10/CDC42 GTPase-activating protein)
Q80YR2	F16B2_MOUSE	Protein FAM160B2 (Retinoic acid-induced protein 16)
Q80YR4	ZN598_MOUSE	E3 ubiquitin-protein ligase ZNF598 (EC 2.3.2.27) (Zinc finger protein 598)
Q80YU0	ABHGB_MOUSE	Protein ABHD16B (EC 3.-.-.-) (Alpha/beta hydrolase domain-containing protein 16B) (Abhydrolase domain-containing protein 16B)
Q80Z96	VANG1_MOUSE	Vang-like protein 1 (Loop-tail protein 2) (Van Gogh-like protein 1)
Q80ZD8	AMGO1_MOUSE	Amphoterin-induced protein 1 (AMIGO-1) (Alivin-2)
Q80ZJ7	SNX32_MOUSE	Sorting nexin-32 (Sorting nexin-6B)
Q80ZQ9	ABITM_MOUSE	Protein Abitram (Actin-binding transcription modulator) (Protein Simiate)
Q80ZU7	BPIB3_MOUSE	BPI fold-containing family B member 3 (Ligand-binding protein RYA3) (Long palate, lung and nasal epithelium carcinoma-associated protein 3)
Q810D6	GRWD1_MOUSE	Glutamate-rich WD repeat-containing protein 1 (Protein A301)
Q810I2	TRI50_MOUSE	E3 ubiquitin-protein ligase TRIM50 (EC 2.3.2.27) (RING-type E3 ubiquitin transferase TRIM50) (Tripartite motif-containing protein 50)
Q810K3	SGPP2_MOUSE	Sphingosine-1-phosphate phosphatase 2 (SPPase2) (Spp2) (EC 3.1.3.-) (Sphingosine-1-phosphatase 2)
Q810N5	CL060_MOUSE	Uncharacterized protein C12orf60 homolog

Q811B1	XYLT1_MOUSE	Xylosyltransferase 1 (EC 2.4.2.26) (Peptide O-xylosyltransferase 1) (Xylosyltransferase I)
Q811D0	DLG1_MOUSE	Disks large homolog 1 (Embryo-dlg/synapse-associated protein 97) (E-dlg/SAP97) (Synapse-associated protein 97) (SAP-97) (SAP97)
Q811L6	MAST4_MOUSE	Microtubule-associated serine/threonine-protein kinase 4 (EC 2.7.11.1)
Q8BFW4	TRI65_MOUSE	Tripartite motif-containing protein 65
Q8BG22	CLCA2_MOUSE	Calcium-activated chloride channel regulator 2 (EC 3.4.-.-) (Calcium-activated chloride channel family member 5) (mCLCA5)
Q8BG40	KTNB1_MOUSE	Katanin p80 WD40 repeat-containing subunit B1 (Katanin p80 subunit B1) (p80 katanin)
Q8BG54	SPTC3_MOUSE	Serine palmitoyltransferase 3 (EC 2.3.1.50) (Long chain base biosynthesis protein 2b) (LCB2b) (Long chain base biosynthesis protein 3) (LCB 3) (Serine-palmityl-CoA transferase 3) (SPT 3)
Q8BGD6	S38A9_MOUSE	Sodium-coupled neutral amino acid transporter 9 (Solute carrier family 38 member 9)
Q8BGJ9	U2AF4_MOUSE	Splicing factor U2AF 26 kDa subunit (U2 auxiliary factor 26) (U2 small nuclear RNA auxiliary factor 1-like protein 4) (U2AF1-like 4)
Q8BGT8	PHIPL_MOUSE	Phytanoyl-CoA hydroxylase-interacting protein-like
Q8BGY4	KLH26_MOUSE	Kelch-like protein 26
Q8BH24	TM9S4_MOUSE	Transmembrane 9 superfamily member 4
Q8BH66	ATLA1_MOUSE	Atlastin-1 (EC 3.6.5.-) (Spastic paraplegia 3A homolog)
Q8BH74	NU107_MOUSE	Nuclear pore complex protein Nup107 (107 kDa nucleoporin) (Nucleoporin Nup107)
Q8BH97	RCN3_MOUSE	Reticulocalbin-3
Q8BHK3	S36A2_MOUSE	Proton-coupled amino acid transporter 2 (Proton/amino acid transporter 2) (Solute carrier family 36 member 2) (Tramdorin-1)
Q8BHW6	SPT21_MOUSE	Spermatogenesis-associated protein 21
Q8BHX3	BOREA_MOUSE	Borealin (Cell division cycle-associated protein 8) (MESrg)
Q8BI84	TGO1_MOUSE	Transport and Golgi organization protein 1 homolog (TANGO1) (Melanoma inhibitory activity protein 3)
Q8BID8	FXL14_MOUSE	F-box/LRR-repeat protein 14 (F-box and leucine-rich repeat protein 14)
Q8BJ25	THAP3_MOUSE	THAP domain-containing protein 3

Q8BJ56	PLPL2_MOUSE	Patatin-like phospholipase domain-containing protein 2 (EC 3.1.1.3) (Adipose triglyceride lipase) (Desnutrin)
Q8BK26	FBX44_MOUSE	F-box only protein 44 (F-box only protein 6a)
Q8BKI2	TNR6B_MOUSE	Trinucleotide repeat-containing gene 6B protein
Q8BKS9	PUM3_MOUSE	Pumilio homolog 3
Q8BL43	RASFA_MOUSE	Ras association domain-containing protein 10
Q8BLB7	LMBL3_MOUSE	Lethal(3)malignant brain tumor-like protein 3 (L(3)mbt-like protein 3) (MBT-1)
Q8BLD9	DRD5_MOUSE	D(1B) dopamine receptor (D(5) dopamine receptor) (Dopamine D5 receptor)
Q8BLR5	PSD4_MOUSE	PH and SEC7 domain-containing protein 4 (Exchange factor for ADP-ribosylation factor guanine nucleotide factor 6 B) (Exchange factor for ARF6 B) (Pleckstrin homology and SEC7 domain-containing protein 4)
Q8BLY2	SYTC2_MOUSE	Threonine--tRNA ligase 2, cytoplasmic (EC 6.1.1.3) (Threonyl-tRNA synthetase) (ThrRS) (Threonyl-tRNA synthetase-like protein 2)
Q8BMA6	SRP68_MOUSE	Signal recognition particle subunit SRP68 (SRP68) (Signal recognition particle 68 kDa protein)
Q8BMF4	ODP2_MOUSE	Dihydrolipoyllysine-residue acetyltransferase component of pyruvate dehydrogenase complex, mitochondrial (EC 2.3.1.12) (Dihydrolipoamide acetyltransferase component of pyruvate dehydrogenase complex) (Pyruvate dehydrogenase complex component E2) (PDC-E2) (PDCE2)
Q8BMT9	HHAT_MOUSE	Protein-cysteine N-palmitoyltransferase HHAT (EC 2.3.1.-) (Hedgehog acyltransferase) (Skinny hedgehog protein)
Q8BNJ2	ATS4_MOUSE	A disintegrin and metalloproteinase with thrombospondin motifs 4 (ADAM-TS 4) (ADAM-TS4) (ADAMTS-4) (EC 3.4.24.82) (Aggrecanase-1)
Q8BNU0	ARMC6_MOUSE	Armadillo repeat-containing protein 6
Q8BP99	CA216_MOUSE	UPF0500 protein C1orf216 homolog
Q8BPA8	DPCD_MOUSE	Protein DPCD
Q8BPC6	IDHG2_MOUSE	Probable isocitrate dehydrogenase [NAD] gamma 2, mitochondrial (Isocitric dehydrogenase subunit gamma 2) (NAD(+)-specific ICDH subunit gamma 2)
Q8BPM0	DAAM1_MOUSE	Disheveled-associated activator of morphogenesis 1
Q8BQ47	CNPY4_MOUSE	Protein canopy homolog 4 (Protein associated with Tlr4)

Q8BQM9	MD12L_MOUSE	Mediator of RNA polymerase II transcription subunit 12-like protein (Mediator complex subunit 12-like protein)
Q8BQP9	R7BP_MOUSE	Regulator of G-protein signaling 7-binding protein (R7 family-binding protein)
Q8BRK9	MA2A2_MOUSE	Alpha-mannosidase 2x (EC 3.2.1.114) (Alpha-mannosidase IIx) (Man IIx) (Mannosidase alpha class 2A member 2) (Mannosyl-oligosaccharide 1,3-1,6-alpha-mannosidase)
Q8BSS9	LIPA2_MOUSE	Liprin-alpha-2 (Protein tyrosine phosphatase receptor type f polypeptide-interacting protein alpha-2) (PTPRF-interacting protein alpha-2)
Q8BTH8	KC1G1_MOUSE	Casein kinase I isoform gamma-1 (CKI-gamma 1) (EC 2.7.11.1)
Q8BTI7	ANR52_MOUSE	Serine/threonine-protein phosphatase 6 regulatory ankyrin repeat subunit C (PP6-ARS-C) (Serine/threonine-protein phosphatase 6 regulatory subunit ARS-C)
Q8BTK5	SMYD4_MOUSE	SET and MYND domain-containing protein 4 (EC 2.1.1.-)
Q8BTV2	CPSF7_MOUSE	Cleavage and polyadenylation specificity factor subunit 7
Q8BU88	RM22_MOUSE	39S ribosomal protein L22, mitochondrial (L22mt) (MRP-L22)
Q8BVI5	STX16_MOUSE	Syntaxin-16
Q8BW74	HLF_MOUSE	Hepatic leukemia factor
Q8BWB6	STEA2_MOUSE	Metalloreductase STEAP2 (EC 1.16.1.-) (Six-transmembrane epithelial antigen of prostate 2)
Q8BWJ3	KPB2_MOUSE	Phosphorylase b kinase regulatory subunit alpha, liver isoform (Phosphorylase kinase alpha L subunit)
Q8BWS5	GRIN3_MOUSE	G protein-regulated inducer of neurite outgrowth 3 (GRIN3)
Q8BX22	SALL4_MOUSE	Sal-like protein 4 (Zinc finger protein SALL4)
Q8BXA6	CLD17_MOUSE	Claudin-17
Q8BXA7	PHLP2_MOUSE	PH domain leucine-rich repeat-containing protein phosphatase 2 (EC 3.1.3.16) (PH domain leucine-rich repeat-containing protein phosphatase-like) (PHLPP-like)
Q8BXC6	COMD2_MOUSE	COMM domain-containing protein 2
Q8BXK9	CLIC5_MOUSE	Chloride intracellular channel protein 5
Q8BYH8	CHD9_MOUSE	Chromodomain-helicase-DNA-binding protein 9 (CHD-9) (EC 3.6.4.12) (ATP-dependent helicase CHD9) (PPAR-alpha-interacting complex protein

		320 kDa) (Peroxisomal proliferator-activated receptor A-interacting complex 320 kDa protein)
Q8BYK5	PHAR3_MOUSE	Phosphatase and actin regulator 3 (Scaffold-associated PP1-inhibiting protein) (Scapinin)
Q8BYU6	TOIP2_MOUSE	Torsin-1A-interacting protein 2
Q8BYY4	TT39B_MOUSE	Tetratricopeptide repeat protein 39B (TPR repeat protein 39B)
Q8BYY9	SPA3B_MOUSE	Serine protease inhibitor A3B (Serpin A3B)
Q8BYZ7	ELMO3_MOUSE	Engulfment and cell motility protein 3
Q8BZ05	ARAP2_MOUSE	Arf-GAP with Rho-GAP domain, ANK repeat and PH domain-containing protein 2 (Centaurin-delta-1) (Cnt-d1)
Q8BZ25	ANKK1_MOUSE	Ankyrin repeat and protein kinase domain-containing protein 1 (EC 2.7.11.1)
Q8BZK4	S35F4_MOUSE	Solute carrier family 35 member F4
Q8BZT5	LRC19_MOUSE	Leucine-rich repeat-containing protein 19
Q8C031	LRC4C_MOUSE	Leucine-rich repeat-containing protein 4C (Netrin-G1 ligand) (NGL-1)
Q8C0D0	TRUB1_MOUSE	Probable tRNA pseudouridine synthase 1 (EC 5.4.99.-)
Q8C0E3	TRI47_MOUSE	E3 ubiquitin-protein ligase TRIM47 (EC 2.3.2.27) (Tripartite motif-containing protein 47)
Q8C0F9	PRS35_MOUSE	Inactive serine protease 35
Q8C0I1	ADAS_MOUSE	Alkyldihydroxyacetonephosphate synthase, peroxisomal (Alkyl-DHAP synthase) (EC 2.5.1.26) (Alkylglycerone-phosphate synthase)
Q8C0I4	EPC2_MOUSE	Enhancer of polycomb homolog 2 (EPC-like)
Q8C0V0	TLK1_MOUSE	Serine/threonine-protein kinase tousled-like 1 (EC 2.7.11.1) (Tousled-like kinase 1)
Q8C0Y0	PP4R4_MOUSE	Serine/threonine-protein phosphatase 4 regulatory subunit 4
Q8C0Y1	TGIF2_MOUSE	Homeobox protein TGIF2 (5'-TG-3'-interacting factor 2) (TGF-beta-induced transcription factor 2) (TGFB-induced factor 2)
Q8C142	ARH_MOUSE	Low density lipoprotein receptor adapter protein 1 (Autosomal recessive hypercholesterolemia protein homolog)
Q8C190	VP9D1_MOUSE	VPS9 domain-containing protein 1 (5-day ovary-specific transcript 1 protein)
Q8C436	MT21D_MOUSE	Protein-lysine methyltransferase METTL21D (EC 2.1.1.-) (Methyltransferase-like protein 21D) (VCP lysine methyltransferase) (VCP-KMT) (Valosin-containing protein lysine methyltransferase)
Q8C456	FRITZ_MOUSE	WD repeat-containing and planar cell polarity effector protein fritz homolog (mFrtz) (Homolog-

		13) (WD repeat-containing and planar cell polarity effector protein)
Q8C5L3	CNOT2_MOUSE	CCR4-NOT transcription complex subunit 2 (CCR4-associated factor 2)
Q8C5V5	WDR27_MOUSE	WD repeat-containing protein 27
Q8C863	ITCH_MOUSE	E3 ubiquitin-protein ligase Itchy (EC 2.3.2.26) (HECT-type E3 ubiquitin transferase Itchy homolog)
Q8C8H8	KY_MOUSE	Kyphoscoliosis peptidase (EC 3.4.-.-)
Q8CAY6	THIC_MOUSE	Acetyl-CoA acetyltransferase, cytosolic (EC 2.3.1.9) (Cytosolic acetoacetyl-CoA thiolase)
Q8CB96	RASF4_MOUSE	Ras association domain-containing protein 4
Q8CBB9	RSAD2_MOUSE	Radical S-adenosyl methionine domain-containing protein 2 (Viperin) (Virus inhibitory protein, endoplasmic reticulum-associated, interferon-inducible)
Q8CD15	RIOX2_MOUSE	Ribosomal oxygenase 2 (Bifunctional lysine-specific demethylase and histidyl-hydroxylase MINA) (EC 1.14.11.-) (Histone lysine demethylase MINA) (MYC-induced nuclear antigen)
Q8CDG3	VCIP1_MOUSE	Deubiquitinating protein VCIP135 (EC 3.4.19.12) (Valosin-containing protein p97/p47 complex-interacting protein 1) (Valosin-containing protein p97/p47 complex-interacting protein p135)
Q8CDK3	IQUB_MOUSE	IQ and ubiquitin-like domain-containing protein
Q8CES0	NAA30_MOUSE	N-alpha-acetyltransferase 30 (EC 2.3.1.256) (N-acetyltransferase 12) (N-acetyltransferase MAK3 homolog) (NatC catalytic subunit)
Q8CFA1	IRAK2_MOUSE	Interleukin-1 receptor-associated kinase-like 2 (IRAK-2) (mu-IRAK-2)
Q8CFD4	SNX8_MOUSE	Sorting nexin-8
Q8CFE4	SCYL2_MOUSE	SCY1-like protein 2 (Coated vesicle-associated kinase of 104 kDa)
Q8CG48	SMC2_MOUSE	Structural maintenance of chromosomes protein 2 (SMC protein 2) (SMC-2) (Chromosome-associated protein E) (FGF-inducible protein 16) (XCAP-E homolog)
Q8CG73	FTM_MOUSE	Protein fantom (Nephrocystin-8) (RPGR-interacting protein 1-like protein) (RPGRIP1-like protein)
Q8CGA3	LAT4_MOUSE	Large neutral amino acids transporter small subunit 4 (L-type amino acid transporter 4) (Solute carrier family 43 member 2)

Q8CGB3	UACA_MOUSE	Uveal autoantigen with coiled-coil domains and ankyrin repeats (Nuclear membrane-binding protein) (Nucling)
Q8CGC4	LS14B_MOUSE	Protein LSM14 homolog B (Protein FAM61B) (RNA-associated protein 55B) (mRAP55B)
Q8CGF6	WDR47_MOUSE	WD repeat-containing protein 47 (Neuronal enriched MAP interacting protein) (Nemitin)
Q8CGM2	RP1L1_MOUSE	Retinitis pigmentosa 1-like 1 protein (Retinitis pigmentosa 1-like protein 1)
Q8CHT3	INT5_MOUSE	Integrator complex subunit 5 (Int5)
Q8CI32	BAG5_MOUSE	BAG family molecular chaperone regulator 5 (BAG-5) (Bcl-2-associated athanogene 5)
Q8CIB9	ESCO2_MOUSE	N-acetyltransferase ESCO2 (EC 2.3.1.-) (Establishment of cohesion 1 homolog 2) (ECO1 homolog 2)
Q8CIM5	GPR84_MOUSE	G-protein coupled receptor 84
Q8CIP5	DISP2_MOUSE	Protein dispatched homolog 2
Q8CIR4	TRPM6_MOUSE	Transient receptor potential cation channel subfamily M member 6 (EC 2.7.11.1) (Channel kinase 2) (Melastatin-related TRP cation channel 6)
Q8CIV3	LIPH_MOUSE	Lipase member H (EC 3.1.1.-)
Q8CIV8	TBCE_MOUSE	Tubulin-specific chaperone E (Tubulin-folding cofactor E)
Q8CJG0	AGO2_MOUSE	Protein argonaute-2 (Argonaute2) (mAgo2) (EC 3.1.26.n2) (Argonaute RISC catalytic component 2) (Eukaryotic translation initiation factor 2C 2) (eIF-2C 2) (eIF2C 2) (Piwi/argonaute family protein meIF2C2) (Protein slicer)
Q8K021	SCAM1_MOUSE	Secretory carrier-associated membrane protein 1 (Secretory carrier membrane protein 1)
Q8K093	TRHDE_MOUSE	Thyrotropin-releasing hormone-degrading ectoenzyme (TRH-DE) (TRH-degrading ectoenzyme) (EC 3.4.19.6) (Pyroglutamyl-peptidase II) (PAP-II) (TRH-specific aminopeptidase) (Thyroliberinase)
Q8K0B2	LMBD1_MOUSE	Probable lysosomal cobalamin transporter (LMBR1 domain-containing protein 1) (Protein N90b)
Q8K0E1	KCD15_MOUSE	BTB/POZ domain-containing protein KCTD15 (Potassium channel tetramerization domain-containing protein 15)
Q8K0Q5	RHG18_MOUSE	Rho GTPase-activating protein 18 (Rho-type GTPase-activating protein 18)
Q8K1C0	ANGE2_MOUSE	Protein angel homolog 2
Q8K1C9	LRC41_MOUSE	Leucine-rich repeat-containing protein 41 (Protein Muf1)

Q8K1K4	CENPI_MOUSE	Centromere protein I (CENP-I) (FSH primary response protein 1) (Follicle-stimulating hormone primary response protein)
Q8K1K6	SPB10_MOUSE	Serpin B10
Q8K1Y2	KPCD3_MOUSE	Serine/threonine-protein kinase D3 (EC 2.7.11.13) (Protein kinase C nu type) (nPKC-nu)
Q8K2G4	BBS7_MOUSE	Bardet-Biedl syndrome 7 protein homolog (BBS2-like protein 1)
Q8K2J7	RELL1_MOUSE	RELT-like protein 1
Q8K2U2	ALKB6_MOUSE	Alpha-ketoglutarate-dependent dioxygenase alkB homolog 6 (EC 1.14.11.-) (Alkylated DNA repair protein alkB homolog 6)
Q8K2V6	IPO11_MOUSE	Importin-11 (Imp11) (Ran-binding protein 11) (RanBP11)
Q8K2X2	RBM48_MOUSE	RNA-binding protein 48
Q8K327	CHAP1_MOUSE	Chromosome alignment-maintaining phosphoprotein 1 (Zinc finger protein 828)
Q8K352	SASH3_MOUSE	SAM and SH3 domain-containing protein 3 (SH3 protein expressed in lymphocytes)
Q8K3Q3	FOXN4_MOUSE	Forkhead box protein N4
Q8K3R3	PLCD4_MOUSE	1-phosphatidylinositol 4,5-bisphosphate phosphodiesterase delta-4 (EC 3.1.4.11) (Phosphoinositide phospholipase C-delta-4) (Phospholipase C-delta-4) (PLC-delta-4)
Q8K458	PKR2_MOUSE	Prokineticin receptor 2 (PK-R2) (G-protein coupled receptor 73-like 1)
Q8K482	EMIL2_MOUSE	EMILIN-2 (Basilin) (Elastin microfibril interface-located protein 2) (Elastin microfibril interfacer 2)
Q8QZR5	ALAT1_MOUSE	Alanine aminotransferase 1 (ALT1) (EC 2.6.1.2) (Glutamate pyruvate transaminase 1) (GPT 1) (Glutamic--alanine transaminase 1) (Glutamic--pyruvic transaminase 1)
Q8QZY9	SF3B4_MOUSE	Splicing factor 3B subunit 4
Q8R0F3	SUMF1_MOUSE	Formylglycine-generating enzyme (FGE) (EC 1.8.3.7) (C-alpha-formylglycine-generating enzyme 1) (Sulfatase-modifying factor 1)
Q8R0G7	SPNS1_MOUSE	Protein spinster homolog 1
Q8R0G9	NU133_MOUSE	Nuclear pore complex protein Nup133 (133 kDa nucleoporin) (Nucleoporin Nup133)
Q8R0X7	SGPL1_MOUSE	Sphingosine-1-phosphate lyase 1 (S1PL) (SP-lyase 1) (SPL 1) (mSPL) (EC 4.1.2.27) (Sphingosine-1-phosphate aldolase)
Q8R179	KBTB4_MOUSE	Kelch repeat and BTB domain-containing protein 4 (BTB and kelch domain-containing protein 4)

Q8R1Q3	ANGL7_MOUSE	Angiopoietin-related protein 7 (Angiopoietin-like protein 7)
Q8R1T1	CHMP7_MOUSE	Charged multivesicular body protein 7 (Chromatin-modifying protein 7)
Q8R1U1	COG4_MOUSE	Conserved oligomeric Golgi complex subunit 4 (COG complex subunit 4) (Component of oligomeric Golgi complex 4)
Q8R1W2	GSG1_MOUSE	Germ cell-specific gene 1 protein (Germ cell-associated protein 1)
Q8R2U4	NTM1A_MOUSE	N-terminal Xaa-Pro-Lys N-methyltransferase 1 (EC 2.1.1.244) (Alpha N-terminal protein methyltransferase 1A) (Methyltransferase-like protein 11A) (X-Pro-Lys N-terminal protein methyltransferase 1A) (NTM1A) [Cleaved into: N-terminal Xaa-Pro-Lys N-methyltransferase 1, N-terminally processed]
Q8R2V5	ADAP2_MOUSE	Arf-GAP with dual PH domain-containing protein 2 (Centaurin-alpha-2) (Cnt-a2)
Q8R3Y8	I2BP1_MOUSE	Interferon regulatory factor 2-binding protein 1 (IRF-2-binding protein 1) (IRF-2BP1) (Probable E3 ubiquitin-protein ligase IRF2BP1) (EC 2.3.2.27) (Probable RING-type E3 ubiquitin transferase IRF2BP1)
Q8R418	DICER_MOUSE	Endoribonuclease Dicer (EC 3.1.26.3) (Double-strand-specific ribonuclease mDCR-1)
Q8R4H4	CBPA5_MOUSE	Carboxypeptidase A5 (EC 3.4.17.-)
Q8R4V4	CBPZ_MOUSE	Carboxypeptidase Z (CPZ) (EC 3.4.17.-)
Q8R502	LRC8C_MOUSE	Volume-regulated anion channel subunit LRRC8C (Factor for adipocyte differentiation 158) (Leucine-rich repeat-containing protein 8C)
Q8R5K2	UBP33_MOUSE	Ubiquitin carboxyl-terminal hydrolase 33 (EC 3.4.19.12) (Deubiquitinating enzyme 33) (Ubiquitin thioesterase 33) (Ubiquitin-specific-processing protease 33) (VHL-interacting deubiquitinating enzyme 1)
Q8R5L3	VPS39_MOUSE	Vam6/Vps39-like protein
Q8VBT0	TMX1_MOUSE	Thioredoxin-related transmembrane protein 1 (Thioredoxin domain-containing protein 1)
Q8VBT2	SDHL_MOUSE	L-serine dehydratase/L-threonine deaminase (SDH) (EC 4.3.1.17) (L-serine deaminase) (L-threonine dehydratase) (TDH) (EC 4.3.1.19)
Q8VBV3	EXOS2_MOUSE	Exosome complex component RRP4 (Exosome component 2) (Ribosomal RNA-processing protein 4)

Q8VBV7	CSN8_MOUSE	COP9 signalosome complex subunit 8 (SGN8) (Signalosome subunit 8) (COP9 homolog) (JAB1-containing signalosome subunit 8)
Q8VC30	TKFC_MOUSE	Triokinase/FMN cyclase (Bifunctional ATP-dependent dihydroxyacetone kinase/FAD-AMP lyase (cyclizing)) [Includes: ATP-dependent dihydroxyacetone kinase (DHA kinase) (EC 2.7.1.28) (EC 2.7.1.29) (Glycerone kinase) (Triokinase) (Triose kinase); FAD-AMP lyase (cyclizing) (EC 4.6.1.15) (FAD-AMP lyase (cyclic FMN forming)) (FMN cyclase)]
Q8VC49	IF27B_MOUSE	Interferon alpha-inducible protein 27-like protein 2B (Interferon-stimulated gene 12 protein B2) (ISG12(b2))
Q8VC56	RNF8_MOUSE	E3 ubiquitin-protein ligase RNF8 (EC 2.3.2.27) (ActA-interacting protein 37) (AIP37) (LaXp180) (RING finger protein 8) (RING-type E3 ubiquitin transferase RNF8)
Q8VC69	S22A6_MOUSE	Solute carrier family 22 member 6 (Kidney-specific transport protein) (Novel kidney transcript) (mNKT) (Organic anion transporter 1) (Renal organic anion transporter 1) (mROAT1)
Q8VCD3	LMA1L_MOUSE	Protein ERGIC-53-like (ERGIC53-like protein) (Lectin mannose-binding 1-like) (LMAN1-like protein) (Sublingual acinar membrane protein) (Slamp)
Q8VCT4	CES1D_MOUSE	Carboxylesterase 1D (Carboxylesterase 3) (EC 3.1.1.1) (EC 3.1.1.67) (Fatty acid ethyl ester synthase) (FAEE synthase) (Triacylglycerol hydrolase) (TGH)
Q8VCW4	UN93B_MOUSE	Protein unc-93 homolog B1 (Unc-93B1)
Q8VD62	CK068_MOUSE	UPF0696 protein C11orf68 homolog (Basophilic leukemia-expressed protein Bles03) (Protein WF-3)
Q8VDD8	WASH1_MOUSE	WASH complex subunit 1 (WAS protein family homolog 1)
Q8VDP4	CCAR2_MOUSE	Cell cycle and apoptosis regulator protein 2 (Cell division cycle and apoptosis regulator protein 2)
Q8VDZ4	ZDHC5_MOUSE	Palmitoyltransferase ZDHHC5 (EC 2.3.1.225) (Zinc finger DHHC domain-containing protein 5) (DHHC-5)
Q8VE08	FBX33_MOUSE	F-box only protein 33 (AIG30-12-1)
Q8VE96	S35F6_MOUSE	Solute carrier family 35 member F6 (ANT2-binding protein) (ANT2BP) (Transport and Golgi organization 9 homolog)
Q8VEE0	RPE_MOUSE	Ribulose-phosphate 3-epimerase (EC 5.1.3.1) (Ribulose-5-phosphate-epimerase)

Q8VEH5	EPMIP_MOUSE	EPM2A-interacting protein 1 (Laforin-interacting protein)
Q8VFV4	O1440_MOUSE	Olfactory receptor 1440 (Olfactory receptor 202-4)
Q8VG09	OL502_MOUSE	Olfactory receptor 502 (Olfactory receptor 204-8)
Q8VGI4	OL476_MOUSE	Olfactory receptor 476 (Olfactory receptor 204-3)
Q8VH51	RBM39_MOUSE	RNA-binding protein 39 (Coactivator of activating protein 1 and estrogen receptors) (Coactivator of AP-1 and ERs) (RNA-binding motif protein 39) (RNA-binding region-containing protein 2) (Transcription coactivator CAPER)
Q8VHF2	CDHR5_MOUSE	Cadherin-related family member 5 (Mu-protocadherin)
Q8VHR0	PCD18_MOUSE	Protocadherin 18
Q8VIC6	V1R51_MOUSE	Vomeronasal type-1 receptor 51 (Pheromone receptor 1) (Vomeronasal type-1 receptor A1) (mV1R1) (Vomeronasal type-1 receptor A8)
Q8VIM5	MYCD_MOUSE	Myocardin (Basic SAP coiled-coil transcription activator 2) (SRF cofactor protein)
Q91VF6	COQA1_MOUSE	Collagen alpha-1(XXVI) chain (Alpha-1 type XXVI collagen) (EMI domain-containing protein 2) (Emilin and multimerin domain-containing protein 2) (Emu2)
Q91VH6	MEMO1_MOUSE	Protein MEMO1 (Mediator of ErbB2-driven cell motility 1) (Memo-1)
Q91VS7	MGST1_MOUSE	Microsomal glutathione S-transferase 1 (Microsomal GST-1) (EC 2.5.1.18) (Microsomal GST-I)
Q91VX9	TM168_MOUSE	Transmembrane protein 168
Q91W27	TIP39_MOUSE	Tuberoinfundibular peptide of 39 residues (TIP39) (Parathyroid hormone 2)
Q91W64	CP270_MOUSE	Cytochrome P450 2C70 (EC 1.14.14.1) (CYPIIC70)
Q91WB7	UBTD1_MOUSE	Ubiquitin domain-containing protein 1
Q91WC3	ACSL6_MOUSE	Long-chain-fatty-acid--CoA ligase 6 (EC 6.2.1.3) (Arachidonate--CoA ligase) (EC 6.2.1.15) (Long-chain acyl-CoA synthetase 6) (LACS 6)
Q91WC7	NPAL2_MOUSE	NIPA-like protein 2
Q91WE1	SNX15_MOUSE	Sorting nexin-15
Q91WE3	RPP25_MOUSE	Ribonuclease P protein subunit p25 (RNase P protein subunit p25) (EC 3.1.26.5)
Q91WG4	ELP2_MOUSE	Elongator complex protein 2 (ELP2) (STAT3-interacting protein 1) (StIP1)
Q91WM1	STRBP_MOUSE	Spermatid perinuclear RNA-binding protein
Q91WN2	T150A_MOUSE	Transmembrane protein 150A (Transmembrane protein 150)

Q91WT8	RBM47_MOUSE	RNA-binding protein 47 (RNA-binding motif protein 47)
Q91WU4	TMCO4_MOUSE	Transmembrane and coiled-coil domain-containing protein 4
Q91X77	CY250_MOUSE	Cytochrome P450 2C50 (EC 1.14.14.1) (CYPIIC50)
Q91XY4	PCDG4_MOUSE	Protocadherin gamma-A4 (PCDH-gamma-A4)
Q91Y02	PCDBI_MOUSE	Protocadherin beta-18 (PCDH-beta-18)
Q91YE6	IPO9_MOUSE	Importin-9 (Imp9) (Importin-9a) (Imp9a) (Importin-9b) (Imp9b) (Ran-binding protein 9) (RanBP9)
Q91YQ7	RMD5B_MOUSE	E3 ubiquitin-protein transferase RMND5B (EC 2.3.2.27) (Protein RMD5 homolog B)
Q91YR7	PRP6_MOUSE	Pre-mRNA-processing factor 6 (PRP6 homolog) (U5 snRNP-associated 102 kDa protein) (U5-102 kDa protein)
Q91ZB0	ALPK2_MOUSE	Alpha-protein kinase 2 (EC 2.7.11.1) (Heart alpha-protein kinase)
Q91ZC4	MRGA8_MOUSE	Mas-related G-protein coupled receptor member A8
Q91ZR4	NEK8_MOUSE	Serine/threonine-protein kinase Nek8 (EC 2.7.11.1) (Never in mitosis A-related kinase 8) (NimA-related protein kinase 8)
Q920B0	FRM4B_MOUSE	FERM domain-containing protein 4B (GRP1-binding protein GRSP1) (Golgi-associated band 4.1-like protein) (GOBLIN)
Q921G7	ETFD_MOUSE	Electron transfer flavoprotein-ubiquinone oxidoreductase, mitochondrial (ETF-QO) (ETF-ubiquinone oxidoreductase) (EC 1.5.5.1) (Electron-transferring-flavoprotein dehydrogenase) (ETF dehydrogenase)
Q921G8	GCP2_MOUSE	Gamma-tubulin complex component 2 (GCP-2)
Q921K8	TCAF2_MOUSE	TRPM8 channel-associated factor 2 (TRP channel-associated factor 2)
Q922J3	CLIP1_MOUSE	CAP-Gly domain-containing linker protein 1 (Cytoplasmic linker protein 170) (CLIP-170) (Restin)
Q922K7	NOP2_MOUSE	Probable 28S rRNA (cytosine-C(5))-methyltransferase (EC 2.1.1.-) (Nucleolar protein 1) (Nucleolar protein 2 homolog) (Proliferating-cell nucleolar antigen p120) (Proliferation-associated nucleolar protein p120)
Q923E4	SIR1_MOUSE	NAD-dependent protein deacetylase sirtuin-1 (EC 2.3.1.286) (NAD-dependent protein deacylase sirtuin-1) (EC 2.3.1.-) (Regulatory protein SIR2 homolog 1) (SIR2-like protein 1) (SIR2alpha)

		(Sir2) (mSIR2a) [Cleaved into: SirtT1 75 kDa fragment (75SirT1)]
Q923L3	CSMD1_MOUSE	CUB and sushi domain-containing protein 1 (CUB and sushi multiple domains protein 1)
Q924A2	CIC_MOUSE	Protein capicua homolog
Q924N9	PRS28_MOUSE	Serine protease 28 (EC 3.4.21.-) (Implantation serine proteinase 1) (ISP-1) (Strypsin) (Tryptase-like proteinase)
Q925N2	SFXN2_MOUSE	Sideroflexin-2
Q925Q3	NCLX_MOUSE	Mitochondrial sodium/calcium exchanger protein (Na(+)/K(+)/Ca(2+)-exchange protein 6) (Sodium/calcium exchanger protein, mitochondrial) (Sodium/potassium/calcium exchanger 6) (Solute carrier family 24 member 6) (Solute carrier family 8 member B1)
Q99J45	NRBP_MOUSE	Nuclear receptor-binding protein (HLS7-interacting protein kinase) (MLF1 adapter molecule)
Q99JB2	STML2_MOUSE	Stomatin-like protein 2, mitochondrial (SLP-2) (mslp2)
Q99JB8	PACN3_MOUSE	Protein kinase C and casein kinase II substrate protein 3
Q99JR1	SFXN1_MOUSE	Sideroflexin-1
Q99JR5	TINAL_MOUSE	Tubulointerstitial nephritis antigen-like (Adrenocortical zonation factor 1) (AZ-1) (Androgen-regulated gene 1 protein) (Tubulointerstitial nephritis antigen-related protein) (TARP)
Q99JV5	STAR4_MOUSE	StAR-related lipid transfer protein 4 (START domain-containing protein 4) (StARD4)
Q99JW5	EPCAM_MOUSE	Epithelial cell adhesion molecule (Ep-CAM) (Epithelial glycoprotein 314) (EGP314) (mEGP314) (Protein 289A) (Tumor-associated calcium signal transducer 1) (CD antigen CD326)
Q99KE1	MAOM_MOUSE	NAD-dependent malic enzyme, mitochondrial (NAD-ME) (EC 1.1.1.38) (Malic enzyme 2)
Q99KW3	TARA_MOUSE	TRIO and F-actin-binding protein (Protein Tara) (Trio-associated repeat on actin)
Q99L88	SNTB1_MOUSE	Beta-1-syntrophin (59 kDa dystrophin-associated protein A1 basic component 1) (DAPA1B) (Syntrophin-2)
Q99LE6	ABCF2_MOUSE	ATP-binding cassette sub-family F member 2
Q99LH1	NOG2_MOUSE	Nucleolar GTP-binding protein 2
Q99LL3	CHSTC_MOUSE	Carbohydrate sulfotransferase 12 (EC 2.8.2.5) (Chondroitin 4-O-sulfotransferase 2) (Chondroitin 4-sulfotransferase 2) (C4ST-2) (C4ST2)

Q99M03	RWD2B_MOUSE	RWD domain-containing protein 2B
Q99MR0	ACL6B_MOUSE	Actin-like protein 6B (53 kDa BRG1-associated factor B) (Actin-related protein Baf53b) (ArpN-alpha) (ArpNa) (BRG1-associated factor 53B) (BAF53B)
Q99MR3	S12A9_MOUSE	Solute carrier family 12 member 9 (Cation-chloride cotransporter-interacting protein 1) (Potassium-chloride transporter 9)
Q99MX7	T121B_MOUSE	Transmembrane protein 121B (Cat eye syndrome critical region protein 6 homolog)
Q99MZ6	MYO7B_MOUSE	Unconventional myosin-VIIb
Q99N05	M4A4D_MOUSE	Membrane-spanning 4-domains subfamily A member 4D
Q99PL6	UBXN6_MOUSE	UBX domain-containing protein 6 (UBX domain-containing protein 1)
Q99PP6	TR34A_MOUSE	Tripartite motif-containing protein 34A
Q99PT3	IN80B_MOUSE	INO80 complex subunit B (High mobility group AT-hook 1-like 4) (PAP-1-associated protein 1) (PAPA-1) (Zinc finger HIT domain-containing protein 4)
Q9CQ02	COMD4_MOUSE	COMM domain-containing protein 4
Q9CQ80	VPS25_MOUSE	Vacuolar protein-sorting-associated protein 25 (ESCRT-II complex subunit VPS25)
Q9CQ92	FIS1_MOUSE	Mitochondrial fission 1 protein (FIS1 homolog) (Tetratricopeptide repeat protein 11) (TPR repeat protein 11)
Q9CQF9	PCYOX_MOUSE	Prenylcysteine oxidase (EC 1.8.3.5)
Q9CQM2	ERD22_MOUSE	ER lumen protein-retaining receptor 2 (KDEL endoplasmic reticulum protein retention receptor 2) (KDEL receptor 2)
Q9CQT5	POMP_MOUSE	Proteasome maturation protein (Proteassemblin) (Protein UMP1 homolog) (mUMP1)
Q9CSN1	SNW1_MOUSE	SNW domain-containing protein 1 (Nuclear protein SkiP) (Ski-interacting protein)
Q9CSP9	TTC14_MOUSE	Tetratricopeptide repeat protein 14 (TPR repeat protein 14)
Q9CT10	RANB3_MOUSE	Ran-binding protein 3 (RanBP3)
Q9CU65	ZMYM2_MOUSE	Zinc finger MYM-type protein 2 (Zinc finger protein 198)
Q9CWG8	NDUF7_MOUSE	Protein arginine methyltransferase NDUFAF7, mitochondrial (EC 2.1.1.320) (NADH dehydrogenase [ubiquinone] complex I, assembly factor 7) (Protein midA homolog)

Q9CWT6	DDX28_MOUSE	Probable ATP-dependent RNA helicase DDX28 (EC 3.6.4.13) (Mitochondrial DEAD box protein 28)
Q9CWU0	TDR12_MOUSE	Putative ATP-dependent RNA helicase TDRD12 (EC 3.6.4.13) (ES cell-associated transcript 8 protein) (Tudor domain-containing protein 12)
Q9CX53	GEMI6_MOUSE	Gem-associated protein 6 (Gemin-6)
Q9CXE7	TMED5_MOUSE	Transmembrane emp24 domain-containing protein 5 (p24 family protein gamma-2) (p24gamma2)
Q9CXJ1	SYEM_MOUSE	Probable glutamate--tRNA ligase, mitochondrial (EC 6.1.1.17) (Glutamyl-tRNA synthetase) (GluRS)
Q9CXP9	EXO5_MOUSE	Exonuclease V (Exo V) (mExo5) (EC 3.1.-.-) (Defects in morphology protein 1 homolog)
Q9CY52	THG1_MOUSE	Probable tRNA(His) guanylyltransferase (EC 2.7.7.79) (tRNA-histidine guanylyltransferase)
Q9CYB0	TRI13_MOUSE	E3 ubiquitin-protein ligase TRIM13 (EC 2.3.2.27) (Putative tumor suppressor RFP2) (RING-type E3 ubiquitin transferase TRIM13) (Ret finger protein 2) (Tripartite motif-containing protein 13)
Q9CYN2	SPCS2_MOUSE	Signal peptidase complex subunit 2 (EC 3.4.-.-) (Microsomal signal peptidase 25 kDa subunit) (SPase 25 kDa subunit)
Q9CYQ7	NARF_MOUSE	Nuclear prelamin A recognition factor (Iron-only hydrogenase-like protein 2) (IOP2)
Q9CYS6	CB072_MOUSE	Uncharacterized protein C2orf72 homolog
Q9CZ13	QCR1_MOUSE	Cytochrome b-c1 complex subunit 1, mitochondrial (Complex III subunit 1) (Core protein I) (Ubiquinol-cytochrome-c reductase complex core protein 1)
Q9CZB3	THUM2_MOUSE	THUMP domain-containing protein 2
Q9CZT5	VASN_MOUSE	Vasorin (Protein slit-like 2)
Q9CZW5	TOM70_MOUSE	Mitochondrial import receptor subunit TOM70 (Mitochondrial precursor proteins import receptor) (Translocase of outer membrane 70 kDa subunit) (Translocase of outer mitochondrial membrane protein 70)
Q9CZX0	ELP3_MOUSE	Elongator complex protein 3 (Protein lysine acetyltransferase ELP3) (EC 2.3.1.-) (tRNA uridine(34) acetyltransferase) (EC 2.3.1.-)
Q9D067	MDM1_MOUSE	Nuclear protein MDM1 (Mdm4 transformed 3T3 cell double minute 1 protein) (Mouse double minute 1)
Q9D071	MMS19_MOUSE	MMS19 nucleotide excision repair protein homolog (MET18 homolog) (MMS19-like protein)

Q9D0J4	ARL2_MOUSE	ADP-ribosylation factor-like protein 2
Q9D0L8	MCES_MOUSE	mRNA cap guanine-N7 methyltransferase (EC 2.1.1.56) (RG7MT1) (mRNA (guanine-N(7)-)-methyltransferase) (mRNA cap methyltransferase)
Q9D0Z3	TMM53_MOUSE	Transmembrane protein 53
Q9D115	ZN706_MOUSE	Zinc finger protein 706
Q9D119	PPR27_MOUSE	Protein phosphatase 1 regulatory subunit 27 (Dysferlin-interacting protein 1)
Q9D1E6	TBCB_MOUSE	Tubulin-folding cofactor B (Cytoskeleton-associated protein 1) (Cytoskeleton-associated protein CKAPI) (Tubulin-specific chaperone B)
Q9D1L9	LTOR5_MOUSE	Ragulator complex protein LAMTOR5 (Late endosomal/lysosomal adaptor and MAPK and MTOR activator 5)
Q9D2G2	ODO2_MOUSE	Dihydrolipoyllysine-residue succinyltransferase component of 2-oxoglutarate dehydrogenase complex, mitochondrial (EC 2.3.1.61) (2-oxoglutarate dehydrogenase complex component E2) (OGDC-E2) (Dihydrolipoamide succinyltransferase component of 2-oxoglutarate dehydrogenase complex) (E2K)
Q9D3J9	CX021_MOUSE	Protein CXorf21 homolog
Q9D4A5	CP086_MOUSE	Uncharacterized protein C16orf86 homolog
Q9D4C9	CLVS1_MOUSE	Clavesin-1 (Retinaldehyde-binding protein 1-like 1)
Q9D618	KBTBC_MOUSE	Kelch repeat and BTB domain-containing protein 12 (Kelch domain-containing protein 6)
Q9D646	KRT34_MOUSE	Keratin, type I cuticular Ha4 (Hair keratin, type I Ha4) (Keratin-34) (K34)
Q9D684	RIN2_MOUSE	Ras and Rab interactor 2 (Ras interaction/interference protein 2)
Q9D695	SPB7_MOUSE	Serpin B7 (Megsin)
Q9D6J1	CERS4_MOUSE	Ceramide synthase 4 (CerS4) (EC 2.3.1.-) (LAG1 longevity assurance homolog 4) (Sphingosine N-acyltransferase CERS4) (EC 2.3.1.24) (Translocating chain-associating membrane protein homolog 1) (TRAM homolog 1)
Q9D6R2	IDH3A_MOUSE	Isocitrate dehydrogenase [NAD] subunit alpha, mitochondrial (EC 1.1.1.41) (Isocitric dehydrogenase subunit alpha) (NAD(+)-specific ICDH subunit alpha)
Q9D799	FMT_MOUSE	Methionyl-tRNA formyltransferase, mitochondrial (MtFMT) (EC 2.1.2.9)
Q9D7J9	ECHD3_MOUSE	Enoyl-CoA hydratase domain-containing protein 3, mitochondrial

Q9D7L8	TMIG1_MOUSE	Transmembrane and immunoglobulin domain-containing protein 1
Q9D8C6	MED11_MOUSE	Mediator of RNA polymerase II transcription subunit 11 (Mediator complex subunit 11)
Q9D8N1	CK024_MOUSE	Uncharacterized protein C11orf24 homolog
Q9D8Z1	ASCC1_MOUSE	Activating signal cointegrator 1 complex subunit 1 (ASC-1 complex subunit p50) (Trip4 complex subunit p50)
Q9D920	BORC5_MOUSE	BLOC-1-related complex subunit 5
Q9D924	ISCA1_MOUSE	Iron-sulfur cluster assembly 1 homolog, mitochondrial (HESB-like domain-containing protein 2) (Iron-sulfur assembly protein IscA)
Q9D994	WDR38_MOUSE	WD repeat-containing protein 38
Q9D9G3	CHIC2_MOUSE	Cysteine-rich hydrophobic domain-containing protein 2
Q9DA60	TEX44_MOUSE	Testis-expressed protein 44
Q9DA80	RSH3B_MOUSE	Radial spoke head protein 3 homolog B (A-kinase anchor protein RSPH3B) (Radial spoke head-like protein 2B)
Q9DAM2	EFCB9_MOUSE	EF-hand calcium-binding domain-containing protein 9
Q9DAZ2	PR2B1_MOUSE	Prolactin-2B1 (Placental prolactin-like protein K) (PLP-K) (PRL-like protein K)
Q9DB91	MED29_MOUSE	Mediator of RNA polymerase II transcription subunit 29 (Intersex-like protein) (Mediator complex subunit 29)
Q9DBC0	SELO_MOUSE	Protein adenylyltransferase SelO, mitochondrial (EC 2.7.7.-) (EC 2.7.7.n1) (Selenoprotein O) (SelO)
Q9DBD2	MARH8_MOUSE	E3 ubiquitin-protein ligase MARCH8 (EC 2.3.2.27) (Cellular modulator of immune recognition) (c-MIR) (Membrane-associated RING finger protein 8) (Membrane-associated RING-CH protein VIII) (MARCH-VIII) (RING-type E3 ubiquitin transferase MARCH8)
Q9DBH5	LMAN2_MOUSE	Vesicular integral-membrane protein VIP36 (Lectin mannose-binding 2) (Vesicular integral-membrane protein 36) (VIP36)
Q9DBN5	LONP2_MOUSE	Lon protease homolog 2, peroxisomal (EC 3.4.21.53) (Lon protease-like protein 2) (Lon protease 2) (Peroxisomal Lon protease)
Q9DBS1	TMM43_MOUSE	Transmembrane protein 43 (Protein LUMA)
Q9DBS8	POC5_MOUSE	Centrosomal protein POC5 (Protein of centriole 5)
Q9DBU2	NAA60_MOUSE	N-alpha-acetyltransferase 60 (EC 2.3.1.259) (Histone acetyltransferase type B protein 4)

		(HAT4) (EC 2.3.1.48) (N-acetyltransferase 15) (N-alpha-acetyltransferase F) (NatF)
Q9DC11	PXDC2_MOUSE	Plexin domain-containing protein 2 (Tumor endothelial marker 7-related protein)
Q9DC16	ERGI1_MOUSE	Endoplasmic reticulum-Golgi intermediate compartment protein 1 (ER-Golgi intermediate compartment 32 kDa protein) (ERGIC-32)
Q9DC37	MFSD1_MOUSE	Major facilitator superfamily domain-containing protein 1
Q9DCL8	IPP2_MOUSE	Protein phosphatase inhibitor 2 (IPP-2)
Q9DCM2	GSTK1_MOUSE	Glutathione S-transferase kappa 1 (EC 2.5.1.18) (GST 13-13) (GST class-kappa) (GSTK1-1) (mGSTK1) (Glutathione S-transferase subunit 13)
Q9DCX2	ATP5H_MOUSE	ATP synthase subunit d, mitochondrial (ATPase subunit d) (ATP synthase peripheral stalk subunit d)
Q9EP53	TSC1_MOUSE	Hamartin (Tuberous sclerosis 1 protein homolog)
Q9EPK6	SIL1_MOUSE	Nucleotide exchange factor SIL1
Q9EQ46	V1R40_MOUSE	Vomeronasal type-1 receptor 40 (Vomeronasal type-1 receptor A11) (Vomeronasal type-1 receptor B7)
Q9EQG7	ENPP5_MOUSE	Ectonucleotide pyrophosphatase/phosphodiesterase family member 5 (E-NPP 5) (NPP-5) (EC 3.1.-.-)
Q9EQQ2	YIPF5_MOUSE	Protein YIPF5 (YIP1 family member 5) (YPT-interacting protein 1 A)
Q9ER00	STX12_MOUSE	Syntaxin-12
Q9ER60	SCN4A_MOUSE	Sodium channel protein type 4 subunit alpha (Sodium channel protein skeletal muscle subunit alpha) (Sodium channel protein type IV subunit alpha) (Voltage-gated sodium channel subunit alpha Nav1.4)
Q9ER75	IRX6_MOUSE	Iroquois-class homeodomain protein IRX-6 (Homeodomain protein IRXB3) (Iroquois homeobox protein 6)
Q9ERH8	S28A3_MOUSE	Solute carrier family 28 member 3 (Concentrative Na(+)-nucleoside cotransporter 3) (CNT 3) (mCNT3)
Q9ERI2	RB27A_MOUSE	Ras-related protein Rab-27A (EC 3.6.5.2)
Q9ERL7	GMFG_MOUSE	Glia maturation factor gamma (GMF-gamma)
Q9ERS5	PKHA2_MOUSE	Pleckstrin homology domain-containing family A member 2 (PH domain-containing family A member 2) (PH domain-containing adaptor PHAD47) (Tandem PH domain-containing protein 2) (TAPP-2)

Q9ES28	ARHG7_MOUSE	Rho guanine nucleotide exchange factor 7 (Beta-Pix) (PAK-interacting exchange factor beta) (p85SPR)
Q9ES63	UBP29_MOUSE	Ubiquitin carboxyl-terminal hydrolase 29 (EC 3.4.19.12) (Deubiquitinating enzyme 29) (Ubiquitin thioesterase 29) (Ubiquitin-specific-processing protease 29)
Q9ES81	POPD3_MOUSE	Popeye domain-containing protein 3 (Popeye protein 3)
Q9ESK3	CAN10_MOUSE	Calpain-10 (EC 3.4.22.-) (Calcium-activated neutral proteinase 10) (CANP 10)
Q9ESL0	SCO2B_MOUSE	Succinyl-CoA:3-ketoacid coenzyme A transferase 2B, mitochondrial (EC 2.8.3.5) (3-oxoacid CoA-transferase 2B) (Testis-specific succinyl-CoA:3-oxoacid CoA-transferase 2) (SCOT-t2)
Q9ESN9	JIP3_MOUSE	C-Jun-amino-terminal kinase-interacting protein 3 (JIP-3) (JNK-interacting protein 3) (JNK MAP kinase scaffold protein 3) (JNK/SAPK-associated protein 1) (JSAP1) (Mitogen-activated protein kinase 8-interacting protein 3) (Sunday driver 2)
Q9ESU6	BRD4_MOUSE	Bromodomain-containing protein 4 (Mitotic chromosome-associated protein) (MCAP)
Q9JHZ2	ANKH_MOUSE	Progressive ankylosis protein (Fn54 protein)
Q9JI78	NGLY1_MOUSE	Peptide-N(4)-(N-acetyl-beta-glucosaminyl)asparagine amidase (PNGase) (mPNGase) (EC 3.5.1.52) (N-glycanase 1) (Peptide:N-glycanase)
Q9JII5	DAZP1_MOUSE	DAZ-associated protein 1 (Deleted in azoospermia-associated protein 1)
Q9JIK5	DDX21_MOUSE	Nucleolar RNA helicase 2 (EC 3.6.4.13) (DEAD box protein 21) (Gu-alpha) (Nucleolar RNA helicase Gu) (Nucleolar RNA helicase II) (RH II/Gu)
Q9JIL5	TULP4_MOUSE	Tubby-related protein 4 (Tubby superfamily protein) (Tubby-like protein 4)
Q9JIT0	LMBR1_MOUSE	Limb region 1 protein
Q9JJ59	ABCB9_MOUSE	ATP-binding cassette sub-family B member 9 (ATP-binding cassette transporter 9) (ABC transporter 9 protein) (mABCB9) (TAP-like protein) (TAPL)
Q9JJI8	RL38_MOUSE	60S ribosomal protein L38
Q9JJK2	LANC2_MOUSE	LanC-like protein 2 (Testis-specific adriamycin sensitivity protein)
Q9JJX6	P2RX4_MOUSE	P2X purinoceptor 4 (P2X4) (ATP receptor) (Purinergic receptor)

Q9JJZ4	UB2J1_MOUSE	Ubiquitin-conjugating enzyme E2 J1 (EC 2.3.2.23) (E2 ubiquitin-conjugating enzyme J1) (Non-canonical ubiquitin-conjugating enzyme 1) (NCUBE-1)
Q9JK38	GNA1_MOUSE	Glucosamine 6-phosphate N-acetyltransferase (EC 2.3.1.4) (Phosphoglucosamine acetylase) (Phosphoglucosamine transacetylase) (Protein EMeg32)
Q9JK45	KCNQ5_MOUSE	Potassium voltage-gated channel subfamily KQT member 5 (KQT-like 5) (Potassium channel subunit alpha KvLQT5) (Voltage-gated potassium channel subunit Kv7.5)
Q9JK81	MYG1_MOUSE	UPF0160 protein MYG1, mitochondrial (Protein Gamm1)
Q9JK97	KCNQ4_MOUSE	Potassium voltage-gated channel subfamily KQT member 4 (KQT-like 4) (Potassium channel subunit alpha KvLQT4) (Voltage-gated potassium channel subunit Kv7.4)
Q9JKU8	LMX1A_MOUSE	LIM homeobox transcription factor 1-alpha (LIM/homeobox protein 1.1) (LMX-1.1) (LIM/homeobox protein LMX1A)
Q9JKY0	CNOT9_MOUSE	CCR4-NOT transcription complex subunit 9 (Cell differentiation protein RQCD1 homolog) (Rcd-1) (EPO-induced protein FL10)
Q9JL99	CLC1B_MOUSE	C-type lectin domain family 1 member B (C-type lectin-like receptor 2) (CLEC-2)
Q9JLI6	SCLY_MOUSE	Selenocysteine lyase (mSCL) (EC 4.4.1.16)
Q9JLI8	SART3_MOUSE	Squamous cell carcinoma antigen recognized by T-cells 3 (SART-3) (mSART-3) (Tumor-rejection antigen SART3)
Q9JLV2	TP4AP_MOUSE	Short transient receptor potential channel 4-associated protein (Trp4-associated protein) (Trpc4-associated protein) (Protein TAP1) (Rabex-5/Rin2-interacting protein) (TNF-receptor ubiquitous scaffolding/signaling protein) (Protein TRUSS)
Q9JM90	STAP1_MOUSE	Signal-transducing adaptor protein 1 (STAP-1) (Stem cell adaptor protein 1)
Q9JM99	PRG4_MOUSE	Proteoglycan 4 (Lubricin) (Megakaryocyte-stimulating factor) (Superficial zone proteoglycan) [Cleaved into: Proteoglycan 4 C-terminal part]
Q9JMC8	E41LB_MOUSE	Band 4.1-like protein 4B (Protein EHM2)
Q9JME7	TPC2L_MOUSE	Trafficking protein particle complex subunit 2-like protein
Q9QUK4	BIR1B_MOUSE	Baculoviral IAP repeat-containing protein 1b (Neuronal apoptosis inhibitory protein 2)

Q9QXD6	F16P1_MOUSE	Fructose-1,6-bisphosphatase 1 (FBPase 1) (EC 3.1.3.11) (D-fructose-1,6-bisphosphate 1-phosphohydrolase 1) (Fructose-1,6-bisphosphatase isozyme 3) (FBPase 3) (Liver FBPase)
Q9QXX4	CMC2_MOUSE	Calcium-binding mitochondrial carrier protein Aralar2 (Citrin) (Mitochondrial aspartate glutamate carrier 2) (Solute carrier family 25 member 13)
Q9QYA2	TOM40_MOUSE	Mitochondrial import receptor subunit TOM40 homolog (Mitochondrial outer membrane protein of 35 kDa) (MOM35) (Protein Haymaker) (Translocase of outer membrane 40 kDa subunit homolog)
Q9QYE3	BC11A_MOUSE	B-cell lymphoma/leukemia 11A (BCL-11A) (B-cell CLL/lymphoma 11A) (COUP-TF-interacting protein 1) (Ecotropic viral integration site 9 protein) (EVI-9)
Q9QYE9	PKHB1_MOUSE	Pleckstrin homology domain-containing family B member 1 (PH domain-containing family B member 1) (Evectin-1) (PH domain-containing protein in retina 1) (PHRET1) (Pleckstrin homology domain retinal protein 1)
Q9QYS2	GRM3_MOUSE	Metabotropic glutamate receptor 3 (mGluR3)
Q9QZ11	EXO1_MOUSE	Exonuclease 1 (mExo1) (EC 3.1.-.-) (Exonuclease I)
Q9QZ15	CLC4A_MOUSE	C-type lectin domain family 4 member A (C-type lectin superfamily member 6) (Dendritic cell immunoreceptor) (CD antigen CD367)
Q9QZ39	SIA7A_MOUSE	Alpha-N-acetylgalactosaminide alpha-2,6-sialyltransferase 1 (EC 2.4.99.3) (GalNAc alpha-2,6-sialyltransferase I) (ST6GalNAc I) (ST6GalNAcI) (Sialyltransferase 7A) (SIAT7-A)
Q9QZ73	DCNL1_MOUSE	DCN1-like protein 1 (DCUN1 domain-containing protein 1) (Defective in cullin neddylation protein 1-like protein 1) (Testis-specific protein 3)
Q9QZI9	SERC3_MOUSE	Serine incorporator 3 (Axotomy-induced glyco/Golgi protein 1) (AIGP-1) (Axotomy-induced glycoprotein 1) (Membrane protein TMS-1) (Tumor differentially expressed protein 1)
Q9QZQ1	AFAD_MOUSE	Afadin (Afadin adherens junction formation factor) (Protein Af-6)
Q9QZR0	RNF25_MOUSE	E3 ubiquitin-protein ligase RNF25 (EC 2.3.2.27) (RING finger protein 25) (RING finger protein AO7) (RING-type E3 ubiquitin transferase RNF25)
Q9QZS3	NUMB_MOUSE	Protein numb homolog (m-Nb) (m-Numb)
Q9R059	FHL3_MOUSE	Four and a half LIM domains protein 3 (FHL-3) (Skeletal muscle LIM-protein 2) (SLIM-2)

Q9R061	NUBP2_MOUSE	Cytosolic Fe-S cluster assembly factor NUBP2 (Nucleotide-binding protein 2) (NBP 2)
Q9R0B6	LAMC3_MOUSE	Laminin subunit gamma-3 (Laminin-12 subunit gamma) (Laminin-14 subunit gamma) (Laminin-15 subunit gamma)
Q9R0Q1	SYTL4_MOUSE	Synaptotagmin-like protein 4 (Exophilin-2) (Granuphilin)
Q9R0X4	ACOT9_MOUSE	Acyl-coenzyme A thioesterase 9, mitochondrial (Acyl-CoA thioesterase 9) (EC 3.1.2.-) (Acyl coenzyme A thioester hydrolase 2) (MTE-2) (Acyl-CoA thioester hydrolase 9) (Mitochondrial 48 kDa acyl-CoA thioester hydrolase 1) (Mt-ACT48.1) (Protein U8) (p48)
Q9R112	SQOR_MOUSE	Sulfide:quinone oxidoreductase, mitochondrial (SQOR) (EC 1.8.5.-) (Sulfide quinone oxidoreductase)
Q9R187	EDAR_MOUSE	Tumor necrosis factor receptor superfamily member EDAR (Anhidrotic ectodysplasin receptor 1) (Downless) (Ectodermal dysplasia receptor) (Ectodysplasin-A receptor)
Q9R1A8	COP1_MOUSE	E3 ubiquitin-protein ligase COP1 (EC 2.3.2.27) (Constitutive photomorphogenesis protein 1 homolog) (mCOP1) (RING finger and WD repeat domain protein 2) (RING-type E3 ubiquitin transferase RFWD2)
Q9R1X5	MRP5_MOUSE	Multidrug resistance-associated protein 5 (ATP-binding cassette sub-family C member 5) (Multi-specific organic anion transporter C) (MOAT-C) (SMRP)
Q9R226	KHDR3_MOUSE	KH domain-containing, RNA-binding, signal transduction-associated protein 3 (RNA-binding protein Etoile) (Sam68-like mammalian protein 2) (SLM-2)
Q9R229	BMP10_MOUSE	Bone morphogenetic protein 10 (BMP-10)
Q9WTK2	CDYL_MOUSE	Chromodomain Y-like protein (CDY-like) (Crotonyl-CoA hydratase) (EC 4.2.1.-) (Putative histone acetyltransferase Cdyl) (EC 2.3.1.48)
Q9WTW3	KCNE4_MOUSE	Potassium voltage-gated channel subfamily E member 4 (MinK-related peptide 3) (Minimum potassium ion channel-related peptide 3) (Potassium channel subunit beta MiRP3)
Q9WU56	TRUA_MOUSE	tRNA pseudouridine synthase A (EC 5.4.99.12) (tRNA pseudouridine(38-40) synthase) (tRNA pseudouridylate synthase I) (tRNA-uridine isomerase I)

Q9WUD1	CHIP_MOUSE	STIP1 homology and U box-containing protein 1 (EC 2.3.2.27) (Carboxy terminus of Hsp70-interacting protein) (E3 ubiquitin-protein ligase CHIP) (RING-type E3 ubiquitin transferase CHIP)
Q9WUH5	TRI10_MOUSE	Tripartite motif-containing protein 10 (Hematopoietic RING finger 1) (RING finger protein 9)
Q9WUL7	ARL3_MOUSE	ADP-ribosylation factor-like protein 3
Q9WVD4	CLCN5_MOUSE	H(+)/Cl(-) exchange transporter 5 (Chloride channel protein 5) (ClC-5) (Chloride transporter ClC-5)
Q9WVD5	ORNT1_MOUSE	Mitochondrial ornithine transporter 1 (Solute carrier family 25 member 15)
Q9WVJ3	CBPQ_MOUSE	Carboxypeptidase Q (EC 3.4.17.-) (Hematopoietic lineage switch 2) (Plasma glutamate carboxypeptidase)
Q9WVM3	APC7_MOUSE	Anaphase-promoting complex subunit 7 (APC7) (Cyclosome subunit 7) (Prediabetic NOD sera-reactive autoantigen)
Q9WVP1	AP1M2_MOUSE	AP-1 complex subunit mu-2 (AP-mu chain family member mu1B) (Adaptor protein complex AP-1 subunit mu-2) (Adaptor-related protein complex 1 subunit mu-2) (Clathrin assembly protein complex 1 mu-2 medium chain 2) (Golgi adaptor HA1/AP1 adaptin mu-2 subunit) (Mu-adaptin 2) (Mu1B-adaptin)
Q9WVP6	PAPOB_MOUSE	Poly(A) polymerase beta (PAP-beta) (EC 2.7.7.19) (Polynucleotide adenylyltransferase beta) (Testis-specific poly(A) polymerase)
Q9WVS4	MOK_MOUSE	MAPK/MAK/MRK overlapping kinase (EC 2.7.11.22) (MOK protein kinase) (Serine/threonine kinase 30)
Q9Z0H4	CELF2_MOUSE	CUGBP Elav-like family member 2 (CELF-2) (Bruno-like protein 3) (CUG triplet repeat RNA-binding protein 2) (CUG-BP2) (CUG-BP- and ETR-3-like factor 2) (ELAV-type RNA-binding protein 3) (ETR-3) (mETR-3) (Neuroblastoma apoptosis-related RNA-binding protein) (mNapor) (RNA-binding protein BRUNOL-3)
Q9Z0M9	I18BP_MOUSE	Interleukin-18-binding protein (IL-18BP) (Interferon gamma-inducing factor-binding protein)
Q9Z0S9	PRAF1_MOUSE	Prenylated Rab acceptor protein 1 (PRA1 family protein 1) (Prenylin)
Q9Z0X4	PDE3A_MOUSE	cGMP-inhibited 3',5'-cyclic phosphodiesterase A (EC 3.1.4.17) (Cyclic GMP-inhibited phosphodiesterase A) (CGI-PDE A)

Q9Z129	RECQ1_MOUSE	ATP-dependent DNA helicase Q1 (EC 3.6.4.12) (DNA-dependent ATPase Q1) (RecQ protein-like 1)
Q9Z1S3	GRP1_MOUSE	RAS guanyl-releasing protein 1 (Calcium and DAG-regulated guanine nucleotide exchange factor II) (CalDAG-GEFII) (Ras guanyl-releasing protein)
Q9Z1X9	CDC45_MOUSE	Cell division control protein 45 homolog (PORC-PI-1)
Q9Z1Z0	USO1_MOUSE	General vesicular transport factor p115 (Protein USO1 homolog) (Transcytosis-associated protein) (TAP) (Vesicle-docking protein)
Q9Z2D7	MBD4_MOUSE	Methyl-CpG-binding domain protein 4 (EC 3.2.2.-) (Methyl-CpG-binding protein MBD4) (Mismatch-specific DNA N-glycosylase)
Q9Z2I0	LETM1_MOUSE	Mitochondrial proton/calcium exchanger protein (Leucine zipper-EF-hand-containing transmembrane protein 1)
Q9Z2L6	MINP1_MOUSE	Multiple inositol polyphosphate phosphatase 1 (EC 3.1.3.62) (2,3-bisphosphoglycerate 3-phosphatase) (2,3-BPG phosphatase) (EC 3.1.3.80) (Inositol (1,3,4,5)-tetrakisphosphate 3-phosphatase) (Ins(1,3,4,5)P(4) 3-phosphatase)
Q9Z304	MYCS_MOUSE	Protein S-Myc
Q9Z351	KCNQ2_MOUSE	Potassium voltage-gated channel subfamily KQT member 2 (KQT-like 2) (Potassium channel subunit alpha KvLQT2) (Voltage-gated potassium channel subunit Kv7.2)

Table 2. Proteins unique to iDCs EXO.

Accession	Entry name	Protein names
A1EGX6	FSCB_MOUSE	Fibrous sheath CABYR-binding protein (PKA-phosphorylated calcium and CABYR-binding protein)
A2AG50	MA7D2_MOUSE	MAP7 domain-containing protein 2
A2AKM2	HACD4_MOUSE	Very-long-chain (3R)-3-hydroxyacyl-CoA dehydratase 4 (EC 4.2.1.134) (3-hydroxyacyl-CoA dehydratase 4) (HACD4) (Protein-tyrosine phosphatase-like A domain-containing protein 2)
A2AQH4	BCORL_MOUSE	BCL-6 corepressor-like protein 1 (BCoR-L1) (BCoR-like protein 1)
A2ARJ3	TM236_MOUSE	Transmembrane protein 236
A2AV25	FBCD1_MOUSE	Fibrinogen C domain-containing protein 1
A2AVZ9	S43A3_MOUSE	Solute carrier family 43 member 3 (Embryonic epithelia gene 1 protein)
A2CG49	KALRN_MOUSE	Kalirin (EC 2.7.11.1) (Protein Duo) (Serine/threonine-protein kinase with Dbl- and pleckstrin homology domain)
A3KFU9	DISP3_MOUSE	Protein dispatched homolog 3 (Patched domain-containing protein 2) (RND-type protein RNDEu-2)
A3KGV1	ODFP2_MOUSE	Outer dense fiber protein 2 (84 kDa outer dense fiber protein) (Cenexin) (Outer dense fiber of sperm tails protein 2)
A4Q9F4	TTL11_MOUSE	Tubulin polyglutamylase TTLL11 (EC 6.-.-.-) (Tubulin--tyrosine ligase-like protein 11)
A6H639	DRC5_MOUSE	Dynein regulatory complex subunit 5 (T-complex-associated testis-expressed protein 1) (Tcte-1)
A8R0V4	ESP22_MOUSE	Exocrine gland-secreted peptide 22
B9EKI3	TMF1_MOUSE	TATA element modulatory factor (TMF) (Androgen receptor coactivator 160 kDa protein) (Androgen receptor-associated protein of 160 kDa)
C3VPR6	NLRC5_MOUSE	Protein NLRC5
D3YX43	VSI10_MOUSE	V-set and immunoglobulin domain-containing protein 10
D3Z4R1	HFM1_MOUSE	Probable ATP-dependent DNA helicase HFM1 (EC 3.6.4.12)
D3Z6S9	ERIP6_MOUSE	Glutamate-rich protein 6 (Protein FAM194A)
E2JF22	PIEZ1_MOUSE	Piezo-type mechanosensitive ion channel component 1 (Protein FAM38A)
E5FYH1	TOPZ1_MOUSE	Protein TOPAZ1 (Testis- and ovary-specific PAZ domain-containing protein 1)
E9PV87	TALD3_MOUSE	Protein TALPID3

E9Q557	DESP_MOUSE	Desmoplakin (DP)
E9Q612	PTPRO_MOUSE	Receptor-type tyrosine-protein phosphatase O (R-PTP-O) (EC 3.1.3.48) (Glomerular epithelial protein 1) (Protein tyrosine phosphatase U2) (PTP-U2) (PTPase U2)
G3X9R7	RN148_MOUSE	RING finger protein 148 (Goliath-related E3 ubiquitin-protein ligase 3)
O08705	NTCP_MOUSE	Sodium/bile acid cotransporter (Na(+)/bile acid cotransporter) (Na(+)/taurocholate transport protein) (Sodium/taurocholate cotransporting polypeptide) (Solute carrier family 10 member 1)
O08746	MATN2_MOUSE	Matrilin-2
O09047	C3AR_MOUSE	C3a anaphylatoxin chemotactic receptor (C3AR) (C3a-R) (Complement component 3a receptor 1)
O35136	NCAM2_MOUSE	Neural cell adhesion molecule 2 (N-CAM-2) (NCAM-2) (Neural cell adhesion molecule RB-8) (R4B12)
O35144	TERF2_MOUSE	Telomeric repeat-binding factor 2 (TTAGGG repeat-binding factor 2) (Telomeric DNA-binding protein)
O35256	PR4A1_MOUSE	Prolactin-4A1 (Placental prolactin-like protein A) (PLP-A) (PRL-like protein A)
O35348	COLQ_MOUSE	Acetylcholinesterase collagenic tail peptide (AChE Q subunit) (Acetylcholinesterase-associated collagen)
O35464	SEM6A_MOUSE	Semaphorin-6A (Semaphorin Q) (Sema Q) (Semaphorin VIA) (Sema VIA) (Semaphorin-6A-1) (SEMA6A-1)
O35621	PMM1_MOUSE	Phosphomannomutase 1 (PMM 1) (EC 5.4.2.8)
O35709	ENC1_MOUSE	Ectoderm-neural cortex protein 1 (ENC-1)
O35710	NOCT_MOUSE	Nocturnin (EC 3.1.3.-) (Carbon catabolite repression 4-like protein)
O35740	CITE2_MOUSE	Cbp/p300-interacting transactivator 2 (MSG-related protein 1) (MRG-1) (P35srj)
O54692	ZW10_MOUSE	Centromere/kinetochore protein zw10 homolog
O54714	PIAS3_MOUSE	E3 SUMO-protein ligase PIAS3 (EC 2.3.2.-) (E3 SUMO-protein transferase PIAS3) (Protein inhibitor of activated STAT protein 3)
O54754	AOXA_MOUSE	Aldehyde oxidase 1 (EC 1.2.3.1) (Azaheterocycle hydroxylase 1) (EC 1.17.3.-) (Retinal oxidase)
O54818	TPD53_MOUSE	Tumor protein D53 (mD53) (Tumor protein D52-like 1)
O54885	TYOBP_MOUSE	TYRO protein tyrosine kinase-binding protein (DNAX-activation protein 12) (Killer-activating

		receptor-associated protein) (KAR-associated protein)
O54967	ACK1_MOUSE	Activated CDC42 kinase 1 (ACK-1) (EC 2.7.10.2) (EC 2.7.11.1) (Non-receptor protein tyrosine kinase Ack) (Tyrosine kinase non-receptor protein 2)
O55017	CAC1B_MOUSE	Voltage-dependent N-type calcium channel subunit alpha-1B (Brain calcium channel III) (BIII) (Calcium channel, L type, alpha-1 polypeptide isoform 5) (Voltage-gated calcium channel subunit alpha Cav2.2)
O55070	DNSL3_MOUSE	Deoxyribonuclease gamma (DNase gamma) (EC 3.1.21.-) (DNase I homolog protein DHP2) (Deoxyribonuclease I-like 3) (DNase I-like 3) (Liver and spleen DNase) (LS-DNase) (LSD)
O55071	CP2BJ_MOUSE	Cytochrome P450 2B19 (EC 1.14.14.1) (CYPIIB19)
O55082	PTN20_MOUSE	Tyrosine-protein phosphatase non-receptor type 20 (EC 3.1.3.48) (Testis-specific tyrosine phosphatase)
O55112	AFF2_MOUSE	AF4/FMR2 family member 2 (FMR2P) (Fragile X mental retardation protein 2 homolog) (Protein FMR-2) (Protein Ox19)
O70579	PM34_MOUSE	Peroxisomal membrane protein PMP34 (34 kDa peroxisomal membrane protein) (Solute carrier family 25 member 17)
O88319	NTR1_MOUSE	Neurotensin receptor type 1 (NT-R-1) (NTR1)
O88329	MYO1A_MOUSE	Unconventional myosin-Ia (Brush border myosin I) (BBM-I) (BBMI) (Myosin I heavy chain) (MIHC)
O88425	NDK6_MOUSE	Nucleoside diphosphate kinase 6 (NDK 6) (NDP kinase 6) (EC 2.7.4.6) (nm23-M6)
O88513	GEMI_MOUSE	Geminin
O88520	SHOC2_MOUSE	Leucine-rich repeat protein SHOC-2 (Protein soc-2 homolog) (Protein sur-8 homolog)
O88627	S28A2_MOUSE	Sodium/nucleoside cotransporter 2 (Concentrative nucleoside transporter 2) (CNT 2) (Na(+)/nucleoside cotransporter 2) (Sodium-coupled nucleoside transporter 2) (Sodium/purine nucleoside cotransporter) (SPNT) (Solute carrier family 28 member 2)
O88796	RPP30_MOUSE	Ribonuclease P protein subunit p30 (RNaseP protein p30) (EC 3.1.26.5) (RNase P subunit 2)
O88839	ADA15_MOUSE	Disintegrin and metalloproteinase domain-containing protein 15 (ADAM 15) (EC 3.4.24.-) (AD56) (Metalloprotease RGD disintegrin protein)

		(Metalloproteinase-like, disintegrin-like, and cysteine-rich protein 15) (MDC-15) (Metargidin)
O88842	FGD3_MOUSE	FYVE, RhoGEF and PH domain-containing protein 3
O88854	GALR2_MOUSE	Galanin receptor type 2 (GAL2-R) (GALR-2)
O88942	NFAC1_MOUSE	Nuclear factor of activated T-cells, cytoplasmic 1 (NF-ATc1) (NFATc1) (NFAT transcription complex cytosolic component) (NF-ATc) (NFATc)
O88947	FA10_MOUSE	Coagulation factor X (EC 3.4.21.6) (Stuart factor) [Cleaved into: Factor X light chain; Factor X heavy chain; Activated factor Xa heavy chain]
O88968	TCO2_MOUSE	Transcobalamin-2 (TC-2) (Transcobalamin II) (TC II) (TCII)
O88974	SETB1_MOUSE	Histone-lysine N-methyltransferase SETDB1 (EC 2.1.1.-) (ERG-associated protein with SET domain) (ESET) (SET domain bifurcated 1)
O89017	LGMN_MOUSE	Legumain (EC 3.4.22.34) (Asparaginyl endopeptidase) (Protease, cysteine 1)
O89103	C1QR1_MOUSE	Complement component C1q receptor (C1q/MBL/SPA receptor) (C1qR(p)) (C1qRp) (Cell surface antigen AA4) (Complement component 1 q subcomponent receptor 1) (Lymphocyte antigen 68) (Ly-68) (CD antigen CD93)
P01132	EGF_MOUSE	Pro-epidermal growth factor (EGF) [Cleaved into: Epidermal growth factor]
P01819	HVM43_MOUSE	Ig heavy chain V region MOPC 141
P01881	IGHD_MOUSE	Ig delta chain C region secreted form
P03888	NU1M_MOUSE	NADH-ubiquinone oxidoreductase chain 1 (EC 7.1.1.2) (NADH dehydrogenase subunit 1)
P04760	ACHG_MOUSE	Acetylcholine receptor subunit gamma
P04944	KV6AA_MOUSE	Ig kappa chain V-VI region NQ5-78.2.6
P04946	KV5AM_MOUSE	Ig kappa chain V-V region NQ5-89.4
P05524	FGF3_MOUSE	Fibroblast growth factor 3 (FGF-3) (Heparin-binding growth factor 3) (HBGF-3) (Proto-oncogene Int-2)
P06537	GCR_MOUSE	Glucocorticoid receptor (GR) (Nuclear receptor subfamily 3 group C member 1)
P06684	CO5_MOUSE	Complement C5 (Hemolytic complement) [Cleaved into: Complement C5 beta chain; Complement C5 alpha chain; C5a anaphylatoxin; Complement C5 alpha' chain]
P06797	CATL1_MOUSE	Cathepsin L1 (EC 3.4.22.15) (Cathepsin L) (Major excreted protein) (MEP) (p39 cysteine proteinase)

		[Cleaved into: Cathepsin L1 heavy chain; Cathepsin L1 light chain]
P07141	CSF1_MOUSE	Macrophage colony-stimulating factor 1 (CSF-1) (MCSF) [Cleaved into: Processed macrophage colony-stimulating factor 1]
P08905	LYZ2_MOUSE	Lysozyme C-2 (EC 3.2.1.17) (1,4-beta-N-acetylmuramidase C) (Lysozyme C type M)
P0C192	LRC4B_MOUSE	Leucine-rich repeat-containing protein 4B (Netrin-G3 ligand) (NGL-3)
P0C1Q2	PDE11_MOUSE	Dual 3',5'-cyclic-AMP and -GMP phosphodiesterase 11A (EC 3.1.4.35) (EC 3.1.4.53) (cAMP and cGMP phosphodiesterase 11A)
P0CB42	ALKB1_MOUSE	Nucleic acid dioxygenase ALKBH1 (EC 1.14.11.-) (Alkylated DNA repair protein alkB homolog 1) (Alpha-ketoglutarate-dependent dioxygenase ABH1) (DNA 6mA demethylase) (DNA N6-methyl adenine demethylase ALKBH1) (EC 1.14.11.51) (DNA lyase ABH1) (EC 4.2.99.18) (DNA oxidative demethylase ALKBH1) (EC 1.14.11.33) (mRNA N(3)-methylcytidine demethylase) (EC 1.14.11.-) (tRNA N1-methyl adenine demethylase) (EC 1.14.11.-)
P0CC03	ST6B1_MOUSE	Sulfotransferase 6B1 (ST6B1) (Thyroxine sulfotransferase) (EC 2.8.2.n2)
P0CG14	DERPC_MOUSE	Decreased expression in renal and prostate cancer protein
P0DJF2	PT117_MOUSE	Protein PET117 homolog, mitochondrial
P10761	ZP3_MOUSE	Zona pellucida sperm-binding protein 3 (Sperm receptor) (Zona pellucida glycoprotein 3) (Zp-3) (Zona pellucida protein C) [Cleaved into: Processed zona pellucida sperm-binding protein 3]
P10923	OSTP_MOUSE	Osteopontin (2AR) (Bone sialoprotein 1) (Calcium oxalate crystal growth inhibitor protein) (Early T-lymphocyte activation 1 protein) (Minopontin) (Secreted phosphoprotein 1) (SPP-1)
P11672	NGAL_MOUSE	Neutrophil gelatinase-associated lipocalin (NGAL) (Lipocalin-2) (Oncogene 24p3) (24p3) (SV-40-induced 24p3 protein) (Siderocalin LCN2) (p25)
P11680	PROP_MOUSE	Properdin (Complement factor P)
P12023	A4_MOUSE	Amyloid-beta A4 protein (ABPP) (APP) (Alzheimer disease amyloid A4 protein homolog) (Amyloid precursor protein) (Amyloid-beta precursor protein) (Amyloidogenic glycoprotein) (AG) [Cleaved into: N-APP; Soluble APP-alpha (S-APP-alpha); Soluble APP-beta (S-APP-beta);

		C99 (APP-C99) (Beta-secretase C-terminal fragment) (Beta-CTF); Amyloid-beta protein 42 (Abeta42) (Beta-APP42); Amyloid-beta protein 40 (Abeta40) (Beta-APP40); C83 (Alpha-secretase C-terminal fragment) (Alpha-CTF); P3(42); P3(40); C80; Gamma-secretase C-terminal fragment 59 (APP-C59) (Amyloid intracellular domain 59) (AID(59)) (Gamma-CTF(59)); Gamma-secretase C-terminal fragment 57 (APP-C57) (Amyloid intracellular domain 57) (AID(57)) (Gamma-CTF(57)); Gamma-secretase C-terminal fragment 50 (Amyloid intracellular domain 50) (AID(50)) (Gamma-CTF(50)); C31]
P12960	CNTN1_MOUSE	Contactin-1 (Neural cell surface protein F3)
P13542	MYH8_MOUSE	Myosin-8 (Myosin heavy chain 8) (Myosin heavy chain, skeletal muscle, perinatal) (MyHC-perinatal)
P16125	LDHB_MOUSE	L-lactate dehydrogenase B chain (LDH-B) (EC 1.1.1.27) (LDH heart subunit) (LDH-H)
P17892	LIPR2_MOUSE	Pancreatic lipase-related protein 2 (PL-RP2) (EC 3.1.1.26) (EC 3.1.1.3) (Cytotoxic T-lymphocyte lipase) (Galactolipase)
P18052	PTPRA_MOUSE	Receptor-type tyrosine-protein phosphatase alpha (Protein-tyrosine phosphatase alpha) (R-PTP-alpha) (EC 3.1.3.48) (LCA-related phosphatase) (PTPTY-28)
P18826	KPB1_MOUSE	Phosphorylase b kinase regulatory subunit alpha, skeletal muscle isoform (Phosphorylase kinase alpha M subunit)
P21129	P3_MOUSE	P3 protein (Solute carrier family 10 member 3)
P21183	IL5RA_MOUSE	Interleukin-5 receptor subunit alpha (IL-5 receptor subunit alpha) (IL-5R subunit alpha) (IL-5R-alpha) (IL-5RA) (CD antigen CD125)
P21619	LMNB2_MOUSE	Lamin-B2
P23738	DCHS_MOUSE	Histidine decarboxylase (HDC) (EC 4.1.1.22)
P23780	BGAL_MOUSE	Beta-galactosidase (EC 3.2.1.23) (Acid beta-galactosidase) (Lactase)
P24456	CP2DA_MOUSE	Cytochrome P450 2D10 (EC 1.14.14.1) (CYPIID10) (Cytochrome P450-16-alpha) (Cytochrome P450CB) (Testosterone 16-alpha hydroxylase)
P24638	PPAL_MOUSE	Lysosomal acid phosphatase (LAP) (EC 3.1.3.2)
P25206	MCM3_MOUSE	DNA replication licensing factor MCM3 (EC 3.6.4.12) (DNA polymerase alpha holoenzyme-associated protein P1) (P1-MCM3)

P26151	FCGR1_MOUSE	High affinity immunoglobulin gamma Fc receptor I (IgG Fc receptor I) (Fc-gamma RI) (FcRI) (CD antigen CD64)
P28659	CELF1_MOUSE	CUGBP Elav-like family member 1 (CELF-1) (50 kDa nuclear polyadenylated RNA-binding protein) (Brain protein F41) (Bruno-like protein 2) (CUG triplet repeat RNA-binding protein 1) (CUG-BP1) (CUG-BP- and ETR-3-like factor 1) (Deadenylation factor CUG-BP) (Deadenylation factor EDEN-BP) (Embryo deadenylation element-binding protein homolog) (EDEN-BP homolog) (RNA-binding protein BRUNOL-2)
P28705	RXRG_MOUSE	Retinoic acid receptor RXR-gamma (Nuclear receptor subfamily 2 group B member 3) (Retinoid X receptor gamma)
P30873	SSR1_MOUSE	Somatostatin receptor type 1 (SS-1-R) (SS1-R) (SS1R) (SRIF-2)
P31428	DPEP1_MOUSE	Dipeptidase 1 (EC 3.4.13.19) (Membrane-bound dipeptidase 1) (MBD-1) (Microsomal dipeptidase) (Renal dipeptidase)
P31649	S6A13_MOUSE	Sodium- and chloride-dependent GABA transporter 2 (GAT-2) (Sodium- and chloride-dependent GABA transporter 3) (GAT-3) (Solute carrier family 6 member 13)
P31695	NOTC4_MOUSE	Neurogenic locus notch homolog protein 4 (Notch 4) [Cleaved into: Transforming protein Int-3; Notch 4 extracellular truncation; Notch 4 intracellular domain]
P32020	NLTP_MOUSE	Non-specific lipid-transfer protein (NSL-TP) (EC 2.3.1.176) (Propanoyl-CoA C-acyltransferase) (SCP-chi) (SCPX) (Sterol carrier protein 2) (SCP-2) (Sterol carrier protein X) (SCP-X)
P32211	ACM4_MOUSE	Muscarinic acetylcholine receptor M4 (Mm4 mAChR)
P32972	TNFL8_MOUSE	Tumor necrosis factor ligand superfamily member 8 (CD30 ligand) (CD30-L) (CD antigen CD153)
P33033	MC3R_MOUSE	Melanocortin receptor 3 (MC3-R)
P33434	MMP2_MOUSE	72 kDa type IV collagenase (EC 3.4.24.24) (72 kDa gelatinase) (Gelatinase A) (Matrix metalloproteinase-2) (MMP-2) [Cleaved into: PEX]
P34057	RECO_MOUSE	Recoverin (23 kDa photoreceptor cell-specific protein) (Cancer-associated retinopathy protein) (Protein CAR)
P35175	CYT1_MOUSE	Stefin-1

P35438	NMDZ1_MOUSE	Glutamate receptor ionotropic, NMDA 1 (GluN1) (Glutamate [NMDA] receptor subunit zeta-1) (N-methyl-D-aspartate receptor subunit NR1) (NMD-R1)
P35710	SOX5_MOUSE	Transcription factor SOX-5
P35917	VGFR3_MOUSE	Vascular endothelial growth factor receptor 3 (VEGFR-3) (EC 2.7.10.1) (Fms-like tyrosine kinase 4) (FLT-4) (Tyrosine-protein kinase receptor FLT4)
P35951	LDLR_MOUSE	Low-density lipoprotein receptor (LDL receptor)
P36423	THAS_MOUSE	Thromboxane-A synthase (TS) (TXA synthase) (TXS) (EC 5.3.99.5) (Cytochrome P450 5A1)
P39447	ZO1_MOUSE	Tight junction protein ZO-1 (Tight junction protein 1) (Zona occludens protein 1) (Zonula occludens protein 1)
P39654	LOX15_MOUSE	Arachidonate 15-lipoxygenase (15-LOX) (EC 1.13.11.33) (12/15-lipoxygenase) (12/15-LO) (Arachidonate 12-lipoxygenase, leukocyte-type) (12-LOX) (L-12LO) (EC 1.13.11.31) (Arachidonate omega-6 lipoxygenase)
P43120	HHEX_MOUSE	Hematopoietically-expressed homeobox protein Hhex (Homeobox protein HEX) (mHex) (Homeobox protein PRH)
P43267	SOX15_MOUSE	Protein SOX-15
P47802	MTX1_MOUSE	Metaxin-1 (Mitochondrial outer membrane import complex protein 1)
P48967	MPIP3_MOUSE	M-phase inducer phosphatase 3 (EC 3.1.3.48) (Dual specificity phosphatase Cdc25C)
P49025	CTRO_MOUSE	Citron Rho-interacting kinase (CRIK) (EC 2.7.11.1) (Rho-interacting, serine/threonine-protein kinase 21)
P49817	CAV1_MOUSE	Caveolin-1
P50220	NKX21_MOUSE	Homeobox protein Nkx-2.1 (Thyroid nuclear factor 1) (Thyroid transcription factor 1) (TTF-1) (Thyroid-specific enhancer-binding protein) (T/EBP)
P51655	GPC4_MOUSE	Glypican-4 (K-glypican) [Cleaved into: Secreted glypican-4]
P51944	CCNF_MOUSE	Cyclin-F
P53349	M3K1_MOUSE	Mitogen-activated protein kinase kinase kinase 1 (EC 2.7.11.25) (MAPK/ERK kinase kinase 1) (MEK kinase 1) (MEKK 1)
P54254	ATX1_MOUSE	Ataxin-1 (Spinocerebellar ataxia type 1 protein homolog)

P55096	ABCD3_MOUSE	ATP-binding cassette sub-family D member 3 (68 kDa peroxisomal membrane protein) (PMP68) (70 kDa peroxisomal membrane protein) (PMP70)
P55937	GOGA3_MOUSE	Golgin subfamily A member 3 (Golgin-160) (Male-enhanced antigen 2) (MEA-2)
P56203	CATW_MOUSE	Cathepsin W (EC 3.4.22.-) (Lymphopain)
P58058	NADK_MOUSE	NAD kinase (EC 2.7.1.23) (Poly(P)/ATP NAD kinase)
P58682	TLR8_MOUSE	Toll-like receptor 8 (CD antigen CD288)
P59438	HPS5_MOUSE	Hermansky-Pudlak syndrome 5 protein homolog (Ruby-eye protein 2) (Ru2)
P60521	GBRL2_MOUSE	Gamma-aminobutyric acid receptor-associated protein-like 2 (GABA(A) receptor-associated protein-like 2) (Golgi-associated ATPase enhancer of 16 kDa) (GATE-16)
P61967	AP1S1_MOUSE	AP-1 complex subunit sigma-1A (Adaptor protein complex AP-1 subunit sigma-1A) (Adaptor-related protein complex 1 subunit sigma-1A) (Clathrin assembly protein complex 1 sigma-1A small chain) (Clathrin coat assembly protein AP19) (Golgi adaptor HA1/AP1 adaptin sigma-1A subunit) (HA1 19 kDa subunit) (Sigma 1a subunit of AP-1 clathrin) (Sigma-adaptin 1A) (Sigma1A-adaptin)
P62812	GBRA1_MOUSE	Gamma-aminobutyric acid receptor subunit alpha-1 (GABA(A) receptor subunit alpha-1)
P63013	PRRX1_MOUSE	Paired mesoderm homeobox protein 1 (Homeobox protein K-2) (Homeobox protein mHox) (Paired-related homeobox protein 1) (PRX-1)
P70224	GIMA1_MOUSE	GTPase IMAP family member 1 (Immune-associated protein 38) (IAP38) (Immunity-associated protein 1)
P70297	STAM1_MOUSE	Signal transducing adapter molecule 1 (STAM-1)
P70389	ALS_MOUSE	Insulin-like growth factor-binding protein complex acid labile subunit (ALS)
P70671	IRF3_MOUSE	Interferon regulatory factor 3 (IRF-3)
P82350	SGCA_MOUSE	Alpha-sarcoglycan (Alpha-SG) (50 kDa dystrophin-associated glycoprotein) (50DAG) (Adhalin)
P86044	ANO9_MOUSE	Anoctamin-9 (Transmembrane protein 16J)
P86045	TM207_MOUSE	Transmembrane protein 207
P97311	MCM6_MOUSE	DNA replication licensing factor MCM6 (EC 3.6.4.12) (Mis5 homolog)
P97333	NRP1_MOUSE	Neuropilin-1 (A5 protein) (CD antigen CD304)
P97376	FRG1_MOUSE	Protein FRG1 (FSHD region gene 1 protein)

P97400	LEG10_MOUSE	Galectin-10 (Gal-10) (Charcot-Leyden crystal protein homolog) (CLC) (Fragment)
P97489	GATA5_MOUSE	Transcription factor GATA-5 (GATA-binding factor 5)
P97490	ADCY8_MOUSE	Adenylate cyclase type 8 (EC 4.6.1.1) (ATP pyrophosphate-lyase 8) (Adenylate cyclase type VIII) (Adenylyl cyclase 8) (Ca(2+)/calmodulin-activated adenylyl cyclase)
P97737	GDF10_MOUSE	Growth/differentiation factor 10 (GDF-10) (Bone morphogenetic protein 3B) (BMP-3B)
P97813	PLD2_MOUSE	Phospholipase D2 (PLD 2) (mPLD2) (EC 3.1.4.4) (Choline phosphatase 2) (PLD1C) (Phosphatidylcholine-hydrolyzing phospholipase D2)
P97819	PLPL9_MOUSE	85/88 kDa calcium-independent phospholipase A2 (CaI-PLA2) (EC 3.1.1.4) (Group VI phospholipase A2) (GVI PLA2) (Intracellular membrane-associated calcium-independent phospholipase A2 beta) (iPLA2-beta) (Patatin-like phospholipase domain-containing protein 9) (PNPLA9)
P98156	VLDLR_MOUSE	Very low-density lipoprotein receptor (VLDL receptor) (VLDL-R)
Q00422	GABPA_MOUSE	GA-binding protein alpha chain (GABP subunit alpha)
Q00560	IL6RB_MOUSE	Interleukin-6 receptor subunit beta (IL-6 receptor subunit beta) (IL-6R subunit beta) (IL-6R-beta) (IL-6RB) (Interleukin-6 signal transducer) (Membrane glycoprotein 130) (gp130) (Oncostatin-M receptor subunit alpha) (CD antigen CD130)
Q00623	APOA1_MOUSE	Apolipoprotein A-I (Apo-AI) (ApoA-I) (Apolipoprotein A1) [Cleaved into: Proapolipoprotein A-I (ProapoA-I); Truncated apolipoprotein A-I]
Q00977	CXB2_MOUSE	Gap junction beta-2 protein (Connexin-26) (Cx26)
Q01339	APOH_MOUSE	Beta-2-glycoprotein 1 (APC inhibitor) (Activated protein C-binding protein) (Apolipoprotein H) (Apo-H) (Beta-2-glycoprotein I) (B2GPI) (Beta(2)GPI)
Q02819	NUCB1_MOUSE	Nucleobindin-1 (CALNUC)
Q03145	EPHA2_MOUSE	Ephrin type-A receptor 2 (EC 2.7.10.1) (Epithelial cell kinase) (Tyrosine-protein kinase receptor ECK) (Tyrosine-protein kinase receptor MPK-5) (Tyrosine-protein kinase receptor SEK-2)

Q03719	KCND1_MOUSE	Potassium voltage-gated channel subfamily D member 1 (Voltage-gated potassium channel subunit Kv4.1) (mShal)
Q04592	PCSK5_MOUSE	Proprotein convertase subtilisin/kexin type 5 (EC 3.4.21.-) (Proprotein convertase 5) (PC5) (Proprotein convertase 6) (PC6) (Subtilisin-like proprotein convertase 6) (SPC6) (Subtilisin/kexin-like protease PC5)
Q05910	ADAM8_MOUSE	Disintegrin and metalloproteinase domain-containing protein 8 (ADAM 8) (EC 3.4.24.-) (Cell surface antigen MS2) (Macrophage cysteine-rich glycoprotein) (CD antigen CD156a)
Q06335	APLP2_MOUSE	Amyloid-like protein 2 (APLP-2) (CDEI box-binding protein) (CDEBP)
Q08775	RUNX2_MOUSE	Runt-related transcription factor 2 (Acute myeloid leukemia 3 protein) (Core-binding factor subunit alpha-1) (CBF-alpha-1) (Oncogene AML-3) (Osteoblast-specific transcription factor 2) (OSF-2) (Polyomavirus enhancer-binding protein 2 alpha A subunit) (PEA2-alpha A) (PEBP2-alpha A) (SL3-3 enhancer factor 1 alpha A subunit) (SL3/AKV core-binding factor alpha A subunit)
Q0GGX2	ZN541_MOUSE	Zinc finger protein 541 (Spermatogenic cell HDAC-interacting protein 1)
Q0HA38	TT21B_MOUSE	Tetratricopeptide repeat protein 21B (TPR repeat protein 21B) (Intraflagellar transport 139 homolog) (Tetratricopeptide repeat-containing hedgehog modulator 1)
Q0P543	GIPR_MOUSE	Gastric inhibitory polypeptide receptor (GIP-R) (Glucose-dependent insulinotropic polypeptide receptor)
Q0P5W1	VPS8_MOUSE	Vacuolar protein sorting-associated protein 8 homolog
Q0P5X5	ZBBX_MOUSE	Zinc finger B-box domain-containing protein 1
Q0QWG9	GRD2I_MOUSE	Delphilin (Glutamate receptor, ionotropic, delta 2-interacting protein 1)
Q0VB26	TEX26_MOUSE	Testis-expressed protein 26
Q0VET5	LMTD2_MOUSE	Lamin tail domain-containing protein 2
Q0VGT4	ZGRF1_MOUSE	Protein ZGRF1 (GRF-type zinc finger domain-containing protein 1)
Q14C59	TM11B_MOUSE	Transmembrane protease serine 11B-like protein (EC 3.4.21.-) (Airway trypsin-like protease 5) (Transmembrane protease serine 11B)
Q14DK4	GPAT2_MOUSE	Glycerol-3-phosphate acyltransferase 2, mitochondrial (GPAT-2) (EC 2.3.1.15) (xGPAT1)

Q2EG98	PK1L3_MOUSE	Polycystic kidney disease protein 1-like 3 (PC1-like 3 protein) (Polycystin-1L3)
Q2QI47	USH2A_MOUSE	Usherin (Usher syndrome type IIa protein homolog) (Usher syndrome type-2A protein homolog)
Q2TV84	TRPM1_MOUSE	Transient receptor potential cation channel subfamily M member 1 (Long transient receptor potential channel 1) (LTrpC1) (Melastatin-1)
Q30KP3	DFB20_MOUSE	Beta-defensin 20 (BD-20) (mBD-20) (Defensin, beta 20)
Q32KG4	RTL9_MOUSE	Retrotransposon Gag-like protein 9 (Retrotransposon gag domain-containing protein 1) (Sushi-XF2)
Q3TC72	FAHD2_MOUSE	Fumarylacetoacetate hydrolase domain-containing protein 2A (EC 3.-.-.-)
Q3TDX8	NB5R4_MOUSE	Cytochrome b5 reductase 4 (EC 1.6.2.2) (Flavohemoprotein b5/b5R) (b5+b5R) (N-terminal cytochrome b5 and cytochrome b5 oxidoreductase domain-containing protein) (cb5/cb5R)
Q3TIT8	SPX3_MOUSE	Sugar phosphate exchanger 3 (Solute carrier family 37 member 3)
Q3TNA1	XYLB_MOUSE	Xylulose kinase (Xylulokinase) (EC 2.7.1.17)
Q3TRJ4	K1C26_MOUSE	Keratin, type I cytoskeletal 26 (Cytokeratin-26) (CK-26) (Keratin-26) (K26) (Type I inner root sheath-specific keratin-K25irs2)
Q3TTY0	PLB1_MOUSE	Phospholipase B1, membrane-associated (Phospholipase B) (Phospholipase B/lipase) (PLB/LIP) [Includes: Phospholipase A2 (EC 3.1.1.4); Lysophospholipase (EC 3.1.1.5)]
Q3TUA9	SG196_MOUSE	Protein O-mannose kinase (POMK) (EC 2.7.1.183) (Protein kinase-like protein SgK196) (Sugen kinase 196)
Q3TX51	LRC28_MOUSE	Leucine-rich repeat-containing protein 28
Q3TXX4	VGLU1_MOUSE	Vesicular glutamate transporter 1 (VGluT1) (Brain-specific Na(+)-dependent inorganic phosphate cotransporter) (Solute carrier family 17 member 7)
Q3U284	TM231_MOUSE	Transmembrane protein 231
Q3U3D7	T131L_MOUSE	Transmembrane protein 131-like
Q3U487	HECD3_MOUSE	E3 ubiquitin-protein ligase HECTD3 (EC 2.3.2.26) (HECT domain-containing protein 3) (HECT-type E3 ubiquitin transferase HECTD3)
Q3UD82	PARP8_MOUSE	Protein mono-ADP-ribosyltransferase PARP8 (EC 2.4.2.-) (ADP-ribosyltransferase diphtheria toxin-

		like 16) (ARTD16) (Poly [ADP-ribose] polymerase 8) (PARP-8)
Q3UE37	UBE2Z_MOUSE	Ubiquitin-conjugating enzyme E2 Z (EC 2.3.2.23) (E2 ubiquitin-conjugating enzyme Z) (Uba6-specific E2 conjugating enzyme 1) (Use1) (Ubiquitin carrier protein Z) (Ubiquitin-protein ligase Z)
Q3UHD1	AGRB1_MOUSE	Adhesion G protein-coupled receptor B1 (Brain-specific angiogenesis inhibitor 1) [Cleaved into: Vasculostatin-120 (Vstat120); Vasculostatin-40 (Vstat-40)]
Q3ULZ2	FHDC1_MOUSE	FH2 domain-containing protein 1 (Inverted formin-1)
Q3UMG5	LRCH2_MOUSE	Leucine-rich repeat and calponin homology domain-containing protein 2
Q3UMU9	HDGR2_MOUSE	Hepatoma-derived growth factor-related protein 2 (HRP-2)
Q3UN16	GP162_MOUSE	Probable G-protein coupled receptor 162
Q3UPC7	K0825_MOUSE	Uncharacterized protein KIAA0825 homolog
Q3UR70	TGFA1_MOUSE	Transforming growth factor-beta receptor-associated protein 1 (TGF-beta receptor-associated protein 1) (TRAP-1) (TRAP1)
Q3URD3	SLMAP_MOUSE	Sarcolemmal membrane-associated protein (Sarcolemmal-associated protein)
Q3USQ7	SYN1L_MOUSE	Synapse differentiation-inducing gene protein 1-like (Capucin) (Caudate and putamen-enriched protein) (Dispanin subfamily C member 1) (DSPC1) (Synapse differentiation-induced protein 2) (Transmembrane protein 90A)
Q3UVX5	GRM5_MOUSE	Metabotropic glutamate receptor 5 (mGluR5)
Q3UVY1	GP149_MOUSE	Probable G-protein coupled receptor 149 (G-protein coupled receptor PGR10)
Q3UYR4	ESPNL_MOUSE	Espin-like protein
Q3UZD5	PRDM6_MOUSE	Putative histone-lysine N-methyltransferase PRDM6 (EC 2.1.1.-) (PR domain zinc finger protein 6) (PR domain-containing protein 6) (PR domain-containing protein in smooth muscle)
Q3V036	CCD27_MOUSE	Coiled-coil domain-containing protein 27
Q499E0	BRNP3_MOUSE	BMP/retinoic acid-inducible neural-specific protein 3
Q4ACU6	SHAN3_MOUSE	SH3 and multiple ankyrin repeat domains protein 3 (Shank3) (Proline-rich synapse-associated protein 2) (ProSAP2) (SPANK-2)
Q4JIM5	ABL2_MOUSE	Tyrosine-protein kinase ABL2 (EC 2.7.10.2) (Abelson murine leukemia viral oncogene homolog

		2) (Abelson tyrosine-protein kinase 2) (Abelson-related gene protein) (Tyrosine-protein kinase ARG)
Q4PJX1	ODR4_MOUSE	Protein odr-4 homolog (mODR-4)
Q4ZJM9	C1QL4_MOUSE	Complement C1q-like protein 4 (C1q and tumor necrosis factor-related protein 11) (C1q/TNF-related protein 11) (C1qTNF11) (CTRP11)
Q505G8	ZN827_MOUSE	Zinc finger protein 827
Q5BKP2	UBP13_MOUSE	Ubiquitin carboxyl-terminal hydrolase 13 (EC 3.4.19.12) (Deubiquitinating enzyme 13) (Ubiquitin thioesterase 13) (Ubiquitin-specific-processing protease 13)
Q5DQQ6	WFD13_MOUSE	WAP four-disulfide core domain protein 13 (WAP four-disulfide core domain 13-like 1)
Q5DTT3	TASO2_MOUSE	Protein TASOR 2 (Peripheral benzodiazepine receptor-associated protein 20)
Q5DTU0	AF1L2_MOUSE	Actin filament-associated protein 1-like 2 (AFAP1-like protein 2)
Q5EBI0	S2610_MOUSE	Solute carrier family 26 member 10
Q5F2E7	NUFP2_MOUSE	Nuclear fragile X mental retardation-interacting protein 2 (82 kDa FMRP-interacting protein) (82-FIP) (FMRP-interacting protein 2)
Q5GIG6	TNI3K_MOUSE	Serine/threonine-protein kinase TNNI3K (EC 2.7.11.1) (Cardiac ankyrin repeat kinase) (TNNI3-interacting kinase)
Q5MJS3	FA20C_MOUSE	Extracellular serine/threonine protein kinase FAM20C (EC 2.7.11.1) (Dentin matrix protein 4) (DMP-4) (Golgi-enriched fraction casein kinase) (GEF-CK)
Q5NCS9	MID49_MOUSE	Mitochondrial dynamics protein MID49 (Mitochondrial dynamics protein of 49 kDa homolog) (Mitochondrial elongation factor 2) (Smith-Magenis syndrome chromosomal region candidate gene 7 protein homolog)
Q5PRF0	HTR5A_MOUSE	HEAT repeat-containing protein 5A
Q5RI75	RASEF_MOUSE	Ras and EF-hand domain-containing protein homolog
Q5S006	LRRK2_MOUSE	Leucine-rich repeat serine/threonine-protein kinase 2 (EC 2.7.11.1) (EC 3.6.5.-)
Q5SSH7	ZZEF1_MOUSE	Zinc finger ZZ-type and EF-hand domain-containing protein 1
Q5SSZ5	TENS3_MOUSE	Tensin-3 (Tensin-like SH2 domain-containing protein 1)
Q5SUV1	RSAD1_MOUSE	Radical S-adenosyl methionine domain-containing protein 1, mitochondrial (Putative heme chaperone)

Q5U458	DJC11_MOUSE	DnaJ homolog subfamily C member 11
Q60592	MAST2_MOUSE	Microtubule-associated serine/threonine-protein kinase 2 (EC 2.7.11.1)
Q60604	ADSV_MOUSE	Adseverin (Gelsolin-like protein) (Scinderin)
Q60648	SAP3_MOUSE	Ganglioside GM2 activator (Cerebroside sulfate activator protein) (GM2-AP) (Sphingolipid activator protein 3) (SAP-3)
Q60675	LAMA2_MOUSE	Laminin subunit alpha-2 (Laminin M chain) (Laminin-12 subunit alpha) (Laminin-2 subunit alpha) (Laminin-4 subunit alpha) (Merosin heavy chain)
Q60823	AKT2_MOUSE	RAC-beta serine/threonine-protein kinase (EC 2.7.11.1) (Protein kinase Akt-2) (Protein kinase B beta) (PKB beta) (RAC-PK-beta)
Q60825	NPT2A_MOUSE	Sodium-dependent phosphate transport protein 2A (Sodium-phosphate transport protein 2A) (Na(+)-dependent phosphate cotransporter 2A) (NaPi-7) (Sodium/phosphate cotransporter 2A) (Na(+)/Pi cotransporter 2A) (NaPi-2a) (Solute carrier family 34 member 1)
Q60928	GGT1_MOUSE	Glutathione hydrolase 1 proenzyme (EC 3.4.19.13) (Gamma-glutamyltransferase 1) (Gamma-glutamyltranspeptidase 1) (GGT 1) (EC 2.3.2.2) (Leukotriene-C4 hydrolase) (EC 3.4.19.14) (CD antigen CD224) [Cleaved into: Glutathione hydrolase 1 heavy chain; Glutathione hydrolase 1 light chain]
Q60943	I17RA_MOUSE	Interleukin-17 receptor A (IL-17 receptor A) (IL-17RA) (CD antigen CD217)
Q60952	CP250_MOUSE	Centrosome-associated protein CEP250 (250 kDa centrosomal protein) (Cep250) (Centrosomal Nek2-associated protein 1) (C-Nap1) (Centrosomal protein 2) (Intranuclear matrix protein)
Q60963	PAFA_MOUSE	Platelet-activating factor acetylhydrolase (PAF acetylhydrolase) (EC 3.1.1.47) (1-alkyl-2-acetylglycerophosphocholine esterase) (2-acetyl-1-alkylglycerophosphocholine esterase) (LDL-associated phospholipase A2) (LDL-PLA(2)) (PAF 2-acylhydrolase)
Q60991	CP7B1_MOUSE	Cytochrome P450 7B1 (24-hydroxycholesterol 7-alpha-hydroxylase) (EC 1.14.14.26) (25/26-hydroxycholesterol 7-alpha-hydroxylase) (EC 1.14.14.29) (3-hydroxysteroid 7-alpha hydroxylase) (Hippocampal transcript 1 protein) (HCT-1) (Oxysterol 7-alpha-hydroxylase)

Q61127	NAB2_MOUSE	NGFI-A-binding protein 2 (EGR-1-binding protein 2)
Q61147	CERU_MOUSE	Ceruloplasmin (EC 1.16.3.1) (Ferroxidase)
Q61165	SL9A1_MOUSE	Sodium/hydrogen exchanger 1 (Na(+)/H(+) exchanger 1) (NHE-1) (Solute carrier family 9 member 1)
Q61199	NXPH2_MOUSE	Neurexophilin-2
Q61214	DYR1A_MOUSE	Dual specificity tyrosine-phosphorylation-regulated kinase 1A (EC 2.7.12.1) (Dual specificity YAK1-related kinase) (MP86) (Protein kinase minibrain homolog) (MNBH)
Q61329	ZFHX3_MOUSE	Zinc finger homeobox protein 3 (AT motif-binding factor 1) (AT-binding transcription factor 1) (Alpha-fetoprotein enhancer-binding protein) (Zinc finger homeodomain protein 3) (ZFH-3)
Q61810	LTBP3_MOUSE	Latent-transforming growth factor beta-binding protein 3 (LTBP-3)
Q61830	MRC1_MOUSE	Macrophage mannose receptor 1 (MMR) (CD antigen CD206)
Q62000	MIME_MOUSE	Mimecan (Osteoglycin)
Q62028	PLA2R_MOUSE	Secretory phospholipase A2 receptor (PLA2-R) (PLA2R) (180 kDa secretory phospholipase A2 receptor) (M-type receptor) [Cleaved into: Soluble secretory phospholipase A2 receptor (Soluble PLA2-R) (Soluble PLA2R)]
Q62052	P_MOUSE	P protein (Melanocyte-specific transporter protein) (Pink-eyed dilution protein)
Q62073	M3K7_MOUSE	Mitogen-activated protein kinase kinase kinase 7 (EC 2.7.11.25) (Transforming growth factor-beta-activated kinase 1) (TGF-beta-activated kinase 1)
Q62273	S26A2_MOUSE	Sulfate transporter (Diastrophic dysplasia protein homolog) (ST-OB) (Solute carrier family 26 member 2)
Q62431	ARI3A_MOUSE	AT-rich interactive domain-containing protein 3A (ARID domain-containing protein 3A) (B-cell regulator of IgH transcription) (Bright) (Dead ringer-like protein 1)
Q62521	ZIC3_MOUSE	Zinc finger protein ZIC 3 (Zinc finger protein of the cerebellum 3)
Q63870	CO7A1_MOUSE	Collagen alpha-1(VII) chain (Long-chain collagen) (LC collagen)
Q64010	CRK_MOUSE	Adapter molecule crk (Proto-oncogene c-Crk) (p38)
Q640M1	UT14A_MOUSE	U3 small nucleolar RNA-associated protein 14 homolog A (Juvenile spermatogonial depletion-like X-linked protein) (Jsd-like X-linked protein)

Q640P7	TBCC1_MOUSE	TBCC domain-containing protein 1
Q64299	CCN3_MOUSE	CCN family member 3 (Cellular communication network factor 3) (Nephroblastoma-overexpressed gene protein homolog) (Protein NOV homolog) (NovH)
Q64739	COBA2_MOUSE	Collagen alpha-2(XI) chain
Q66GT5	PTPM1_MOUSE	Phosphatidylglycerophosphatase and protein-tyrosine phosphatase 1 (EC 3.1.3.27) (PTEN-like phosphatase) (Phosphoinositide lipid phosphatase) (Protein-tyrosine phosphatase mitochondrial 1) (EC 3.1.3.16) (EC 3.1.3.48)
Q66JY2	IN80D_MOUSE	INO80 complex subunit D
Q67FY2	BCL9L_MOUSE	B-cell CLL/lymphoma 9-like protein (B-cell lymphoma 9-like protein) (BCL9-like protein) (BCL9-related beta-catenin-binding protein) (Protein BCL9-2)
Q68EF4	GRM4_MOUSE	Metabotropic glutamate receptor 4 (mGluR4)
Q68FE7	T151B_MOUSE	Transmembrane protein 151B
Q68FG3	SPT2_MOUSE	Protein SPT2 homolog (SPT2 domain-containing protein 1)
Q69ZF8	MSL2_MOUSE	E3 ubiquitin-protein ligase MSL2 (EC 2.3.2.-) (E3 ubiquitin-protein transferase MSL2) (Male-specific lethal 2-like 1) (MSL2-like 1) (Male-specific lethal-2 homolog) (MSL-2) (Male-specific lethal-2 homolog 1) (RING finger protein 184)
Q69ZJ7	RIC1_MOUSE	RAB6A-GEF complex partner protein 1 (Protein RIC1 homolog)
Q6A000	MOONR_MOUSE	Protein moonraker (MNR)
Q6DFV8	VWDE_MOUSE	von Willebrand factor D and EGF domain-containing protein
Q6GU68	ISLR_MOUSE	Immunoglobulin superfamily containing leucine-rich repeat protein
Q6GYP7	RGPA1_MOUSE	Ral GTPase-activating protein subunit alpha-1 (GAP-related-interacting partner to E12) (GRIPE) (GTPase-activating RapGAP domain-like 1) (Tuberin-like protein 1) (p240)
Q6KAQ7	ZZZ3_MOUSE	ZZ-type zinc finger-containing protein 3
Q6NVF0	OCRL_MOUSE	Inositol polyphosphate 5-phosphatase OCRL (EC 3.1.3.36) (EC 3.1.3.56) (Inositol polyphosphate 5-phosphatase OCRL-1) (Phosphatidylinositol 3,4,5-triphosphate 5-phosphatase) (EC 3.1.3.86)
Q6NXK7	DPP10_MOUSE	Inactive dipeptidyl peptidase 10 (Dipeptidyl peptidase X) (DPP X)
Q6NZF1	ZC11A_MOUSE	Zinc finger CCCH domain-containing protein 11A

Q6NZL6	TONSL_MOUSE	Tonsoku-like protein (Inhibitor of kappa B-related protein) (I-kappa-B-related protein) (IkappaBR) (NF-kappa-B inhibitor-like protein 2) (Nuclear factor of kappa light polypeptide gene enhancer in B-cells inhibitor-like 2)
Q6P4S8	INT1_MOUSE	Integrator complex subunit 1 (Int1)
Q6P5D8	SMHD1_MOUSE	Structural maintenance of chromosomes flexible hinge domain-containing protein 1 (SMC hinge domain-containing protein 1) (EC 3.6.1.-)
Q6PB44	PTN23_MOUSE	Tyrosine-protein phosphatase non-receptor type 23 (EC 3.1.3.48)
Q6PCP7	GP156_MOUSE	Probable G-protein coupled receptor 156 (GABAB-related G-protein coupled receptor)
Q6PDH0	PHLB1_MOUSE	Pleckstrin homology-like domain family B member 1 (Protein LL5-alpha)
Q6PDQ2	CHD4_MOUSE	Chromodomain-helicase-DNA-binding protein 4 (CHD-4) (EC 3.6.4.12)
Q6PE65	GPT2L_MOUSE	G patch domain-containing protein 2-like
Q6PFD9	NUP98_MOUSE	Nuclear pore complex protein Nup98-Nup96 (EC 3.4.21.-) [Cleaved into: Nuclear pore complex protein Nup98 (98 kDa nucleoporin) (Nucleoporin Nup98) (Nup98); Nuclear pore complex protein Nup96 (96 kDa nucleoporin) (Nucleoporin Nup96) (Nup96)]
Q6PFY1	FCSD1_MOUSE	F-BAR and double SH3 domains protein 1 (Protein nervous wreck 2) (NWK2)
Q6PG95	CRML_MOUSE	Protein cramped-like (Cramped chromatin regulator homolog 1) (Hematological and neurological expressed 1-like protein)
Q6PHN1	CCD57_MOUSE	Coiled-coil domain-containing protein 57
Q6QR59	TCAF3_MOUSE	TRPM8 channel-associated factor 3 (Experimental autoimmune prostatitis antigen 2)
Q6RHW0	K1C9_MOUSE	Keratin, type I cytoskeletal 9 (Cytokeratin-9) (CK-9) (Keratin-9) (K9)
Q6XUX1	DUSTY_MOUSE	Dual serine/threonine and tyrosine protein kinase (EC 2.7.12.1) (Dusty protein kinase) (Dusty PK) (Receptor-interacting serine/threonine-protein kinase 5)
Q6ZPI0	JADE1_MOUSE	Protein Jade-1 (Jade family PHD finger protein 1) (PHD finger protein 17)
Q6ZPS2	CRNS1_MOUSE	Carnosine synthase 1 (EC 6.3.2.11) (ATP-grasp domain-containing protein 1)
Q6ZQ03	FNBP4_MOUSE	Formin-binding protein 4 (Formin-binding protein 30)
Q6ZQL4	WDR43_MOUSE	WD repeat-containing protein 43

Q71FD5	ZNRF2_MOUSE	E3 ubiquitin-protein ligase ZNRF2 (EC 2.3.2.27) (RING-type E3 ubiquitin transferase ZNRF2) (Zinc/RING finger protein 2)
Q76LL6	FHOD3_MOUSE	FH1/FH2 domain-containing protein 3 (Formin homolog overexpressed in spleen 2) (mFHOS2)
Q7M6Y6	MRO2B_MOUSE	Maestro heat-like repeat-containing protein family member 2B (HEAT repeat-containing protein 7B2) (Sperm PKA-interacting factor) (SPIF)
Q7M732	RTL1_MOUSE	Retrotransposon-like protein 1 (Mammalian retrotransposon derived protein 1) (Paternally expressed gene 11 protein) (Retrotransposon-derived protein PEG11)
Q7TN98	CPEB4_MOUSE	Cytoplasmic polyadenylation element-binding protein 4 (CPE-BP4) (CPE-binding protein 4) (mCPEB-4)
Q7TNF9	F117A_MOUSE	Protein FAM117A
Q7TNT2	FACR2_MOUSE	Fatty acyl-CoA reductase 2 (EC 1.2.1.84)
Q7TPD0	INT3_MOUSE	Integrator complex subunit 3 (Int3) (SOSS complex subunit A) (Sensor of single-strand DNA complex subunit A) (SOSS-A) (Sensor of ssDNA subunit A)
Q7TPS5	C2CD5_MOUSE	C2 domain-containing protein 5 (138 kDa C2 domain-containing phosphoprotein)
Q7TQ07	DPOLN_MOUSE	DNA polymerase nu (EC 2.7.7.7)
Q7TQ48	SRCA_MOUSE	Sarcalumenin
Q7TQG0	ZBTB5_MOUSE	Zinc finger and BTB domain-containing protein 5 (Transcription factor ZNF-POZ)
Q7TS75	AMER1_MOUSE	APC membrane recruitment protein 1 (Amer1) (Protein FAM123B)
Q7TSG5	SH321_MOUSE	SH3 domain-containing protein 21
Q7TSJ2	MAP6_MOUSE	Microtubule-associated protein 6 (MAP-6) (Stable tubule-only polypeptide) (STOP)
Q7TSQ8	PDPR_MOUSE	Pyruvate dehydrogenase phosphatase regulatory subunit, mitochondrial (PDPr)
Q80SS6	GPBAR_MOUSE	G-protein coupled bile acid receptor 1 (Membrane-type receptor for bile acids) (M-BAR)
Q80SU3	ZAR1_MOUSE	Zygote arrest protein 1 (Oocyte-specific maternal effect factor)
Q80T32	AGRD1_MOUSE	Adhesion G-protein coupled receptor D1 (G-protein coupled receptor 133) (G-protein coupled receptor PGR25)
Q80TL1	ADCY2_MOUSE	Adenylate cyclase type 2 (EC 4.6.1.1) (ATP pyrophosphate-lyase 2) (Adenylate cyclase type II) (Adenylyl cyclase 2)
Q80TY4	ST18_MOUSE	Suppression of tumorigenicity 18 protein

Q80UK0	SESD1_MOUSE	SEC14 domain and spectrin repeat-containing protein 1 (Huntingtin-interacting protein-like protein)
Q80V62	FACD2_MOUSE	Fanconi anemia group D2 protein homolog (Protein FACD2)
Q80VD1	FA98B_MOUSE	Protein FAM98B
Q80VM7	ANR24_MOUSE	Ankyrin repeat domain-containing protein 24
Q80VP1	EPN1_MOUSE	Epsin-1 (EPS-15-interacting protein 1) (Intersectin-EH-binding protein 1) (Ibp1)
Q80VW5	WHRN_MOUSE	Whirlin
Q80VW7	AKNA_MOUSE	Microtubule organization protein AKNA (AT-hook-containing transcription factor)
Q80W04	TMCC2_MOUSE	Transmembrane and coiled-coil domains protein 2
Q80W94	MOGT2_MOUSE	2-acylglycerol O-acyltransferase 2 (EC 2.3.1.22) (Acyl-CoA:monoacylglycerol acyltransferase 2) (MGAT2) (Diacylglycerol acyltransferase 2-like protein 5) (Monoacylglycerol O-acyltransferase 1-like) (Monoacylglycerol O-acyltransferase 2)
Q80WQ9	ZBED4_MOUSE	Zinc finger BED domain-containing protein 4
Q80WY3	NANO1_MOUSE	Nanos homolog 1 (NOS-1)
Q80XR2	AT2C1_MOUSE	Calcium-transporting ATPase type 2C member 1 (ATPase 2C1) (EC 7.2.2.10) (ATP-dependent Ca(2+) pump PMR1)
Q80Y20	ALKB8_MOUSE	Alkylated DNA repair protein alkB homolog 8 (EC 1.14.11.-) (Probable alpha-ketoglutarate-dependent dioxygenase ABH8) (S-adenosyl-L-methionine-dependent tRNA methyltransferase ABH8) (tRNA (carboxymethyluridine(34)-5-O)-methyltransferase ABH8) (EC 2.1.1.-) (EC 2.1.1.229)
Q80Z60	EFCE2_MOUSE	EEF1AKMT4-ECE2 readthrough transcript protein (EC 3.4.24.71) [Includes: Methyltransferase-like region (EC 2.1.1.-); Endothelin-converting enzyme 2 region (EC 3.4.24.71)]
Q80ZW2	THEM6_MOUSE	Protein THEM6
Q8BFR1	ZCCHL_MOUSE	Zinc finger CCCH-type antiviral protein 1-like
Q8BG87	TET3_MOUSE	Methylcytosine dioxygenase TET3 (EC 1.14.11.n2)
Q8BGJ0	ZDH15_MOUSE	Palmitoyltransferase ZDHHC15 (EC 2.3.1.225) (Zinc finger DHHC domain-containing protein 15) (DHHC-15)
Q8BGR2	LRC8D_MOUSE	Volume-regulated anion channel subunit LRRC8D (Leucine-rich repeat-containing protein 5) (Leucine-rich repeat-containing protein 8D)
Q8BH79	ANO10_MOUSE	Anoctamin-10 (Transmembrane protein 16K)

Q8BHG1	NRDC_MOUSE	Nardilysin (EC 3.4.24.61) (N-arginine dibasic convertase) (NRD convertase) (NRD-C) (Nardilysin convertase)
Q8BIA4	FBXW8_MOUSE	F-box/WD repeat-containing protein 8 (F-box and WD-40 domain-containing protein 8)
Q8BIR2	ASTE1_MOUSE	Protein asteroid homolog 1
Q8BJQ4	TCAM2_MOUSE	TIR domain-containing adapter molecule 2 (TICAM-2) (TRIF-related adapter molecule) (Toll/interleukin-1 receptor domain-containing protein)
Q8BK03	MIGA2_MOUSE	Mitoguardin 2 (Protein FAM73B)
Q8BKX1	BAIP2_MOUSE	Brain-specific angiogenesis inhibitor 1-associated protein 2 (BAI-associated protein 2) (BAI1-associated protein 2) (Insulin receptor substrate protein of 53 kDa) (IRSp53) (Insulin receptor substrate p53) (Insulin receptor tyrosine kinase 53 kDa substrate)
Q8BL99	DOP1_MOUSE	Protein dopey-1
Q8BLA8	TRPA1_MOUSE	Transient receptor potential cation channel subfamily A member 1 (Ankyrin-like with transmembrane domains protein 1) (Wasabi receptor)
Q8BM47	PKHM3_MOUSE	Pleckstrin homology domain-containing family M member 3 (PH domain-containing family M member 3) (Differentiation-associated protein)
Q8BM96	AGRG7_MOUSE	Adhesion G-protein coupled receptor G7 (G-protein coupled receptor 128)
Q8BMA5	NPAT_MOUSE	Protein NPAT
Q8BMB3	IF4E2_MOUSE	Eukaryotic translation initiation factor 4E type 2 (eIF-4E type 2) (eIF4E type 2) (eIF4E-2) (mRNA cap-binding protein type 2) (Eukaryotic translation initiation factor 4E-like 3) (eIF4E-like protein 4E-LP)
Q8BMK0	CEP85_MOUSE	Centrosomal protein of 85 kDa (Cep85) (Coiled-coil domain-containing protein 21)
Q8BQM4	HEAT3_MOUSE	HEAT repeat-containing protein 3
Q8BR27	F214B_MOUSE	Protein FAM214B
Q8BR65	SDS3_MOUSE	Sin3 histone deacetylase corepressor complex component SDS3 (Suppressor of defective silencing 3 protein homolog)
Q8BT14	CNOT4_MOUSE	CCR4-NOT transcription complex subunit 4 (EC 2.3.2.27) (CCR4-associated factor 4) (E3 ubiquitin-protein ligase CNOT4) (Potential transcriptional repressor NOT4Hp) (RING-type E3 ubiquitin transferase CNOT4)

Q8BTF7	DNLI4_MOUSE	DNA ligase 4 (EC 6.5.1.1) (DNA ligase IV) (Polydeoxyribonucleotide synthase [ATP] 4)
Q8BTW8	CK5P1_MOUSE	CDK5 regulatory subunit-associated protein 1 (CDK5 activator-binding protein C42)
Q8BU11	TOX4_MOUSE	TOX high mobility group box family member 4 (Epidermal Langerhans cell protein LCP1)
Q8BUB4	WDFY2_MOUSE	WD repeat and FYVE domain-containing protein 2 (Propeller-FYVE protein) (Prof) (WD40- and FYVE domain-containing protein 2)
Q8BUH7	RNF26_MOUSE	E3 ubiquitin-protein ligase RNF26 (EC 2.3.2.27) (RING finger protein 26)
Q8BVF9	AMZ1_MOUSE	Archaemetzincin-1 (EC 3.4.-.-) (Archeobacterial metalloproteinase-like protein 1)
Q8BVU0	LRCH3_MOUSE	DISP complex protein LRCH3 (Leucine-rich repeat and calponin homology domain-containing protein 3)
Q8BXN9	TM87A_MOUSE	Transmembrane protein 87A
Q8BXQ2	PIGT_MOUSE	GPI transamidase component PIG-T (Neuronal development-associated protein 7) (Phosphatidylinositol-glycan biosynthesis class T protein)
Q8BXX9	CC169_MOUSE	Coiled-coil domain-containing protein 169
Q8BYM8	SYCM_MOUSE	Probable cysteine--tRNA ligase, mitochondrial (EC 6.1.1.16) (Cysteinyl-tRNA synthetase) (CysRS)
Q8BYR5	CAPS2_MOUSE	Calcium-dependent secretion activator 2 (Calcium-dependent activator protein for secretion 2) (CAPS-2)
Q8BZE1	DMBTL_MOUSE	Putative DMBT1-like protein
Q8BZH4	POGZ_MOUSE	Pogo transposable element with ZNF domain
Q8BZL1	NEUR4_MOUSE	Sialidase-4 (EC 3.2.1.18) (N-acetyl-alpha-neuraminidase 4) (Neuraminidase 4)
Q8BZQ7	ANC2_MOUSE	Anaphase-promoting complex subunit 2 (APC2) (Cyclosome subunit 2)
Q8C0M9	ASGL1_MOUSE	Isoaspartyl peptidase/L-asparaginase (EC 3.4.19.5) (EC 3.5.1.1) (Asparaginase-like protein 1) (Beta-aspartyl-peptidase) (Isoaspartyl dipeptidase) (L-asparagine amidohydrolase) [Cleaved into: Isoaspartyl peptidase/L-asparaginase alpha chain; Isoaspartyl peptidase/L-asparaginase beta chain]
Q8C0S1	DI3L1_MOUSE	DIS3-like exonuclease 1 (EC 3.1.13.-)
Q8C0S4	TT21A_MOUSE	Tetratricopeptide repeat protein 21A (TPR repeat protein 21A) (Tetratricopeptide repeat-containing hedgehog modulator 2)
Q8C1D8	IWS1_MOUSE	Protein IWS1 homolog (IWS1-like protein)

Q8C1W1	VASH1_MOUSE	Tubulinyl-Tyr carboxypeptidase 1 (EC 3.4.17.17) (Tyrosine carboxypeptidase 1) (TTCP 1) (Vasohibin-1)
Q8C353	TM252_MOUSE	Transmembrane protein 252
Q8C419	GP158_MOUSE	Probable G-protein coupled receptor 158
Q8C4Q9	TM154_MOUSE	Transmembrane protein 154
Q8C5H1	ANO4_MOUSE	Anoctamin-4 (Transmembrane protein 16D)
Q8C627	F221B_MOUSE	Protein FAM221B
Q8C6B2	RTKN_MOUSE	Rhotekin
Q8C6C9	LEG1H_MOUSE	Protein LEG1 homolog
Q8C6N3	SYT15_MOUSE	Synaptotagmin-15 (Synaptotagmin XV) (SytXV)
Q8C6S9	CFA54_MOUSE	Cilia- and flagella-associated protein 54
Q8C9S4	CC186_MOUSE	Coiled-coil domain-containing protein 186 (Oocyte-testis gene 1 protein)
Q8CBW4	DC121_MOUSE	DDB1- and CUL4-associated factor 12-like protein 1 (WD repeat-containing protein 40B)
Q8CBY3	LENG8_MOUSE	Leukocyte receptor cluster member 8 homolog
Q8CD54	PIEZ2_MOUSE	Piezo-type mechanosensitive ion channel component 2 (Protein FAM38B)
Q8CDI2	FBX43_MOUSE	F-box only protein 43 (Endogenous meiotic inhibitor 2)
Q8CDK2	CBPC2_MOUSE	Cytosolic carboxypeptidase 2 (EC 3.4.17.-) (ATP/GTP-binding protein-like 2)
Q8CDN9	LRRC9_MOUSE	Leucine-rich repeat-containing protein 9
Q8CDU6	HECD2_MOUSE	Probable E3 ubiquitin-protein ligase HECTD2 (EC 2.3.2.26) (HECT domain-containing protein 2) (HECT-type E3 ubiquitin transferase HECTD2)
Q8CEC0	NUP88_MOUSE	Nuclear pore complex protein Nup88 (88 kDa nucleoporin) (Nucleoporin Nup88)
Q8CEE0	CEP57_MOUSE	Centrosomal protein of 57 kDa (Cep57) (Testis-specific protein 57) (Translokin)
Q8CEE7	RDH13_MOUSE	Retinol dehydrogenase 13 (EC 1.1.1.300)
Q8CFE3	RCOR1_MOUSE	REST corepressor 1 (Protein CoREST)
Q8CFE5	BTBD7_MOUSE	BTB/POZ domain-containing protein 7 (Function-unkown protein 1)
Q8CGK3	LONM_MOUSE	Lon protease homolog, mitochondrial (EC 3.4.21.53) (Lon protease-like protein) (LONP) (Mitochondrial ATP-dependent protease Lon) (Serine protease 15)
Q8CGW4	SOX30_MOUSE	Transcription factor SOX-30
Q8CHI8	EP400_MOUSE	E1A-binding protein p400 (EC 3.6.4.-) (Domino homolog) (mDomino) (p400 kDa SWI2/SNF2-related protein)

Q8CHP0	ZC3H3_MOUSE	Zinc finger CCCH domain-containing protein 3 (Smad-interacting CPSF-like factor)
Q8CHR6	DPYD_MOUSE	Dihydropyrimidine dehydrogenase [NADP(+)] (DHPDHase) (DPD) (EC 1.3.1.2) (Dihydrothymine dehydrogenase) (Dihydrouracil dehydrogenase)
Q8CHY3	DYM_MOUSE	Dymeclin
Q8CI03	FWCH1_MOUSE	FLYWCH-type zinc finger-containing protein 1
Q8CII8	SYNE4_MOUSE	Nesprin-4 (KASH domain-containing protein 4) (KASH4) (Nuclear envelope spectrin repeat protein 4)
Q8CIM1	LRC45_MOUSE	Leucine-rich repeat-containing protein 45
Q8CJ19	MICA3_MOUSE	[F-actin]-monooxygenase MICAL3 (EC 1.14.13.225) (Molecule interacting with CasL protein 3) (MICAL-3)
Q8CJ53	CIP4_MOUSE	Cdc42-interacting protein 4 (Thyroid receptor-interacting protein 10) (TR-interacting protein 10) (TRIP-10)
Q8JZS7	HMGC2_MOUSE	3-hydroxy-3-methylglutaryl-CoA lyase, cytoplasmic (EC 4.1.3.4) (3-hydroxy-3-methylglutaryl-CoA lyase-like protein 1)
Q8K045	PKN3_MOUSE	Serine/threonine-protein kinase N3 (EC 2.7.11.13) (Protein kinase PKN-beta) (Protein-kinase C-related kinase 3)
Q8K0T7	UN13C_MOUSE	Protein unc-13 homolog C (Munc13-3)
Q8K0Z9	GP153_MOUSE	Probable G-protein coupled receptor 153 (G-protein coupled receptor PGR1)
Q8K177	KCP3_MOUSE	Keratinocyte-associated protein 3 (KCP-3)
Q8K1G2	LBN_MOUSE	Limbin
Q8K1H7	T11L2_MOUSE	T-complex protein 11-like protein 2
Q8K2W3	TXD11_MOUSE	Thioredoxin domain-containing protein 11
Q8K2X1	AT10D_MOUSE	Probable phospholipid-transporting ATPase VD (EC 7.6.2.1) (ATPase class V type 10D) (P4-ATPase flippase complex alpha subunit ATP10D)
Q8K2X3	STN1_MOUSE	CST complex subunit STN1 (Alpha-accessory factor of 44 kDa) (AAF-44) (AAF44) (Oligonucleotide/oligosaccharide-binding fold-containing protein 1) (Suppressor of cdc thirteen homolog)
Q8K371	AMOL2_MOUSE	Angiomotin-like protein 2
Q8K387	UBP45_MOUSE	Ubiquitin carboxyl-terminal hydrolase 45 (EC 3.4.19.12) (Deubiquitinating enzyme 45) (Ubiquitin thioesterase 45) (Ubiquitin-specific-processing protease 45)

Q8K394	PLCL2_MOUSE	Inactive phospholipase C-like protein 2 (PLC-L(2)) (PLC-L2) (Phospholipase C-L2) (Phospholipase C-epsilon-2) (PLC-epsilon-2)
Q8K3E5	AHI1_MOUSE	Jouberin (Abelson helper integration site 1 protein) (AHI-1)
Q8K3H0	DP13A_MOUSE	DCC-interacting protein 13-alpha (Dip13-alpha) (Adapter protein containing PH domain, PTB domain and leucine zipper motif 1)
Q8K3M5	CABL2_MOUSE	CDK5 and ABL1 enzyme substrate 2 (Interactor with CDK3 2) (Ik3-2)
Q8K402	TBX22_MOUSE	T-box transcription factor TBX22 (T-box protein 22)
Q8K4B2	IRAK3_MOUSE	Interleukin-1 receptor-associated kinase 3 (IRAK-3) (EC 2.7.11.1) (IL-1 receptor-associated kinase M) (IRAK-M)
Q8K4C2	I17RC_MOUSE	Interleukin-17 receptor C (IL-17 receptor C) (IL-17RC) (Interleukin-17 receptor-like protein) (IL-17RL) (ZcytoR14)
Q8K4E0	ALMS1_MOUSE	Alstrom syndrome protein 1 homolog
Q8K4F0	CD226_MOUSE	CD226 antigen (Platelet and T-cell activation antigen 1) (CD antigen CD226)
Q8K4G1	LTBP4_MOUSE	Latent-transforming growth factor beta-binding protein 4 (LTBP-4)
Q8K4H1	KFA_MOUSE	Kynurenine formamidase (KFA) (KFase) (EC 3.5.1.9) (Arylformamidase) (N-formylkynurenine formamidase) (FKF)
Q8K4S1	PLCE1_MOUSE	1-phosphatidylinositol 4,5-bisphosphate phosphodiesterase epsilon-1 (EC 3.1.4.11) (Phosphoinositide phospholipase C-epsilon-1) (Phospholipase C-epsilon-1) (PLC-epsilon-1)
Q8K4Z3	NNRE_MOUSE	NAD(P)H-hydrate epimerase (EC 5.1.99.6) (Apolipoprotein A-I-binding protein) (AI-BP) (NAD(P)HX epimerase)
Q8R088	GLP3L_MOUSE	Golgi phosphoprotein 3-like
Q8R0W1	CIPC_MOUSE	CLOCK-interacting pacemaker (CLOCK-interacting circadian protein)
Q8R139	S29A4_MOUSE	Equilibrative nucleoside transporter 4 (Solute carrier family 29 member 4)
Q8R184	F110A_MOUSE	Protein FAM110A
Q8R1Q9	RBSK_MOUSE	Ribokinase (RK) (EC 2.7.1.15)
Q8R1Z4	PPR42_MOUSE	Protein phosphatase 1 regulatory subunit 42 (Leucine-rich repeat-containing protein 67) (Testis leucine-rich repeat protein) (TLRR)
Q8R2G6	CCD80_MOUSE	Coiled-coil domain-containing protein 80 (Up-regulated in BRS-3 deficient mouse)

Q8R395	COMD5_MOUSE	COMM domain-containing protein 5
Q8R3L2	TCF25_MOUSE	Transcription factor 25 (TCF-25) (Nuclear localized protein 1)
Q8R3Q0	SARAF_MOUSE	Store-operated calcium entry-associated regulatory factor (SARAF) (SOCE-associated regulatory factor) (Transmembrane protein 66)
Q8R493	ACHB4_MOUSE	Neuronal acetylcholine receptor subunit beta-4
Q8R4T9	UT2_MOUSE	Urea transporter 2 (Solute carrier family 14 member 2) (Urea transporter, kidney)
Q8R4X3	RBM12_MOUSE	RNA-binding protein 12 (RNA-binding motif protein 12) (SH3/WW domain anchor protein in the nucleus) (SWAN)
Q8R5F3	OARD1_MOUSE	ADP-ribose glycohydrolase OARD1 (O-acetyl-ADP-ribose deacetylase 1) (EC 3.5.1.-) (Terminal ADP-ribose protein glycohydrolase 1) ([Protein ADP-ribosylglutamate] hydrolase OARD1) (EC 3.2.2.-)
Q8R5M8	CADM1_MOUSE	Cell adhesion molecule 1 (Immunoglobulin superfamily member 4) (IgSF4) (Nectin-like protein 2) (NECL-2) (Spermatogenic immunoglobulin superfamily) (SgIgSF) (Synaptic cell adhesion molecule) (SynCAM) (Tumor suppressor in lung cancer 1) (TSLC-1)
Q8VBX6	MPDZ_MOUSE	Multiple PDZ domain protein (Multi-PDZ domain protein 1)
Q8VC03	EMAL3_MOUSE	Echinoderm microtubule-associated protein-like 3 (EMAP-3)
Q8VC98	PKHA4_MOUSE	Pleckstrin homology domain-containing family A member 4 (PH domain-containing family A member 4) (Phosphoinositol 3-phosphate-binding protein 1) (PEPP-1)
Q8VCC9	SPON1_MOUSE	Spondin-1 (F-spondin)
Q8VCI0	PLBL1_MOUSE	Phospholipase B-like 1 (EC 3.1.1.-) (LAMA-like protein 1) (Lamina ancestor homolog 1) (Phospholipase B domain-containing protein 1) [Cleaved into: Phospholipase B-like 1 chain A; Phospholipase B-like 1 chain B; Phospholipase B-like 1 chain C]
Q8VDD9	PHIP_MOUSE	PH-interacting protein (PHIP) (IRS-1 PH domain-binding protein) (Neuronal differentiation-related protein) (NDRP) (WD repeat-containing protein 11)
Q8VE11	MTMR6_MOUSE	Myotubularin-related protein 6 (Phosphatidylinositol-3,5-bisphosphate 3-phosphatase) (EC 3.1.3.95) (Phosphatidylinositol-3-phosphate phosphatase) (EC 3.1.3.64)

Q8VE88	F1142_MOUSE	Protein FAM114A2
Q8VE94	F110C_MOUSE	Protein FAM110C
Q8VED9	LEGL_MOUSE	Galectin-related protein (Galectin-related protein A) (Lectin galactoside-binding-like protein A)
Q8VHG0	FMO4_MOUSE	Dimethylaniline monooxygenase [N-oxide-forming] 4 (EC 1.14.13.8) (Dimethylaniline oxidase 4) (Hepatic flavin-containing monooxygenase 4) (FMO 4)
Q8VHJ7	PRGC2_MOUSE	Peroxisome proliferator-activated receptor gamma coactivator 1-beta (PGC-1-beta) (PPAR-gamma coactivator 1-beta) (PPARGC-1-beta) (ERR ligand 1)
Q8VHQ4	RB40C_MOUSE	Ras-related protein Rab-40C (SOCS box-containing protein RAR3)
Q8VHW4	CCG5_MOUSE	Voltage-dependent calcium channel gamma-5 subunit (Neuronal voltage-gated calcium channel gamma-5 subunit) (Transmembrane AMPAR regulatory protein gamma-5) (TARP gamma-5)
Q8VIK5	PEAR1_MOUSE	Platelet endothelial aggregation receptor 1 (mPEAR1) (Jagged and Delta protein) (Protein Jedi) (Multiple epidermal growth factor-like domains protein 12) (Multiple EGF-like domains protein 12)
Q91V17	ZNRF1_MOUSE	E3 ubiquitin-protein ligase ZNRF1 (EC 2.3.2.27) (Nerve injury-induced gene 283 protein) (RING-type E3 ubiquitin transferase ZNRF1) (Zinc/RING finger protein 1)
Q91V80	APOF_MOUSE	Apolipoprotein F (Apo-F) (Leukemia virus-inactivating factor) (LVIF)
Q91VJ2	CAVN3_MOUSE	Caveolae-associated protein 3 (Cavin-3) (Protein kinase C delta-binding protein) (Serum deprivation response factor-related gene product that binds to C-kinase)
Q91VT4	CBR4_MOUSE	Carbonyl reductase family member 4 (EC 1.-.-.-) (3-ketoacyl-[acyl-carrier-protein] reductase beta subunit) (KAR beta subunit) (3-oxoacyl-[acyl-carrier-protein] reductase) (EC 1.1.1.-) (Quinone reductase CBR4)
Q91WR6	GINM1_MOUSE	Glycoprotein integral membrane protein 1
Q91X44	GCKR_MOUSE	Glucokinase regulatory protein (GKRP) (Glucokinase regulator)
Q91XD8	S2538_MOUSE	Mitochondrial glycine transporter (Solute carrier family 25 member 38)
Q91XE9	CR3L3_MOUSE	Cyclic AMP-responsive element-binding protein 3-like protein 3 (cAMP-responsive element-binding protein 3-like protein 3) (Transcription factor

		CREB-H) [Cleaved into: Processed cyclic AMP-responsive element-binding protein 3-like protein 3]
Q91XI1	DUS3L_MOUSE	tRNA-dihydrouridine(47) synthase [NAD(P)(+)]-like (EC 1.3.1.-) (tRNA-dihydrouridine synthase 3-like)
Q91XM9	DLG2_MOUSE	Disks large homolog 2 (Channel-associated protein of synapse-110) (Chapsyn-110) (Postsynaptic density protein PSD-93)
Q91XQ0	DYH8_MOUSE	Dynein heavy chain 8, axonemal (Axonemal beta dynein heavy chain 8) (Ciliary dynein heavy chain 8)
Q91XT6	TAC2N_MOUSE	Tandem C2 domains nuclear protein (Membrane targeting tandem C2 domain-containing protein 1) (Tandem C2 protein in nucleus) (Tac2-N)
Q91YJ3	THYN1_MOUSE	Thymocyte nuclear protein 1 (Thymocyte protein Thy28) (mThy28)
Q91YJ5	IF2M_MOUSE	Translation initiation factor IF-2, mitochondrial (IF-2(Mt)) (IF-2Mt) (IF2(mt))
Q91YL7	PG2IP_MOUSE	PGAP2-interacting protein (Cell wall biogenesis protein 43 C-terminal homolog)
Q91YT8	CSCL1_MOUSE	CSC1-like protein 1 (Transmembrane protein 63A)
Q91YW3	DNJC3_MOUSE	DnaJ homolog subfamily C member 3 (Interferon-induced, double-stranded RNA-activated protein kinase inhibitor) (Protein kinase inhibitor of 58 kDa) (Protein kinase inhibitor p58)
Q91ZI0	CELR3_MOUSE	Cadherin EGF LAG seven-pass G-type receptor 3
Q91ZR3	ZHANG_MOUSE	CREB/ATF bZIP transcription factor (Host cell factor-binding transcription factor Zhangfei) (HCF-binding transcription factor Zhangfei) (Tyrosine kinase-associated leucine zipper protein LAZip)
Q91ZS8	RED1_MOUSE	Double-stranded RNA-specific editase 1 (EC 3.5.4.37) (RNA-editing deaminase 1) (RNA-editing enzyme 1) (dsRNA adenosine deaminase)
Q920A0	OPT_MOUSE	Opticin (Oculoglycan)
Q920A7	AFG31_MOUSE	AFG3-like protein 1 (EC 3.4.24.-)
Q920H4	ACM5_MOUSE	Muscarinic acetylcholine receptor M5
Q920P3	BRNP1_MOUSE	BMP/retinoic acid-inducible neural-specific protein 1 (Deleted in bladder cancer protein 1 homolog)
Q921I1	TRFE_MOUSE	Serotransferrin (Transferrin) (Beta-1 metal-binding globulin) (Siderophilin)
Q921V5	MGAT2_MOUSE	Alpha-1,6-mannosyl-glycoprotein 2-beta-N-acetylglucosaminyltransferase (EC 2.4.1.143) (Beta-1,2-N-acetylglucosaminyltransferase II)

		(GlcNAc-T II) (GNT-II) (Mannoside acetylglucosaminyltransferase 2) (N-glycosyl-oligosaccharide-glycoprotein N-acetylglucosaminyltransferase II)
Q922S8	KIF2C_MOUSE	Kinesin-like protein KIF2C (Mitotic centromere-associated kinesin) (MCAK)
Q924H7	WAC_MOUSE	WW domain-containing adapter protein with coiled-coil
Q925H0	ASIC2_MOUSE	Acid-sensing ion channel 2 (ASIC2) (Amiloride-sensitive brain sodium channel) (Amiloride-sensitive cation channel 1, neuronal) (Brain sodium channel 1) (BNC1) (BNaC1)
Q925I1	ATAD3_MOUSE	ATPase family AAA domain-containing protein 3 (AAA-ATPase TOB3)
Q925I4	TS1R2_MOUSE	Taste receptor type 1 member 2 (G-protein coupled receptor 71) (Sweet taste receptor T1R2)
Q925N1	SFXN4_MOUSE	Sideroflexin-4
Q925S4	IL24_MOUSE	Interleukin-24 (IL-24) (IL-4-induced secreted protein) (Melanoma differentiation-associated gene 7 protein) (MDA-7) (Th2-specific cytokine FISP)
Q96DY5	RN112_MOUSE	RING finger protein 112 (EC 2.3.2.27) (Brain finger protein) (Neurolastin) (Zinc finger protein 179)
Q99J23	GHDC_MOUSE	GH3 domain-containing protein
Q99JP6	HOME3_MOUSE	Homer protein homolog 3 (Homer-3)
Q99LM2	CK5P3_MOUSE	CDK5 regulatory subunit-associated protein 3
Q99ME3	SNCAP_MOUSE	Synphilin-1 (Alpha-synuclein-interacting protein)
Q99MQ5	COPA1_MOUSE	Collagen alpha-1(XXV) chain (CLAC-P) [Cleaved into: Collagen-like Alzheimer amyloid plaque component (CLAC)]
Q99MW1	STK31_MOUSE	Serine/threonine-protein kinase 31 (EC 2.7.11.1)
Q99N69	LPXN_MOUSE	Leupaxin
Q99NB8	UBQL4_MOUSE	Ubiquilin-4 (Ataxin-1 interacting ubiquitin-like protein) (A1Up) (Ataxin-1 ubiquitin-like-interacting protein A1U) (Connexin43-interacting protein of 75 kDa) (CIP75)
Q99NH0	ANR17_MOUSE	Ankyrin repeat domain-containing protein 17 (Ankyrin repeat domain-containing protein FOE) (Gene trap ankyrin repeat protein)
Q99NH8	TREM2_MOUSE	Triggering receptor expressed on myeloid cells 2 (TREM-2) (Triggering receptor expressed on monocytes 2)
Q99P47	CNTP4_MOUSE	Contactin-associated protein-like 4 (Cell recognition molecule Caspr4)

Q99P65	S29A3_MOUSE	Equilibrative nucleoside transporter 3 (mENT3) (Solute carrier family 29 member 3)
Q99PI5	LPIN2_MOUSE	Phosphatidate phosphatase LPIN2 (EC 3.1.3.4) (Lipin-2)
Q99PU8	DHX30_MOUSE	ATP-dependent RNA helicase DHX30 (EC 3.6.4.13) (DEAH box protein 30)
Q9CR62	M2OM_MOUSE	Mitochondrial 2-oxoglutarate/malate carrier protein (OGCP) (Solute carrier family 25 member 11)
Q9CW79	GOGA1_MOUSE	Golgin subfamily A member 1 (Golgin-97)
Q9CWF6	BBS2_MOUSE	Bardet-Biedl syndrome 2 protein homolog
Q9CWH4	RE114_MOUSE	Meiotic recombination protein REC114
Q9CWY3	SETD6_MOUSE	N-lysine methyltransferase SETD6 (EC 2.1.1.-) (SET domain-containing protein 6)
Q9CXB8	ALPK1_MOUSE	Alpha-protein kinase 1 (EC 2.7.11.1)
Q9CY73	RM44_MOUSE	39S ribosomal protein L44, mitochondrial (L44mt) (MRP-L44) (EC 3.1.26.-)
Q9CYC3	TM39A_MOUSE	Transmembrane protein 39A
Q9CYH5	GFOD2_MOUSE	Glucose-fructose oxidoreductase domain-containing protein 2 (EC 1.-.-.-)
Q9CYK2	QPCT_MOUSE	Glutaminyl-peptide cyclotransferase (EC 2.3.2.5) (Glutaminyl cyclase) (QC) (Glutaminyl-tRNA cyclotransferase)
Q9CYW4	HDHD3_MOUSE	Haloacid dehalogenase-like hydrolase domain-containing protein 3
Q9CZ28	SNF8_MOUSE	Vacuolar-sorting protein SNF8 (ESCRT-II complex subunit VPS22)
Q9CZ49	KLH35_MOUSE	Kelch-like protein 35
Q9CZK6	ANKS3_MOUSE	Ankyrin repeat and SAM domain-containing protein 3
Q9CZX7	PP4P2_MOUSE	Type 2 phosphatidylinositol 4,5-bisphosphate 4-phosphatase (Type 2 PtdIns-4,5-P2 4-Ptase) (EC 3.1.3.78) (PtdIns-4,5-P2 4-Ptase II) (Transmembrane protein 55A)
Q9D0G0	RT30_MOUSE	28S ribosomal protein S30, mitochondrial (MRP-S30) (S30mt)
Q9D0U6	MAF1_MOUSE	Repressor of RNA polymerase III transcription MAF1 homolog
Q9D176	SUSD3_MOUSE	Sushi domain-containing protein 3
Q9D1G3	HHATL_MOUSE	Protein-cysteine N-palmitoyltransferase HHAT-like protein (Glycerol uptake/transporter homolog) (Hedgehog acyltransferase-like protein)
Q9D236	HTRA3_MOUSE	Serine protease HTRA3 (EC 3.4.21.-) (High-temperature requirement factor A3) (Pregnancy-

		related serine protease) (Toll-associated serine protease)
Q9D2J7	ANKE1_MOUSE	Ankyrin repeat and EF-hand domain-containing protein 1 (Ankyrin repeat domain-containing protein 5)
Q9D2X8	CH076_MOUSE	Uncharacterized protein C8orf76 homolog
Q9D312	K1C20_MOUSE	Keratin, type I cytoskeletal 20 (Cytokeratin-20) (CK-20) (Keratin-20) (K20)
Q9D3X9	MFA3L_MOUSE	Microfibrillar-associated protein 3-like
Q9D4P0	ARL5B_MOUSE	ADP-ribosylation factor-like protein 5B (ADP-ribosylation factor-like protein 8)
Q9D5V6	SYAP1_MOUSE	Synapse-associated protein 1 (BSD domain-containing signal transducer and Akt interactor protein) (BSTA)
Q9D668	ARRD2_MOUSE	Arrestin domain-containing protein 2
Q9D710	TMX2_MOUSE	Thioredoxin-related transmembrane protein 2 (Thioredoxin domain-containing protein 14)
Q9D711	PIR_MOUSE	Pirin (EC 1.13.11.24) (Probable quercetin 2,3-dioxygenase PIR) (Probable quercetinase)
Q9D733	GP2_MOUSE	Pancreatic secretory granule membrane major glycoprotein GP2 (Pancreatic zymogen granule membrane protein GP-2)
Q9D7M1	GID8_MOUSE	Glucose-induced degradation protein 8 homolog (Two hybrid-associated protein 1 with RanBPM) (Twa1)
Q9D952	EVPL_MOUSE	Envoplakin (210 kDa cornified envelope precursor protein) (p210)
Q9D9B7	PRR30_MOUSE	Proline-rich protein 30
Q9D9C7	CC062_MOUSE	Uncharacterized protein C3orf62 homolog
Q9D9M0	PRS52_MOUSE	Serine protease 52 (EC 3.4.21.-) (Testicular-specific serine protease 3)
Q9DA15	THEGL_MOUSE	Testicular haploid expressed gene protein-like
Q9DAA7	CA100_MOUSE	Uncharacterized protein C1orf100 homolog
Q9DAC9	PO5F2_MOUSE	POU domain, class 5, transcription factor 2 (Sperm 1 POU domain transcription factor) (SPRM-1)
Q9DAQ4	CB081_MOUSE	Uncharacterized protein C2orf81 homolog
Q9DBD5	PELP1_MOUSE	Proline-, glutamic acid- and leucine-rich protein 1 (Modulator of non-genomic activity of estrogen receptor)
Q9DBP0	NPT2B_MOUSE	Sodium-dependent phosphate transport protein 2B (Sodium-phosphate transport protein 2B) (Na(+)-dependent phosphate cotransporter 2B) (Sodium/phosphate cotransporter 2B) (Na(+)/Pi cotransporter 2B) (NaPi-2b) (Solute carrier family 34 member 2)

Q9DBS5	KLC4_MOUSE	Kinesin light chain 4 (KLC 4) (Kinesin-like protein 8)
Q9DBU5	RNF6_MOUSE	E3 ubiquitin-protein ligase RNF6 (EC 2.3.2.27) (RING-type E3 ubiquitin transferase RNF6) (RLIM-like protein)
Q9DBY4	TRIL_MOUSE	TLR4 interactor with leucine rich repeats (Leucine-rich repeat-containing protein KIAA0644)
Q9DC26	S46A3_MOUSE	Solute carrier family 46 member 3
Q9EPB5	SERHL_MOUSE	Serine hydrolase-like protein (SHL) (EC 3.1.-.-)
Q9EPS3	GLCE_MOUSE	D-glucuronyl C5-epimerase (EC 5.1.3.17) (Heparan sulfate C5-epimerase) (Hsepi) (Heparin sulfate C5-epimerase) (Heparin/heparan sulfate:glucuronic acid C5-epimerase) (Heparosan-N-sulfate-glucuronate 5-epimerase)
Q9EPX5	FXL12_MOUSE	F-box/LRR-repeat protein 12 (F-box and leucine-rich repeat protein 12) (F-box protein FBL12)
Q9EQH2	ERAP1_MOUSE	Endoplasmic reticulum aminopeptidase 1 (EC 3.4.11.-) (ARTS-1) (Adipocyte-derived leucine aminopeptidase) (A-LAP) (Aminopeptidase PILS) (Puromycin-insensitive leucyl-specific aminopeptidase) (PILS-AP) (VEGF-induced aminopeptidase)
Q9EQY0	ERN1_MOUSE	Serine/threonine-protein kinase/endoribonuclease IRE1 (Endoplasmic reticulum-to-nucleus signaling 1) (Inositol-requiring protein 1) (Ire1-alpha) (IRE1a) [Includes: Serine/threonine-protein kinase (EC 2.7.11.1); Endoribonuclease (EC 3.1.26.-)]
Q9ER69	FL2D_MOUSE	Pre-mRNA-splicing regulator WTAP (Female-lethal(2)D homolog) (WT1-associated protein) (Wilms tumor 1-associating protein)
Q9ERH6	MOAP1_MOUSE	Modulator of apoptosis 1 (MAP-1)
Q9ES34	UBE3B_MOUSE	Ubiquitin-protein ligase E3B (EC 2.3.2.26) (HECT-type ubiquitin transferase E3B)
Q9EST3	4ET_MOUSE	Eukaryotic translation initiation factor 4E transporter (4E-T) (eIF4E transporter) (Eukaryotic translation initiation factor 4E nuclear import factor 1)
Q9ESY9	GILT_MOUSE	Gamma-interferon-inducible lysosomal thiol reductase (EC 1.8.-.-) (Gamma-interferon-inducible protein IP-30) (Lysosomal thiol reductase IP30)
Q9JHJ5	5HT3B_MOUSE	5-hydroxytryptamine receptor 3B (5-HT3-B) (5-HT3B) (Serotonin receptor 3B)
Q9JII2	PR5A1_MOUSE	Prolactin-5A1 (Placental prolactin-like protein L) (PLP-L) (PRL-like protein L)

Q9JIM1	S29A1_MOUSE	Equilibrative nucleoside transporter 1 (Equilibrative nitrobenzylmercaptopurine riboside-sensitive nucleoside transporter) (Equilibrative NBMPR-sensitive nucleoside transporter) (Nucleoside transporter, es-type) (Solute carrier family 29 member 1)
Q9JJ04	B4GT4_MOUSE	Beta-1,4-galactosyltransferase 4 (Beta-1,4-GalTase 4) (Beta4Gal-T4) (b4Gal-T4) (EC 2.4.1.-) (Beta-N-acetylglucosaminyl-glycolipid beta-1,4-galactosyltransferase) (Lactotriaosylceramide beta-1,4-galactosyltransferase) (EC 2.4.1.275) (N-acetyllactosamine synthase) (EC 2.4.1.90) (Nal synthase) (UDP-Gal:beta-GlcNAc beta-1,4-galactosyltransferase 4) (UDP-galactose:beta-N-acetylglucosamine beta-1,4-galactosyltransferase 4)
Q9JJA2	COG8_MOUSE	Conserved oligomeric Golgi complex subunit 8 (COG complex subunit 8) (Component of oligomeric Golgi complex 8)
Q9JJA7	CCNL2_MOUSE	Cyclin-L2 (Cyclin Ania-6b) (Paneth cell-enhanced expression protein) (PCEE)
Q9JKC0	CCL24_MOUSE	C-C motif chemokine 24 (Eosinophil chemotactic protein 2) (Eotaxin-2) (Small-inducible cytokine A24)
Q9JKF6	NECT1_MOUSE	Nectin-1 (Herpes virus entry mediator C) (Herpesvirus entry mediator C) (HveC) (Nectin cell adhesion molecule 1) (Poliovirus receptor-related protein 1) (CD antigen CD111)
Q9JKK8	ATR_MOUSE	Serine/threonine-protein kinase ATR (EC 2.7.11.1) (Ataxia telangiectasia and Rad3-related protein)
Q9JKT2	TR119_MOUSE	Taste receptor type 2 member 119 (T2R119) (Taste receptor type 2 member 19) (T2R19)
Q9JKV9	IL20_MOUSE	Interleukin-20 (IL-20) (Cytokine Zcyto10)
Q9JL19	NCOA6_MOUSE	Nuclear receptor coactivator 6 (Activating signal cointegrator 2) (ASC-2) (Amplified in breast cancer protein 3) (Cancer-amplified transcriptional coactivator ASC-2) (Nuclear receptor coactivator RAP250) (NRC) (Nuclear receptor-activating protein, 250 kDa) (Peroxisome proliferator-activated receptor-interacting protein) (PPAR-interacting protein) (Thyroid hormone receptor-binding protein)
Q9JL60	GMEB1_MOUSE	Glucocorticoid modulatory element-binding protein 1 (GMEB-1)
Q9JLF7	TLR5_MOUSE	Toll-like receptor 5

Q9JME2	CHSTB_MOUSE	Carbohydrate sulfotransferase 11 (EC 2.8.2.5) (Chondroitin 4-O-sulfotransferase 1) (Chondroitin 4-sulfotransferase 1) (C4S-1) (C4ST-1) (C4ST1)
Q9JME5	AP3B2_MOUSE	AP-3 complex subunit beta-2 (Adaptor protein complex AP-3 subunit beta-2) (Adaptor-related protein complex 3 subunit beta-2) (Beta-3B-adaptin) (Clathrin assembly protein complex 3 beta-2 large chain)
Q9QUN5	PR3C1_MOUSE	Prolactin-3C1 (Decidualin) (Placental prolactin-like protein J) (PLP-J) (PRL-like protein J) (Prolactin-like protein I) (PLP-I) (PRL-like protein I)
Q9QUP5	HPLN1_MOUSE	Hyaluronan and proteoglycan link protein 1 (Cartilage-linking protein 1) (Cartilage-link protein) (Proteoglycan link protein)
Q9QUR8	SEM7A_MOUSE	Semaphorin-7A (Semaphorin-K1) (Sema K1) (Semaphorin-L) (Sema L) (CD antigen CD108)
Q9QWF0	CAF1A_MOUSE	Chromatin assembly factor 1 subunit A (CAF-1 subunit A) (Chromatin assembly factor I p150 subunit) (CAF-I 150 kDa subunit) (CAF-I p150)
Q9QXT5	EGFL7_MOUSE	Epidermal growth factor-like protein 7 (EGF-like protein 7) (Multiple epidermal growth factor-like domains protein 7) (Multiple EGF-like domains protein 7) (NOTCH4-like protein) (Vascular endothelial statin) (VE-statin) (Zneu1)
Q9QXT8	CSEN_MOUSE	Calsenilin (A-type potassium channel modulatory protein 3) (DRE-antagonist modulator) (DREAM) (Kv channel-interacting protein 3) (KChIP3)
Q9QY93	DCTP1_MOUSE	dCTP pyrophosphatase 1 (EC 3.6.1.12) (Deoxycytidine-triphosphatase 1) (dCTPase 1) (RS21-C6)
Q9QZ67	PPM1D_MOUSE	Protein phosphatase 1D (EC 3.1.3.16) (Protein phosphatase 2C isoform delta) (PP2C-delta) (Protein phosphatase magnesium-dependent 1 delta) (p53-induced protein phosphatase 1)
Q9QZF2	GPC1_MOUSE	Glypican-1 [Cleaved into: Secreted glypican-1]
Q9QZS0	CO4A3_MOUSE	Collagen alpha-3(IV) chain [Cleaved into: Tumstatin]
Q9R098	HGFA_MOUSE	Hepatocyte growth factor activator (HGF activator) (HGFA) (EC 3.4.21.-) [Cleaved into: Hepatocyte growth factor activator short chain; Hepatocyte growth factor activator long chain]
Q9R0E2	PLOD1_MOUSE	Procollagen-lysine,2-oxoglutarate 5-dioxygenase 1 (EC 1.14.11.4) (Lysyl hydroxylase 1) (LH1)

Q9R0M1	XCR1_MOUSE	Chemokine XC receptor 1 (Lymphotactin receptor) (SCM1 receptor) (XC chemokine receptor 1) (mXCR1)
Q9R0P6	SC11A_MOUSE	Signal peptidase complex catalytic subunit SEC11A (EC 3.4.21.89) (Endopeptidase SP18) (Microsomal signal peptidase 18 kDa subunit) (SPase 18 kDa subunit) (SEC11 homolog A) (SEC11-like protein 1) (SPC18) (Sid 2895)
Q9R0Q3	TMED2_MOUSE	Transmembrane emp24 domain-containing protein 2 (COPI-coated vesicle membrane protein p24) (Membrane protein p24A) (Sid 394) (p24 family protein beta-1) (p24beta1)
Q9R0R1	TRFM_MOUSE	Melanotransferrin (Membrane-bound transferrin-like protein p97) (MTf) (CD antigen CD228)
Q9R0Z9	RHG07_MOUSE	Rho GTPase-activating protein 7 (Deleted in liver cancer 1 protein homolog) (DLC-1) (Rho-type GTPase-activating protein 7) (START domain-containing protein 12) (StARD12) (StAR-related lipid transfer protein 12)
Q9R100	CAD17_MOUSE	Cadherin-17 (BILL-cadherin) (Liver-intestine cadherin) (LI-cadherin) (P130)
Q9R158	AD26A_MOUSE	Disintegrin and metalloproteinase domain-containing protein 26A (ADAM 26A) (EC 3.4.24.-) (Testase-3)
Q9R1C7	PR40A_MOUSE	Pre-mRNA-processing factor 40 homolog A (Formin-binding protein 11) (FBP-11) (Formin-binding protein 3)
Q9R1E6	ENPP2_MOUSE	Ectonucleotide pyrophosphatase/phosphodiesterase family member 2 (E-NPP 2) (EC 3.1.4.39) (Autotaxin) (Extracellular lysophospholipase D) (LysoPLD)
Q9R1S7	MRP6_MOUSE	Multidrug resistance-associated protein 6 (ATP-binding cassette sub-family C member 6)
Q9R207	NBN_MOUSE	Nibrin (Cell cycle regulatory protein p95) (Nijmegen breakage syndrome protein 1 homolog)
Q9R269	PEPL_MOUSE	Periplakin
Q9WTK3	GPAA1_MOUSE	Glycosylphosphatidylinositol anchor attachment 1 protein (GPI anchor attachment protein 1) (GAA1 protein homolog) (mGAA1)
Q9WTM4	NF2L3_MOUSE	Nuclear factor erythroid 2-related factor 3 (NF-E2-related factor 3) (NFE2-related factor 3) (Nuclear factor, erythroid derived 2, like 3)
Q9WTP7	KAD3_MOUSE	GTP:AMP phosphotransferase AK3, mitochondrial (EC 2.7.4.10) (Adenylate kinase 3) (AK 3) (Adenylate kinase 3 alpha-like 1)

Q9WTR5	CAD13_MOUSE	Cadherin-13 (Heart cadherin) (H-cadherin) (Truncated cadherin) (T-cad) (T-cadherin)
Q9WU02	V1BR_MOUSE	Vasopressin V1b receptor (V1bR) (AVPR V1b) (AVPR V3) (Antidiuretic hormone receptor 1b) (Vasopressin V3 receptor)
Q9WU20	MTHR_MOUSE	Methylenetetrahydrofolate reductase (EC 1.5.1.20)
Q9WU72	TN13B_MOUSE	Tumor necrosis factor ligand superfamily member 13B (B-cell-activating factor) (BAFF) (CD antigen CD257) [Cleaved into: Tumor necrosis factor ligand superfamily member 13b, membrane form; Tumor necrosis factor ligand superfamily member 13b, soluble form]
Q9WUV0	ORC5_MOUSE	Origin recognition complex subunit 5
Q9WV07	LOXE3_MOUSE	Hydroperoxide isomerase ALOXE3 (EC 5.4.4.7) (Epidermis-type lipoxygenase 3) (Epidermal LOX-3) (e-LOX-3) (eLOX-3) (Hydroperoxy icosatetraenoate dehydratase) (EC 4.2.1.152)
Q9WV85	NDK3_MOUSE	Nucleoside diphosphate kinase 3 (NDK 3) (NDP kinase 3) (EC 2.7.4.6) (DR-nm23) (Nucleoside diphosphate kinase C) (NDPKC) (nm23-M3)
Q9WVH3	FOXO4_MOUSE	Forkhead box protein O4 (Afxh) (Fork head domain transcription factor AFX1)
Q9Z0G2	SRPK3_MOUSE	SRSF protein kinase 3 (EC 2.7.11.1) (Muscle-specific serine kinase 1) (MSSK-1) (Serine/arginine-rich protein-specific kinase 3) (SR-protein-specific kinase 3) (Serine/threonine-protein kinase 23)
Q9Z0H3	SNF5_MOUSE	SWI/SNF-related matrix-associated actin-dependent regulator of chromatin subfamily B member 1 (BRG1-associated factor 47) (BAF47) (Integrase interactor 1 protein) (SNF5 homolog) (mSNF5)
Q9Z0S6	CLD10_MOUSE	Claudin-10
Q9Z0T6	PKDRE_MOUSE	Polycystic kidney disease and receptor for egg jelly-related protein (PKD and REJ homolog)
Q9Z2A5	ATE1_MOUSE	Arginyl-tRNA--protein transferase 1 (Arginyltransferase 1) (R-transferase 1) (EC 2.3.2.8) (Arginine-tRNA--protein transferase 1)
Q9Z2G9	HTAI2_MOUSE	Oxidoreductase HTATIP2 (EC 1.1.1.-)
Q9Z2J6	PD2R2_MOUSE	Prostaglandin D2 receptor 2 (Chemoattractant receptor-homologous molecule expressed on Th2 cells) (G-protein coupled receptor 44) (CD antigen CD294)

Table 3. Proteins unique to stimDCs EXO.

Accession	Entry name	Protein names
A1A535	MELT_MOUSE	Ventricular zone-expressed PH domain-containing protein 1 (Protein melted homolog)
A1IGU4	ARH37_MOUSE	Rho guanine nucleotide exchange factor 37
A2A559	PGAP3_MOUSE	Post-GPI attachment to proteins factor 3 (PER1-like domain-containing protein 1)
A2A5Z6	SMUF2_MOUSE	E3 ubiquitin-protein ligase SMURF2 (EC 2.3.2.26) (HECT-type E3 ubiquitin transferase SMURF2) (SMAD ubiquitination regulatory factor 2) (SMAD-specific E3 ubiquitin-protein ligase 2)
A2A791	ZMYM4_MOUSE	Zinc finger MYM-type protein 4 (Zinc finger protein 262)
A2A8L1	CHD5_MOUSE	Chromodomain-helicase-DNA-binding protein 5 (CHD-5) (EC 3.6.4.12) (ATP-dependent helicase CHD5)
A2A8L5	PTPRF_MOUSE	Receptor-type tyrosine-protein phosphatase F (EC 3.1.3.48) (Leukocyte common antigen related) (LAR)
A2A8U2	TM201_MOUSE	Transmembrane protein 201 (Spindle-associated membrane protein 1)
A2AB59	RHG27_MOUSE	Rho GTPase-activating protein 27 (CIN85-associated multi-domain-containing Rho GTPase-activating protein 1) (Rho-type GTPase-activating protein 27)
A2AGH6	MED12_MOUSE	Mediator of RNA polymerase II transcription subunit 12 (Mediator complex subunit 12) (OPA-containing protein) (Thyroid hormone receptor-associated protein complex 230 kDa component) (Trap230) (Trinucleotide repeat-containing gene 11 protein)
A2AGL3	RYR3_MOUSE	Ryanodine receptor 3 (RYR-3) (RyR3) (Brain ryanodine receptor-calcium release channel) (Brain-type ryanodine receptor) (Type 3 ryanodine receptor)
A2AIV8	CARD9_MOUSE	Caspase recruitment domain-containing protein 9
A2AJA7	AEGP_MOUSE	Apical endosomal glycoprotein (MAM domain-containing protein 4)
A2AJK6	CHD7_MOUSE	Chromodomain-helicase-DNA-binding protein 7 (CHD-7) (EC 3.6.4.12) (ATP-dependent helicase CHD7)
A2AKB9	DCA10_MOUSE	DDB1- and CUL4-associated factor 10 (WD repeat-containing protein 32)

A2AKK5	ACNT1_MOUSE	Acyl-coenzyme A amino acid N-acyltransferase 1 (EC 2.3.1.-)
A2ALI5	AJAP1_MOUSE	Adherens junction-associated protein 1
C0HKD1	F25A2_MOUSE	Protein FAM205A
A2AQ19	RTF1_MOUSE	RNA polymerase-associated protein RTF1 homolog
A2AQP0	MYH7B_MOUSE	Myosin-7B (Myosin cardiac muscle beta chain) (Myosin heavy chain 7B, cardiac muscle beta isoform)
A2ARV4	LRP2_MOUSE	Low-density lipoprotein receptor-related protein 2 (LRP-2) (Glycoprotein 330) (gp330) (Megalin)
A2AS55	ANR16_MOUSE	Ankyrin repeat domain-containing protein 16
A2AS89	SPEB_MOUSE	Agmatinase, mitochondrial (EC 3.5.3.11) (Agmatine ureohydrolase) (AUH)
A2AVA0	SVEP1_MOUSE	Sushi, von Willebrand factor type A, EGF and pentraxin domain-containing protein 1 (Polydom)
A2AVR2	MROH7_MOUSE	Maestro heat-like repeat-containing protein family member 7 (HEAT repeat-containing protein 8)
A2AX52	CO6A4_MOUSE	Collagen alpha-4(VI) chain
A2BE28	LAS1L_MOUSE	Ribosomal biogenesis protein LAS1L (Protein LAS1 homolog)
A2RSQ0	DEN5B_MOUSE	DENN domain-containing protein 5B (Rab6IP1-like protein)
A2RT91	ANKAR_MOUSE	Ankyrin and armadillo repeat-containing protein
A2VDH3	LRC38_MOUSE	Leucine-rich repeat-containing protein 38 (BK channel auxiliary gamma subunit LRRC38)
A3FIN4	AT8B5_MOUSE	Phospholipid-transporting ATPase FetA (EC 7.6.2.1) (ATPase class I type 8B member 2-like protein) (ATPase class I type 8B member 5) (Flippase expressed in testis A)
A3KGK3	FR1L4_MOUSE	Fer-1-like protein 4
A6H6E2	MMRN2_MOUSE	Multimerin-2
A6X942	SH24B_MOUSE	SH2 domain-containing protein 4B
B0F2B4	NLGN4_MOUSE	Neuroligin 4-like (Neuroligin-4) (NL-4)
B1AS29	GRIK3_MOUSE	Glutamate receptor ionotropic, kainate 3 (GluK3) (Glutamate receptor 7) (GluR-7) (GluR7)
B1AWI6	MSMP_MOUSE	Prostate-associated microseminoprotein (PC3-secreted microprotein homolog)
B1AXH1	NHSL2_MOUSE	NHS-like protein 2
B1AY10	NFX1_MOUSE	Transcriptional repressor NF-X1 (m-Nfx.1) (EC 2.3.2.-) (Nuclear transcription factor, X box-binding protein 1)
B2KFW1	ZSC20_MOUSE	Zinc finger and SCAN domain-containing protein 20 (Zinc finger protein 31)
B2RRE4	Z518B_MOUSE	Zinc finger protein 518B

B2RS91	RRN3_MOUSE	RNA polymerase I-specific transcription initiation factor RRN3
B2RU80	PTPRB_MOUSE	Receptor-type tyrosine-protein phosphatase beta (Protein-tyrosine phosphatase beta) (R-PTP-beta) (EC 3.1.3.48) (Vascular endothelial protein tyrosine phosphatase) (VE-PTP)
B2RWS6	EP300_MOUSE	Histone acetyltransferase p300 (p300 HAT) (EC 2.3.1.48) (E1A-associated protein p300) (Histone butyryltransferase p300) (EC 2.3.1.-) (Histone crotonyltransferase p300) (EC 2.3.1.-) (Protein 2-hydroxyisobutyryltransferase p300) (EC 2.3.1.-) (Protein propionyltransferase p300) (EC 2.3.1.-)
B2RX14	TUT4_MOUSE	Terminal uridylyltransferase 4 (TUTase 4) (EC 2.7.7.52) (Zinc finger CCHC domain-containing protein 11)
B9EHT4	CLIP3_MOUSE	CAP-Gly domain-containing linker protein 3 (Cytoplasmic linker protein 170-related 59 kDa protein) (CLIP-170-related 59 kDa protein) (CLIPR-59)
B9EJI9	T229A_MOUSE	Transmembrane protein 229A
C0LLJ0	IMA8_MOUSE	Importin subunit alpha-8 (Karyopherin subunit alpha-7)
C6KI89	CTSG2_MOUSE	Cation channel sperm-associated protein subunit gamma 2
D3YZF7	VS10L_MOUSE	V-set and immunoglobulin domain-containing protein 10-like
E9Q0B3	CC080_MOUSE	Uncharacterized membrane protein C3orf80 homolog
E9Q3S4	M3K19_MOUSE	Mitogen-activated protein kinase kinase kinase 19 (EC 2.7.11.1) (SPS1/STE20-related protein kinase YSK4)
E9Q401	RYR2_MOUSE	Ryanodine receptor 2 (RYR-2) (RyR2) (Cardiac muscle ryanodine receptor) (Cardiac muscle ryanodine receptor-calcium release channel) (Type 2 ryanodine receptor)
E9Q5R7	NAL12_MOUSE	NACHT, LRR and PYD domains-containing protein 12 (Monarch-1) (PYRIN-containing APAF1-like protein 7) (PYPAF7)
E9Q784	ZC3HD_MOUSE	Zinc finger CCCH domain-containing protein 13
E9Q7X6	HEG1_MOUSE	Protein HEG homolog 1
E9Q9F6	CTSRD_MOUSE	Cation channel sperm-associated protein subunit delta (CatSper-delta) (CatSperdelta) (Transmembrane protein 146)
F2Z461	HERC6_MOUSE	E3 ISG15--protein ligase Herc6 (EC 2.3.2.-)

F6W8I0	YJEN3_MOUSE	YjeF N-terminal domain-containing protein 3 (YjeF_N3) (hYjeF_N3)
F6ZDS4	TPR_MOUSE	Nucleoprotein TPR (NPC-associated intranuclear protein) (Translocated promoter region and nuclear basket protein)
F8VQB6	MYO10_MOUSE	Unconventional myosin-X (Unconventional myosin-10)
G3UZ78	ADGB_MOUSE	Androglobin (Calpain-7-like protein)
O08539	BIN1_MOUSE	Myc box-dependent-interacting protein 1 (Amphiphysin II) (Amphiphysin-like protein) (Bridging integrator 1) (SH3 domain-containing protein 9)
O08573	LEG9_MOUSE	Galectin-9 (Gal-9)
O08601	MTP_MOUSE	Microsomal triglyceride transfer protein large subunit
O08641	SH3Y1_MOUSE	SH3 domain-containing YSC84-like protein 1
O08648	M3K4_MOUSE	Mitogen-activated protein kinase kinase kinase 4 (EC 2.7.11.25) (MAPK/ERK kinase kinase 4) (MEK kinase 4) (MEKK 4)
O08663	MAP2_MOUSE	Methionine aminopeptidase 2 (MAP 2) (MetAP 2) (EC 3.4.11.18) (Initiation factor 2-associated 67 kDa glycoprotein) (p67) (p67eIF2) (Peptidase M)
O08739	AMPD3_MOUSE	AMP deaminase 3 (EC 3.5.4.6) (AMP deaminase H-type) (AMP deaminase isoform E) (Heart-type AMPD)
O08756	HCD2_MOUSE	3-hydroxyacyl-CoA dehydrogenase type-2 (EC 1.1.1.35) (17-beta-hydroxysteroid dehydrogenase 10) (17-beta-HSD 10) (EC 1.1.1.51) (3-hydroxy-2-methylbutyryl-CoA dehydrogenase) (EC 1.1.1.178) (3-hydroxyacyl-CoA dehydrogenase type II) (Endoplasmic reticulum-associated amyloid beta-peptide-binding protein) (Mitochondrial ribonuclease P protein 2) (Mitochondrial RNase P protein 2) (Type II HADH)
O08759	UBE3A_MOUSE	Ubiquitin-protein ligase E3A (EC 2.3.2.26) (HECT-type ubiquitin transferase E3A) (Oncogenic protein-associated protein E6-AP)
O08785	CLOCK_MOUSE	Circadian locomoter output cycles protein kaput (mCLOCK) (EC 2.3.1.48)
O08848	RO60_MOUSE	60 kDa SS-A/Ro ribonucleoprotein (60 kDa Ro protein) (60 kDa ribonucleoprotein Ro) (Ro60) (RoRNP) (TROVE domain family member 2)
O08914	FAAH1_MOUSE	Fatty-acid amide hydrolase 1 (EC 3.5.1.99) (Anandamide amidohydrolase 1) (Oleamide hydrolase 1)

O09110	MP2K3_MOUSE	Dual specificity mitogen-activated protein kinase kinase 3 (MAP kinase kinase 3) (MAPKK 3) (EC 2.7.12.2) (MAPK/ERK kinase 3) (MEK 3)
O09167	RL21_MOUSE	60S ribosomal protein L21
O35280	CHK1_MOUSE	Serine/threonine-protein kinase Chk1 (EC 2.7.11.1) (CHK1 checkpoint homolog) (Checkpoint kinase-1)
O35345	IMA7_MOUSE	Importin subunit alpha-7 (Importin alpha-S2) (Karyopherin subunit alpha-6)
O35386	PAHX_MOUSE	Phytanoyl-CoA dioxygenase, peroxisomal (EC 1.14.11.18) (Lupus nephritis-associated peptide 1) (Phytanic acid oxidase) (Phytanoyl-CoA alpha-hydroxylase) (PhyH)
O35493	CLK4_MOUSE	Dual specificity protein kinase CLK4 (EC 2.7.12.1) (CDC-like kinase 4)
O35594	IFT81_MOUSE	Intraflagellar transport protein 81 homolog (Carnitine deficiency-associated protein expressed in ventricle 1) (CDV-1)
O35626	RASD1_MOUSE	Dexamethasone-induced Ras-related protein 1
O35638	STAG2_MOUSE	Cohesin subunit SA-2 (SCC3 homolog 2) (Stromal antigen 2)
O35914	BNC1_MOUSE	Zinc finger protein basonuclin-1
O35954	PITM1_MOUSE	Membrane-associated phosphatidylinositol transfer protein 1 (Drosophila retinal degeneration B homolog 1) (RdgB1) (Mpt-1) (Phosphatidylinositol transfer protein, membrane-associated 1) (PITPnm 1) (Pyk2 N-terminal domain-interacting receptor 2) (NIR-2)
O54784	DAPK3_MOUSE	Death-associated protein kinase 3 (DAP kinase 3) (EC 2.7.11.1) (DAP-like kinase) (Dlk) (MYPT1 kinase) (ZIP-kinase)
O54794	AQP7_MOUSE	Aquaporin-7 (AQP-7) (Aquaglyceroporin-7)
O54824	IL16_MOUSE	Pro-interleukin-16 [Cleaved into: Interleukin-16 (IL-16) (Lymphocyte chemoattractant factor) (LCF)]
O54825	BYST_MOUSE	Bystin
O54910	IKBE_MOUSE	NF-kappa-B inhibitor epsilon (NF-kappa-BIE) (I-kappa-B-epsilon) (IkB-E) (IkB-epsilon) (IkappaBepsilon)
O54928	SOCS5_MOUSE	Suppressor of cytokine signaling 5 (SOCS-5) (Cytokine-inducible SH2-containing protein 5)
O54940	BNIP2_MOUSE	BCL2/adenovirus E1B 19 kDa protein-interacting protein 2
O55013	TPPC3_MOUSE	Trafficking protein particle complex subunit 3 (BET3 homolog)

O55106	STRN_MOUSE	Striatin
O55225	OTOG_MOUSE	Otogelin
O55233	CER1_MOUSE	Cerberus (Cerberus-like protein) (Cer-l) (Cerberus-related protein)
O55234	PSB5_MOUSE	Proteasome subunit beta type-5 (EC 3.4.25.1) (Macropain epsilon chain) (Multicatalytic endopeptidase complex epsilon chain) (Proteasome chain 6) (Proteasome epsilon chain) (Proteasome subunit X)
O70279	ESS2_MOUSE	Splicing factor ESS-2 homolog (ES2 protein) (Expressed sequence 2 embryonic lethal)
O70305	ATX2_MOUSE	Ataxin-2 (Spinocerebellar ataxia type 2 protein homolog)
O70373	XIRP1_MOUSE	Xin actin-binding repeat-containing protein 1 (Cardiomyopathy-associated protein 1)
O70400	PDLI1_MOUSE	PDZ and LIM domain protein 1 (C-terminal LIM domain protein 1) (Elfin) (LIM domain protein CLP-36)
O70422	TF2H4_MOUSE	General transcription factor IIH subunit 4 (Basic transcription factor 2 52 kDa subunit) (BTF2 p52) (General transcription factor IIH polypeptide 4) (TFIIH basal transcription factor complex p52 subunit)
O70458	OSMR_MOUSE	Oncostatin-M-specific receptor subunit beta (Interleukin-31 receptor subunit beta) (IL-31 receptor subunit beta) (IL-31R subunit beta) (IL-31R-beta) (IL-31RB)
O70469	DOK2_MOUSE	Docking protein 2 (Dok-related protein) (Dok-R) (Downstream of tyrosine kinase 2) (IL-four receptor-interacting protein) (FRIP) (p56(dok-2))
O70479	BACD2_MOUSE	BTB/POZ domain-containing adapter for CUL3-mediated RhoA degradation protein 2 (BTB/POZ domain-containing protein TNFAIP1) (Tumor necrosis factor, alpha-induced protein 1, endothelial)
O70491	STRA6_MOUSE	Receptor for retinol uptake STRA6 (Retinoic acid-responsive protein) (Retinol-binding protein receptor STRA6) (Stimulated by retinoic acid gene 6 protein)
O70497	FCN2_MOUSE	Ficolin-2 (Collagen/fibrinogen domain-containing protein 2) (Ficolin-B) (Ficolin-beta) (L-ficolin)
O70546	KDM6A_MOUSE	Lysine-specific demethylase 6A (EC 1.14.11.-) (Histone demethylase UTX) (Ubiquitously transcribed TPR protein on the X chromosome) (Ubiquitously transcribed X chromosome tetratricopeptide repeat protein)

O70576	STAG3_MOUSE	Cohesin subunit SA-3 (SCC3 homolog 3) (Stromal antigen 3) (Stromalin-3)
O70585	DTNB_MOUSE	Dystrobrevin beta (DTN-B) (mDTN-B) (Beta-dystrobrevin)
O70624	MYOC_MOUSE	Myocilin (Trabecular meshwork-induced glucocorticoid response protein) [Cleaved into: Myocilin, N-terminal fragment (Myocilin 20 kDa N-terminal fragment); Myocilin, C-terminal fragment (Myocilin 35 kDa N-terminal fragment)]
O88207	CO5A1_MOUSE	Collagen alpha-1(V) chain
O88444	ADCY1_MOUSE	Adenylate cyclase type 1 (EC 4.6.1.1) (ATP pyrophosphate-lyase 1) (Adenylate cyclase type I) (Adenylyl cyclase 1) (Ca(2+)/calmodulin-activated adenylyl cyclase)
O88492	PLIN4_MOUSE	Perilipin-4 (Adipocyte protein S3-12)
O88495	MTR1L_MOUSE	Melatonin-related receptor (G protein-coupled receptor 50) (H9)
O88536	FPR2_MOUSE	Formyl peptide receptor 2 (Formylpeptide receptor-related sequence 2) (Lipoxin A4 receptor-like protein) (N-formylpeptide receptor-like 2)
O88543	CSN3_MOUSE	COP9 signalosome complex subunit 3 (SGN3) (Signalosome subunit 3) (JAB1-containing signalosome subunit 3)
O88700	BLM_MOUSE	Bloom syndrome protein homolog (mBLM) (EC 3.6.4.12) (RecQ helicase homolog)
O88799	ZAN_MOUSE	Zonadhesin
O88833	CP4AA_MOUSE	Cytochrome P450 4A10 (CYPIVA10) (Cytochrome P450-LA-omega 1) (Cytochrome P452) (Lauric acid omega-hydroxylase) (Long-chain fatty acid omega-monooxygenase) (EC 1.14.14.80)
O88967	YMEL1_MOUSE	ATP-dependent zinc metalloprotease YME1L1 (EC 3.4.24.-) (ATP-dependent metalloprotease FtsH1) (YME1-like protein 1)
O89094	CASPE_MOUSE	Caspase-14 (CASP-14) (EC 3.4.22.-) (Mini-ICE) (MICE) [Cleaved into: Caspase-14 subunit p17, mature form; Caspase-14 subunit p10, mature form; Caspase-14 subunit p20, intermediate form; Caspase-14 subunit p8, intermediate form]
P01582	IL1A_MOUSE	Interleukin-1 alpha (IL-1 alpha)
P01729	LV2B_MOUSE	Ig lambda-2 chain V region MOPC 315
P01759	HVM15_MOUSE	Ig heavy chain V region BCL1
P01783	HVM16_MOUSE	Deleted.
P01821	HVM45_MOUSE	Ig heavy chain V region MC101

P02469	LAMB1_MOUSE	Laminin subunit beta-1 (Laminin B1 chain) (Laminin-1 subunit beta) (Laminin-10 subunit beta) (Laminin-12 subunit beta) (Laminin-2 subunit beta) (Laminin-6 subunit beta) (Laminin-8 subunit beta)
P03966	MYCN_MOUSE	N-myc proto-oncogene protein
P03978	TVC2_MOUSE	T-cell receptor gamma chain V region V108B (Fragment)
P03985	TCC2_MOUSE	T-cell receptor gamma chain C region C7.5
P04919	B3AT_MOUSE	Band 3 anion transport protein (Anion exchange protein 1) (AE 1) (Anion exchanger 1) (MEB3) (Solute carrier family 4 member 1) (CD antigen CD233)
P05533	LY6A_MOUSE	Lymphocyte antigen 6A-2/6E-1 (Ly-6A.2/Ly-6E.1) (Stem cell antigen 1) (SCA-1) (T-cell-activating protein) (TAP)
P06799	IFNA7_MOUSE	Interferon alpha-7 (IFN-alpha-7)
P06804	TNFA_MOUSE	Tumor necrosis factor (Cachectin) (TNF-alpha) (Tumor necrosis factor ligand superfamily member 2) (TNF-a) [Cleaved into: Tumor necrosis factor, membrane form (N-terminal fragment) (NTF); Intracellular domain 1 (ICD1); Intracellular domain 2 (ICD2); C-domain 1; C-domain 2; Tumor necrosis factor, soluble form]
P08074	CBR2_MOUSE	Carbonyl reductase [NADPH] 2 (EC 1.1.1.184) (Adipocyte protein P27) (AP27) (Lung carbonyl reductase) (LCR) (NADPH-dependent carbonyl reductase 2)
P08399	PHXR5_MOUSE	Putative per-hexamer repeat protein 5
P08414	KCC4_MOUSE	Calcium/calmodulin-dependent protein kinase type IV (CaMK IV) (EC 2.7.11.17) (CaM kinase-GR)
P08505	IL6_MOUSE	Interleukin-6 (IL-6) (B-cell hybridoma growth factor) (Interleukin HP-1)
P09671	SODM_MOUSE	Superoxide dismutase [Mn], mitochondrial (EC 1.15.1.1)
P0C0A3	CHMP6_MOUSE	Charged multivesicular body protein 6 (Chromatin-modifying protein 6)
P0C242	CCNO_MOUSE	Cyclin-O
P10417	BCL2_MOUSE	Apoptosis regulator Bcl-2
P10749	IL1B_MOUSE	Interleukin-1 beta (IL-1 beta)
P10810	CD14_MOUSE	Monocyte differentiation antigen CD14 (Myeloid cell-specific leucine-rich glycoprotein) (CD antigen CD14)
P10855	CCL3_MOUSE	C-C motif chemokine 3 (Heparin-binding chemotaxis protein) (L2G25B) (Macrophage

		inflammatory protein 1-alpha) (MIP-1-alpha) (SIS-alpha) (Small-inducible cytokine A3) (TY-5)
P10925	ZFY1_MOUSE	Zinc finger Y-chromosomal protein 1
P11103	PARP1_MOUSE	Poly [ADP-ribose] polymerase 1 (PARP-1) (EC 2.4.2.30) (ADP-ribosyltransferase diphtheria toxin-like 1) (ARTD1) (DNA ADP-ribosyltransferase PARP1) (EC 2.4.2.-) (NAD(+) ADP-ribosyltransferase 1) (ADPRT 1) (Poly[ADP-ribose] synthase 1) (msPARP) (Protein poly-ADP-ribosyltransferase PARP1) (EC 2.4.2.-)
P11610	CD1D2_MOUSE	Antigen-presenting glycoprotein CD1d2 (CD antigen CD1d.2)
P11859	ANGT_MOUSE	Angiotensinogen (Serpin A8) [Cleaved into: Angiotensin-1 (Angiotensin 1-10) (Angiotensin I) (Ang I); Angiotensin-2 (Angiotensin 1-8) (Angiotensin II) (Ang II); Angiotensin-3 (Angiotensin 2-8) (Angiotensin III) (Ang III) (Des-Asp[1]-angiotensin II); Angiotensin-4 (Angiotensin 3-8) (Angiotensin IV) (Ang IV); Angiotensin 1-9; Angiotensin 1-7; Angiotensin 1-5; Angiotensin 1-4]
P12242	UCP1_MOUSE	Mitochondrial brown fat uncoupling protein 1 (UCP 1) (Solute carrier family 25 member 7) (Thermogenin)
P14753	EPOR_MOUSE	Erythropoietin receptor (EPO-R)
P14847	CRP_MOUSE	C-reactive protein
P14873	MAP1B_MOUSE	Microtubule-associated protein 1B (MAP-1B) (MAP1(X)) (MAP1.2) [Cleaved into: MAP1B heavy chain; MAP1 light chain LC1]
P14901	HMOX1_MOUSE	Heme oxygenase 1 (HO-1) (EC 1.14.14.18) (P32 protein)
P15066	JUND_MOUSE	Transcription factor jun-D
P15209	NTRK2_MOUSE	BDNF/NT-3 growth factors receptor (EC 2.7.10.1) (GP145-TrkB/GP95-TrkB) (Trk-B) (Neurotrophic tyrosine kinase receptor type 2) (TrkB tyrosine kinase)
P16388	KCNA1_MOUSE	Potassium voltage-gated channel subfamily A member 1 (MBK1) (MKI) (Voltage-gated potassium channel subunit Kv1.1)
P20033	PDGFA_MOUSE	Platelet-derived growth factor subunit A (PDGF subunit A) (PDGF-1) (Platelet-derived growth factor A chain) (Platelet-derived growth factor alpha polypeptide)
P20917	MAG_MOUSE	Myelin-associated glycoprotein (Siglec-4a)
P21661	NEC2_MOUSE	Neuroendocrine convertase 2 (NEC 2) (EC 3.4.21.94) (KEX2-like endoprotease 2)

		(Prohormone convertase 2) (Proprotein convertase 2) (PC2)
P22366	MYD88_MOUSE	Myeloid differentiation primary response protein MyD88
P22599	A1AT2_MOUSE	Alpha-1-antitrypsin 1-2 (AAT) (Alpha-1 protease inhibitor 2) (Alpha-1-antiproteinase) (Serine protease inhibitor 1-2) (Serine protease inhibitor A1b) (Serpin A1b)
P22907	HEM3_MOUSE	Porphobilinogen deaminase (PBG-D) (EC 2.5.1.61) (Hydroxymethylbilane synthase) (HMBS) (Pre-uroporphyrinogen synthase)
P23198	CBX3_MOUSE	Chromobox protein homolog 3 (Heterochromatin protein 1 homolog gamma) (HP1 gamma) (M32) (Modifier 2 protein)
P23275	OLF15_MOUSE	Olfactory receptor 15 (Odorant receptor OR3) (Olfactory receptor 256-17)
P23804	MDM2_MOUSE	E3 ubiquitin-protein ligase Mdm2 (EC 2.3.2.27) (Double minute 2 protein) (Oncoprotein Mdm2) (RING-type E3 ubiquitin transferase Mdm2) (p53-binding protein Mdm2)
P23819	GRIA2_MOUSE	Glutamate receptor 2 (GluR-2) (AMPA-selective glutamate receptor 2) (GluR-B) (GluR-K2) (Glutamate receptor ionotropic, AMPA 2) (GluA2)
P24699	MYF5_MOUSE	Myogenic factor 5 (Myf-5)
P24823	PPBN_MOUSE	Alkaline phosphatase, germ cell type (EC 3.1.3.1) (Alkaline phosphatase 5) (Alkaline phosphatase, placental-like) (Embryonic alkaline phosphatase) (EAP) (Embryonic-type alkaline phosphatase)
P25446	TNR6_MOUSE	Tumor necrosis factor receptor superfamily member 6 (Apo-1 antigen) (Apoptosis-mediating surface antigen FAS) (FASLG receptor) (CD antigen CD95)
P26883	FKB1A_MOUSE	Peptidyl-prolyl cis-trans isomerase FKBP1A (PPIase FKBP1A) (EC 5.2.1.8) (12 kDa FK506-binding protein) (12 kDa FKBP) (FKBP-12) (Calstabin-1) (FK506-binding protein 1A) (FKBP-1A) (Immunophilin FKBP12) (Rotamase)
P27546	MAP4_MOUSE	Microtubule-associated protein 4 (MAP-4)
P28235	CXA4_MOUSE	Gap junction alpha-4 protein (Connexin-37) (Cx37)
P28776	I23O1_MOUSE	Indoleamine 2,3-dioxygenase 1 (IDO-1) (EC 1.13.11.52) (Indoleamine-pyrrole 2,3-dioxygenase)
P29352	PTN22_MOUSE	Tyrosine-protein phosphatase non-receptor type 22 (EC 3.1.3.48) (Hematopoietic cell protein-tyrosine phosphatase 70Z-PEP) (PEST-domain phosphatase) (PEP)

P30882	CCL5_MOUSE	C-C motif chemokine 5 (MuRantes) (SIS-delta) (Small-inducible cytokine A5) (T-cell-specific protein RANTES)
P31651	S6A12_MOUSE	Sodium- and chloride-dependent betaine transporter (Na(+)/Cl(-) betaine/GABA transporter) (Sodium- and chloride-dependent GABA transporter 2) (GAT-2) (Solute carrier family 6 member 12)
P32240	PE2R4_MOUSE	Prostaglandin E2 receptor EP4 subtype (PGE receptor EP4 subtype) (PGE2 receptor EP4 subtype) (Prostanoid EP4 receptor)
P32507	NECT2_MOUSE	Nectin-2 (Herpes virus entry mediator B) (Herpesvirus entry mediator B) (HveB) (Murine herpes virus entry protein B) (mHveB) (Nectin cell adhesion molecule 2) (Poliovirus receptor homolog) (Poliovirus receptor-related protein 2) (CD antigen CD112)
P33174	KIF4_MOUSE	Chromosome-associated kinesin KIF4 (Chromokinesin)
P33242	STF1_MOUSE	Steroidogenic factor 1 (SF-1) (STF-1) (Adrenal 4-binding protein) (Embryonal LTR-binding protein) (ELP) (Embryonal long terminal repeat-binding protein) (Fushi tarazu factor homolog 1) (Nuclear receptor subfamily 5 group A member 1) (Steroid hormone receptor Ad4BP) (Steroid hydroxylase positive regulator)
P34914	HYES_MOUSE	Bifunctional epoxide hydrolase 2 [Includes: Cytosolic epoxide hydrolase 2 (CEH) (EC 3.3.2.10) (Epoxide hydratase) (Soluble epoxide hydrolase) (SEH); Lipid-phosphate phosphatase (EC 3.1.3.76)]
P34927	ASGR1_MOUSE	Asialoglycoprotein receptor 1 (ASGP-R 1) (ASGPR 1) (Hepatic lectin 1) (HL-1) (mHL-1)
P35285	RB22A_MOUSE	Ras-related protein Rab-22A (Rab-22) (Rab-14)
P35459	LY6D_MOUSE	Lymphocyte antigen 6D (Ly-6D) (Thymocyte B-cell antigen) (ThB)
P35582	FOXA1_MOUSE	Hepatocyte nuclear factor 3-alpha (HNF-3-alpha) (HNF-3A) (Forkhead box protein A1)
P35831	PTN12_MOUSE	Tyrosine-protein phosphatase non-receptor type 12 (EC 3.1.3.48) (MPTP-PEST) (Protein-tyrosine phosphatase P19) (P19-PTP)
P35991	BTK_MOUSE	Tyrosine-protein kinase BTK (EC 2.7.10.2) (Agammaglobulinemia tyrosine kinase) (ATK) (B-cell progenitor kinase) (BPK) (Bruton tyrosine kinase) (Kinase EMB)
P38060	HMGCL_MOUSE	Hydroxymethylglutaryl-CoA lyase, mitochondrial (HL) (HMG-CoA lyase) (EC 4.1.3.4) (3-hydroxy-3-methylglutarate-CoA lyase)

P40645	SOX6_MOUSE	Transcription factor SOX-6 (SOX-LZ)
P40694	SMBP2_MOUSE	DNA-binding protein SMUBP-2 (EC 3.6.4.12) (EC 3.6.4.13) (ATP-dependent helicase IGHMBP2) (Cardiac transcription factor 1) (CATF1) (Immunoglobulin mu-binding protein 2)
P41216	ACSL1_MOUSE	Long-chain-fatty-acid--CoA ligase 1 (EC 6.2.1.3) (Arachidonate--CoA ligase) (EC 6.2.1.15) (Long-chain acyl-CoA synthetase 1) (LACS 1) (Phytanate--CoA ligase) (EC 6.2.1.24)
P41242	MATK_MOUSE	Megakaryocyte-associated tyrosine-protein kinase (EC 2.7.10.2) (Protein kinase NTK) (Tyrosine-protein kinase CTK)
P42125	ECI1_MOUSE	Enoyl-CoA delta isomerase 1, mitochondrial (EC 5.3.3.8) (3,2-trans-enoyl-CoA isomerase) (Delta(3),Delta(2)-enoyl-CoA isomerase) (D3,D2-enoyl-CoA isomerase) (Dodecenoyl-CoA isomerase)
P42581	HMX3_MOUSE	Homeobox protein HMX3 (Homeobox protein H6 family member 3) (Homeobox protein Nkx-5.1)
P43247	MSH2_MOUSE	DNA mismatch repair protein Msh2 (MutS protein homolog 2)
P47713	PA24A_MOUSE	Cytosolic phospholipase A2 (cPLA2) (Phospholipase A2 group IVA) [Includes: Phospholipase A2 (EC 3.1.1.4) (Phosphatidylcholine 2-acylhydrolase); Lysophospholipase (EC 3.1.1.5)]
P47739	AL3A1_MOUSE	Aldehyde dehydrogenase, dimeric NADP-preferring (EC 1.2.1.5) (Aldehyde dehydrogenase 4) (Aldehyde dehydrogenase family 3 member A1) (Dioxin-inducible aldehyde dehydrogenase 3)
P47879	IBP4_MOUSE	Insulin-like growth factor-binding protein 4 (IBP-4) (IGF-binding protein 4) (IGFBP-4)
P47880	IBP6_MOUSE	Insulin-like growth factor-binding protein 6 (IBP-6) (IGF-binding protein 6) (IGFBP-6)
P48757	GAST_MOUSE	Gastrin [Cleaved into: Gastrin-71 (G71); Big gastrin (Gastrin-34) (G34); Gastrin]
P48760	FOLC_MOUSE	Folylpolyglutamate synthase, mitochondrial (EC 6.3.2.17) (Folylpoly-gamma-glutamate synthetase) (FPGS) (Tetrahydrofolylpolyglutamate synthase) (Tetrahydrofolate synthase)
P49194	RET3_MOUSE	Retinol-binding protein 3 (Interphotoreceptor retinoid-binding protein) (IRBP) (Interstitial retinol-binding protein)
P50428	ARSA_MOUSE	Arylsulfatase A (ASA) (EC 3.1.6.8) (Cerebroside-sulfatase)
P51125	ICAL_MOUSE	Calpastatin (Calpain inhibitor)

P51491	OPSB_MOUSE	Short-wave-sensitive opsin 1 (S opsin) (Blue cone photoreceptor pigment) (Blue-sensitive opsin) (BOP) (Short wavelength-sensitive cone opsin)
P51807	DYLT1_MOUSE	Dynein light chain Tctex-type 1 (Activator of G-protein signaling 2) (AGS2) (T-complex testis-specific protein 1) (TCTEX-1)
P51855	GSHB_MOUSE	Glutathione synthetase (GSH synthetase) (GSH-S) (EC 6.3.2.3) (Glutathione synthase)
P52332	JAK1_MOUSE	Tyrosine-protein kinase JAK1 (EC 2.7.10.2) (Janus kinase 1) (JAK-1)
P52633	STAT6_MOUSE	Signal transducer and transcription activator 6
P53784	SOX3_MOUSE	Transcription factor SOX-3
P54751	SIA4A_MOUSE	CMP-N-acetylneuraminate-beta-galactosamide-alpha-2,3-sialyltransferase 1 (Alpha 2,3-ST 1) (Beta-galactoside alpha-2,3-sialyltransferase 1) (EC 2.4.99.4) (Gal-NAc6S) (Gal-beta-1,3-GalNAc-alpha-2,3-sialyltransferase) (ST3Gal I) (ST3GalI) (ST3GalA.1) (ST3O) (Sialyltransferase 4A) (SIAT4-A)
P55098	PEX2_MOUSE	Peroxisome biogenesis factor 2 (Peroxin-2) (Peroxisomal membrane protein 3) (Peroxisome assembly factor 1) (PAF-1)
P55264	ADK_MOUSE	Adenosine kinase (AK) (EC 2.7.1.20) (Adenosine 5'-phosphotransferase)
P55288	CAD11_MOUSE	Cadherin-11 (OSF-4) (Osteoblast cadherin) (OB-cadherin)
P56380	AP4A_MOUSE	Bis(5'-nucleosyl)-tetraphosphatase [asymmetrical] (EC 3.6.1.17) (Diadenosine 5',5'''-P1,P4-tetraphosphate asymmetrical hydrolase) (Ap4A hydrolase) (Ap4Aase) (Diadenosine tetraphosphatase) (Nucleoside diphosphate-linked moiety X motif 2) (Nudix motif 2)
P56450	MC4R_MOUSE	Melanocortin receptor 4 (MC4-R)
P56476	GBRR2_MOUSE	Gamma-aminobutyric acid receptor subunit rho-2 (GABA(A) receptor subunit rho-2) (GABA(C) receptor)
P56546	CTBP2_MOUSE	C-terminal-binding protein 2 (CtBP2)
P56593	CP2AC_MOUSE	Cytochrome P450 2A12 (EC 1.14.14.1) (CYPIIA12) (Steroid hormones 7-alpha-hydroxylase) (Testosterone 7-alpha-hydroxylase)
P58044	IDI1_MOUSE	Isopentenyl-diphosphate Delta-isomerase 1 (EC 5.3.3.2) (Isopentenyl pyrophosphate isomerase 1) (IPP isomerase 1) (IPPI1)

P58459	ATS10_MOUSE	A disintegrin and metalloproteinase with thrombospondin motifs 10 (ADAM-TS 10) (ADAM-TS10) (ADAMTS-10) (EC 3.4.24.-)
P58659	EVA1C_MOUSE	Protein eva-1 homolog C (Protein FAM176C)
P58871	TB182_MOUSE	182 kDa tankyrase-1-binding protein
P59017	B2L13_MOUSE	Bcl-2-like protein 13 (Bcl2-L-13) (Bcl-rambo) (Protein Mil1)
P59328	WDHD1_MOUSE	WD repeat and HMG-box DNA-binding protein 1 (Acidic nucleoplasmic DNA-binding protein 1) (And-1)
P59509	ATS19_MOUSE	A disintegrin and metalloproteinase with thrombospondin motifs 19 (ADAM-TS 19) (ADAM-TS19) (ADAMTS-19) (EC 3.4.24.-)
P59511	ATS20_MOUSE	A disintegrin and metalloproteinase with thrombospondin motifs 20 (ADAM-TS 20) (ADAM-TS20) (ADAMTS-20) (EC 3.4.24.-)
P59530	TA2R7_MOUSE	Taste receptor type 2 member 7 (T2R7) (STC7-4) (T2R30) (T2R6) (mT2R42)
P59672	ANS1A_MOUSE	Ankyrin repeat and SAM domain-containing protein 1A (Odin)
P59764	DOCK4_MOUSE	Dedicator of cytokinesis protein 4
P59997	KDM2A_MOUSE	Lysine-specific demethylase 2A (EC 1.14.11.27) (F-box and leucine-rich repeat protein 11) (F-box/LRR-repeat protein 11) (JmjC domain-containing histone demethylation protein 1A) ([Histone-H3]-lysine-36 demethylase 1A)
P61080	UB2D1_MOUSE	Ubiquitin-conjugating enzyme E2 D1 (EC 2.3.2.23) ((E3-independent) E2 ubiquitin-conjugating enzyme D1) (EC 2.3.2.24) (E2 ubiquitin-conjugating enzyme D1) (Ubiquitin carrier protein D1) (Ubiquitin-conjugating enzyme E2(17)KB 1) (Ubiquitin-conjugating enzyme E2-17 kDa 1) (Ubiquitin-protein ligase D1)
P61202	CSN2_MOUSE	COP9 signalosome complex subunit 2 (SGN2) (Signalosome subunit 2) (Alien homolog) (JAB1-containing signalosome subunit 2) (Thyroid receptor-interacting protein 15) (TR-interacting protein 15) (TRIP-15)
P61255	RL26_MOUSE	60S ribosomal protein L26 (Silica-induced gene 20 protein) (SIG-20)
P61793	LPAR1_MOUSE	Lysophosphatidic acid receptor 1 (LPA receptor 1) (LPA-1) (Lysophosphatidic acid receptor Edg-2) (Rec1.3) (VZG-1)
P62077	TIM8B_MOUSE	Mitochondrial import inner membrane translocase subunit Tim8 B (Deafness dystonia protein 2 homolog)

P62311	LSM3_MOUSE	U6 snRNA-associated Sm-like protein LSm3
P62761	VISL1_MOUSE	Visinin-like protein 1 (VILIP) (Neural visinin-like protein 1) (NVL-1) (NVP-1)
P62862	RS30_MOUSE	40S ribosomal protein S30
P63056	NOE3_MOUSE	Noelin-3 (Olfactomedin-3) (Optimedin)
P63080	GBRB3_MOUSE	Gamma-aminobutyric acid receptor subunit beta-3 (GABA(A) receptor subunit beta-3)
P63213	GBG2_MOUSE	Guanine nucleotide-binding protein G(I)/G(S)/G(O) subunit gamma-2 (G gamma-I)
P70178	SIX5_MOUSE	Homeobox protein SIX5 (DM locus-associated homeodomain protein homolog) (Sine oculis homeobox homolog 5)
P70211	DCC_MOUSE	Netrin receptor DCC (Tumor suppressor protein DCC)
P70295	AUP1_MOUSE	Ancient ubiquitous protein 1
P70298	CUX2_MOUSE	Homeobox protein cut-like 2 (Homeobox protein Cux-2)
P70303	PYRG2_MOUSE	CTP synthase 2 (EC 6.3.4.2) (CTP synthetase 2) (CTPsH) (UTP--ammonia ligase 2)
P70312	HYAS2_MOUSE	Hyaluronan synthase 2 (EC 2.4.1.212) (Hyaluronate synthase 2) (Hyaluronic acid synthase 2) (HA synthase 2)
P70326	TBX5_MOUSE	T-box transcription factor TBX5 (T-box protein 5)
P70336	ROCK2_MOUSE	Rho-associated protein kinase 2 (EC 2.7.11.1) (Rho-associated, coiled-coil-containing protein kinase 2) (Rho-associated, coiled-coil-containing protein kinase II) (ROCK-II) (p164 ROCK-2)
P70387	HFE_MOUSE	Hereditary hemochromatosis protein homolog
P70407	CADH9_MOUSE	Cadherin-9 (T1-cadherin)
P70414	NAC1_MOUSE	Sodium/calcium exchanger 1 (Na(+)/Ca(2+)-exchange protein 1) (Solute carrier family 8 member 1)
P70423	CTR3_MOUSE	Cationic amino acid transporter 3 (CAT-3) (CAT3) (Cationic amino acid transporter y+) (Solute carrier family 7 member 3)
P70426	RIT1_MOUSE	GTP-binding protein Rit1 (Ras-like protein expressed in many tissues) (Ras-like without CAAX protein 1)
P70428	EXT2_MOUSE	Exostosin-2 (EC 2.4.1.224) (EC 2.4.1.225) (Glucuronosyl-N-acetylglucosaminyl-proteoglycan/N-acetylglucosaminyl-proteoglycan 4-alpha-N-acetylglucosaminyltransferase) (Multiple exostoses protein 2 homolog)
P70451	FER_MOUSE	Tyrosine-protein kinase Fer (EC 2.7.10.2) (Proto-oncogene c-Fer) (p94-Fer)

P80560	PTPR2_MOUSE	Receptor-type tyrosine-protein phosphatase N2 (R-PTP-N2) (EC 3.1.3.-) (EC 3.1.3.48) (PTP IA-2beta) (Phogrin) (Protein tyrosine phosphatase-NP) (PTP-NP) [Cleaved into: IA-2beta71; IA-2beta64; IA-2beta60]
P81122	IRS2_MOUSE	Insulin receptor substrate 2 (IRS-2) (4PS)
P82343	RENBP_MOUSE	N-acylglucosamine 2-epimerase (AGE) (EC 5.1.3.8) (GlcNAc 2-epimerase) (N-acetyl-D-glucosamine 2-epimerase) (Renin-binding protein) (RnBP)
P97310	MCM2_MOUSE	DNA replication licensing factor MCM2 (EC 3.6.4.12) (Minichromosome maintenance protein 2 homolog) (Nuclear protein BM28)
P97313	PRKDC_MOUSE	DNA-dependent protein kinase catalytic subunit (DNA-PK catalytic subunit) (DNA-PKcs) (EC 2.7.11.1) (p460)
P97347	RPTN_MOUSE	Repetin
P97360	ETV6_MOUSE	Transcription factor ETV6 (ETS translocation variant 6) (ETS-related protein Tel1) (Tel)
P97379	G3BP2_MOUSE	Ras GTPase-activating protein-binding protein 2 (G3BP-2) (GAP SH3 domain-binding protein 2)
P97402	GCNT2_MOUSE	N-acetyllactosaminide beta-1,6-N-acetylglucosaminyl-transferase (N-acetylglucosaminyltransferase) (EC 2.4.1.150) (I-branching enzyme) (IGNT) (Large I antigen-forming beta-1,6-N-acetylglucosaminyltransferase)
P97433	ARG28_MOUSE	Rho guanine nucleotide exchange factor 28 (190 kDa guanine nucleotide exchange factor) (p190-RhoGEF) (p190RhoGEF) (Rho guanine nucleotide exchange factor) (Rho-interacting protein 2)
P97459	NPAS1_MOUSE	Neuronal PAS domain-containing protein 1 (Neuronal PAS1)
P97474	PITX2_MOUSE	Pituitary homeobox 2 (ALL1-responsive protein ARP1) (BRX1 homeoprotein) (Homeobox protein PITX2) (Orthodenticle-like homeobox 2) (Paired-like homeodomain transcription factor 2) (Paired-like homeodomain transcription factor Munc 30) (Solurshin)
P97479	MYO7A_MOUSE	Unconventional myosin-VIIa
P97494	GSH1_MOUSE	Glutamate--cysteine ligase catalytic subunit (EC 6.3.2.2) (GCS heavy chain) (Gamma-ECS) (Gamma-glutamylcysteine synthetase)
P97500	MYT1L_MOUSE	Myelin transcription factor 1-like protein (MyT1-L) (MyT1L) (Neural zinc finger factor 1) (NZF-1) (Postmitotic neural gene 1 protein) (Zinc finger protein Png-1)

P97770	THUM3_MOUSE	THUMP domain-containing protein 3 (GtROSA26asSor)
P97798	NEO1_MOUSE	Neogenin
P97814	PPIP1_MOUSE	Proline-serine-threonine phosphatase-interacting protein 1 (PEST phosphatase-interacting protein 1)
P97872	FMO5_MOUSE	Dimethylaniline monooxygenase [N-oxide-forming] 5 (EC 1.14.13.8) (Dimethylaniline oxidase 5) (Hepatic flavin-containing monooxygenase 5) (FMO 5)
P97929	BRCA2_MOUSE	Breast cancer type 2 susceptibility protein homolog (Fanconi anemia group D1 protein homolog)
P98191	CDS1_MOUSE	Phosphatidate cytidylyltransferase 1 (EC 2.7.7.41) (CDP-DAG synthase 1) (CDP-DG synthase 1) (CDP-diacylglycerol synthase 1) (CDS 1) (CDP-diglyceride pyrophosphorylase 1) (CDP-diglyceride synthase 1) (CTP:phosphatidate cytidylyltransferase 1)
Q00342	FLT3_MOUSE	Receptor-type tyrosine-protein kinase FLT3 (EC 2.7.10.1) (FL cytokine receptor) (Fetal liver kinase 2) (FLK-2) (Fms-like tyrosine kinase 3) (FLT-3) (Tyrosine-protein kinase receptor flk-2) (CD antigen CD135)
Q01063	PDE4D_MOUSE	cAMP-specific 3',5'-cyclic phosphodiesterase 4D (EC 3.1.4.53) (DPDE3)
Q02357	ANK1_MOUSE	Ankyrin-1 (ANK-1) (Erythrocyte ankyrin)
Q03391	NMDE4_MOUSE	Glutamate receptor ionotropic, NMDA 2D (GluN2D) (Glutamate [NMDA] receptor subunit epsilon-4) (N-methyl D-aspartate receptor subtype 2D) (NMDAR2D) (NR2D)
Q03963	E2AK2_MOUSE	Interferon-induced, double-stranded RNA-activated protein kinase (EC 2.7.11.1) (Eukaryotic translation initiation factor 2-alpha kinase 2) (eIF-2A protein kinase 2) (Interferon-inducible RNA-dependent protein kinase) (P1/eIF-2A protein kinase) (Protein kinase RNA-activated) (PKR) (Protein kinase R) (Serine/threonine-protein kinase TIK) (Tyrosine-protein kinase EIF2AK2) (EC 2.7.10.2) (p68 kinase)
Q04519	ASM_MOUSE	Sphingomyelin phosphodiesterase (EC 3.1.4.12) (Acid sphingomyelinase) (ASMase)
Q04891	SOX13_MOUSE	Transcription factor SOX-13 (SRY (Sex determining region Y)-box 13) (mSox13)
Q05117	PPA5_MOUSE	Tartrate-resistant acid phosphatase type 5 (TR-AP) (EC 3.1.3.2) (Tartrate-resistant acid ATPase) (TrATPase) (Type 5 acid phosphatase)
Q05306	COAA1_MOUSE	Collagen alpha-1(X) chain

Q05512	MARK2_MOUSE	Serine/threonine-protein kinase MARK2 (EC 2.7.11.1) (EC 2.7.11.26) (ELKL motif kinase 1) (EMK-1) (MAP/microtubule affinity-regulating kinase 2) (PAR1 homolog) (PAR1 homolog b) (Par-1b) (mPar-1b)
Q05769	PGH2_MOUSE	Prostaglandin G/H synthase 2 (EC 1.14.99.1) (Cyclooxygenase-2) (COX-2) (Glucocorticoid-regulated inflammatory cyclooxygenase) (Gripghs) (Macrophage activation-associated marker protein P71/73) (PES-2) (PHS II) (Prostaglandin H2 synthase 2) (PGH synthase 2) (PGHS-2) (Prostaglandin-endoperoxide synthase 2) (TIS10 protein)
Q05860	FMN1_MOUSE	Formin-1 (Limb deformity protein)
Q06806	TIE1_MOUSE	Tyrosine-protein kinase receptor Tie-1 (EC 2.7.10.1)
Q07417	ACADS_MOUSE	Short-chain specific acyl-CoA dehydrogenase, mitochondrial (SCAD) (EC 1.3.8.1) (Butyryl-CoA dehydrogenase)
Q08024	PEBB_MOUSE	Core-binding factor subunit beta (CBF-beta) (Polyomavirus enhancer-binding protein 2 beta subunit) (PEA2-beta) (PEBP2-beta) (SL3-3 enhancer factor 1 subunit beta) (SL3/AKV core-binding factor beta subunit)
Q09XV5	CHD8_MOUSE	Chromodomain-helicase-DNA-binding protein 8 (CHD-8) (EC 3.6.4.12) (ATP-dependent helicase CHD8) (Axis duplication inhibitor) (Duplin)
Q0VAY3	F187B_MOUSE	Protein FAM187B (Transmembrane protein 162)
Q0VBL1	TIGD2_MOUSE	Tigger transposable element-derived protein 2
Q0VBU9	PLPP4_MOUSE	Phospholipid phosphatase 4 (EC 3.1.3.4) (Phosphatidic acid phosphatase type 2 domain-containing protein 1A)
Q0VDU3	GPR15_MOUSE	G-protein coupled receptor 15
Q0VF58	COJA1_MOUSE	Collagen alpha-1(XIX) chain (Collagen alpha-1(Y) chain)
Q0VGM9	RTEL1_MOUSE	Regulator of telomere elongation helicase 1 (EC 3.6.4.12)
Q148W0	AT8B1_MOUSE	Phospholipid-transporting ATPase IC (EC 7.6.2.1) (ATPase class I type 8B member 1) (P4-ATPase flippase complex alpha subunit ATP8B1)
Q14AW5	NSUN7_MOUSE	Putative methyltransferase NSUN7 (EC 2.1.1.-) (NOL1/NOP2/Sun domain family member 7)
Q1PSW8	LIN41_MOUSE	E3 ubiquitin-protein ligase TRIM71 (EC 2.3.2.27) (Protein lin-41 homolog) (mLin41) (RING-type E3

		ubiquitin transferase TRIM71) (Tripartite motif-containing protein 71)
Q2TA57	ASPH1_MOUSE	Aspartate beta-hydroxylase domain-containing protein 1 (EC 1.14.11.-)
Q2TBA3	MALT1_MOUSE	Mucosa-associated lymphoid tissue lymphoma translocation protein 1 homolog (EC 3.4.22.-) (Paracaspase)
Q2WF71	LRFN1_MOUSE	Leucine-rich repeat and fibronectin type III domain-containing protein 1 (Synaptic adhesion-like molecule 2) (Synaptic differentiation-enhancing molecule 1)
Q32Q92	ACOT6_MOUSE	Acyl-coenzyme A thioesterase 6 (Acyl-CoA thioesterase 6) (EC 3.1.2.-)
Q3LAC4	PREX2_MOUSE	Phosphatidylinositol 3,4,5-trisphosphate-dependent Rac exchanger 2 protein (P-Rex2) (PtdIns(3,4,5)-dependent Rac exchanger 2) (DEP domain-containing protein 2)
Q3SXG2	FPRS6_MOUSE	Formyl peptide receptor-related sequence 6
Q3TC93	H1BP3_MOUSE	HCLS1-binding protein 3 (HS1-binding protein 3) (HSP1BP-3)
Q3TCV3	GSAP_MOUSE	Gamma-secretase-activating protein (GSAP) (Protein pigeon homolog) [Cleaved into: Gamma-secretase-activating protein 16 kDa C-terminal form (GSAP-16K)]
Q3TFQ1	SPRY7_MOUSE	SPRY domain-containing protein 7 (Chronic lymphocytic leukemia deletion region gene 6 protein homolog) (CLL deletion region gene 6 protein homolog)
Q3TIR1	TPC13_MOUSE	Trafficking protein particle complex subunit 13
Q3TIX9	SNUT2_MOUSE	U4/U6.U5 tri-snRNP-associated protein 2 (Inactive ubiquitin-specific peptidase 39)
Q3TNW5	MUCL3_MOUSE	Mucin-like protein 3 (Diffuse panbronchiolitis critical region protein 1 homolog)
Q3TP92	CNEP1_MOUSE	CTD nuclear envelope phosphatase 1 (EC 3.1.3.16) (Serine/threonine-protein phosphatase dullard)
Q3TPX4	EXOC5_MOUSE	Exocyst complex component 5 (Exocyst complex component Sec10)
Q3TUU5	TEX30_MOUSE	Testis-expressed protein 30
Q3TYA6	MPP8_MOUSE	M-phase phosphoprotein 8
Q3U0S6	RAIN_MOUSE	Ras-interacting protein 1 (Rain)
Q3U133	ZN746_MOUSE	Zinc finger protein 746
Q3U1J4	DDB1_MOUSE	DNA damage-binding protein 1 (DDB p127 subunit) (Damage-specific DNA-binding protein 1) (UV-damaged DNA-binding factor)

Q3U269	TSTD2_MOUSE	Thiosulfate sulfurtransferase/rhodanese-like domain-containing protein 2
Q3U276	SDHF1_MOUSE	Succinate dehydrogenase assembly factor 1, mitochondrial (SDH assembly factor 1) (SDHAF1) (LYR motif-containing protein 8)
Q3U2C5	RN149_MOUSE	E3 ubiquitin-protein ligase RNF149 (EC 2.3.2.27) (Goliath-related E3 ubiquitin-protein ligase 4) (RING finger protein 149) (RING-type E3 ubiquitin transferase RNF149)
Q3U2K0	F193B_MOUSE	Protein FAM193B
Q3U481	MFS12_MOUSE	Major facilitator superfamily domain-containing protein 12
Q3U4G3	XXLT1_MOUSE	Xyloside xylosyltransferase 1 (EC 2.4.2.n3) (UDP-xylose:alpha-xyloside alpha-1,3-xylosyltransferase)
Q3U5C7	PRIC1_MOUSE	Prickle-like protein 1
Q3U5Q7	CMPK2_MOUSE	UMP-CMP kinase 2, mitochondrial (EC 2.7.4.14) (Nucleoside-diphosphate kinase) (EC 2.7.4.6) (Thymidylate kinase LPS-inducible member) (TYKi)
Q3UA06	PCH2_MOUSE	Pachytene checkpoint protein 2 homolog (Thyroid hormone receptor interactor 13) (Thyroid receptor-interacting protein 13) (TR-interacting protein 13) (TRIP-13)
Q3UGC7	EI3JA_MOUSE	Eukaryotic translation initiation factor 3 subunit J-A (eIF3j-A) (Eukaryotic translation initiation factor 3 subunit 1-A) (eIF-3-alpha-A) (eIF3 p35)
Q3UGF1	WDR19_MOUSE	WD repeat-containing protein 19 (Intraflagellar transport 144 homolog)
Q3UGP9	LRC58_MOUSE	Leucine-rich repeat-containing protein 58
Q3UGS4	MCRI1_MOUSE	Mapk-regulated corepressor-interacting protein 1 (Protein FAM195B)
Q3UHB1	NT5D3_MOUSE	5'-nucleotidase domain-containing protein 3 (EC 3.1.3.-) (GRP94-neighboring nucleotidase)
Q3UHC7	DAB2P_MOUSE	Disabled homolog 2-interacting protein (DAB2-interacting protein) (ASK-interacting protein 1) (DOC-2/DAB-2 interactive protein)
Q3UHD9	AGAP2_MOUSE	Arf-GAP with GTPase, ANK repeat and PH domain-containing protein 2 (AGAP-2) (Centaurin-gamma-1) (Cnt-g1) (Phosphatidylinositol 3-kinase enhancer) (PIKE)
Q3UHF7	ZEP2_MOUSE	Transcription factor HIVEP2 (Human immunodeficiency virus type I enhancer-binding protein 2 homolog) (Myc intron-binding protein 1) (MIBP-1)

Q3UHH8	GXLT1_MOUSE	Glucoside xylosyltransferase 1 (EC 2.4.2.42) (Glycosyltransferase 8 domain-containing protein 3)
Q3UHL1	CAMKV_MOUSE	CaM kinase-like vesicle-associated protein
Q3UJP5	CH037_MOUSE	Protein C8orf37 homolog
Q3UKK2	CEAM5_MOUSE	Carcinoembryonic antigen-related cell adhesion molecule 5 (Pregnancy-specific glycoprotein 30)
Q3UMW7	MAPK3_MOUSE	MAP kinase-activated protein kinase 3 (MAPK-activated protein kinase 3) (MAPKAP kinase 3) (MAPKAP-K3) (MAPKAPK-3) (MK-3) (EC 2.7.11.1)
Q3UQ41	TM11A_MOUSE	Transmembrane protease serine 11A (EC 3.4.21.-) (Airway trypsin-like protease 1) (Serine protease DESC3) (DESC-3)
Q3UQV5	KBTB8_MOUSE	Kelch repeat and BTB domain-containing protein 8
Q3UR50	VW5B2_MOUSE	von Willebrand factor A domain-containing protein 5B2
Q3URE1	ACSF3_MOUSE	Malonate--CoA ligase ACSF3, mitochondrial (EC 6.2.1.n3) (Acyl-CoA synthetase family member 3)
Q3URU2	PEG3_MOUSE	Paternally-expressed gene 3 protein (ASF-1)
Q3UTQ8	CDKL5_MOUSE	Cyclin-dependent kinase-like 5 (EC 2.7.11.22)
Q3UUY6	PROM2_MOUSE	Prominin-2 (PROM-2) (Prominin-like protein 2) (mPROML2)
Q3UV70	PDP1_MOUSE	[Pyruvate dehydrogenase [acetyl-transferring]]-phosphatase 1, mitochondrial (PDP 1) (EC 3.1.3.43) (Protein phosphatase 2C) (Pyruvate dehydrogenase phosphatase catalytic subunit 1) (PDPC 1)
Q3UVL4	VPS51_MOUSE	Vacuolar protein sorting-associated protein 51 homolog (Protein fat-free homolog)
Q3UX61	NAA11_MOUSE	N-alpha-acetyltransferase 11 (EC 2.3.1.255) (N-terminal acetyltransferase complex ARD1 subunit homolog B) (NatA catalytic subunit Naa11)
Q3UZ01	RNPC3_MOUSE	RNA-binding region-containing protein 3 (RNA-binding motif protein 40) (RNA-binding protein 40)
Q3UZV7	K132L_MOUSE	UPF0577 protein KIAA1324-like homolog (Estrogen-induced gene 121-like protein) (EIG121L)
Q3V0Q1	DYH12_MOUSE	Dynein heavy chain 12, axonemal (Axonemal beta dynein heavy chain 12) (Axonemal dynein heavy chain 12-like protein) (Axonemal dynein heavy chain 7-like protein) (Ciliary dynein heavy chain 12)

Q3V1F8	EPHX3_MOUSE	Epoxide hydrolase 3 (EH3) (EC 3.3.2.10) (Abhydrolase domain-containing protein 9)
Q3V3V9	CARL2_MOUSE	Capping protein, Arp2/3 and myosin-I linker protein 2 (Capping protein regulator and myosin 1 linker 2) (F-actin-uncapping protein RLTPR) (Leucine-rich repeat-containing protein 16C) (RGD, leucine-rich repeat, tropomodulin and proline-rich-containing protein)
Q4VA45	CK095_MOUSE	Uncharacterized protein C11orf95 homolog
Q4VBE8	WDR18_MOUSE	WD repeat-containing protein 18
Q4VC33	MAEA_MOUSE	E3 ubiquitin-protein transferase MAEA (EC 2.3.2.27) (Erythroblast macrophage protein) (Macrophage erythroblast attacher)
Q505D1	ANR28_MOUSE	Serine/threonine-protein phosphatase 6 regulatory ankyrin repeat subunit A (PP6-ARS-A) (Serine/threonine-protein phosphatase 6 regulatory subunit ARS-A) (Ankyrin repeat domain-containing protein 28) (Phosphatase interactor targeting protein hnRNP K) (PITK)
Q505F1	NR2C1_MOUSE	Nuclear receptor subfamily 2 group C member 1 (Orphan nuclear receptor TR2) (Testicular receptor 2) (mTR2)
Q569Z5	DDX46_MOUSE	Probable ATP-dependent RNA helicase DDX46 (EC 3.6.4.13) (DEAD box protein 46)
Q56A10	ZN608_MOUSE	Zinc finger protein 608
Q571F8	GLSL_MOUSE	Glutaminase liver isoform, mitochondrial (GLS) (EC 3.5.1.2) (L-glutaminase) (L-glutamine amidohydrolase) (LGA)
Q58FA4	E2F8_MOUSE	Transcription factor E2F8 (E2F-8)
Q5DTK1	CHSS3_MOUSE	Chondroitin sulfate synthase 3 (EC 2.4.1.175) (EC 2.4.1.226) (Carbohydrate synthase 2) (Chondroitin glucuronyltransferase 3) (Chondroitin synthase 2) (ChSy-2) (Glucuronosyl-N-acetylgalactosaminyl-proteoglycan 4-beta-N-acetylgalactosaminyltransferase II) (N-acetylgalactosaminyl-proteoglycan 3-beta-glucuronosyltransferase 3) (N-acetylgalactosaminyltransferase 3)
Q5DTT8	PNMA5_MOUSE	Paraneoplastic antigen-like protein 5
Q5DTW7	RESF1_MOUSE	Retroelement silencing factor 1
Q5DU41	LRC8B_MOUSE	Volume-regulated anion channel subunit LRRC8B (Leucine-rich repeat-containing protein 8B) (T-cell activation leucine repeat-rich protein) (TA-LRRP)
Q5F226	FAT2_MOUSE	Protocadherin Fat 2 (FAT tumor suppressor homolog 2)

Q5FWH2	UNKL_MOUSE	Putative E3 ubiquitin-protein ligase UNKL (EC 2.3.2.-) (RING finger protein unkempt-like)
Q5H8B9	FREM3_MOUSE	FRAS1-related extracellular matrix protein 3 (NV domain-containing protein 2)
Q5I012	S38AA_MOUSE	Putative sodium-coupled neutral amino acid transporter 10 (Solute carrier family 38 member 10)
Q5K6N0	TM232_MOUSE	Transmembrane protein 232 (Testis-specific protein 13)
Q5M8M9	LRC52_MOUSE	Leucine-rich repeat-containing protein 52 (BK channel auxiliary gamma subunit LRRC52)
Q5PSV9	MDC1_MOUSE	Mediator of DNA damage checkpoint protein 1
Q5RKZ7	MOCS1_MOUSE	Molybdenum cofactor biosynthesis protein 1 [Includes: GTP 3',8-cyclase (EC 4.1.99.22) (Molybdenum cofactor biosynthesis protein A); Cyclic pyranopterin monophosphate synthase (EC 4.6.1.17) (Molybdenum cofactor biosynthesis protein C)]
Q5SNZ0	GRDN_MOUSE	Girdin (Akt phosphorylation enhancer) (APE) (Coiled-coil domain-containing protein 88A) (G alpha-interacting vesicle-associated protein) (GIV) (Girders of actin filament) (Hook-related protein 1) (HkRP1)
Q5SQM0	EMAL6_MOUSE	Echinoderm microtubule-associated protein-like 6 (EMAP-6) (Echinoderm microtubule-associated protein-like 5-like)
Q5SQY2	BOD1_MOUSE	Biorientation of chromosomes in cell division protein 1 (Biorientation defective protein 1) (Protein FAM44B)
Q5SSE9	ABCAD_MOUSE	ATP-binding cassette sub-family A member 13
Q5TIS6	NOTO_MOUSE	Homeobox protein notochord
Q5U430	UBR3_MOUSE	E3 ubiquitin-protein ligase UBR3 (EC 2.3.2.27) (N-recognin-3) (RING-type E3 ubiquitin transferase UBR3) (Ubiquitin-protein ligase E3-alpha-3) (Ubiquitin-protein ligase E3-alpha-III) (Zinc finger protein 650)
Q5U4D9	THOC6_MOUSE	THO complex subunit 6 homolog (WD repeat-containing protein 58)
Q5U5M8	BL1S3_MOUSE	Biogenesis of lysosome-related organelles complex 1 subunit 3 (BLOC-1 subunit 3) (Reduced pigmentation protein)
Q5XJV7	SETD5_MOUSE	Histone-lysine N-methyltransferase SETD5 (EC 2.1.1.-) (SET domain-containing protein 5)
Q5XKE0	MYPC2_MOUSE	Myosin-binding protein C, fast-type (Fast MyBP-C) (C-protein, skeletal muscle fast isoform)
Q60610	TIAM1_MOUSE	T-lymphoma invasion and metastasis-inducing protein 1 (TIAM-1)

Q60629	EPHA5_MOUSE	Ephrin type-A receptor 5 (EC 2.7.10.1) (Brain-specific kinase) (CEK-7) (EPH homology kinase 1) (EHK-1)
Q60680	IKKA_MOUSE	Inhibitor of nuclear factor kappa-B kinase subunit alpha (I-kappa-B kinase alpha) (IKK-A) (IKK-alpha) (IkBKA) (IkappaB kinase) (EC 2.7.11.10) (Conserved helix-loop-helix ubiquitous kinase) (I-kappa-B kinase 1) (IKK1) (Nuclear factor NF-kappa-B inhibitor kinase alpha) (NFKBIKA)
Q60769	TNAP3_MOUSE	Tumor necrosis factor alpha-induced protein 3 (TNF alpha-induced protein 3) (EC 2.3.2.-) (EC 3.4.19.12) (Putative DNA-binding protein A20) (Zinc finger protein A20)
Q60779	DRC4_MOUSE	Dynein regulatory complex subunit 4 (Growth arrest-specific protein 11) (GAS-11) (Growth arrest-specific protein 8) (GAS-8)
Q60795	NF2L2_MOUSE	Nuclear factor erythroid 2-related factor 2 (NF-E2-related factor 2) (NFE2-related factor 2) (Nuclear factor, erythroid derived 2, like 2)
Q60865	CAPR1_MOUSE	Caprin-1 (Cytoplasmic activation- and proliferation-associated protein 1) (GPI-anchored membrane protein 1) (GPI-anchored protein p137) (GPI-p137) (p137GPI) (Membrane component chromosome 11 surface marker 1) (RNA granule protein 105)
Q60866	PTER_MOUSE	Phosphotriesterase-related protein (EC 3.1.-.-) (Parathion hydrolase-related protein)
Q60891	OL139_MOUSE	Olfactory receptor 139 (Odorant receptor M5) (Olfactory receptor 1) (Olfactory receptor 255-2)
Q60936	COQ8A_MOUSE	Atypical kinase COQ8A, mitochondrial (EC 2.7.-.-) (Chaperone activity of bc1 complex-like) (Chaperone-ABC1-like) (Coenzyme Q protein 8A) (aarF domain-containing protein kinase 3)
Q60949	TBCD1_MOUSE	TBC1 domain family member 1
Q60953	PML_MOUSE	Protein PML
Q60960	IMA5_MOUSE	Importin subunit alpha-5 (Importin alpha-S1) (Karyopherin subunit alpha-1) (Nucleoprotein interactor 1) (NPI-1) (RAG cohort protein 2) (SRP1-beta)
Q60967	PAPS1_MOUSE	Bifunctional 3'-phosphoadenosine 5'-phosphosulfate synthase 1 (PAPS synthase 1) (PAPSS 1) (Sulfurylase kinase 1) (SK 1) (SK1) [Includes: Sulfate adenylyltransferase (EC 2.7.7.4) (ATP-sulfurylase) (Sulfate adenylate transferase) (SAT); Adenylyl-sulfate kinase (EC 2.7.1.25) (3'-phosphoadenosine-5'-phosphosulfate synthase)

		(APS kinase) (Adenosine-5'-phosphosulfate 3'-phosphotransferase) (Adenylylsulfate 3'-phosphotransferase)]
Q60974	NCOR1_MOUSE	Nuclear receptor corepressor 1 (N-CoR) (N-CoR1) (Retinoid X receptor-interacting protein 13) (RIP13)
Q61089	FZD6_MOUSE	Frizzled-6 (Fz-6) (mFz6)
Q61107	GBP4_MOUSE	Guanylate-binding protein 4 (EC 3.6.5.-) (GTP-binding protein 3) (GBP-3) (GTP-binding protein 4) (GBP-4) (Guanine nucleotide-binding protein 4) (Guanylate-binding protein 3)
Q61116	ZN235_MOUSE	Zinc finger protein 235 (Zinc finger protein 93) (Zfp-93)
Q61143	TRPC6_MOUSE	Short transient receptor potential channel 6 (TrpC6) (Calcium entry channel) (Transient receptor protein 6) (TRP-6)
Q61161	M4K2_MOUSE	Mitogen-activated protein kinase kinase kinase kinase 2 (EC 2.7.11.1) (Germinal center kinase) (GCK) (MAPK/ERK kinase kinase kinase 2) (MEK kinase kinase 2) (MEKKK 2) (Rab8-interacting protein)
Q61330	CNTN2_MOUSE	Contactin-2 (Axonal glycoprotein TAG-1) (Axonin-1) (Transient axonal glycoprotein 1) (TAX-1)
Q61333	TNAP2_MOUSE	Tumor necrosis factor alpha-induced protein 2 (TNF alpha-induced protein 2) (Primary response gene B94 protein)
Q61471	TOB1_MOUSE	Protein Tob1 (Transducer of erbB-2 1)
Q61526	ERBB3_MOUSE	Receptor tyrosine-protein kinase erbB-3 (EC 2.7.10.1) (Glial growth factor receptor) (Proto-oncogene-like protein c-ErbB-3)
Q61554	FBN1_MOUSE	Fibrillin-1 [Cleaved into: Asprosin]
Q61603	GLRA4_MOUSE	Glycine receptor subunit alpha-4
Q61606	GLR_MOUSE	Glucagon receptor (GL-R)
Q61627	GRID1_MOUSE	Glutamate receptor ionotropic, delta-1 (GluD1) (GluR delta-1 subunit)
Q61666	HIRA_MOUSE	Protein HIRA (TUP1-like enhancer of split protein 1)
Q61694	3BHS5_MOUSE	NADPH-dependent 3-keto-steroid reductase Hsd3b5 (3 beta-hydroxysteroid dehydrogenase type 5) (3 beta-hydroxysteroid dehydrogenase type V) (3 beta-HSD V) (EC 1.1.1.270) (Dihydrotestosterone 3-ketoreductase) (EC 1.1.1.210)
Q61701	ELAV4_MOUSE	ELAV-like protein 4 (Hu-antigen D) (HuD) (Paraneoplastic encephalomyelitis antigen HuD)

Q61772	EPHA7_MOUSE	Ephrin type-A receptor 7 (EC 2.7.10.1) (Developmental kinase 1) (mDK-1) (EPH homology kinase 3) (EHK-3) (Embryonic brain kinase) (EBK)
Q61818	RAI1_MOUSE	Retinoic acid-induced protein 1
Q61824	ADA12_MOUSE	Disintegrin and metalloproteinase domain-containing protein 12 (ADAM 12) (EC 3.4.24.-) (Meltrin-alpha)
Q61850	FOXC2_MOUSE	Forkhead box protein C2 (Brain factor 3) (BF-3) (Forkhead-related protein FKHL14) (Mesenchyme fork head protein 1) (MFH-1 protein) (Transcription factor FKH-14)
Q61985	NF2L1_MOUSE	Endoplasmic reticulum membrane sensor NFE2L1 (Locus control region-factor 1) (LCR-F1) (Nuclear factor erythroid 2-related factor 1) (NF-E2-related factor 1) (NFE2-related factor 1) (Nuclear factor, erythroid derived 2, like 1) [Cleaved into: Transcription factor NRF1]
Q62059	CSPG2_MOUSE	Versican core protein (Chondroitin sulfate proteoglycan core protein 2) (Chondroitin sulfate proteoglycan 2) (Large fibroblast proteoglycan) (PG-M)
Q62388	ATM_MOUSE	Serine-protein kinase ATM (EC 2.7.11.1) (Ataxia telangiectasia mutated homolog) (A-T mutated homolog)
Q62446	FKBP3_MOUSE	Peptidyl-prolyl cis-trans isomerase FKBP3 (PPIase FKBP3) (EC 5.2.1.8) (25 kDa FK506-binding protein) (25 kDa FKBP) (FKBP-25) (FK506-binding protein 3) (FKBP-3) (Immunophilin FKBP25) (Rapamycin-selective 25 kDa immunophilin) (Rotamase)
Q62463	V1AR_MOUSE	Vasopressin V1a receptor (V1aR) (AVPR V1a) (Antidiuretic hormone receptor 1a) (Vascular/hepatic-type arginine vasopressin receptor)
Q62469	ITA2_MOUSE	Integrin alpha-2 (CD49 antigen-like family member B) (Collagen receptor) (Platelet membrane glycoprotein Ia) (GPIa) (VLA-2 subunit alpha) (CD antigen CD49b)
Q63810	CANB1_MOUSE	Calcineurin subunit B type 1 (Protein phosphatase 2B regulatory subunit 1) (Protein phosphatase 3 regulatory subunit B alpha isoform 1)
Q63943	MEF2D_MOUSE	Myocyte-specific enhancer factor 2D
Q63ZW7	INADL_MOUSE	InaD-like protein (Inadl protein) (Channel-interacting PDZ domain-containing protein) (Pals1-

		associated tight junction protein) (Protein associated to tight junctions)
Q64112	IFIT2_MOUSE	Interferon-induced protein with tetratricopeptide repeats 2 (IFIT-2) (Glucocorticoid-attenuated response gene 39 protein) (GARG-39) (Interferon-induced 54 kDa protein) (IFI-54K) (P54)
Q64176	EST1E_MOUSE	Carboxylesterase 1E (EC 3.1.1.1) (Egasyn) (Liver carboxylesterase 22) (Es-22) (Esterase-22)
Q64213	SF01_MOUSE	Splicing factor 1 (CW17) (Mammalian branch point-binding protein) (BBP) (mBBP) (Transcription factor ZFM1) (mZFM) (Zinc finger gene in MEN1 locus) (Zinc finger protein 162)
Q64282	IFIT1_MOUSE	Interferon-induced protein with tetratricopeptide repeats 1 (IFIT-1) (Glucocorticoid-attenuated response gene 16 protein) (GARG-16) (Interferon-induced 56 kDa protein) (IFI-56K) (P56)
Q64287	IRF4_MOUSE	Interferon regulatory factor 4 (IRF-4) (Lymphocyte-specific interferon regulatory factor) (LSIRF) (NF-EM5) (PU.1 interaction partner) (Transcriptional activator PIP)
Q64318	ZEB1_MOUSE	Zinc finger E-box-binding homeobox 1 (Delta EF1) (Transcription factor 8) (TCF-8) (Zinc finger homeobox protein 1a) (MEB1)
Q64321	ZBT7B_MOUSE	Zinc finger and BTB domain-containing protein 7B (Krueppel-related zinc finger protein cKrox) (c-Krox) (T-helper-inducing POZ/Krueppel-like factor) (Zinc finger protein 67) (Zfp-67) (Zinc finger protein Th-POK)
Q64338	PDE1C_MOUSE	Calcium/calmodulin-dependent 3',5'-cyclic nucleotide phosphodiesterase 1C (Cam-PDE 1C) (3',5'-cyclic-AMP phosphodiesterase) (EC 3.1.4.-) (3',5'-cyclic-GMP phosphodiesterase) (EC 3.1.4.35)
Q64345	IFIT3_MOUSE	Interferon-induced protein with tetratricopeptide repeats 3 (IFIT-3) (Glucocorticoid-attenuated response gene 49 protein) (GARG-49) (P49) (IRG2)
Q64433	CH10_MOUSE	10 kDa heat shock protein, mitochondrial (Hsp10) (10 kDa chaperonin) (Chaperonin 10) (CPN10)
Q64459	CP3AB_MOUSE	Cytochrome P450 3A11 (EC 1.14.14.1) (CYPIIIA11) (Cytochrome P-450IIIAM1) (Cytochrome P-450UT)
Q64487	PTPRD_MOUSE	Receptor-type tyrosine-protein phosphatase delta (Protein-tyrosine phosphatase delta) (R-PTP-delta) (EC 3.1.3.48)
Q64512	PTN13_MOUSE	Tyrosine-protein phosphatase non-receptor type 13 (EC 3.1.3.48) (PTP36) (Protein tyrosine

		phosphatase DPZPTP) (Protein tyrosine phosphatase PTP-BL) (Protein-tyrosine phosphatase RIP)
Q64669	NQO1_MOUSE	NAD(P)H dehydrogenase [quinone] 1 (EC 1.6.5.2) (Azoreductase) (DT-diaphorase) (DTD) (Menadione reductase) (NAD(P)H:quinone oxidoreductase 1) (Phylloquinone reductase) (Quinone reductase 1) (QR1)
Q64676	CGT_MOUSE	2-hydroxyacylsphingosine 1-beta-galactosyltransferase (EC 2.4.1.47) (Ceramide UDP-galactosyltransferase) (Cerebroside synthase) (UDP-galactose-ceramide galactosyltransferase)
Q66JT7	COQ2_MOUSE	4-hydroxybenzoate polyprenyltransferase, mitochondrial (4-HB polyprenyltransferase) (EC 2.5.1.39) (Para-hydroxybenzoate--polyprenyltransferase) (PHB:PPT) (PHB:polyprenyltransferase)
Q66JZ4	TCAIM_MOUSE	T-cell activation inhibitor, mitochondrial (Tolerance associated gene-1 protein) (TOAG-1)
Q66K08	CILP1_MOUSE	Cartilage intermediate layer protein 1 (CILP-1) [Cleaved into: Cartilage intermediate layer protein 1 C1; Cartilage intermediate layer protein 1 C2]
Q66LM6	F170A_MOUSE	Protein FAM170A (Zinc finger domain-containing protein) (Zinc finger protein ZNFD)
Q68FE6	RIPR1_MOUSE	Rho family-interacting cell polarization regulator 1
Q68FE8	Z280D_MOUSE	Zinc finger protein 280D (Suppressor of hairy wing homolog 4)
Q69Z26	CNTN4_MOUSE	Contactin-4 (Brain-derived immunoglobulin superfamily protein 2) (BIG-2)
Q69Z37	SAM9L_MOUSE	Sterile alpha motif domain-containing protein 9-like (SAM domain-containing protein 9-like)
Q69Z98	BRSK2_MOUSE	Serine/threonine-protein kinase BRSK2 (EC 2.7.11.1) (EC 2.7.11.26) (Brain-specific serine/threonine-protein kinase 2) (BR serine/threonine-protein kinase 2) (Serine/threonine-protein kinase SAD-A)
Q69ZA1	CDK13_MOUSE	Cyclin-dependent kinase 13 (EC 2.7.11.22) (EC 2.7.11.23) (CDC2-related protein kinase 5) (Cell division cycle 2-like protein kinase 5) (Cell division protein kinase 13)
Q69ZB0	LRCC1_MOUSE	Leucine-rich repeat and coiled-coil domain-containing protein 1
Q69ZM6	STK36_MOUSE	Serine/threonine-protein kinase 36 (EC 2.7.11.1) (Fused homolog)
Q69ZN6	GNPTA_MOUSE	N-acetylglucosamine-1-phosphotransferase subunits alpha/beta (EC 2.7.8.17) (GlcNAc-1-

		phosphotransferase subunits alpha/beta) (Stealth protein GNPTAB) (UDP-N-acetylglucosamine-1-phosphotransferase subunits alpha/beta) [Cleaved into: N-acetylglucosamine-1-phosphotransferase subunit alpha; N-acetylglucosamine-1-phosphotransferase subunit beta]
Q69ZT9	TBC30_MOUSE	TBC1 domain family member 30
Q69ZZ6	TMCC1_MOUSE	Transmembrane and coiled-coil domains protein 1
Q6A037	N4BP1_MOUSE	NEDD4-binding protein 1 (N4BP1)
Q6A070	TGRM1_MOUSE	TOG array regulator of axonemal microtubules protein 1 (Crescerin-1) (Protein FAM179B)
Q6A098	SBP2L_MOUSE	Selenocysteine insertion sequence-binding protein 2-like (SECIS-binding protein 2-like)
Q6B966	NAL14_MOUSE	NACHT, LRR and PYD domains-containing protein 14 (NALP-iota) (Germ cell specific leucine-rich repeat NTPase)
Q6EBV9	ATG9B_MOUSE	Autophagy-related protein 9B (APG9-like 2) (Nitric oxide synthase 3-overlapping antisense gene protein)
Q6GQT5	T151A_MOUSE	Transmembrane protein 151A
Q6GQV7	EDRF1_MOUSE	Erythroid differentiation-related factor 1
Q6GQW0	BTBDB_MOUSE	Ankyrin repeat and BTB/POZ domain-containing protein BTBD11 (BTB/POZ domain-containing protein 11)
Q6IEE6	T132E_MOUSE	Transmembrane protein 132E
Q6IMP4	PANX2_MOUSE	Pannexin-2
Q6KAS7	ZN521_MOUSE	Zinc finger protein 521 (Ecotropic viral integration site 3 protein)
Q6KCD5	NIPBL_MOUSE	Nipped-B-like protein (Delangin homolog) (SCC2 homolog)
Q6NS45	CCD66_MOUSE	Coiled-coil domain-containing protein 66
Q6NS69	AMER3_MOUSE	APC membrane recruitment protein 3 (Amer3) (Protein FAM123C)
Q6NSR8	PEPL1_MOUSE	Probable aminopeptidase NPEPL1 (EC 3.4.11.-) (Aminopeptidase-like 1)
Q6NV72	WDCP_MOUSE	WD repeat and coiled-coil-containing protein
Q6NVE8	WDR44_MOUSE	WD repeat-containing protein 44 (Rabphilin-11)
Q6NXJ0	WWC2_MOUSE	Protein WWC2 (WW domain-containing protein 2)
Q6NXY9	RPC7_MOUSE	DNA-directed RNA polymerase III subunit RPC7 (RNA polymerase III subunit C7) (DNA-directed RNA polymerase III subunit G)
Q6NZB0	DNJC8_MOUSE	DnaJ homolog subfamily C member 8
Q6NZC7	S23IP_MOUSE	SEC23-interacting protein
Q6NZK5	K1328_MOUSE	Protein hinderin

Q6NZR2	MSD2_MOUSE	Myb/SANT-like DNA-binding domain-containing protein 2
Q6P1E8	EFCB6_MOUSE	EF-hand calcium-binding domain-containing protein 6 (DJ-1-binding protein) (DJBP)
Q6P1I3	CL066_MOUSE	KICSTOR complex protein C12orf66 homolog
Q6P1Y8	INP4B_MOUSE	Type II inositol 3,4-bisphosphate 4-phosphatase (EC 3.1.3.66) (Inositol polyphosphate 4-phosphatase type II)
Q6P5C5	SMUG1_MOUSE	Single-strand selective monofunctional uracil DNA glycosylase (EC 3.2.2.-)
Q6P5D3	DHX57_MOUSE	Putative ATP-dependent RNA helicase DHX57 (EC 3.6.4.13) (DEAH box protein 57)
Q6P5G6	UBXN7_MOUSE	UBX domain-containing protein 7
Q6P5H2	NEST_MOUSE	Nestin
Q6P6J9	TXD15_MOUSE	Thioredoxin domain-containing protein 15
Q6P6M5	PX11C_MOUSE	Peroxisomal membrane protein 11C (Peroxin-11C) (Peroxisomal biogenesis factor 11C) (Protein PEX11 homolog gamma) (PEX11-gamma)
Q6P9Q6	FKB15_MOUSE	FK506-binding protein 15 (FKBP-15) (133 kDa FK506-binding protein) (133 kDa FKBP) (FKBP-133) (WASP and FKBP-like) (WAFL)
Q6P9S1	ATMIN_MOUSE	ATM interactor (ATM/ATR-substrate CHK2-interacting zinc finger protein) (ASCIZ)
Q6PAJ3	GARE2_MOUSE	GRB2-associated and regulator of MAPK protein 2 (GRB2-associated and regulator of MAPK1-like)
Q6PAN7	PRR18_MOUSE	Proline-rich protein 18
Q6PAV2	HERC4_MOUSE	Probable E3 ubiquitin-protein ligase HERC4 (EC 2.3.2.26) (HECT domain and RCC1-like domain-containing protein 4) (HECT-type E3 ubiquitin transferase HERC4)
Q6PDK2	KMT2D_MOUSE	Histone-lysine N-methyltransferase 2D (Lysine N-methyltransferase 2D) (EC 2.1.1.354) (ALL1-related protein) (Myeloid/lymphoid or mixed-lineage leukemia protein 2)
Q6PFD6	KI18B_MOUSE	Kinesin-like protein KIF18B
Q6PGG2	GMIP_MOUSE	GEM-interacting protein (GMIP)
Q6PHQ8	NAA35_MOUSE	N-alpha-acetyltransferase 35, NatC auxiliary subunit (Embryonic growth-associated protein) (Protein MAK10 homolog)
Q6PIJ4	NFRKB_MOUSE	Nuclear factor related to kappa-B-binding protein (DNA-binding protein R kappa-B)
Q6PNC0	DMXL1_MOUSE	DmX-like protein 1 (X-like 1 protein)
Q6Q473	CLA4A_MOUSE	Calcium-activated chloride channel regulator 4A (EC 3.4.-.-) (Calcium-activated chloride channel regulator 6) (mClca6) [Cleaved into: Calcium-

		activated chloride channel regulator 4A, 110 kDa form; Calcium-activated chloride channel regulator 4A, 30 kDa form]
Q6R653	UN5CL_MOUSE	UNC5C-like protein (Protein unc-5 homolog C-like) (ZU5 and death domain-containing protein)
Q6VGS5	DAPLE_MOUSE	Protein Daple (Coiled-coil domain-containing protein 88C) (Dvl-associating protein with a high frequency of leucine residues)
Q6VH22	IF172_MOUSE	Intraflagellar transport protein 172 homolog (Protein wimple)
Q6ZPJ3	UBE2O_MOUSE	(E3-independent) E2 ubiquitin-conjugating enzyme UBE2O (EC 2.3.2.24) (E2/E3 hybrid ubiquitin-protein ligase UBE2O) (Ubiquitin carrier protein O) (Ubiquitin-conjugating enzyme E2 O) (Ubiquitin-conjugating enzyme E2 of 230 kDa) (Ubiquitin-conjugating enzyme E2-230K) (Ubiquitin-protein ligase O)
Q6ZQ11	CHSS1_MOUSE	Chondroitin sulfate synthase 1 (EC 2.4.1.175) (EC 2.4.1.226) (Chondroitin glucuronyltransferase 1) (Chondroitin synthase 1) (ChSy-1) (Glucuronosyl-N-acetylgalactosaminyl-proteoglycan 4-beta-N-acetylgalactosaminyltransferase 1) (N-acetylgalactosaminyl-proteoglycan 3-beta-glucuronosyltransferase 1) (N-acetylgalactosaminyltransferase 1)
Q6ZQA6	IGSF3_MOUSE	Immunoglobulin superfamily member 3 (IgSF3)
Q6ZQB6	VIP2_MOUSE	Inositol hexakisphosphate and diphosphoinositol-pentakisphosphate kinase 2 (EC 2.7.4.21) (EC 2.7.4.24) (Diphosphoinositol pentakisphosphate kinase 2) (Histidine acid phosphatase domain-containing protein 1) (InsP6 and PP-IP5 kinase 2) (VIP1 homolog 2) (mmVIP2)
Q6ZQH8	NU188_MOUSE	Nucleoporin NUP188 homolog
Q6ZQK0	CNDD3_MOUSE	Condensin-2 complex subunit D3 (Non-SMC condensin II complex subunit D3)
Q6ZWR6	SYNE1_MOUSE	Nesprin-1 (Enaptin) (KASH domain-containing protein 1) (KASH1) (Myocyte nuclear envelope protein 1) (Myne-1) (Nuclear envelope spectrin repeat protein 1) (Synaptic nuclear envelope protein 1) (Syne-1)
Q6ZWU9	RS27_MOUSE	40S ribosomal protein S27
Q704Y3	TRPV1_MOUSE	Transient receptor potential cation channel subfamily V member 1 (TrpV1) (Osm-9-like TRP channel 1) (OTRPC1) (Vanilloid receptor 1)

Q75NR7	RECQ4_MOUSE	ATP-dependent DNA helicase Q4 (EC 3.6.4.12) (DNA helicase, RecQ-like type 4) (RecQ4) (RecQ protein-like 4)
Q78DX7	ROS1_MOUSE	Proto-oncogene tyrosine-protein kinase ROS (EC 2.7.10.1) (Proto-oncogene c-Ros) (Proto-oncogene c-Ros-1) (Receptor tyrosine kinase c-ros oncogene 1) (c-Ros receptor tyrosine kinase)
Q7JJ13	BRD2_MOUSE	Bromodomain-containing protein 2 (Female sterile homeotic-related protein 1) (Fsrg-1) (Protein RING3)
Q7M710	TR125_MOUSE	Taste receptor type 2 member 125 (T2R125) (mT2R59)
Q7SIG6	ASAP2_MOUSE	Arf-GAP with SH3 domain, ANK repeat and PH domain-containing protein 2 (Development and differentiation-enhancing factor 2) (Paxillin-associated protein with ARF GAP activity 3) (PAG3) (Pyk2 C-terminus-associated protein) (PAP)
Q7TMF2	ERI1_MOUSE	3'-5' exoribonuclease 1 (EC 3.1.-.-) (3'-5' exonuclease ERI1) (Eri-1 homolog) (Histone mRNA 3'-exonuclease 1)
Q7TN37	TRPM4_MOUSE	Transient receptor potential cation channel subfamily M member 4 (Calcium-activated non-selective cation channel 1) (Long transient receptor potential channel 4) (LTrpC-4) (LTrpC4)
Q7TN79	AKA7G_MOUSE	A-kinase anchor protein 7 isoform gamma (AKAP-7 isoform gamma) (A-kinase anchor protein 18) (AKAP-18) (Protein kinase A-anchoring protein 7 isoform gamma) (PRKA7 isoform gamma)
Q7TNC4	LC7L2_MOUSE	Putative RNA-binding protein Luc7-like 2 (CGI-74 homolog)
Q7TND5	RPF1_MOUSE	Ribosome production factor 1 (Brix domain-containing protein 5) (Ribosome biogenesis protein RPF1)
Q7TNR6	IGS21_MOUSE	Immunoglobulin superfamily member 21 (IgSF21)
Q7TPD2	F185A_MOUSE	Protein FAM185A
Q7TPH6	MYCB2_MOUSE	E3 ubiquitin-protein ligase MYCBP2 (EC 2.3.2.-) (Myc-binding protein 2) (Pam/highwire/rpm-1 protein) (Protein Magellan) (Protein associated with Myc)
Q7TPM1	PRC2B_MOUSE	Protein PRRC2B (HLA-B-associated transcript 2-like 1) (Proline-rich coiled-coil protein 2B)
Q7TPZ8	CBPA1_MOUSE	Carboxypeptidase A1 (EC 3.4.17.1)
Q7TQ32	RGMC_MOUSE	Hemojuvelin (Hemochromatosis type 2 protein homolog) (Hemojuvelin BMP coreceptor) (RGM domain family member C)

Q7TQA5	T2R39_MOUSE	Taste receptor type 2 member 39 (T2R39) (mT2R34)
Q7TQG1	PKHA6_MOUSE	Pleckstrin homology domain-containing family A member 6 (PH domain-containing family A member 6) (Phosphoinositol 3-phosphate-binding protein 3) (PEPP-3)
Q7TQP2	GP135_MOUSE	G-protein coupled receptor 135
Q7TSA6	PRSR3_MOUSE	Proline and serine-rich protein 3
Q7TSE6	ST38L_MOUSE	Serine/threonine-protein kinase 38-like (EC 2.7.11.1) (NDR2 protein kinase) (Nuclear Dbf2-related kinase 2)
Q7TSG2	CTDP1_MOUSE	RNA polymerase II subunit A C-terminal domain phosphatase (EC 3.1.3.16) (TFIIF-associating CTD phosphatase)
Q7TT23	CT194_MOUSE	Uncharacterized protein C20orf194 homolog
Q80SX8	PIF1_MOUSE	ATP-dependent DNA helicase PIF1 (EC 3.6.4.12) (DNA repair and recombination helicase PIF1) (Pif1/Rrm3 DNA helicase-like protein)
Q80TE4	SI1L2_MOUSE	Signal-induced proliferation-associated 1-like protein 2 (SIPA1-like protein 2)
Q80TG1	KANL1_MOUSE	KAT8 regulatory NSL complex subunit 1 (NSL complex protein NSL1) (Non-specific lethal 1 homolog)
Q80TI0	ASTRB_MOUSE	Protein Aster-B (GRAM domain-containing protein 1B)
Q80TK0	K1107_MOUSE	AP2-interacting clathrin-endocytosis protein (APache)
Q80TY0	FNBP1_MOUSE	Formin-binding protein 1 (Formin-binding protein 17)
Q80U28	MADD_MOUSE	MAP kinase-activating death domain protein (Rab3 GDP/GTP exchange factor)
Q80UF4	SDCG8_MOUSE	Serologically defined colon cancer antigen 8 homolog (Centrosomal colon cancer autoantigen protein) (mCCCAP)
Q80UN1	KCTD9_MOUSE	BTB/POZ domain-containing protein KCTD9
Q80UU9	PGRC2_MOUSE	Membrane-associated progesterone receptor component 2
Q80UW5	MRCKG_MOUSE	Serine/threonine-protein kinase MRCK gamma (EC 2.7.11.1) (CDC42-binding protein kinase gamma) (DMPK-like gamma) (Myotonic dystrophy kinase-related CDC42-binding kinase gamma) (MRCK gamma) (Myotonic dystrophy protein kinase-like gamma) (Myotonic dystrophy protein kinase-like alpha)

Q80UY2	KCMF1_MOUSE	E3 ubiquitin-protein ligase KCMF1 (EC 2.3.2.27) (Differentially expressed in branching tubulogenesis 91) (Debt-91) (RING-type E3 ubiquitin transferase KCMF1)
Q80VU4	NTF4_MOUSE	Neurotrophin-4 (NT-4) (Neurotrophin-5) (NT-5) (Neutrophic factor 4)
Q80W00	PP1RA_MOUSE	Serine/threonine-protein phosphatase 1 regulatory subunit 10 (MHC class I region proline-rich protein CAT53)
Q80WC3	TNC18_MOUSE	Trinucleotide repeat-containing gene 18 protein (Zinc finger protein 469)
Q80WC9	ACSF4_MOUSE	Beta-alanine-activating enzyme (EC 6.2.1.-) (Acyl-CoA synthetase family member 4) (Protein LYS2 homolog)
Q80X82	SYMPK_MOUSE	Symplekin
Q80YQ8	RMD5A_MOUSE	E3 ubiquitin-protein ligase RMND5A (EC 2.3.2.27) (Protein RMD5 homolog A)
Q80YR3	SKDA1_MOUSE	SKI/DACH domain-containing protein 1 (Protein DLN-1)
Q80YS5	LRC27_MOUSE	Leucine-rich repeat-containing protein 27
Q80YT5	SPT20_MOUSE	Spermatogenesis-associated protein 20 (Sperm-specific protein 411) (Ssp411) (Transcript increased in spermiogenesis 78 protein)
Q80YT7	MYOME_MOUSE	Myomegalin (Phosphodiesterase 4D-interacting protein)
Q80Z10	ASTN2_MOUSE	Astrotactin-2
Q80ZI1	TRNP1_MOUSE	TMF-regulated nuclear protein 1
Q810B9	SLIK3_MOUSE	SLIT and NTRK-like protein 3
Q810C0	SLIK2_MOUSE	SLIT and NTRK-like protein 2
Q811J3	IREB2_MOUSE	Iron-responsive element-binding protein 2 (IRE-BP 2) (Iron regulatory protein 2) (IRP2)
Q811S7	UBIP1_MOUSE	Upstream-binding protein 1 (Nuclear factor 2d9) (NF2d9)
Q812A2	SRGP3_MOUSE	SLIT-ROBO Rho GTPase-activating protein 3 (srGAP3) (Rho GTPase-activating protein 14) (WAVE-associated Rac GTPase-activating protein) (WRP)
Q812C9	AOC2_MOUSE	Retina-specific copper amine oxidase (RAO) (EC 1.4.3.21) (Amine oxidase [copper-containing])
Q8BFS9	ASND1_MOUSE	Asparagine synthetase domain-containing protein 1
Q8BFW7	LPP_MOUSE	Lipoma-preferred partner homolog
Q8BFW9	GTR12_MOUSE	Solute carrier family 2, facilitated glucose transporter member 12 (Glucose transporter type 12) (GLUT-12)

Q8BG02	2ABG_MOUSE	Serine/threonine-protein phosphatase 2A 55 kDa regulatory subunit B gamma isoform (PP2A subunit B isoform B55-gamma) (PP2A subunit B isoform PR55-gamma) (PP2A subunit B isoform R2-gamma) (PP2A subunit B isoform gamma)
Q8BG48	ST17B_MOUSE	Serine/threonine-protein kinase 17B (EC 2.7.11.1) (DAP kinase-related apoptosis-inducing protein kinase 2)
Q8BG55	GP171_MOUSE	Probable G-protein coupled receptor 171
Q8BG79	C19L2_MOUSE	CWF19-like protein 2
Q8BG84	LAIR1_MOUSE	Leukocyte-associated immunoglobulin-like receptor 1 (LAIR-1) (mLAIR1) (CD antigen CD305)
Q8BGD4	SO4C1_MOUSE	Solute carrier organic anion transporter family member 4C1 (Oatp-R) (Solute carrier family 21 member 20)
Q8BGD7	NPAS4_MOUSE	Neuronal PAS domain-containing protein 4 (Neuronal PAS4) (HLH-PAS transcription factor NXF) (Limbic-enhanced PAS protein) (LE-PAS)
Q8BGF6	ELMD2_MOUSE	ELMO domain-containing protein 2
Q8BGF9	S2544_MOUSE	Solute carrier family 25 member 44
Q8BGI7	LRC39_MOUSE	Leucine-rich repeat-containing protein 39 (Myosin-interacting M-band-associated stress-responsive protein) (Myomasp)
Q8BGQ1	SPE39_MOUSE	Spermatogenesis-defective protein 39 homolog (hSPE-39) (VPS33B-interacting protein in apical-basolateral polarity regulator) (VPS33B-interacting protein in polarity and apical restriction)
Q8BGT1	FLRT3_MOUSE	Leucine-rich repeat transmembrane protein FLRT3 (Fibronectin leucine rich transmembrane protein 3)
Q8BGX3	LRTM2_MOUSE	Leucine-rich repeat and transmembrane domain-containing protein 2
Q8BH50	CR025_MOUSE	Uncharacterized protein C18orf25 homolog
Q8BH82	NAPEP_MOUSE	N-acyl-phosphatidylethanolamine-hydrolyzing phospholipase D (N-acyl phosphatidylethanolamine phospholipase D) (NAPE-PLD) (NAPE-hydrolyzing phospholipase D) (EC 3.1.4.54)
Q8BH88	DEP1B_MOUSE	DEP domain-containing protein 1B
Q8BH89	WF15A_MOUSE	WAP four-disulfide core domain protein 15A
Q8BHA9	PXYP1_MOUSE	2-phosphoxylose phosphatase 1 (EC 3.1.3.-) (Acid phosphatase-like protein 2)
Q8BHK9	ERC6L_MOUSE	DNA excision repair protein ERCC-6-like (EC 3.6.4.12) (ATP-dependent helicase ERCC6-like)
Q8BHL3	TB10B_MOUSE	TBC1 domain family member 10B (Protein wz3-85)

Q8BHN3	GANAB_MOUSE	Neutral alpha-glucosidase AB (EC 3.2.1.207) (Alpha-glucosidase 2) (Glucosidase II subunit alpha)
Q8BHP2	NUTM1_MOUSE	NUT family member 1 (Nuclear protein in testis)
Q8BI21	RNF38_MOUSE	E3 ubiquitin-protein ligase RNF38 (EC 2.3.2.27) (RING finger protein 38) (RING-type E3 ubiquitin transferase RNF38)
Q8BIF2	RFOX3_MOUSE	RNA binding protein fox-1 homolog 3 (Fox-1 homolog C) (Hexaribonucleotide-binding protein 3) (Fox-3) (Neuronal nuclei antigen) (NeuN antigen)
Q8BIH0	SP130_MOUSE	Histone deacetylase complex subunit SAP130 (130 kDa Sin3-associated polypeptide) (Sin3-associated polypeptide p130)
Q8BIP0	SYDM_MOUSE	Aspartate--tRNA ligase, mitochondrial (EC 6.1.1.12) (Aspartyl-tRNA synthetase) (AspRS)
Q8BIQ5	CSTF2_MOUSE	Cleavage stimulation factor subunit 2 (CF-1 64 kDa subunit) (Cleavage stimulation factor 64 kDa subunit) (CSTF 64 kDa subunit) (CstF-64)
Q8BJ34	MARF1_MOUSE	Meiosis regulator and mRNA stability factor 1 (Limkain-b1) (Meiosis arrest female protein 1)
Q8BJS4	SUN2_MOUSE	SUN domain-containing protein 2 (Protein unc-84 homolog B) (Sad1/unc-84 protein-like 2)
Q8BJU0	SGTA_MOUSE	Small glutamine-rich tetratricopeptide repeat-containing protein alpha (Alpha-SGT)
Q8BK64	AHSA1_MOUSE	Activator of 90 kDa heat shock protein ATPase homolog 1 (AHA1)
Q8BK84	DUPD1_MOUSE	Dual specificity phosphatase DUPD1 (EC 3.1.3.16) (EC 3.1.3.48)
Q8BKE5	FSBP_MOUSE	Fibrinogen silencer-binding protein
Q8BKF1	RPOM_MOUSE	DNA-directed RNA polymerase, mitochondrial (MtRPOL) (EC 2.7.7.6)
Q8BL06	UBP54_MOUSE	Inactive ubiquitin carboxyl-terminal hydrolase 54 (Inactive ubiquitin-specific peptidase 54)
Q8BLA1	DLEC1_MOUSE	Deleted in lung and esophageal cancer protein 1 homolog
Q8BLH5	TSP50_MOUSE	Probable threonine protease PRSS50 (EC 3.4.25.-) (Serine protease 50) (Testis-specific protease-like protein 50)
Q8BLN5	ERG7_MOUSE	Lanosterol synthase (EC 5.4.99.7) (2,3-epoxysqualene--lanosterol cyclase) (Oxidosqualene--lanosterol cyclase) (OSC)
Q8BLS7	SWAHA_MOUSE	Ankyrin repeat domain-containing protein SOWAHA (Ankyrin repeat domain-containing protein 43) (Protein sosondowah homolog A)
Q8BLX4	FUCT1_MOUSE	GDP-fucose transporter 1 (Solute carrier family 35 member C1)

Q8BM65	NYAP2_MOUSE	Neuronal tyrosine-phosphorylated phosphoinositide-3-kinase adapter 2
Q8BM75	ARI5B_MOUSE	AT-rich interactive domain-containing protein 5B (ARID domain-containing protein 5B) (Developmentally and sexually retarded with transient immune abnormalities protein) (Desrt) (MRF1-like) (Modulator recognition factor protein 2) (MRF-2)
Q8BME9	CBLN4_MOUSE	Cerebellin-4 (Cerebellin-like glycoprotein 1)
Q8BMP6	GCP60_MOUSE	Golgi resident protein GCP60 (Acyl-CoA-binding domain-containing protein 3) (Golgi complex-associated protein 1) (GOCAP1) (Golgi phosphoprotein 1) (GOLPH1) (PBR- and PKA-associated protein 7) (Peripheral benzodiazepine receptor-associated protein PAP7)
Q8BNE1	TCAF1_MOUSE	TRPM8 channel-associated factor 1 (TRP channel-associated factor 1)
Q8BNN1	SPA2L_MOUSE	Spermatogenesis-associated protein 2-like protein (SPATA2-like protein)
Q8BPX9	S15A3_MOUSE	Solute carrier family 15 member 3 (Peptide transporter 3) (Peptide/histidine transporter 2) (cAMP-inducible gene 1 protein)
Q8BQQ1	ZDH14_MOUSE	Probable palmitoyltransferase ZDHHC14 (EC 2.3.1.225) (NEW1 domain-containing protein) (NEW1CP) (Zinc finger DHHC domain-containing protein 14) (DHHC-14)
Q8BRH4	KMT2C_MOUSE	Histone-lysine N-methyltransferase 2C (Lysine N-methyltransferase 2C) (EC 2.1.1.354) (Myeloid/lymphoid or mixed-lineage leukemia protein 3 homolog)
Q8BRJ3	RELL2_MOUSE	RELT-like protein 2
Q8BSF4	PISD_MOUSE	Phosphatidylserine decarboxylase proenzyme, mitochondrial (EC 4.1.1.65) [Cleaved into: Phosphatidylserine decarboxylase beta chain; Phosphatidylserine decarboxylase alpha chain]
Q8BTU7	RCCD1_MOUSE	RCC1 domain-containing protein 1
Q8BTY8	SCFD2_MOUSE	Sec1 family domain-containing protein 2 (Neuronal Sec1) (Syntaxin-binding protein 1-like 1)
Q8BTZ7	GMPPB_MOUSE	Mannose-1-phosphate guanyltransferase beta (EC 2.7.7.13) (GDP-mannose pyrophosphorylase B) (GTP-mannose-1-phosphate guanylyltransferase beta)
Q8BUM6	F163B_MOUSE	Protein FAM163B
Q8BUR4	DOCK1_MOUSE	Dedicator of cytokinesis protein 1 (180 kDa protein downstream of CRK) (DOCK180)

Q8BUR9	MZT1_MOUSE	Mitotic-spindle organizing protein 1 (Mitotic-spindle organizing protein associated with a ring of gamma-tubulin 1)
Q8BUV3	GEPH_MOUSE	Gephyrin [Includes: Molybdopterin adenylyltransferase (MPT adenylyltransferase) (EC 2.7.7.75) (Domain G); Molybdopterin molybdenumtransferase (MPT Mo-transferase) (EC 2.10.1.1) (Domain E)]
Q8BUY8	GASP2_MOUSE	G-protein coupled receptor-associated sorting protein 2 (GASP-2)
Q8BV49	IFIX_MOUSE	Pyrin and HIN domain-containing protein 1 (Interferon-inducible protein 209) (Ifi-209) (Interferon-inducible protein X) (Interferon-inducible protein p209)
Q8BVA5	LDAH_MOUSE	Lipid droplet-associated hydrolase (EC 3.1.1.-) (Lipid droplet-associated serine hydrolase) (mLDAH)
Q8BVL3	SNX17_MOUSE	Sorting nexin-17
Q8BVQ5	PPME1_MOUSE	Protein phosphatase methylesterase 1 (PME-1) (EC 3.1.1.89)
Q8BVU5	NUDT9_MOUSE	ADP-ribose pyrophosphatase, mitochondrial (EC 3.6.1.13) (ADP-ribose diphosphatase) (ADP-ribose phosphohydrolase) (Adenosine diphosphoribose pyrophosphatase) (ADPR-PPase) (Nucleoside diphosphate-linked moiety X motif 9) (Nudix motif 9)
Q8BVV7	CEP95_MOUSE	Centrosomal protein of 95 kDa (Cep95) (Coiled-coil domain-containing protein 45)
Q8BVW0	GANC_MOUSE	Neutral alpha-glucosidase C (EC 3.2.1.20)
Q8BVY0	RL1D1_MOUSE	Ribosomal L1 domain-containing protein 1
Q8BW56	GTDC1_MOUSE	Glycosyltransferase-like domain-containing protein 1
Q8BW66	S2548_MOUSE	Solute carrier family 25 member 48
Q8BWD8	CDK19_MOUSE	Cyclin-dependent kinase 19 (EC 2.7.11.22) (CDC2-related protein kinase 6) (Cell division cycle 2-like protein kinase 6) (Cell division protein kinase 19)
Q8BX90	FND3A_MOUSE	Fibronectin type-III domain-containing protein 3A
Q8BXL9	IFFO1_MOUSE	Intermediate filament family orphan 1
Q8BXR1	S7A14_MOUSE	Probable cationic amino acid transporter (Solute carrier family 7 member 14)
Q8BXR5	NALCN_MOUSE	Sodium leak channel non-selective protein (Voltage gated channel-like protein 1)
Q8BY71	HAT1_MOUSE	Histone acetyltransferase type B catalytic subunit (EC 2.3.1.48) (Histone acetyltransferase 1)

Q8BY79	S35G1_MOUSE	Solute carrier family 35 member G1 (Transmembrane protein 20)
Q8BYI9	TENR_MOUSE	Tenascin-R (TN-R) (Janusin) (Neural recognition molecule J1-160/180) (Restrictin)
Q8BYK8	ZC3H6_MOUSE	Zinc finger CCCH domain-containing protein 6
Q8BYN3	ITPK1_MOUSE	Inositol-tetrakisphosphate 1-kinase (EC 2.7.1.134) (Inositol 1,3,4-trisphosphate 5/6-kinase) (Inositol-triphosphate 5/6-kinase) (Ins(1,3,4)P(3) 5/6-kinase) (EC 2.7.1.159)
Q8BZ03	KPCD2_MOUSE	Serine/threonine-protein kinase D2 (EC 2.7.11.13) (nPKC-D2)
Q8BZ09	ODC_MOUSE	Mitochondrial 2-oxodicarboxylate carrier (Solute carrier family 25 member 21)
Q8BZ47	ZN609_MOUSE	Zinc finger protein 609
Q8BZB3	TM266_MOUSE	Transmembrane protein 266
Q8BZM0	KLH12_MOUSE	Kelch-like protein 12 (CUL3-interacting protein 1)
Q8BZX4	SREK1_MOUSE	Splicing regulatory glutamine/lysine-rich protein 1 (Serine/arginine-rich-splicing regulatory protein 86) (SRrp86) (Splicing factor, arginine/serine-rich 12)
Q8C010	GPR61_MOUSE	G-protein coupled receptor 61
Q8C078	KKCC2_MOUSE	Calcium/calmodulin-dependent protein kinase kinase 2 (CaM-KK 2) (CaM-kinase kinase 2) (CaMKK 2) (EC 2.7.11.17) (Calcium/calmodulin-dependent protein kinase kinase beta) (CaM-KK beta) (CaM-kinase kinase beta) (CaMKK beta)
Q8C0D5	EFL1_MOUSE	Elongation factor-like GTPase 1 (Elongation factor Tu GTP-binding domain-containing protein 1) (Elongation factor-like 1) (Protein FAM42A)
Q8C1B2	PARPT_MOUSE	Protein mono-ADP-ribosyltransferase TIPARP (EC 2.4.2.-) (ADP-ribosyltransferase diphtheria toxin-like 14) (ARTD14) (TCDD-inducible poly [ADP-ribose] polymerase)
Q8C2K1	DEFI6_MOUSE	Differentially expressed in FDCP 6 (DEF-6) (IRF4-binding protein) (SWAP-70-like adapter of T-cells)
Q8C3K6	SC5A1_MOUSE	Sodium/glucose cotransporter 1 (Na(+)/glucose cotransporter 1) (High affinity sodium-glucose cotransporter) (Solute carrier family 5 member 1)
Q8C4A5	ASXL3_MOUSE	Putative Polycomb group protein ASXL3 (Additional sex combs-like protein 3)
Q8C522	ENDD1_MOUSE	Endonuclease domain-containing 1 protein (EC 3.1.30.-)
Q8C5N3	CWC22_MOUSE	Pre-mRNA-splicing factor CWC22 homolog (Nucampholin homolog)

Q8C5S7	REC8_MOUSE	Meiotic recombination protein REC8 homolog (Cohesin Rec8p)
Q8C5U4	SPEM2_MOUSE	Uncharacterized protein SPEM2
Q8C6E0	CFA36_MOUSE	Cilia- and flagella-associated protein 36 (Coiled-coil domain-containing protein 104)
Q8C7H1	MMAA_MOUSE	Methylmalonic aciduria type A homolog, mitochondrial (EC 3.6.-.-)
Q8CB27	OTU1_MOUSE	Ubiquitin thioesterase OTU1 (EC 3.4.19.12)
Q8CBC6	LRRN3_MOUSE	Leucine-rich repeat neuronal protein 3 (Neuronal leucine-rich repeat protein 3) (NLRR-3)
Q8CBY8	DCTN4_MOUSE	Dynactin subunit 4 (Dynactin subunit p62)
Q8CC35	SYNPO_MOUSE	Synaptopodin
Q8CC88	VWA8_MOUSE	von Willebrand factor A domain-containing protein 8
Q8CCJ9	P20L1_MOUSE	PHD finger protein 20-like protein 1
Q8CCP0	NEMF_MOUSE	Nuclear export mediator factor Nemf (Serologically defined colon cancer antigen 1 homolog)
Q8CCT7	NSUN3_MOUSE	tRNA (cytosine(34)-C(5))-methyltransferase, mitochondrial (EC 2.1.1.-) (NOL1/NOP2/Sun domain family member 3)
Q8CDE2	CALI_MOUSE	Calicin
Q8CDM4	CCD73_MOUSE	Coiled-coil domain-containing protein 73
Q8CDV0	CC178_MOUSE	Coiled-coil domain-containing protein 178
Q8CE72	CPLN1_MOUSE	Ciliogenesis and planar polarity effector 1 (Protein C5orf42 homolog) (Protein JBTS17)
Q8CEZ1	SMCO1_MOUSE	Single-pass membrane and coiled-coil domain-containing protein 1 (Single-pass membrane protein with coiled-coil domains 1)
Q8CF25	CS067_MOUSE	UPF0575 protein C19orf67 homolog
Q8CFI7	RPB2_MOUSE	DNA-directed RNA polymerase II subunit RPB2 (EC 2.7.7.6) (DNA-directed RNA polymerase II 140 kDa polypeptide) (DNA-directed RNA polymerase II subunit B) (RNA polymerase II subunit 2) (RNA polymerase II subunit B2)
Q8CFT2	SET1B_MOUSE	Histone-lysine N-methyltransferase SETD1B (EC 2.1.1.354) (SET domain-containing protein 1B)
Q8CG80	SHF_MOUSE	SH2 domain-containing adapter protein F
Q8CGA0	PPM1F_MOUSE	Protein phosphatase 1F (EC 3.1.3.16) (Ca(2+)/calmodulin-dependent protein kinase phosphatase) (CaM-kinase phosphatase) (CaMKPase)
Q8CGB6	TNS2_MOUSE	Tensin-2 (EC 3.1.3.-) (C1 domain-containing phosphatase and tensin homolog) (C1-TEN) (Tensin-like C1 domain-containing phosphatase)

Q8CGM1	AGRB2_MOUSE	Adhesion G protein-coupled receptor B2 (Brain-specific angiogenesis inhibitor 2)
Q8CH40	NUDT6_MOUSE	Nucleoside diphosphate-linked moiety X motif 6 (Nudix motif 6) (EC 3.6.1.-) (Antisense basic fibroblast growth factor B)
Q8CHC4	SYNJ1_MOUSE	Synaptojanin-1 (EC 3.1.3.36) (Synaptic inositol 1,4,5-trisphosphate 5-phosphatase 1)
Q8CHP8	PGP_MOUSE	Glycerol-3-phosphate phosphatase (G3PP) (EC 3.1.3.21) (Aspartate-based ubiquitous Mg(2+)-dependent phosphatase) (AUM) (EC 3.1.3.48) (Phosphoglycolate phosphatase) (PGP)
Q8CHU3	EPN2_MOUSE	Epsin-2 (EPS-15-interacting protein 2) (Intersectin-EH-binding protein 2) (Ibp2)
Q8CHW4	EI2BE_MOUSE	Translation initiation factor eIF-2B subunit epsilon (eIF-2B GDP-GTP exchange factor subunit epsilon)
Q8CHY6	P66A_MOUSE	Transcriptional repressor p66 alpha (GATA zinc finger domain-containing protein 2A)
Q8CI17	MB213_MOUSE	Protein mab-21-like 3
Q8CIV7	OVOL2_MOUSE	Transcription factor Ovo-like 2 (mOvo2) (Zinc finger OVO2) (Zinc finger protein 339) (Zinc finger protein mOVO)
Q8CJF7	ELYS_MOUSE	Protein ELYS (Embryonic large molecule derived from yolk sac) (Protein MEL-28) (Putative AT-hook-containing transcription factor 1)
Q8JZM4	DNER_MOUSE	Delta and Notch-like epidermal growth factor-related receptor (Brain EGF repeat-containing transmembrane protein)
Q8JZP2	SYN3_MOUSE	Synapsin-3 (Synapsin III)
Q8JZQ2	AFG32_MOUSE	AFG3-like protein 2 (EC 3.4.24.-)
Q8JZX4	SPF45_MOUSE	Splicing factor 45 (45 kDa-splicing factor) (RNA-binding motif protein 17)
Q8K010	OPLA_MOUSE	5-oxoprolinase (EC 3.5.2.9) (5-oxo-L-prolinase) (5-OPase) (Pyroglutamase)
Q8K1C7	MOT14_MOUSE	Monocarboxylate transporter 14 (MCT 14) (Solute carrier family 16 member 14)
Q8K1H1	TDRD7_MOUSE	Tudor domain-containing protein 7 (PCTAIRE2-binding protein) (Tudor repeat associator with PCTAIRE-2) (Trap)
Q8K1I3	SPP24_MOUSE	Secreted phosphoprotein 24 (Spp-24) (Secreted phosphoprotein 2)
Q8K1S5	KLF11_MOUSE	Krueppel-like factor 11 (TGFB-inducible early growth response protein 2b) (Transforming growth factor-beta-inducible early growth response protein

		3) (TGFB-inducible early growth response protein 3) (TIEG-3)
Q8K1X1	WDR11_MOUSE	WD repeat-containing protein 11 (Bromodomain and WD repeat-containing protein 2)
Q8K212	PACS1_MOUSE	Phosphofurin acidic cluster sorting protein 1 (PACS-1)
Q8K248	HPDL_MOUSE	4-hydroxyphenylpyruvate dioxygenase-like protein (EC 1.13.-.-) (Glyoxalase domain-containing protein 1)
Q8K268	ABCF3_MOUSE	ATP-binding cassette sub-family F member 3
Q8K299	SCAR5_MOUSE	Scavenger receptor class A member 5
Q8K2D0	F199X_MOUSE	Protein FAM199X
Q8K2F8	LS14A_MOUSE	Protein LSM14 homolog A (Protein FAM61A) (RNA-associated protein 55A) (mRAP55A)
Q8K2Q9	SHOT1_MOUSE	Shootin-1 (Shootin1)
Q8K2Y9	CCM2_MOUSE	Cerebral cavernous malformations protein 2 homolog (Malcavernin) (Osmosensing scaffold for MEKK3)
Q8K2Z8	UB2Q2_MOUSE	Ubiquitin-conjugating enzyme E2 Q2 (EC 2.3.2.23) (E2 ubiquitin-conjugating enzyme Q2) (Ubiquitin carrier protein Q2) (Ubiquitin-protein ligase Q2)
Q8K375	LRC56_MOUSE	Leucine-rich repeat-containing protein 56
Q8K389	CK5P2_MOUSE	CDK5 regulatory subunit-associated protein 2 (CDK5 activator-binding protein C48)
Q8K3A9	MEPCE_MOUSE	7SK snRNA methylphosphate capping enzyme (MePCE) (EC 2.1.1.-)
Q8K3F6	KCNQ3_MOUSE	Potassium voltage-gated channel subfamily KQT member 3 (KQT-like 3) (Potassium channel subunit alpha KvLQT3) (Voltage-gated potassium channel subunit Kv7.3)
Q8K3I9	GLCI1_MOUSE	Glucocorticoid-induced transcript 1 protein (Glucocorticoid-induced gene 18 protein) (Testhymin) (Thymocyte/spermatocyte selection protein 1)
Q8K3P0	DR9C7_MOUSE	Short-chain dehydrogenase/reductase family 9C member 7 (EC 1.1.1.-) (Orphan short-chain dehydrogenase/reductase) (SDR-O) (RDH-S)
Q8K3W0	BABA2_MOUSE	BRISC and BRCA1-A complex member 2 (BRCA1-A complex subunit BRE) (BRCA1/BRCA2-containing complex subunit 45) (Brain and reproductive organ-expressed protein)
Q8K411	PREP_MOUSE	Presequence protease, mitochondrial (EC 3.4.24.-) (Pitrilysin metalloproteinase 1)
Q8K440	ABC8B_MOUSE	ATP-binding cassette sub-family A member 8-B
Q8K448	ABCA5_MOUSE	ATP-binding cassette sub-family A member 5

Q8K4B0	MTA1_MOUSE	Metastasis-associated protein MTA1
Q8K4K3	TRIB2_MOUSE	Tribbles homolog 2 (TRB-2)
Q8K4L3	SVIL_MOUSE	Supervillin (Archvillin) (p205/p250)
Q8K4Q0	RPTOR_MOUSE	Regulatory-associated protein of mTOR (Raptor) (p150 target of rapamycin (TOR)-scaffold protein)
Q8K592	AMHR2_MOUSE	Anti-Muellerian hormone type-2 receptor (EC 2.7.11.30) (Anti-Muellerian hormone type II receptor) (AMH type II receptor) (MIS type II receptor) (MISRII) (MRII)
Q8R0Y6	AL1L1_MOUSE	Cytosolic 10-formyltetrahydrofolate dehydrogenase (10-FTHFDH) (FDH) (EC 1.5.1.6) (Aldehyde dehydrogenase family 1 member L1)
Q8R1C0	KCNC4_MOUSE	Potassium voltage-gated channel subfamily C member 4 (Voltage-gated potassium channel subunit Kv3.4)
Q8R1C6	MET22_MOUSE	Methyltransferase-like protein 22 (EC 2.1.1.-)
Q8R1G1	TASP1_MOUSE	Threonine aspartase 1 (Taspase-1) (EC 3.4.25.-) [Cleaved into: Threonine aspartase subunit alpha; Threonine aspartase subunit beta]
Q8R1G2	CMBL_MOUSE	Carboxymethylenebutenolidase homolog (EC 3.1.-.-)
Q8R2N1	AQP3_MOUSE	Aquaporin-3 (AQP-3) (Aquaglyceroporin-3)
Q8R2R9	AP3M2_MOUSE	AP-3 complex subunit mu-2 (Adaptor-related protein complex 3 subunit mu-2) (Clathrin assembly protein assembly protein complex 3 mu-2 medium chain) (Clathrin coat assembly protein AP47 homolog 2) (Clathrin coat-associated protein AP47 homolog 2) (Golgi adaptor AP-1 47 kDa protein homolog 2) (HA1 47 kDa subunit homolog 2) (Mu3B-adaptin) (m3B) (P47B)
Q8R2Z5	VWA1_MOUSE	von Willebrand factor A domain-containing protein 1 (von Willebrand factor A domain-related protein)
Q8R3C1	CB042_MOUSE	Uncharacterized protein C2orf42 homolog
Q8R3N2	MFS6L_MOUSE	Major facilitator superfamily domain-containing protein 6-like
Q8R3S6	EXOC1_MOUSE	Exocyst complex component 1 (Exocyst complex component Sec3)
Q8R3Y5	CS047_MOUSE	Uncharacterized protein C19orf47 homolog
Q8R4D1	SL9A8_MOUSE	Sodium/hydrogen exchanger 8 (Na(+)/H(+) exchanger 8) (NHE-8) (Solute carrier family 9 member 8)
Q8R554	OTU7A_MOUSE	OTU domain-containing protein 7A (EC 3.4.19.12) (Zinc finger protein Cezanne 2)

Q8R555	CRAC1_MOUSE	Cartilage acidic protein 1 (68 kDa chondrocyte-expressed protein) (CEP-68) (ASPIC) (Protein CRTAC1-B)
Q8R5F8	ES8L1_MOUSE	Epidermal growth factor receptor kinase substrate 8-like protein 1 (EPS8-like protein 1) (Epidermal growth factor receptor pathway substrate 8-related protein 1) (EPS8-related protein 1)
Q8R5K4	NOL6_MOUSE	Nucleolar protein 6 (Nucleolar RNA-associated protein) (Nrap)
Q8VBT6	APOBR_MOUSE	Apolipoprotein B receptor (Apolipoprotein B-100 receptor) (Apolipoprotein B48 receptor) (apoB-48R)
Q8VC60	GLB1L_MOUSE	Beta-galactosidase-1-like protein (EC 3.2.1.-)
Q8VCC6	CCM2L_MOUSE	Cerebral cavernous malformations 2 protein-like (CCM2-like)
Q8VCE1	DJC28_MOUSE	DnaJ homolog subfamily C member 28
Q8VCE9	PKHH3_MOUSE	Pleckstrin homology domain-containing family H member 3 (PH domain-containing family H member 3)
Q8VCH2	CLM5_MOUSE	CMRF35-like molecule 5 (CLM-5) (CD300 antigen like family member D) (Leukocyte mono-Ig-like receptor 4) (Myeloid-associated immunoglobulin-like receptor 4) (MAIR-4) (MAIR-IV)
Q8VCK3	TBG2_MOUSE	Tubulin gamma-2 chain (Gamma-2-tubulin)
Q8VCX6	KPTN_MOUSE	KICSTOR complex protein kaptin
Q8VCZ9	HYPDH_MOUSE	Hydroxyproline dehydrogenase (HYPDH) (EC 1.5.5.3) (Kidney and liver proline oxidase 1) (MmPOX1) (Probable proline dehydrogenase 2) (EC 1.5.5.2) (Probable proline oxidase 2) (Proline oxidase-like protein) (PO) (Proline oxidase)
Q8VD33	SGTB_MOUSE	Small glutamine-rich tetratricopeptide repeat-containing protein beta (Beta-SGT)
Q8VD57	SFT2B_MOUSE	Vesicle transport protein SFT2B (SFT2 domain-containing protein 2)
Q8VD65	PI3R4_MOUSE	Phosphoinositide 3-kinase regulatory subunit 4 (PI3-kinase regulatory subunit 4) (EC 2.7.11.1)
Q8VD72	TTC8_MOUSE	Tetratricopeptide repeat protein 8 (TPR repeat protein 8) (Bardet-Biedl syndrome 8 protein homolog)
Q8VDC0	SYLM_MOUSE	Probable leucine--tRNA ligase, mitochondrial (EC 6.1.1.4) (Leucyl-tRNA synthetase) (LeuRS)
Q8VDK1	NIT1_MOUSE	Deaminated glutathione amidase (dGSH amidase) (EC 3.5.1.128) (Nitrilase homolog 1)

Q8VDQ1	PTGR2_MOUSE	Prostaglandin reductase 2 (PRG-2) (EC 1.3.1.48) (15-oxoprostaglandin 13-reductase) (Zinc-binding alcohol dehydrogenase domain-containing protein 1)
Q8VDS7	CE57L_MOUSE	Centrosomal protein CEP57L1 (Centrosomal protein 57kDa-like protein 1) (Centrosomal protein of 57 kDa-related protein) (Cep57R) (Cep57-related protein)
Q8VDT1	SC5A9_MOUSE	Sodium/glucose cotransporter 4 (Na(+)/glucose cotransporter 4) (mSGLT4) (Solute carrier family 5 member 9)
Q8VE38	OXND1_MOUSE	Oxidoreductase NAD-binding domain-containing protein 1 (EC 1.-.-.-)
Q8VEJ1	GPN2_MOUSE	GPN-loop GTPase 2 (ATP-binding domain 1 family member B)
Q8VEJ4	NLE1_MOUSE	Notchless protein homolog 1
Q8VFB9	OL183_MOUSE	Olfactory receptor 183 (Olfactory receptor 183-2)
Q8VG13	OL507_MOUSE	Olfactory receptor 507 (Olfactory receptor 204-7)
Q8VHY0	CSPG4_MOUSE	Chondroitin sulfate proteoglycan 4 (Chondroitin sulfate proteoglycan NG2) (Proteoglycan AN2)
Q8VI47	MRP2_MOUSE	Canalicular multispecific organic anion transporter 1 (ATP-binding cassette sub-family C member 2) (Multidrug resistance-associated protein 2) (EC 7.6.2.2)
Q8VI59	PCX3_MOUSE	Pecanex-like protein 3 (Pecanex homolog protein 3)
Q8VI93	OAS3_MOUSE	2'-5'-oligoadenylate synthase 3 ((2-5')oligo(A) synthase 3) (2-5A synthase 3) (EC 2.7.7.84) (2',5'-oligoadenylate synthetase-like 10)
Q8VI94	OASL1_MOUSE	2'-5'-oligoadenylate synthase-like protein 1 (2',5'-oligoadenylate synthetase-like 9)
Q8VIG0	ZCH14_MOUSE	Zinc finger CCHC domain-containing protein 14 (BDG-29)
Q8VIM9	IRGQ_MOUSE	Immunity-related GTPase family Q protein
Q91UZ4	EGLN3_MOUSE	Egl nine homolog 3 (EC 1.14.11.29) (Hypoxia-inducible factor prolyl hydroxylase 3) (HIF-PH3) (HIF-prolyl hydroxylase 3) (HPH-3) (Prolyl hydroxylase domain-containing protein 3) (PHD3) (SM-20)
Q91V93	RHBT2_MOUSE	Rho-related BTB domain-containing protein 2 (Deleted in breast cancer 2 gene protein homolog)
Q91VA6	PDIP2_MOUSE	Polymerase delta-interacting protein 2
Q91VC3	IF4A3_MOUSE	Eukaryotic initiation factor 4A-III (eIF-4A-III) (eIF4A-III) (EC 3.6.4.13) (ATP-dependent RNA helicase DDX48) (ATP-dependent RNA helicase

		eIF4A-3) (DEAD box protein 48) (Eukaryotic translation initiation factor 4A isoform 3) [Cleaved into: Eukaryotic initiation factor 4A-III, N-terminally processed]
Q91VE3	KLK7_MOUSE	Kallikrein-7 (EC 3.4.21.117) (Serine protease 6) (Stratum corneum chymotryptic enzyme) (Thymopsin)
Q91W86	VPS11_MOUSE	Vacuolar protein sorting-associated protein 11 homolog
Q91WC0	SETD3_MOUSE	Actin-histidine N-methyltransferase (EC 2.1.1.85) (Endothelial differentiation inhibitory protein D10) (SET domain-containing protein 3)
Q91WC1	POTE1_MOUSE	Protection of telomeres protein 1 (mPot1) (POT1-like telomere end-binding protein)
Q91WD1	RPC4_MOUSE	DNA-directed RNA polymerase III subunit RPC4 (RNA polymerase III subunit C4) (DNA-directed RNA polymerase III subunit D)
Q91WJ8	FUBP1_MOUSE	Far upstream element-binding protein 1 (FBP) (FUSE-binding protein 1)
Q91WM2	HDHD5_MOUSE	Haloacid dehalogenase-like hydrolase domain-containing 5 (Cat eye syndrome critical region protein 5 homolog)
Q91WT9	CBS_MOUSE	Cystathionine beta-synthase (EC 4.2.1.22) (Beta-thionase) (Serine sulfhydrase)
Q91X21	K2013_MOUSE	Uncharacterized protein KIAA2013
Q91X43	SH319_MOUSE	SH3 domain-containing protein 19 (Kryn)
Q91X52	DCXR_MOUSE	L-xylulose reductase (XR) (EC 1.1.1.10) (Dicarbonyl/L-xylulose reductase)
Q91X83	METK1_MOUSE	S-adenosylmethionine synthase isoform type-1 (AdoMet synthase 1) (EC 2.5.1.6) (Methionine adenosyltransferase 1) (MAT 1)
Q91X85	FLVC2_MOUSE	Feline leukemia virus subgroup C receptor-related protein 2 (Calcium-chelate transporter) (CCT)
Q91YD4	TRPM2_MOUSE	Transient receptor potential cation channel subfamily M member 2 (Long transient receptor potential channel 2) (LTrpC-2) (LTrpC2) (Transient receptor potential channel 7) (TrpC7)
Q91YE5	BAZ2A_MOUSE	Bromodomain adjacent to zinc finger domain protein 2A (Transcription termination factor I-interacting protein 5) (TTF-I-interacting protein 5) (Tip5)
Q91YI1	ATG13_MOUSE	Autophagy-related protein 13
Q91YJ2	SNX4_MOUSE	Sorting nexin-4

Q91YM4	FAKD4_MOUSE	FAST kinase domain-containing protein 4 (Protein TBRG4) (Transforming growth factor beta regulator 4)
Q91YN5	UAP1_MOUSE	UDP-N-acetylhexosamine pyrophosphorylase [Includes: UDP-N-acetylgalactosamine pyrophosphorylase (EC 2.7.7.83); UDP-N-acetylglucosamine pyrophosphorylase (EC 2.7.7.23)]
Q91YP2	NEUL_MOUSE	Neurolysin, mitochondrial (EC 3.4.24.16) (Microsomal endopeptidase) (MEP) (Mitochondrial oligopeptidase M) (Neurotensin endopeptidase)
Q91YX0	THMS2_MOUSE	Protein THEMIS2 (Induced by contact to basement membrane 1 protein) (Protein ICB-1) (Thymocyte-expressed molecule involved in selection protein 2)
Q91Z96	BMP2K_MOUSE	BMP-2-inducible protein kinase (BIKe) (EC 2.7.11.1)
Q91ZA8	NRARP_MOUSE	Notch-regulated ankyrin repeat-containing protein
Q91ZC5	MRGA7_MOUSE	Mas-related G-protein coupled receptor member A7
Q921B4	NRIF2_MOUSE	Neurotrophin receptor-interacting factor 2 (Zinc finger protein 369)
Q921C5	BICD2_MOUSE	Protein bicaudal D homolog 2 (Bic-D 2)
Q921G6	LRCH4_MOUSE	Leucine-rich repeat and calponin homology domain-containing protein 4
Q921I6	SH3B4_MOUSE	SH3 domain-binding protein 4
Q921R8	S41A3_MOUSE	Solute carrier family 41 member 3
Q922B1	MACD1_MOUSE	ADP-ribose glycohydrolase MACROD1 (MACRO domain-containing protein 1) (O-acetyl-ADP-ribose deacetylase MACROD1) (EC 3.1.1.106) (Protein LRP16) ([Protein ADP-ribosylaspartate] hydrolase MACROD1) (EC 3.2.2.-) ([Protein ADP-ribosylglutamate] hydrolase MACROD1) (EC 3.2.2.-)
Q922Q2	RIOK1_MOUSE	Serine/threonine-protein kinase RIO1 (EC 2.7.11.1) (EC 3.6.3.-) (RIO kinase 1)
Q922Q8	LRC59_MOUSE	Leucine-rich repeat-containing protein 59 [Cleaved into: Leucine-rich repeat-containing protein 59, N-terminally processed]
Q923U0	TM1L1_MOUSE	TOM1-like protein 1 (Src-activating and signaling molecule protein) (Target of Myb-like protein 1)
Q925F2	ESAM_MOUSE	Endothelial cell-selective adhesion molecule
Q96EQ9	PRDM9_MOUSE	Histone-lysine N-methyltransferase PRDM9 (EC 2.1.1.-) (Hybrid sterility protein 1) (Meiosis-induced factor containing a PR/SET domain and zinc-finger motif) (PR domain zinc finger protein

		9) (PR domain-containing protein 9) (Protein-lysine N-methyltransferase PRDM9) (EC 2.1.1.-)
Q99J83	ATG5_MOUSE	Autophagy protein 5 (APG5-like)
Q99JF5	MVD1_MOUSE	Diphosphomevalonate decarboxylase (EC 4.1.1.33) (Mevalonate (diphospho)decarboxylase) (MDDase) (Mevalonate pyrophosphate decarboxylase)
Q99JF8	PSIP1_MOUSE	PC4 and SFRS1-interacting protein (Lens epithelium-derived growth factor) (mLEDGF)
Q99JT6	TLCD1_MOUSE	TLC domain-containing protein 1 (Calfacilitin)
Q99JX3	GORS2_MOUSE	Golgi reassembly-stacking protein 2 (GRS2) (Golgi reassembly-stacking protein of 55 kDa) (GRASP55)
Q99K48	NONO_MOUSE	Non-POU domain-containing octamer-binding protein (NonO protein)
Q99KE3	DOK4_MOUSE	Docking protein 4 (Downstream of tyrosine kinase 4)
Q99KF0	CAR14_MOUSE	Caspase recruitment domain-containing protein 14 (Bcl10-interacting MAGUK protein 2) (Bimp2)
Q99KN9	EPN4_MOUSE	Clathrin interactor 1 (Enthoprotin) (Epsin-4) (Epsin-related protein) (EpsinR)
Q99LB7	SARDH_MOUSE	Sarcosine dehydrogenase, mitochondrial (SarDH) (EC 1.5.8.3)
Q99LP6	GRPE1_MOUSE	GrpE protein homolog 1, mitochondrial (Mt-GrpE#1)
Q99LP8	YIPF2_MOUSE	Protein YIPF2 (YIP1 family member 2)
Q99LQ1	MBIP1_MOUSE	MAP3K12-binding inhibitory protein 1 (MAPK upstream kinase-binding inhibitory protein) (MUK-binding inhibitory protein)
Q99M51	NCK1_MOUSE	Cytoplasmic protein NCK1 (NCK adaptor protein 1) (Nck-1)
Q99M87	DNJA3_MOUSE	DnaJ homolog subfamily A member 3, mitochondrial (DnaJ protein Tid-1) (mTid-1) (Tumorous imaginal discs protein Tid56 homolog)
Q99MI6	GIMA3_MOUSE	GTPase IMAP family member 3 (Immunity-associated nucleotide 4 protein) (IAN-4)
Q99MQ3	PINK1_MOUSE	Serine/threonine-protein kinase PINK1, mitochondrial (EC 2.7.11.1) (BRPK) (PTEN-induced putative kinase protein 1)
Q99MR6	SRRT_MOUSE	Serrate RNA effector molecule homolog (Arsenite-resistance protein 2)
Q99MR8	MCCA_MOUSE	Methylcrotonoyl-CoA carboxylase subunit alpha, mitochondrial (MCCase subunit alpha) (EC 6.4.1.4) (3-methylcrotonyl-CoA carboxylase 1) (3-methylcrotonyl-CoA carboxylase biotin-containing

		subunit) (3-methylcrotonyl-CoA:carbon dioxide ligase subunit alpha)
Q99MV1	TDRD1_MOUSE	Tudor domain-containing protein 1
Q99MV5	M10L1_MOUSE	RNA helicase Mov10l1 (EC 3.6.4.13) (Cardiac helicase activated by MEF2 protein) (Cardiac-specific RNA helicase) (Moloney leukemia virus 10-like protein 1 homolog) (MOV10-like protein 1 homolog)
Q99N42	TYPH_MOUSE	Thymidine phosphorylase (TP) (EC 2.4.2.4) (TdRPase)
Q99NE5	RIMS1_MOUSE	Regulating synaptic membrane exocytosis protein 1 (Rab-3-interacting molecule 1) (RIM 1) (Rab-3-interacting protein 1)
Q99NF8	RBP17_MOUSE	Ran-binding protein 17
Q99P21	MUTYH_MOUSE	Adenine DNA glycosylase (EC 3.2.2.31) (MutY homolog) (mMYH)
Q99PI8	RTN4R_MOUSE	Reticulon-4 receptor (Nogo receptor) (NgR) (Nogo-66 receptor) (Nogo66 receptor-1) (NgR1)
Q99PP2	ZN318_MOUSE	Zinc finger protein 318 (Testicular zinc finger protein)
Q99PR8	HSPB2_MOUSE	Heat shock protein beta-2 (HspB2)
Q9CPV4	GLOD4_MOUSE	Glyoxalase domain-containing protein 4
Q9CQ35	GRPL2_MOUSE	GLIPR1-like protein 2
Q9CQF3	CPSF5_MOUSE	Cleavage and polyadenylation specificity factor subunit 5 (Nucleoside diphosphate-linked moiety X motif 21) (Nudix motif 21) (Nudix hydrolase 21)
Q9CQI4	CK074_MOUSE	Protein C11orf74 homolog (Protein NWC)
Q9CQJ4	RING2_MOUSE	E3 ubiquitin-protein ligase RING2 (EC 2.3.2.27) (RING finger protein 1B) (RING1b) (RING finger protein 2) (RING-type E3 ubiquitin transferase RING2)
Q9CQK7	RWDD1_MOUSE	RWD domain-containing protein 1 (DRG family-regulatory protein 2) (IH1)
Q9CQL0	MT21A_MOUSE	Protein N-lysine methyltransferase METTL21A (EC 2.1.1.-) (Methyltransferase-like protein 21A)
Q9CQM1	CA210_MOUSE	Type III endosome membrane protein TEMP (TEMP)
Q9CQR2	RS21_MOUSE	40S ribosomal protein S21
Q9CQT1	MTNA_MOUSE	Methylthioribose-1-phosphate isomerase (M1Pi) (MTR-1-P isomerase) (EC 5.3.1.23) (S-methyl-5-thioribose-1-phosphate isomerase) (Translation initiation factor eIF-2B subunit alpha/beta/delta-like protein)
Q9CR00	PSMD9_MOUSE	26S proteasome non-ATPase regulatory subunit 9 (26S proteasome regulatory subunit p27)

Q9CR14	FANCL_MOUSE	E3 ubiquitin-protein ligase FANCL (EC 2.3.2.27) (Fanconi anemia group L protein homolog) (Proliferation of germ cells protein) (RING-type E3 ubiquitin transferase FANCL)
Q9CR23	TMEM9_MOUSE	Transmembrane protein 9
Q9CRY7	GDPD1_MOUSE	Lysophospholipase D GDPD1 (EC 3.1.4.39) (Glycerophosphodiester phosphodiesterase 4) (Glycerophosphodiester phosphodiesterase domain-containing protein 1)
Q9CW07	PP13G_MOUSE	Protein phosphatase 1 regulatory subunit 3G
Q9CW46	RAVR1_MOUSE	Ribonucleoprotein PTB-binding 1 (Protein raver-1)
Q9CW73	B3GA1_MOUSE	Galactosylgalactosylxylosylprotein 3-beta-glucuronosyltransferase 1 (EC 2.4.1.135) (Beta-1,3-glucuronyltransferase 1) (Glucuronosyltransferase P) (GlcAT-P) (UDP-GlcUA:glycoprotein beta-1,3-glucuronyltransferase) (GlcUAT-P)
Q9CWE6	OOEP_MOUSE	Oocyte-expressed protein homolog (Factor located in oocytes permitting embryonic development) (Floped) (Oocyte- and embryo-specific protein 19) (mOEP19) (STAT3 downstream gene and differentiation regulator)
Q9CWL2	CASZ1_MOUSE	Zinc finger protein castor homolog 1 (Castor-related protein)
Q9CWL8	CTBL1_MOUSE	Beta-catenin-like protein 1 (Nuclear-associated protein) (NAP)
Q9CWM4	PFD1_MOUSE	Prefoldin subunit 1
Q9CWP6	MSPD2_MOUSE	Motile sperm domain-containing protein 2
Q9CWQ0	DPH5_MOUSE	Diphthine methyl ester synthase (EC 2.1.1.314) (Diphthamide biosynthesis methyltransferase)
Q9CWR7	STEA1_MOUSE	Metalloreductase STEAP1 (EC 1.16.1.-) (Six-transmembrane epithelial antigen of prostate 1)
Q9CWU5	KHDC3_MOUSE	KH domain-containing protein 3 (Protein Filia)
Q9CX13	CNIH4_MOUSE	Protein cornichon homolog 4 (CNIH-4) (Cornichon family AMPA receptor auxiliary protein 4)
Q9CX30	YIF1B_MOUSE	Protein YIF1B (YIP1-interacting factor homolog B)
Q9CXI5	MANF_MOUSE	Mesencephalic astrocyte-derived neurotrophic factor (Arginine-rich protein) (Protein ARMET)
Q9CXY6	ILF2_MOUSE	Interleukin enhancer-binding factor 2 (Nuclear factor of activated T-cells 45 kDa)
Q9CYK1	SYWM_MOUSE	Tryptophan--tRNA ligase, mitochondrial (EC 6.1.1.2) ((Mt)TrpRS) (Tryptophanyl-tRNA synthetase) (TrpRS)
Q9CYZ8	SSBP2_MOUSE	Single-stranded DNA-binding protein 2 (Sequence-specific single-stranded-DNA-binding protein 2)

Q9CZ69	CKLF6_MOUSE	CKLF-like MARVEL transmembrane domain-containing protein 6 (Chemokine-like factor superfamily member 6)
Q9CZB6	MED7_MOUSE	Mediator of RNA polymerase II transcription subunit 7 (Cofactor required for Sp1 transcriptional activation subunit 9) (CRSP complex subunit 9) (Mediator complex subunit 7)
Q9CZR2	NALD2_MOUSE	N-acetylated-alpha-linked acidic dipeptidase 2 (EC 3.4.17.21) (Glutamate carboxypeptidase III) (GCPIII) (N-acetylaspartylglutamate peptidase II) (NAAG-peptidase II) (N-acetylated-alpha-linked acidic dipeptidase II) (NAALADase II)
Q9CZU3	MTREX_MOUSE	Exosome RNA helicase MTR4 (EC 3.6.4.13) (ATP-dependent helicase SKIV2L2) (Superkiller viralicidic activity 2-like 2) (TRAMP-like complex helicase)
Q9D020	5NT3A_MOUSE	Cytosolic 5'-nucleotidase 3A (EC 3.1.3.5) (7-methylguanosine phosphate-specific 5'-nucleotidase) (7-methylguanosine nucleotidase) (EC 3.1.3.91) (Cytosolic 5'-nucleotidase 3) (Cytosolic 5'-nucleotidase III) (cN-III) (Lupin) (Pyrimidine 5'-nucleotidase 1) (P5'N-1) (P5N-1) (PN-I)
Q9D074	MGRN1_MOUSE	E3 ubiquitin-protein ligase MGRN1 (EC 2.3.2.27) (Mahogunin RING finger protein 1) (RING-type E3 ubiquitin transferase MGRN1)
Q9D0B6	PBDC1_MOUSE	Protein PBDC1 (Polysaccharide biosynthesis domain-containing protein 1)
Q9D0N7	CAF1B_MOUSE	Chromatin assembly factor 1 subunit B (CAF-1 subunit B) (Chromatin assembly factor I p60 subunit) (CAF-I 60 kDa subunit) (CAF-I p60)
Q9D0V8	CINP_MOUSE	Cyclin-dependent kinase 2-interacting protein
Q9D0Y8	RM52_MOUSE	39S ribosomal protein L52, mitochondrial (L52mt) (MRP-L52)
Q9D164	FXYD6_MOUSE	FXYD domain-containing ion transport regulator 6 (PLM-like protein) (Phosphohippolin)
Q9D1J3	SARNP_MOUSE	SAP domain-containing ribonucleoprotein (Nuclear protein Hcc-1)
Q9D2D7	ZN687_MOUSE	Zinc finger protein 687
Q9D2X5	SCC4_MOUSE	MAU2 chromatid cohesion factor homolog (MAU-2) (Cohesin loading complex subunit SCC4 homolog)
Q9D2Z8	KIF12_MOUSE	Kinesin-like protein KIF12
Q9D300	RGF1C_MOUSE	Ras-GEF domain-containing family member 1C

Q9D306	MGT4C_MOUSE	Alpha-1,3-mannosyl-glycoprotein 4-beta-N-acetylglucosaminyltransferase C (EC 2.4.1.145) (N-glycosyl-oligosaccharide-glycoprotein N-acetylglucosaminyltransferase IVc) (GnT-IVc) (N-acetylglucosaminyltransferase IVc) (UDP-N-acetylglucosamine: alpha-1,3-D-mannoside beta-1,4-N-acetylglucosaminyltransferase IVc)
Q9D342	T170A_MOUSE	Transmembrane protein 170A
Q9D3J8	AMTN_MOUSE	Amelotin
Q9D3U0	PUS10_MOUSE	Putative tRNA pseudouridine synthase Pus10 (EC 5.4.99.25) (Coiled-coil domain-containing protein 139) (tRNA pseudouridine 55 synthase) (Psi55 synthase) (tRNA pseudouridylate synthase) (tRNA-uridine isomerase)
Q9D4H1	EXOC2_MOUSE	Exocyst complex component 2 (Exocyst complex component Sec5)
Q9D5K4	S1PBP_MOUSE	S100P-binding protein
Q9D5R4	SPAT1_MOUSE	Spermatogenesis-associated protein 1
Q9D611	OCSTP_MOUSE	Osteoclast stimulatory transmembrane protein (OC-STAMP)
Q9D6J6	NDUV2_MOUSE	NADH dehydrogenase [ubiquinone] flavoprotein 2, mitochondrial (EC 1.6.99.3) (EC 7.1.1.2) (NADH-ubiquinone oxidoreductase 24 kDa subunit)
Q9D6Y7	MSRA_MOUSE	Mitochondrial peptide methionine sulfoxide reductase (EC 1.8.4.11) (Peptide-methionine (S)-S-oxide reductase) (Peptide Met(O) reductase) (Protein-methionine-S-oxide reductase) (PMSR)
Q9D7Z6	CLCA1_MOUSE	Calcium-activated chloride channel regulator 1 (EC 3.4.-.-) (Calcium-activated chloride channel family member 3) (mCLCA3) (Protein gob-5)
Q9D871	CEA18_MOUSE	Carcinoembryonic antigen-related cell adhesion molecule 18
Q9D8B4	NDUAB_MOUSE	NADH dehydrogenase [ubiquinone] 1 alpha subcomplex subunit 11 (Complex I-B14.7) (CI-B14.7) (NADH-ubiquinone oxidoreductase subunit B14.7)
Q9D8N2	DEN10_MOUSE	DENN domain-containing protein 10 (Protein FAM45A)
Q9D8S9	BOLA1_MOUSE	BolA-like protein 1
Q9D939	ST1C2_MOUSE	Sulfotransferase 1C2 (ST1C2) (EC 2.8.2.-)
Q9D967	MGDP1_MOUSE	Magnesium-dependent phosphatase 1 (MDP-1) (EC 3.1.3.-) (EC 3.1.3.48)
Q9D975	SRXN1_MOUSE	Sulfiredoxin-1 (EC 1.8.98.2) (Neoplastic progression protein 3)
Q9D9F8	MIPO1_MOUSE	Mirror-image polydactyly gene 1 protein homolog

Q9DAI6	F135B_MOUSE	Protein FAM135B
Q9DAL3	CCD54_MOUSE	Coiled-coil domain-containing protein 54
Q9DAW9	CNN3_MOUSE	Calponin-3 (Calponin, acidic isoform)
Q9DBB1	DUS6_MOUSE	Dual specificity protein phosphatase 6 (EC 3.1.3.16) (EC 3.1.3.48) (Mitogen-activated protein kinase phosphatase 3) (MAP kinase phosphatase 3) (MKP-3)
Q9DBC3	CMTR1_MOUSE	Cap-specific mRNA (nucleoside-2'-O-)-methyltransferase 1 (EC 2.1.1.57) (Cap methyltransferase 1) (Cap1 2'O-ribose methyltransferase 1) (MTr1) (FtsJ methyltransferase domain-containing protein 2)
Q9DBI2	BBS10_MOUSE	Bardet-Biedl syndrome 10 protein homolog
Q9DBL7	COASY_MOUSE	Bifunctional coenzyme A synthase (CoA synthase) [Includes: Phosphopantetheine adenylyltransferase (EC 2.7.7.3) (Dephospho-CoA pyrophosphorylase) (Pantetheine-phosphate adenylyltransferase) (PPAT); Dephospho-CoA kinase (DPCK) (EC 2.7.1.24) (Dephosphocoenzyme A kinase) (DPCOAK)]
Q9DBN4	P33MX_MOUSE	Putative monooxygenase p33MONOX (EC 1.-.-.-)
Q9DBR1	XRN2_MOUSE	5'-3' exoribonuclease 2 (EC 3.1.13.-) (Protein Dhm1)
Q9DC50	OCTC_MOUSE	Peroxisomal carnitine O-octanoyltransferase (COT) (EC 2.3.1.137)
Q9DCB8	ISCA2_MOUSE	Iron-sulfur cluster assembly 2 homolog, mitochondrial (HESB-like domain-containing protein 1)
Q9DCC4	P5CR3_MOUSE	Pyrroline-5-carboxylate reductase 3 (P5C reductase 3) (P5CR 3) (EC 1.5.1.2) (Pyrroline-5-carboxylate reductase-like protein)
Q9DCU2	PLLP_MOUSE	Plasmolipin (Plasma membrane proteolipid)
Q9DD16	NUD22_MOUSE	Uridine diphosphate glucose pyrophosphatase NUDT22 (UDPG pyrophosphatase) (UGPPase) (EC 3.6.1.45) (Nucleoside diphosphate-linked moiety X motif 22) (Nudix motif 22)
Q9EPJ9	ARFG1_MOUSE	ADP-ribosylation factor GTPase-activating protein 1 (ARF GAP 1) (ADP-ribosylation factor 1 GTPase-activating protein) (ARF1 GAP) (ARF1-directed GTPase-activating protein)
Q9EPL5	MMP1A_MOUSE	Interstitial collagenase A (EC 3.4.24.7) (Matrix metalloproteinase-1a) (MMP-1a) (Mcol-A)
Q9EPU5	TNR21_MOUSE	Tumor necrosis factor receptor superfamily member 21 (Death receptor 6) (CD antigen CD358)

Q9EPW0	INP4A_MOUSE	Inositol polyphosphate-4-phosphatase type I A (Inositol polyphosphate 4-phosphatase type I) (Inositol polyphosphate 4-phosphatase-1) (4-Ptase-1) (Type I inositol 3,4-bisphosphate 4-phosphatase) (EC 3.1.3.66)
Q9EPZ6	TBX18_MOUSE	T-box transcription factor TBX18 (T-box protein 18)
Q9EQ28	DPOD3_MOUSE	DNA polymerase delta subunit 3 (DNA polymerase delta subunit p66)
Q9EQC5	SCYL1_MOUSE	N-terminal kinase-like protein (105 kDa kinase-like protein) (Mitosis-associated kinase-like protein NTKL) (SCY1-like protein 1)
Q9EQF5	DPYS_MOUSE	Dihydropyrimidinase (DHP) (DHPase) (EC 3.5.2.2) (Dihydropyrimidine amidohydrolase) (Hydantoinase)
Q9EQZ7	RIMS2_MOUSE	Regulating synaptic membrane exocytosis protein 2 (Rab-3-interacting molecule 2) (RIM 2) (Rab-3-interacting protein 2)
Q9ER74	SALL1_MOUSE	Sal-like protein 1 (Zinc finger protein Spalt-3) (Sal-3) (mSal-3)
Q9ERG0	LIMA1_MOUSE	LIM domain and actin-binding protein 1 (Epithelial protein lost in neoplasm) (mEPLIN)
Q9ERK0	RIPK4_MOUSE	Receptor-interacting serine/threonine-protein kinase 4 (EC 2.7.11.1) (Ankyrin repeat domain-containing protein 3) (PKC-associated protein kinase) (PKC-regulated protein kinase)
Q9ERV7	PIDD1_MOUSE	p53-induced death domain-containing protein 1 (EC 3.4.21.-) (Leucine-rich repeat and death domain-containing protein) [Cleaved into: PIDD-N; PIDD-C; PIDD-CC]
Q9ES07	S15A2_MOUSE	Solute carrier family 15 member 2 (Kidney H(+)/peptide cotransporter) (Oligopeptide transporter, kidney isoform) (Peptide transporter 2)
Q9ES64	USH1C_MOUSE	Harmonin (PDZ domain-containing protein) (Usher syndrome type-1C protein homolog)
Q9ES88	S13A2_MOUSE	Solute carrier family 13 member 2 (Na(+)/dicarboxylate cotransporter 1) (NaDC-1) (Renal sodium/dicarboxylate cotransporter)
Q9ESC8	AFF4_MOUSE	AF4/FMR2 family member 4
Q9ESF1	OTOF_MOUSE	Otoferlin (Fer-1-like protein 2)
Q9ESG8	ZDH16_MOUSE	Palmitoyltransferase ZDHHC16 (EC 2.3.1.225) (Abl-philin 2) (Zinc finger DHHC domain-containing protein 16) (DHHC-16)
Q9ESJ7	IKBP1_MOUSE	Interleukin-1 receptor-associated kinase 1-binding protein 1 (IRAK1-binding protein 1) (ActA-binding protein 70) (PLK-interacting protein) (Signaling

		molecule that associates with the mouse pelle-like kinase) (SIMPL)
Q9ESM6	GDPD2_MOUSE	Glycerophosphoinositol inositolphosphodiesterase GDPD2 (EC 3.1.4.43) (Glycerophosphodiester phosphodiesterase 3) (Glycerophosphodiester phosphodiesterase domain-containing protein 2) (Osteoblast differentiation promoting factor)
Q9ESN4	C1QL3_MOUSE	Complement C1q-like protein 3 (C1q and tumor necrosis factor-related protein 13) (C1q/TNF-related protein 13) (CTRP13) (Gliacolin)
Q9ET26	RN114_MOUSE	E3 ubiquitin-protein ligase RNF114 (EC 2.3.2.27) (RING finger protein 114) (RING-type E3 ubiquitin transferase RNF114) (Zinc finger protein 228) (Zinc finger protein 313)
Q9ET37	S5A4A_MOUSE	Solute carrier family 5 member 4A
Q9ET66	PI16_MOUSE	Peptidase inhibitor 16 (PI-16) (Cysteine-rich protease inhibitor) (CD antigen CD364)
Q9JHA8	VWA7_MOUSE	von Willebrand factor A domain-containing protein 7 (Protein G7c)
Q9JHC9	ELF2_MOUSE	ETS-related transcription factor Elf-2 (E74-like factor 2) (New ETS-related factor)
Q9JHG7	PK3CG_MOUSE	Phosphatidylinositol 4,5-bisphosphate 3-kinase catalytic subunit gamma isoform (PI3-kinase subunit gamma) (PI3K-gamma) (PI3Kgamma) (PtdIns-3-kinase subunit gamma) (EC 2.7.1.153) (Phosphatidylinositol 4,5-bisphosphate 3-kinase 110 kDa catalytic subunit gamma) (PtdIns-3-kinase subunit p110-gamma) (p110gamma) (Phosphoinositide-3-kinase catalytic gamma polypeptide) (Serine/threonine protein kinase PIK3CG) (EC 2.7.11.1) (p120-PI3K)
Q9JHI8	NOX4_MOUSE	NADPH oxidase 4 (EC 1.6.3.-) (Kidney oxidase-1) (KOX-1) (Kidney superoxide-producing NADPH oxidase) (Renal NAD(P)H-oxidase) (Superoxide-generating NADPH oxidase 4)
Q9JHS3	LTOR2_MOUSE	Ragulator complex protein LAMTOR2 (Endosomal adaptor protein p14) (Late endosomal/lysosomal Mp1-interacting protein) (Late endosomal/lysosomal adaptor and MAPK and MTOR activator 2) (Mitogen-activated protein-binding protein-interacting protein) (Roadblock domain-containing protein 3)
Q9JIF3	GTR8_MOUSE	Solute carrier family 2, facilitated glucose transporter member 8 (Glucose transporter type 8) (GLUT-8) (Glucose transporter type X1)

Q9JIM3	ER6L2_MOUSE	DNA excision repair protein ERCC-6-like 2 (EC 3.6.4.-) (DNA repair and recombination protein RAD26-like)
Q9JIP3	I17RB_MOUSE	Interleukin-17 receptor B (IL-17 receptor B) (IL-17RB) (IL-17 receptor homolog 1) (IL-17ER) (IL-17Rh1) (IL17Rh1) (Interleukin-17B receptor) (IL-17B receptor)
Q9JIQ3	DBLOH_MOUSE	Diablo homolog, mitochondrial (Direct IAP-binding protein with low pI) (Second mitochondria-derived activator of caspase) (Smac)
Q9JIW4	DPOLM_MOUSE	DNA-directed DNA/RNA polymerase mu (Pol Mu) (EC 2.7.7.7) (Terminal transferase)
Q9JJB9	TM183_MOUSE	Transmembrane protein 183
Q9JJC6	RIPL1_MOUSE	RILP-like protein 1 (Rab-interacting lysosomal-like protein 1)
Q9JJE4	PAQR4_MOUSE	Progestin and adipoQ receptor family member 4 (Progestin and adipoQ receptor family member IV)
Q9JJN2	ZFHX4_MOUSE	Zinc finger homeobox protein 4 (Zinc finger homeodomain protein 4) (ZFH-4)
Q9JJX7	TYDP2_MOUSE	Tyrosyl-DNA phosphodiesterase 2 (Tyr-DNA phosphodiesterase 2) (EC 3.1.4.-) (5'-tyrosyl-DNA phosphodiesterase) (5'-Tyr-DNA phosphodiesterase) (TRAF and TNF receptor-associated protein)
Q9JJZ9	CNGB3_MOUSE	Cyclic nucleotide-gated cation channel beta-3 (Cone photoreceptor cGMP-gated channel subunit beta) (Cyclic nucleotide-gated cation channel modulatory subunit) (Cyclic nucleotide-gated channel beta-3) (CNG channel beta-3) (Cyclic nucleotide-gated channel subunit CNG6)
Q9JKL5	CHP3_MOUSE	Calcineurin B homologous protein 3 (Tescalcin) (TE-1) (TSC)
Q9JKN6	NOVA1_MOUSE	RNA-binding protein Nova-1 (Neuro-oncological ventral antigen 1) (Ventral neuron-specific protein 1)
Q9JKS4	LDB3_MOUSE	LIM domain-binding protein 3 (Protein cypher) (Protein oracle) (Z-band alternatively spliced PDZ-motif protein)
Q9JL16	ISG20_MOUSE	Interferon-stimulated gene 20 kDa protein (EC 3.1.13.1) (Promyelocytic leukemia nuclear body-associated protein ISG20) (Protein DnaQL)
Q9JL21	CCR10_MOUSE	C-C chemokine receptor type 10 (C-C CKR-10) (CC-CKR-10) (CCR-10) (Chemokine C-C receptor 9) (G-protein coupled receptor 2)
Q9JL95	PRG3_MOUSE	Proteoglycan 3 (Eosinophil major basic protein 2)
Q9JLB4	CUBN_MOUSE	Cubilin (Intrinsic factor-cobalamin receptor)

Q9JLG8	CAN15_MOUSE	Calpain-15 (EC 3.4.22.-) (Small optic lobes homolog)
Q9JLI7	SPAG6_MOUSE	Sperm-associated antigen 6 (Axoneme central apparatus protein) (Protein PF16 homolog)
Q9JLN9	MTOR_MOUSE	Serine/threonine-protein kinase mTOR (EC 2.7.11.1) (FK506-binding protein 12-rapamycin complex-associated protein 1) (FKBP12-rapamycin complex-associated protein) (Mammalian target of rapamycin) (mTOR) (Mechanistic target of rapamycin) (Rapamycin target protein 1) (RAPT1)
Q9JLV1	BAG3_MOUSE	BAG family molecular chaperone regulator 3 (BAG-3) (Bcl-2-associated athanogene 3) (Bcl-2-binding protein Bis)
Q9JM13	RABX5_MOUSE	Rab5 GDP/GTP exchange factor (Rabex-5)
Q9JM55	TOB2_MOUSE	Protein Tob2 (Transducer of erbB-2 2)
Q9JM58	CRLF1_MOUSE	Cytokine receptor-like factor 1 (Cytokine receptor-like molecule 3) (CRLM-3) (Cytokine-like factor 1) (CLF-1) (Novel cytokine receptor 6) (NR6)
Q9JMA9	S6A14_MOUSE	Sodium- and chloride-dependent neutral and basic amino acid transporter B(0+) (Amino acid transporter ATB0+) (Colonic system B0+ amino acid transporter CATB0+) (Solute carrier family 6 member 14)
Q9JMB7	PIWL1_MOUSE	Piwi-like protein 1 (EC 3.1.26.-)
Q9JMD0	ZN207_MOUSE	BUB3-interacting and GLEBS motif-containing protein ZNF207 (BuGZ) (49 kDa zinc finger protein) (Zinc finger protein 207)
Q9QUG9	GRP2_MOUSE	RAS guanyl-releasing protein 2 (Calcium and DAG-regulated guanine nucleotide exchange factor I) (CalDAG-GEFI) (F25B3.3 kinase-like protein)
Q9QUH0	GLRX1_MOUSE	Glutaredoxin-1 (Thioltransferase-1) (TTase-1)
Q9QUM7	MSH5_MOUSE	MutS protein homolog 5
Q9QUP4	CHST5_MOUSE	Carbohydrate sulfotransferase 5 (EC 2.8.2.-) (Galactose/N-acetylglucosamine/N-acetylglucosamine 6-O-sulfotransferase 4) (GST4) (Intestinal N-acetylglucosamine-6-O-sulfotransferase) (I-GlcNAc6ST) (Intestinal GlcNAc-6-sulfotransferase) (mIGn6ST) (N-acetylglucosamine 6-O-sulfotransferase 3) (GlcNAc6ST-3) (Gn6st-3)
Q9QUR7	PIN1_MOUSE	Peptidyl-prolyl cis-trans isomerase NIMA-interacting 1 (EC 5.2.1.8) (Peptidyl-prolyl cis-trans isomerase Pin1) (PPIase Pin1)
Q9QWT9	KIFC1_MOUSE	Kinesin-like protein KIFC1

Q9QXD1	ACOX2_MOUSE	Peroxisomal acyl-coenzyme A oxidase 2 (EC 1.17.99.3) (3-alpha,7-alpha,12-alpha-trihydroxy-5-beta-cholestanoyl-CoA 24-hydroxylase) (3-alpha,7-alpha,12-alpha-trihydroxy-5-beta-cholestanoyl-CoA oxidase) (Trihydroxycoprostanoyl-CoA oxidase) (THCA-CoA oxidase) (THCCox)
Q9QXG4	ACSA_MOUSE	Acetyl-coenzyme A synthetase, cytoplasmic (EC 6.2.1.1) (Acetate--CoA ligase) (Acetyl-CoA synthetase) (ACS) (AceCS) (Acetyl-CoA synthetase 1) (AceCS1) (Acyl-CoA synthetase short-chain family member 2) (Acyl-activating enzyme) (Propionate--CoA ligase) (EC 6.2.1.17)
Q9QXK7	CPSF3_MOUSE	Cleavage and polyadenylation specificity factor subunit 3 (EC 3.1.27.-) (Cleavage and polyadenylation specificity factor 73 kDa subunit) (CPSF 73 kDa subunit) (mRNA 3'-end-processing endonuclease CPSF-73)
Q9QXT6	IL17B_MOUSE	Interleukin-17B (IL-17B) (Cytokine CX1) (Cytokine-like protein ZCYTO7) (Neuronal interleukin-17-related factor)
Q9QY24	ZBP1_MOUSE	Z-DNA-binding protein 1 (DNA-dependent activator of IFN-regulatory factors) (DAI) (Tumor stroma and activated macrophage protein DLM-1)
Q9QY81	PO210_MOUSE	Nuclear pore membrane glycoprotein 210 (Nuclear pore protein gp210) (Nuclear envelope pore membrane protein POM 210) (POM210) (Nucleoporin Nup210) (Pore membrane protein of 210 kDa)
Q9QYB8	ADDB_MOUSE	Beta-adducin (Add97) (Erythrocyte adducin subunit beta)
Q9QYJ3	DNJB1_MOUSE	DnaJ homolog subfamily B member 1 (Heat shock 40 kDa protein 1) (HSP40) (Heat shock protein 40)
Q9QYK9	KCC1B_MOUSE	Calcium/calmodulin-dependent protein kinase type 1B (EC 2.7.11.17) (CaM kinase I beta) (CaM kinase IB) (CaM-KI beta) (CaMKI-beta) (Pregnancy up-regulated non-ubiquitously-expressed CaM kinase homolog)
Q9QZ06	TOLIP_MOUSE	Toll-interacting protein
Q9QZ85	IIGP1_MOUSE	Interferon-inducible GTPase 1 (EC 3.6.5.-)
Q9QZB9	DCTN5_MOUSE	Dynactin subunit 5 (Dynactin subunit p25)
Q9QZE7	TSNAX_MOUSE	Translin-associated protein X (Translin-associated factor X)
Q9QZL6	UBP21_MOUSE	Ubiquitin carboxyl-terminal hydrolase 21 (EC 3.4.19.12) (Deubiquitinating enzyme 21) (Ubiquitin thioesterase 21) (Ubiquitin-specific-processing protease 21)

Q9QZM0	UBQL2_MOUSE	Ubiquilin-2 (Chap1) (DSK2 homolog) (Protein linking IAP with cytoskeleton 2) (PLIC-2) (Ubiquitin-like product Chap1/Dsk2)
Q9QZU9	UB2L6_MOUSE	Ubiquitin/ISG15-conjugating enzyme E2 L6 (EC 2.3.2.23) (E2 ubiquitin-conjugating enzyme L6) (UbcM8) (Ubiquitin carrier protein L6) (Ubiquitin-protein ligase L6)
Q9QZZ4	MYO15_MOUSE	Unconventional myosin-XV (Unconventional myosin-15)
Q9R053	SCNBA_MOUSE	Sodium channel protein type 11 subunit alpha (NaN) (Sensory neuron sodium channel 2) (Sodium channel protein type XI subunit alpha) (Voltage-gated sodium channel subunit alpha Nav1.9)
Q9R069	BCAM_MOUSE	Basal cell adhesion molecule (B-CAM cell surface glycoprotein) (Lutheran antigen) (CD antigen CD239)
Q9R078	AAKB1_MOUSE	5'-AMP-activated protein kinase subunit beta-1 (AMPK subunit beta-1) (AMPKb)
Q9R0P9	UCHL1_MOUSE	Ubiquitin carboxyl-terminal hydrolase isozyme L1 (UCH-L1) (EC 3.4.19.12) (Neuron cytoplasmic protein 9.5) (PGP 9.5) (PGP9.5) (Ubiquitin thioesterase L1)
Q9R0X5	RPGR_MOUSE	X-linked retinitis pigmentosa GTPase regulator (mRpgr)
Q9R117	TYK2_MOUSE	Non-receptor tyrosine-protein kinase TYK2 (EC 2.7.10.2)
Q9R1S3	PIGN_MOUSE	GPI ethanolamine phosphate transferase 1 (EC 2.-.-.-) (Phosphatidylinositol-glycan biosynthesis class N protein) (PIG-N)
Q9WTJ8	FA50B_MOUSE	Protein FAM50B (Protein XAP-5-like)
Q9WTL2	RAB25_MOUSE	Ras-related protein Rab-25
Q9WTR6	XCT_MOUSE	Cystine/glutamate transporter (Amino acid transport system xc-) (Solute carrier family 7 member 11) (xCT)
Q9WTS4	TEN1_MOUSE	Teneurin-1 (Ten-1) (Protein Odd Oz/ten-m homolog 1) (Tenascin-M1) (Ten-m1) (Teneurin transmembrane protein 1) [Cleaved into: Ten-1 intracellular domain (IDten-1) (Ten-1 ICD); Teneurin C-terminal-associated peptide (TCPA-1) (Ten-1 extracellular domain) (Ten-1 ECD)]
Q9WTS6	TEN3_MOUSE	Teneurin-3 (Ten-3) (Protein Odd Oz/ten-m homolog 3) (Tenascin-M3) (Ten-m3) (Teneurin transmembrane protein 3)
Q9WTX5	SKP1_MOUSE	S-phase kinase-associated protein 1 (Cyclin-A/CDK2-associated protein p19) (S-phase kinase-associated protein 1A) (p19A) (p19skp1)

Q9WU60	ATRN_MOUSE	Attractin (Protein mahogany)
Q9WU81	G6PT3_MOUSE	Glucose-6-phosphate exchanger SLC37A2 (Solute carrier family 37 member 2) (cAMP-inducible protein 2)
Q9WUF3	C8AP2_MOUSE	CASP8-associated protein 2 (FLICE-associated huge protein)
Q9WUK4	RFC2_MOUSE	Replication factor C subunit 2 (Activator 1 40 kDa subunit) (A1 40 kDa subunit) (Activator 1 subunit 2) (Replication factor C 40 kDa subunit) (RF-C 40 kDa subunit) (RFC40)
Q9WUT7	CCR9_MOUSE	C-C chemokine receptor type 9 (C-C CKR-9) (CC-CKR-9) (CCR-9) (Chemokine C-C receptor 10) (CD antigen CDw199)
Q9WV04	KIF9_MOUSE	Kinesin-like protein KIF9
Q9WV18	GABR1_MOUSE	Gamma-aminobutyric acid type B receptor subunit 1 (GABA-B receptor 1) (GABA-B-R1) (GABA-BR1) (GABABR1) (Gb1)
Q9WVF7	DPOE1_MOUSE	DNA polymerase epsilon catalytic subunit A (EC 2.7.7.7) (3'-5' exodeoxyribonuclease) (EC 3.1.11.-) (DNA polymerase II subunit A)
Q9WVG6	CARM1_MOUSE	Histone-arginine methyltransferase CARM1 (EC 2.1.1.319) (Coactivator-associated arginine methyltransferase 1) (Protein arginine N-methyltransferase 4)
Q9Z0E3	AIRE_MOUSE	Autoimmune regulator (Autoimmune polyendocrinopathy candidiasis ectodermal dystrophy protein homolog) (APECED protein homolog)
Q9Z0H8	CLIP2_MOUSE	CAP-Gly domain-containing linker protein 2 (Cytoplasmic linker protein 115) (CLIP-115) (Cytoplasmic linker protein 2)
Q9Z0M3	CAD20_MOUSE	Cadherin-20 (Cadherin-7)
Q9Z0P4	PALM_MOUSE	Paralemmin-1 (Paralemmin)
Q9Z0R6	ITSN2_MOUSE	Intersectin-2 (EH domain and SH3 domain regulator of endocytosis 2) (EH and SH3 domains protein 2) (SH3 domain-containing protein 1B)
Q9Z0W1	TNR16_MOUSE	Tumor necrosis factor receptor superfamily member 16 (Low affinity neurotrophin receptor p75NTR) (Low-affinity nerve growth factor receptor) (NGF receptor) (CD antigen CD271)
Q9Z0Y1	DCTN3_MOUSE	Dynactin subunit 3 (Dynactin light chain p24)
Q9Z139	ROR1_MOUSE	Inactive tyrosine-protein kinase transmembrane receptor ROR1 (mROR1) (Neurotrophic tyrosine kinase, receptor-related 1)

Q9Z148	EHMT2_MOUSE	Histone-lysine N-methyltransferase EHMT2 (EC 2.1.1.-) (Euchromatic histone-lysine N-methyltransferase 2) (HLA-B-associated transcript 8) (Histone H3-K9 methyltransferase 3) (H3-K9-HMTase 3) (Protein G9a)
Q9Z1B3	PLCB1_MOUSE	1-phosphatidylinositol 4,5-bisphosphate phosphodiesterase beta-1 (EC 3.1.4.11) (PLC-154) (Phosphoinositide phospholipase C-beta-1) (Phospholipase C-beta-1) (PLC-beta-1)
Q9Z1B5	MD2L1_MOUSE	Mitotic spindle assembly checkpoint protein MAD2A (Mitotic arrest deficient 2-like protein 1) (MAD2-like protein 1)
Q9Z1D9	ZN394_MOUSE	Zinc finger protein 394 (Zinc finger protein 94) (Zfp-94) (Zinc finger protein with KRAB and SCAN domains 14)
Q9Z1K6	ARI2_MOUSE	E3 ubiquitin-protein ligase ARIH2 (ARI-2) (Protein ariadne-2 homolog) (EC 2.3.2.31) (RING-type E3 ubiquitin transferase ARIH2) (Triad1 protein) (UbcM4-interacting protein 48)
Q9Z1L5	CA2D3_MOUSE	Voltage-dependent calcium channel subunit alpha-2/delta-3 (Voltage-gated calcium channel subunit alpha-2/delta-3) [Cleaved into: Voltage-dependent calcium channel subunit alpha-2-3; Voltage-dependent calcium channel subunit delta-3]
Q9Z1N2	ORC1_MOUSE	Origin recognition complex subunit 1
Q9Z1W9	STK39_MOUSE	STE20/SPS1-related proline-alanine-rich protein kinase (Ste-20-related kinase) (EC 2.7.11.1) (Serine/threonine-protein kinase 39)
Q9Z2B5	E2AK3_MOUSE	Eukaryotic translation initiation factor 2-alpha kinase 3 (EC 2.7.11.1) (PRKR-like endoplasmic reticulum kinase) (Pancreatic eIF2-alpha kinase)
Q9Z2C5	MTM1_MOUSE	Myotubularin (Phosphatidylinositol-3,5-bisphosphate 3-phosphatase) (EC 3.1.3.95) (Phosphatidylinositol-3-phosphate phosphatase) (EC 3.1.3.64)

Table 4. Non differentially expressed proteins.

Accession	Entry name	Protein names
A1L314	MPEG1_MOUSE	Macrophage-expressed gene 1 protein (Macrophage gene 1 protein) (Mpg-1) (Perforin-2) (P-2) (Protein MPS1)
A2A690	TANC2_MOUSE	Protein TANC2 (Tetratricopeptide repeat, ankyrin repeat and coiled-coil domain-containing protein 2)
A2A935	PRD16_MOUSE	Histone-lysine N-methyltransferase PRDM16 (EC 2.1.1.-) (PR domain zinc finger protein 16) (PR domain-containing protein 16) (Transcription factor MEL1) (MDS1/EVI1-like gene 1)
A2ABU4	MYOM3_MOUSE	Myomesin-3 (Myomesin family member 3)
A2AGT5	CKAP5_MOUSE	Cytoskeleton-associated protein 5
A2AN08	UBR4_MOUSE	E3 ubiquitin-protein ligase UBR4 (EC 2.3.2.27) (N-recognin-4) (RING-type E3 ubiquitin transferase UBR4) (Zinc finger UBR1-type protein 1) (p600)
A2APV2	FMNL2_MOUSE	Formin-like protein 2 (Protein Man)
A2AQ07	TBB1_MOUSE	Tubulin beta-1 chain
A2ASS6	TITIN_MOUSE	Titin (EC 2.7.11.1) (Connectin)
A4Q9E8	TTLL6_MOUSE	Tubulin polyglutamylase TTLL6 (EC 6.-.-.-) (Tubulin--tyrosine ligase-like protein 6)
A6H6A9	RBG1L_MOUSE	Rab GTPase-activating protein 1-like
B2RQC6	PYR1_MOUSE	CAD protein [Includes: Glutamine-dependent carbamoyl-phosphate synthase (EC 6.3.5.5); Aspartate carbamoyltransferase (EC 2.1.3.2); Dihydroorotase (EC 3.5.2.3)]
B2RXC1	TPC11_MOUSE	Trafficking protein particle complex subunit 11
B2RXV4	FLVC1_MOUSE	Feline leukemia virus subgroup C receptor-related protein 1 (Feline leukemia virus subgroup C receptor) (Major facilitator superfamily domain containing 7B) (Mfsd7b)
D3Z6Q9	BIN2_MOUSE	Bridging integrator 2
E9Q414	APOB_MOUSE	Apolipoprotein B-100 (Apo B-100) [Cleaved into: Apolipoprotein B-48 (Apo B-48)]
E9Q555	RN213_MOUSE	E3 ubiquitin-protein ligase RNF213 (EC 2.3.2.27) (EC 3.6.4.-) (Mysterin) (RING finger protein 213) (RING-type E3 ubiquitin transferase RNF213)
E9Q634	MYO1E_MOUSE	Unconventional myosin-Ie (Unconventional myosin 1E)
G5E829	AT2B1_MOUSE	Plasma membrane calcium-transporting ATPase 1 (EC 7.2.2.10) (Plasma membrane calcium ATPase isoform 1) (PMCA1) (Plasma membrane calcium pump isoform 1)
O08529	CAN2_MOUSE	Calpain-2 catalytic subunit (EC 3.4.22.53) (80 kDa M-calpain subunit) (CALP80) (Calcium-activated neutral proteinase 2) (CANP 2) (Calpain M-type) (Calpain-2 large subunit) (Millimolar-calpain) (M-calpain)
O08553	DPYL2_MOUSE	Dihydropyrimidinase-related protein 2 (DRP-2) (Unc-33-like phosphoprotein 2) (ULIP-2)

O08692	NGP_MOUSE	Neutrophilic granule protein (NGP) (Cystatin-like protein) (Myeloid bactenecin protein) (Myeloid secondary granule protein)
O08738	CASP6_MOUSE	Caspase-6 (CASP-6) (EC 3.4.22.59) (Apoptotic protease Mch-2) [Cleaved into: Caspase-6 subunit p18; Caspase-6 subunit p11]
O08788	DCTN1_MOUSE	Dynactin subunit 1 (150 kDa dynein-associated polypeptide) (DAP-150) (DP-150) (p150-glued)
O08808	DIAP1_MOUSE	Protein diaphanous homolog 1 (Diaphanous-related formin-1) (DRF1) (p140mDIA) (mDIA1)
O08810	U5S1_MOUSE	116 kDa U5 small nuclear ribonucleoprotein component (Elongation factor Tu GTP-binding domain-containing protein 2) (U5 snRNP-specific protein, 116 kDa) (U5-116 kDa)
O08917	FLOT1_MOUSE	Flotillin-1
O08966	S22A1_MOUSE	Solute carrier family 22 member 1 (Organic cation transporter 1) (mOCT1)
O09044	SNP23_MOUSE	Synaptosomal-associated protein 23 (SNAP-23) (Syndet) (Vesicle-membrane fusion protein SNAP-23)
O09061	PSB1_MOUSE	Proteasome subunit beta type-1 (EC 3.4.25.1) (Macropain subunit C5) (Multicatalytic endopeptidase complex subunit C5) (Proteasome component C5) (Proteasome gamma chain)
O09106	HDAC1_MOUSE	Histone deacetylase 1 (HD1) (EC 3.5.1.98)
O09131	GSTO1_MOUSE	Glutathione S-transferase omega-1 (GSTO-1) (EC 2.5.1.18) (Glutathione S-transferase omega 1-1) (GSTO 1-1) (Glutathione-dependent dehydroascorbate reductase) (EC 1.8.5.1) (Monomethylarsonic acid reductase) (MMA(V) reductase) (EC 1.20.4.2) (S-(Phenacyl)glutathione reductase) (SPG-R) (p28)
O09159	MA2B1_MOUSE	Lysosomal alpha-mannosidase (Laman) (EC 3.2.1.24) (Lysosomal acid alpha-mannosidase) (Mannosidase alpha class 2B member 1) (Mannosidase alpha-B)
O35075	VP26C_MOUSE	Vacuolar protein sorting-associated protein 26C (Down syndrome critical region protein 3 homolog) (Down syndrome critical region protein A homolog)
O35226	PSMD4_MOUSE	26S proteasome non-ATPase regulatory subunit 4 (26S proteasome regulatory subunit RPN10) (26S proteasome regulatory subunit S5A) (Multiubiquitin chain-binding protein)
O35286	DHX15_MOUSE	Pre-mRNA-splicing factor ATP-dependent RNA helicase DHX15 (EC 3.6.4.13) (DEAH box protein 15)
O35295	PURB_MOUSE	Transcriptional activator protein Pur-beta (Purine-rich element-binding protein B) (Vascular actin single-stranded DNA-binding factor 2 p44 component)
O35309	NMI_MOUSE	N-myc-interactor (Nmi) (N-myc and STAT interactor)
O35343	IMA3_MOUSE	Importin subunit alpha-3 (Importin alpha Q1) (Qip1) (Karyopherin subunit alpha-4)

O35350	CAN1_MOUSE	Calpain-1 catalytic subunit (EC 3.4.22.52) (Calcium-activated neutral proteinase 1) (CANP 1) (Calpain mu-type) (Calpain-1 large subunit) (Micromolar-calpain) (muCANP)
O35375	NRP2_MOUSE	Neuropilin-2 (Vascular endothelial cell growth factor 165 receptor 2)
O35379	MRP1_MOUSE	Multidrug resistance-associated protein 1 (EC 7.6.2.2) (ATP-binding cassette sub-family C member 1) (Glutathione-S-conjugate-translocating ATPase ABCC1) (EC 7.6.2.3) (Leukotriene C(4) transporter) (LTC4 transporter)
O35381	AN32A_MOUSE	Acidic leucine-rich nuclear phosphoprotein 32 family member A (Acidic nuclear phosphoprotein pp32) (Leucine-rich acidic nuclear protein) (LANP) (Potent heat-stable protein phosphatase 2A inhibitor I1PP2A)
O35593	PSDE_MOUSE	26S proteasome non-ATPase regulatory subunit 14 (EC 3.4.19.-) (26S proteasome regulatory subunit RPN11) (MAD1)
O35625	AXIN1_MOUSE	Axin-1 (Axis inhibition protein 1) (Protein Fused)
O35643	AP1B1_MOUSE	AP-1 complex subunit beta-1 (Adaptor protein complex AP-1 subunit beta-1) (Adaptor-related protein complex 1 subunit beta-1) (Beta-1-adaptin) (Beta-adaptin 1) (Clathrin assembly protein complex 1 beta large chain) (Golgi adaptor HA1/AP1 adaptin beta subunit)
O35678	MGLL_MOUSE	Monoglyceride lipase (MGL) (EC 3.1.1.23) (Monoacylglycerol lipase) (MAGL)
O35682	MYADM_MOUSE	Myeloid-associated differentiation marker (Myeloid up-regulated protein)
O35737	HNRH1_MOUSE	Heterogeneous nuclear ribonucleoprotein H (hnRNP H) [Cleaved into: Heterogeneous nuclear ribonucleoprotein H, N-terminally processed]
O35855	BCAT2_MOUSE	Branched-chain-amino-acid aminotransferase, mitochondrial (BCAT(m)) (EC 2.6.1.42)
O35874	SATT_MOUSE	Neutral amino acid transporter A (Alanine/serine/cysteine/threonine transporter 1) (ASCT-1) (SATT) (Solute carrier family 1 member 4)
O35904	PK3CD_MOUSE	Phosphatidylinositol 4,5-bisphosphate 3-kinase catalytic subunit delta isoform (PI3-kinase subunit delta) (PI3K-delta) (PI3Kdelta) (PtdIns-3-kinase subunit delta) (EC 2.7.1.153) (Phosphatidylinositol 4,5-bisphosphate 3-kinase 110 kDa catalytic subunit delta) (PtdIns-3-kinase subunit p110-delta) (p110delta)
O54750	CP2J6_MOUSE	Cytochrome P450 2J6 (EC 1.14.14.1) (Arachidonic acid epoxygenase) (CYPIIJ6)
O54774	AP3D1_MOUSE	AP-3 complex subunit delta-1 (AP-3 complex subunit delta) (Adaptor-related protein complex 3 subunit delta-1) (Delta-adaptin) (mBLVR1)
O54890	ITB3_MOUSE	Integrin beta-3 (Platelet membrane glycoprotein IIIa) (GPIIIa) (CD antigen CD61)

O54941	SMCE1_MOUSE	SWI/SNF-related matrix-associated actin-dependent regulator of chromatin subfamily E member 1 (BRG1-associated factor 57) (BAF57)
O54950	AAKG1_MOUSE	5'-AMP-activated protein kinase subunit gamma-1 (AMPK gamma1) (AMPK subunit gamma-1) (AMPKg)
O54962	BAF_MOUSE	Barrier-to-autointegration factor (Breakpoint cluster region protein 1) (LAP2-binding protein 1) [Cleaved into: Barrier-to-autointegration factor, N-terminally processed]
O55029	COPB2_MOUSE	Coatomer subunit beta' (Beta'-coat protein) (Beta'-COP) (p102)
O55098	STK10_MOUSE	Serine/threonine-protein kinase 10 (EC 2.7.11.1) (Lymphocyte-oriented kinase)
O55131	SEPT7_MOUSE	Septin-7 (CDC10 protein homolog)
O55135	IF6_MOUSE	Eukaryotic translation initiation factor 6 (eIF-6) (B4 integrin interactor) (CAB) (p27(BBP))
O55143	AT2A2_MOUSE	Sarcoplasmic/endoplasmic reticulum calcium ATPase 2 (SERCA2) (SR Ca(2+)-ATPase 2) (EC 7.2.2.10) (Calcium pump 2) (Calcium-transporting ATPase sarcoplasmic reticulum type, slow twitch skeletal muscle isoform) (Endoplasmic reticulum class 1/2 Ca(2+) ATPase)
O70172	PI42A_MOUSE	Phosphatidylinositol 5-phosphate 4-kinase type-2 alpha (EC 2.7.1.149) (1-phosphatidylinositol 5-phosphate 4-kinase 2-alpha) (Diphosphoinositide kinase 2-alpha) (Phosphatidylinositol 5-phosphate 4-kinase type II alpha) (PI(5)P 4-kinase type II alpha) (PIP4KII-alpha) (PtdIns(5)P-4-kinase isoform 2-alpha)
O70251	EF1B_MOUSE	Elongation factor 1-beta (EF-1-beta)
O70310	NMT1_MOUSE	Glycylpeptide N-tetradecanoyltransferase 1 (EC 2.3.1.97) (Myristoyl-CoA:protein N-myristoyltransferase 1) (NMT 1) (Type I N-myristoyltransferase) (Peptide N-myristoyltransferase 1)
O70311	NMT2_MOUSE	Glycylpeptide N-tetradecanoyltransferase 2 (EC 2.3.1.97) (Myristoyl-CoA:protein N-myristoyltransferase 2) (NMT 2) (Peptide N-myristoyltransferase 2) (Type II N-myristoyltransferase)
O70325	GPX4_MOUSE	Phospholipid hydroperoxide glutathione peroxidase (PHGPx) (EC 1.11.1.12) (Glutathione peroxidase 4) (GPx-4) (GSHPx-4)
O70435	PSA3_MOUSE	Proteasome subunit alpha type-3 (EC 3.4.25.1) (Macropain subunit C8) (Multicatalytic endopeptidase complex subunit C8) (Proteasome component C8) (Proteasome subunit K)
O70439	STX7_MOUSE	Syntaxin-7
O70468	MYPC3_MOUSE	Myosin-binding protein C, cardiac-type (Cardiac MyBP-C) (C-protein, cardiac muscle isoform)
O70591	PFD2_MOUSE	Prefoldin subunit 2
O88342	WDR1_MOUSE	WD repeat-containing protein 1 (Actin-interacting protein 1) (AIP1)
O88427	CAC1H_MOUSE	Voltage-dependent T-type calcium channel subunit alpha-1H (Voltage-gated calcium channel subunit alpha Cav3.2)

O88487	DC1I2_MOUSE	Cytoplasmic dynein 1 intermediate chain 2 (Cytoplasmic dynein intermediate chain 2) (Dynein intermediate chain 2, cytosolic) (DH IC-2)
O88544	CSN4_MOUSE	COP9 signalosome complex subunit 4 (SGN4) (Signalosome subunit 4) (JAB1-containing signalosome subunit 4)
O88569	ROA2_MOUSE	Heterogeneous nuclear ribonucleoproteins A2/B1 (hnRNP A2/B1)
O88587	COMT_MOUSE	Catechol O-methyltransferase (EC 2.1.1.6)
O88685	PRS6A_MOUSE	26S proteasome regulatory subunit 6A (26S proteasome AAA-ATPase subunit RPT5) (Proteasome 26S subunit ATPase 3) (Tat-binding protein 1) (TBP-1)
O88738	BIRC6_MOUSE	Baculoviral IAP repeat-containing protein 6 (EC 2.3.2.27) (BIR repeat-containing ubiquitin-conjugating enzyme) (BRUCE) (RING-type E3 ubiquitin transferase BIRC6) (Ubiquitin-conjugating BIR domain enzyme apollon) (APOLLON)
O88746	TOM1_MOUSE	Target of Myb protein 1
O88783	FA5_MOUSE	Coagulation factor V (Activated protein C cofactor) [Cleaved into: Coagulation factor V heavy chain; Coagulation factor V light chain]
O88844	IDHC_MOUSE	Isocitrate dehydrogenase [NADP] cytoplasmic (IDH) (EC 1.1.1.42) (Cytosolic NADP-isocitrate dehydrogenase) (IDP) (NADP(+)-specific ICDH) (Oxalosuccinate decarboxylase)
O89023	TPP1_MOUSE	Tripeptidyl-peptidase 1 (TPP-1) (EC 3.4.14.9) (Lysosomal pepstatin-insensitive protease) (LPIC) (Tripeptidyl aminopeptidase) (Tripeptidyl-peptidase I) (TPP-I)
O89051	ITM2B_MOUSE	Integral membrane protein 2B (Immature BRI2) (imBRI2) (Protein E25B) (Transmembrane protein BRI) (Bri) [Cleaved into: BRI2, membrane form (Mature BRI2) (mBRI2); BRI2 intracellular domain (BRI2 ICD); BRI2C, soluble form; Bri23 peptide (Bri2-23) (ABri23) (C-terminal peptide) (P23 peptide)]
O89053	COR1A_MOUSE	Coronin-1A (Coronin-like protein A) (Clipin-A) (Coronin-like protein p57) (Tryptophan aspartate-containing coat protein) (TACO)
O89079	COPE_MOUSE	Coatomer subunit epsilon (Epsilon-coat protein) (Epsilon-COP)
P01872	IGHM_MOUSE	Immunoglobulin heavy constant mu [Cleaved into: Mu' chain (55 kDa mu' chain)]
P01898	HA10_MOUSE	H-2 class I histocompatibility antigen, Q10 alpha chain
P01899	HA11_MOUSE	H-2 class I histocompatibility antigen, D-B alpha chain (H-2D(B))
P01901	HA1B_MOUSE	H-2 class I histocompatibility antigen, K-B alpha chain (H-2K(B))
P02088	HBB1_MOUSE	Hemoglobin subunit beta-1 (Beta-1-globin) (Hemoglobin beta-1 chain) (Hemoglobin beta-major chain)
P02301	H3C_MOUSE	Histone H3.3C (Embryonic)

P03911	NU4M_MOUSE	NADH-ubiquinone oxidoreductase chain 4 (EC 7.1.1.2) (NADH dehydrogenase subunit 4)
P04186	CFAB_MOUSE	Complement factor B (EC 3.4.21.47) (C3/C5 convertase) [Cleaved into: Complement factor B Ba fragment; Complement factor B Bb fragment]
P04223	HA1K_MOUSE	H-2 class I histocompatibility antigen, K-K alpha chain (H-2K(K))
P05064	ALDOA_MOUSE	Fructose-bisphosphate aldolase A (EC 4.1.2.13) (Aldolase 1) (Muscle-type aldolase)
P05132	KAPCA_MOUSE	cAMP-dependent protein kinase catalytic subunit alpha (PKA C-alpha) (EC 2.7.11.11)
P05201	AATC_MOUSE	Aspartate aminotransferase, cytoplasmic (cAspAT) (EC 2.6.1.1) (EC 2.6.1.3) (Cysteine aminotransferase, cytoplasmic) (Cysteine transaminase, cytoplasmic) (cCAT) (Glutamate oxaloacetate transaminase 1) (Transaminase A)
P05202	AATM_MOUSE	Aspartate aminotransferase, mitochondrial (mAspAT) (EC 2.6.1.1) (EC 2.6.1.7) (Fatty acid-binding protein) (FABP-1) (Glutamate oxaloacetate transaminase 2) (Kynurenine aminotransferase 4) (Kynurenine aminotransferase IV) (Kynurenine--oxoglutarate transaminase 4) (Kynurenine--oxoglutarate transaminase IV) (Plasma membrane-associated fatty acid-binding protein) (FABPpm) (Transaminase A)
P05480	SRC_MOUSE	Neuronal proto-oncogene tyrosine-protein kinase Src (EC 2.7.10.2) (Proto-oncogene c-Src) (pp60c-src) (p60-Src)
P06151	LDHA_MOUSE	L-lactate dehydrogenase A chain (LDH-A) (EC 1.1.1.27) (LDH muscle subunit) (LDH-M)
P06800	PTPRC_MOUSE	Receptor-type tyrosine-protein phosphatase C (EC 3.1.3.48) (Leukocyte common antigen) (L-CA) (Lymphocyte antigen 5) (Ly-5) (T200) (CD antigen CD45)
P07091	S10A4_MOUSE	Protein S100-A4 (Metastasin) (Metastatic cell protein) (PEL98) (Placental calcium-binding protein) (Protein 18A2) (Protein Mts1) (S100 calcium-binding protein A4)
P07742	RIR1_MOUSE	Ribonucleoside-diphosphate reductase large subunit (EC 1.17.4.1) (Ribonucleoside-diphosphate reductase subunit M1) (Ribonucleotide reductase large subunit)
P07901	HS90A_MOUSE	Heat shock protein HSP 90-alpha (Heat shock 86 kDa) (HSP 86) (HSP86) (Tumor-specific transplantation 86 kDa antigen) (TSTA)
P08003	PDIA4_MOUSE	Protein disulfide-isomerase A4 (EC 5.3.4.1) (Endoplasmic reticulum resident protein 72) (ER protein 72) (ERp-72) (ERp72)
P08030	APT_MOUSE	Adenine phosphoribosyltransferase (APRT) (EC 2.4.2.7)
P08101	FCGR2_MOUSE	Low affinity immunoglobulin gamma Fc region receptor II (Fc gamma receptor IIB) (Fc-gamma RII) (Fc-gamma-RIIB) (FcRII) (IgG Fc receptor II beta) (Lymphocyte antigen 17) (Ly-17) (CD antigen CD32)
P08103	HCK_MOUSE	Tyrosine-protein kinase HCK (EC 2.7.10.2) (B-cell/myeloid kinase) (BMK) (Hematopoietic cell kinase) (Hemopoietic cell kinase) (p56-HCK/p59-HCK)

P08249	MDHM_MOUSE	Malate dehydrogenase, mitochondrial (EC 1.1.1.37)
P08508	FCGR3_MOUSE	Low affinity immunoglobulin gamma Fc region receptor III (IgG Fc receptor III) (Fc-gamma RIII) (FcRIII) (CD antigen CD16)
P08556	RASN_MOUSE	GTPase NRas (Transforming protein N-Ras)
P08752	GNAI2_MOUSE	Guanine nucleotide-binding protein G(i) subunit alpha-2 (Adenylate cyclase-inhibiting G alpha protein)
P09055	ITB1_MOUSE	Integrin beta-1 (Fibronectin receptor subunit beta) (VLA-4 subunit beta) (CD antigen CD29)
P09405	NUCL_MOUSE	Nucleolin (Protein C23)
P09411	PGK1_MOUSE	Phosphoglycerate kinase 1 (EC 2.7.2.3)
P09803	CADH1_MOUSE	Cadherin-1 (ARC-1) (Epithelial cadherin) (E-cadherin) (Uvomorulin) (CD antigen CD324) [Cleaved into: E-Cad/CTF1; E-Cad/CTF2; E-Cad/CTF3]
P0C0S6	H2AZ_MOUSE	Histone H2A.Z (H2A/z)
P0C5E4	PTPRQ_MOUSE	Phosphatidylinositol phosphatase PTPRQ (EC 3.1.3.-) (Receptor-type tyrosine-protein phosphatase Q) (PTP-RQ) (R-PTP-Q) (EC 3.1.3.48)
P10107	ANXA1_MOUSE	Annexin A1 (Annexin I) (Annexin-1) (Calpactin II) (Calpactin-2) (Chromobindin-9) (Lipocortin I) (Phospholipase A2 inhibitory protein) (p35)
P10639	THIO_MOUSE	Thioredoxin (Trx) (ATL-derived factor) (ADF)
P10833	RRAS_MOUSE	Ras-related protein R-Ras (p23)
P10852	4F2_MOUSE	4F2 cell-surface antigen heavy chain (4F2hc) (Solute carrier family 3 member 2) (CD antigen CD98)
P11031	TCP4_MOUSE	Activated RNA polymerase II transcriptional coactivator p15 (Positive cofactor 4) (PC4) (SUB1 homolog) (Single-stranded DNA-binding protein p9) (p14)
P11247	PERM_MOUSE	Myeloperoxidase (MPO) (EC 1.11.2.2) [Cleaved into: Myeloperoxidase light chain; Myeloperoxidase heavy chain]
P11928	OAS1A_MOUSE	2'-5'-oligoadenylate synthase 1A ((2-5')oligo(A) synthase 1A) (2-5A synthase 1A) (EC 2.7.7.84) (p42 OAS)
P11983	TCPA_MOUSE	T-complex protein 1 subunit alpha (TCP-1-alpha) (CCT-alpha) (Tailless complex polypeptide 1A) (TCP-1-A) (Tailless complex polypeptide 1B) (TCP-1-B)
P12265	BGLR_MOUSE	Beta-glucuronidase (EC 3.2.1.31)
P13020	GELS_MOUSE	Gelsolin (Actin-depolymerizing factor) (ADF) (Brevin)
P13597	ICAM1_MOUSE	Intercellular adhesion molecule 1 (ICAM-1) (MALA-2) (MyD10) (CD antigen CD54)
P13609	SRGN_MOUSE	Serglycin (Mastocytoma proteoglycan core protein) (Secretory granule proteoglycan core protein) (gp600)
P14115	RL27A_MOUSE	60S ribosomal protein L27a (L29)
P14131	RS16_MOUSE	40S ribosomal protein S16
P14148	RL7_MOUSE	60S ribosomal protein L7
P14152	MDHC_MOUSE	Malate dehydrogenase, cytoplasmic (EC 1.1.1.37) (Cytosolic malate dehydrogenase)

P14206	RSSA_MOUSE	40S ribosomal protein SA (37 kDa laminin receptor precursor) (37LRP) (37 kDa oncofetal antigen) (37/67 kDa laminin receptor) (LRP/LR) (67 kDa laminin receptor) (67LR) (Laminin receptor 1) (LamR) (Laminin-binding protein precursor p40) (LBP/p40) (OFA/iLRP)
P14211	CALR_MOUSE	Calreticulin (CRP55) (Calregulin) (Endoplasmic reticulum resident protein 60) (ERp60) (HACBP)
P14426	HA13_MOUSE	H-2 class I histocompatibility antigen, D-K alpha chain (H-2D(K))
P14427	HA14_MOUSE	H-2 class I histocompatibility antigen, D-P alpha chain (H-2D(P))
P14429	HA17_MOUSE	H-2 class I histocompatibility antigen, Q7 alpha chain (QA-2 antigen)
P14430	HA18_MOUSE	H-2 class I histocompatibility antigen, Q8 alpha chain
P14434	HA2B_MOUSE	H-2 class II histocompatibility antigen, A-B alpha chain (IAalpha)
P14436	HA2R_MOUSE	H-2 class II histocompatibility antigen, A-R alpha chain
P14483	HB2A_MOUSE	H-2 class II histocompatibility antigen, A beta chain
P14685	PSMD3_MOUSE	26S proteasome non-ATPase regulatory subunit 3 (26S proteasome regulatory subunit RPN3) (26S proteasome regulatory subunit S3) (Proteasome subunit p58) (Transplantation antigen P91A) (Tum-P91A antigen)
P14824	ANXA6_MOUSE	Annexin A6 (67 kDa calelectrin) (Annexin VI) (Annexin-6) (Calphobindin-II) (CPB-II) (Chromobindin-20) (Lipocortin VI) (Protein III) (p68) (p70)
P14869	RLA0_MOUSE	60S acidic ribosomal protein P0 (60S ribosomal protein L10E)
P15105	GLNA_MOUSE	Glutamine synthetase (GS) (EC 6.3.1.2) (Glutamate--ammonia ligase) (Palmitoyltransferase GLUL) (EC 2.3.1.225)
P15379	CD44_MOUSE	CD44 antigen (Extracellular matrix receptor III) (ECMR-III) (GP90 lymphocyte homing/adhesion receptor) (HUTCH-I) (Hermes antigen) (Hyaluronate receptor) (Lymphocyte antigen 24) (Ly-24) (Phagocytic glycoprotein 1) (PGP-1) (Phagocytic glycoprotein I) (PGP-I) (CD antigen CD44)
P15532	NDKA_MOUSE	Nucleoside diphosphate kinase A (NDK A) (NDP kinase A) (EC 2.7.4.6) (Metastasis inhibition factor NM23) (NDPK-A) (Tumor metastatic process-associated protein) (nm23-M1)
P15535	B4GT1_MOUSE	Beta-1,4-galactosyltransferase 1 (Beta-1,4-GalTase 1) (Beta4Gal-T1) (b4Gal-T1) (EC 2.4.1.-) (Beta-N-acetylglucosaminyl-glycolipid beta-1,4-galactosyltransferase) (Beta-N-acetylglucosaminylglycopeptide beta-1,4-galactosyltransferase) (EC 2.4.1.38) (Lactose synthase A protein) (EC 2.4.1.22) (N-acetyllactosamine synthase) (EC 2.4.1.90) (Nal synthase) (UDP-Gal:beta-GlcNAc beta-1,4-galactosyltransferase 1) (UDP-galactose:beta-N-

		acetylglucosamine beta-1,4-galactosyltransferase 1) [Cleaved into: Processed beta-1,4-galactosyltransferase 1]
P15702	LEUK_MOUSE	Leukosialin (B-cell differentiation antigen LP-3) (Leukocyte sialoglycoprotein) (Lymphocyte antigen 48) (Ly-48) (Sialophorin) (CD antigen CD43) [Cleaved into: CD43 cytoplasmic tail (CD43-ct) (CD43ct)]
P15864	H12_MOUSE	Histone H1.2 (H1 VAR.1) (H1c)
P16045	LEG1_MOUSE	Galectin-1 (Gal-1) (14 kDa lectin) (Beta-galactoside-binding lectin L-14-I) (Galaptin) (Lactose-binding lectin 1) (Lectin galactoside-binding soluble 1) (S-Lac lectin 1)
P16330	CN37_MOUSE	2',3'-cyclic-nucleotide 3'-phosphodiesterase (CNP) (CNPase) (EC 3.1.4.37)
P16460	ASSY_MOUSE	Argininosuccinate synthase (EC 6.3.4.5) (Citrulline--aspartate ligase)
P16546	SPTN1_MOUSE	Spectrin alpha chain, non-erythrocytic 1 (Alpha-II spectrin) (Fodrin alpha chain)
P16858	G3P_MOUSE	Glyceraldehyde-3-phosphate dehydrogenase (GAPDH) (EC 1.2.1.12) (Peptidyl-cysteine S-nitrosylase GAPDH) (EC 2.6.99.-)
P17047	LAMP2_MOUSE	Lysosome-associated membrane glycoprotein 2 (LAMP-2) (Lysosome-associated membrane protein 2) (CD107 antigen-like family member B) (Lysosomal membrane glycoprotein type B) (LGP-B) (CD antigen CD107b)
P17182	ENOA_MOUSE	Alpha-enolase (EC 4.2.1.11) (2-phospho-D-glycerate hydro-lyase) (Enolase 1) (Non-neural enolase) (NNE)
P17426	AP2A1_MOUSE	AP-2 complex subunit alpha-1 (100 kDa coated vesicle protein A) (Adaptor protein complex AP-2 subunit alpha-1) (Adaptor-related protein complex 2 subunit alpha-1) (Alpha-adaptin A) (Alpha1-adaptin) (Clathrin assembly protein complex 2 alpha-A large chain) (Plasma membrane adaptor HA2/AP2 adaptin alpha A subunit)
P17427	AP2A2_MOUSE	AP-2 complex subunit alpha-2 (100 kDa coated vesicle protein C) (Adaptor protein complex AP-2 subunit alpha-2) (Adaptor-related protein complex 2 subunit alpha-2) (Alpha-adaptin C) (Alpha2-adaptin) (Clathrin assembly protein complex 2 alpha-C large chain) (Plasma membrane adaptor HA2/AP2 adaptin alpha C subunit)
P17439	GLCM_MOUSE	Lysosomal acid glucosylceramidase (Lysosomal acid GCase) (EC 3.2.1.45) (Acid beta-glucosidase) (Beta-glucocerebrosidase) (Cholesterol glucosyltransferase) (SGTase) (EC 2.4.1.-) (Cholesteryl-beta-glucosidase) (EC 3.2.1.104) (D-glucosyl-N-acylsphingosine glucohydrolase)
P17742	PPIA_MOUSE	Peptidyl-prolyl cis-trans isomerase A (PPIase A) (EC 5.2.1.8) (Cyclophilin A) (Cyclosporin A-binding protein) (Rotamase A) (SP18) [Cleaved into: Peptidyl-prolyl cis-trans isomerase A, N-terminally processed]
P17751	TPIS_MOUSE	Triosephosphate isomerase (TIM) (EC 5.3.1.1) (Methylglyoxal synthase) (EC 4.2.3.3) (Triose-phosphate isomerase)
P17918	PCNA_MOUSE	Proliferating cell nuclear antigen (PCNA) (Cyclin)

P18181	CD48_MOUSE	CD48 antigen (BCM1 surface antigen) (BLAST-1) (HM48-1) (MRC OX-45 surface antigen) (SLAM family member 2) (SLAMF2) (Signaling lymphocytic activation molecule 2) (sgp-60) (CD antigen CD48)
P18572	BASI_MOUSE	Basigin (Basic immunoglobulin superfamily) (HT7 antigen) (Membrane glycoprotein gp42) (CD antigen CD147)
P18654	KS6A3_MOUSE	Ribosomal protein S6 kinase alpha-3 (S6K-alpha-3) (EC 2.7.11.1) (90 kDa ribosomal protein S6 kinase 3) (p90-RSK 3) (p90RSK3) (MAP kinase-activated protein kinase 1b) (MAPK-activated protein kinase 1b) (MAPKAP kinase 1b) (MAPKAPK-1b) (Ribosomal S6 kinase 2) (RSK-2) (pp90RSK2)
P18760	COF1_MOUSE	Cofilin-1 (Cofilin, non-muscle isoform)
P19091	ANDR_MOUSE	Androgen receptor (Dihydrotestosterone receptor) (Nuclear receptor subfamily 3 group C member 4)
P19096	FAS_MOUSE	Fatty acid synthase (EC 2.3.1.85) [Includes: [Acyl-carrier-protein] S-acetyltransferase (EC 2.3.1.38); [Acyl-carrier-protein] S-malonyltransferase (EC 2.3.1.39); 3-oxoacyl-[acyl-carrier-protein] synthase (EC 2.3.1.41); 3-oxoacyl-[acyl-carrier-protein] reductase (EC 1.1.1.100); 3-hydroxyacyl-[acyl-carrier-protein] dehydratase (EC 4.2.1.59); Enoyl-[acyl-carrier-protein] reductase (EC 1.3.1.39); Oleoyl-[acyl-carrier-protein] hydrolase (EC 3.1.2.14)]
P19157	GSTP1_MOUSE	Glutathione S-transferase P 1 (Gst P1) (EC 2.5.1.18) (GST YF-YF) (GST class-pi) (GST-piB) (Preadipocyte growth factor)
P19221	THRB_MOUSE	Prothrombin (EC 3.4.21.5) (Coagulation factor II) [Cleaved into: Activation peptide fragment 1; Activation peptide fragment 2; Thrombin light chain; Thrombin heavy chain]
P19253	RL13A_MOUSE	60S ribosomal protein L13a (Transplantation antigen P198) (Tum-P198 antigen)
P20029	BIP_MOUSE	Endoplasmic reticulum chaperone BiP (EC 3.6.4.10) (78 kDa glucose-regulated protein) (GRP-78) (Binding-immunoglobulin protein) (BiP) (Heat shock protein 70 family protein 5) (HSP70 family protein 5) (Heat shock protein family A member 5) (Immunoglobulin heavy chain-binding protein)
P21107	TPM3_MOUSE	Tropomyosin alpha-3 chain (Gamma-tropomyosin) (Tropomyosin-3)
P21271	MYO5B_MOUSE	Unconventional myosin-Vb
P21279	GNAQ_MOUSE	Guanine nucleotide-binding protein G(q) subunit alpha (Guanine nucleotide-binding protein alpha-q)
P21460	CYTC_MOUSE	Cystatin-C (Cystatin-3)
P21958	TAP1_MOUSE	Antigen peptide transporter 1 (APT1) (ATP-binding cassette sub-family B member 2) (Histocompatibility antigen modifier 1) (Peptide transporter TAP1)

P21981	TGM2_MOUSE	Protein-glutamine gamma-glutamyltransferase 2 (EC 2.3.2.13) (Tissue transglutaminase) (Transglutaminase C) (TG(C)) (TGC) (TGase C) (Transglutaminase-2) (TGase-2)
P22437	PGH1_MOUSE	Prostaglandin G/H synthase 1 (EC 1.14.99.1) (Cyclooxygenase-1) (COX-1) (Prostaglandin H2 synthase 1) (PGH synthase 1) (PGHS-1) (PHS 1) (Prostaglandin-endoperoxide synthase 1)
P22777	PAI1_MOUSE	Plasminogen activator inhibitor 1 (PAI) (PAI-1) (Endothelial plasminogen activator inhibitor) (Serpin E1)
P22892	AP1G1_MOUSE	AP-1 complex subunit gamma-1 (Adaptor protein complex AP-1 subunit gamma-1) (Adaptor-related protein complex 1 subunit gamma-1) (Clathrin assembly protein complex 1 gamma-1 large chain) (Gamma-adaptin) (Gamma1-adaptin) (Golgi adaptor HA1/AP1 adaptin subunit gamma-1)
P23116	EIF3A_MOUSE	Eukaryotic translation initiation factor 3 subunit A (eIF3a) (Centrosomin) (Eukaryotic translation initiation factor 3 subunit 10) (eIF-3-theta) (eIF3 p167) (eIF3 p180) (eIF3 p185) (p162)
P23492	PNPH_MOUSE	Purine nucleoside phosphorylase (PNP) (EC 2.4.2.1) (Inosine phosphorylase) (Inosine-guanosine phosphorylase)
P23591	FCL_MOUSE	GDP-L-fucose synthase (EC 1.1.1.271) (GDP-4-keto-6-deoxy-D-mannose-3,5-epimerase-4-reductase) (Protein FX) (Red cell NADP(H)-binding protein) (Transplantation antigen P35B) (Tum-P35B antigen)
P24063	ITAL_MOUSE	Integrin alpha-L (CD11 antigen-like family member A) (Leukocyte adhesion glycoprotein LFA-1 alpha chain) (LFA-1A) (Leukocyte function-associated molecule 1 alpha chain) (Lymphocyte antigen 15) (Ly-15) (CD antigen CD11a)
P24270	CATA_MOUSE	Catalase (EC 1.11.1.6)
P24369	PPIB_MOUSE	Peptidyl-prolyl cis-trans isomerase B (PPIase B) (EC 5.2.1.8) (CYP-S1) (Cyclophilin B) (Rotamase B) (S-cyclophilin) (SCYLP)
P24452	CAPG_MOUSE	Macrophage-capping protein (Actin regulatory protein CAP-G) (Actin-capping protein GCAP39) (Myc basic motif homolog 1)
P24527	LKHA4_MOUSE	Leukotriene A-4 hydrolase (LTA-4 hydrolase) (EC 3.3.2.6) (Leukotriene A(4) hydrolase)
P24547	IMDH2_MOUSE	Inosine-5'-monophosphate dehydrogenase 2 (IMP dehydrogenase 2) (IMPD 2) (IMPDH 2) (EC 1.1.1.205) (IMPDH-II)
P24668	MPRD_MOUSE	Cation-dependent mannose-6-phosphate receptor (CD Man-6-P receptor) (CD-MPR) (46 kDa mannose 6-phosphate receptor) (MPR 46)
P25444	RS2_MOUSE	40S ribosomal protein S2 (40S ribosomal protein S4) (Protein LLRep3)
P25799	NFKB1_MOUSE	Nuclear factor NF-kappa-B p105 subunit (DNA-binding factor KBF1) (EBP-1) (NF-kappa-B1 p84/NF-kappa-B1 p98) (Nuclear factor of kappa light polypeptide gene

		enhancer in B-cells 1) [Cleaved into: Nuclear factor NF-kappa-B p50 subunit]
P25911	LYN_MOUSE	Tyrosine-protein kinase Lyn (EC 2.7.10.2) (V-yes-1 Yamaguchi sarcoma viral related oncogene homolog) (p53Lyn) (p56Lyn)
P26039	TLN1_MOUSE	Talin-1
P26040	EZRI_MOUSE	Ezrin (Cytovillin) (Villin-2) (p81)
P26041	MOES_MOUSE	Moesin (Membrane-organizing extension spike protein)
P26043	RADI_MOUSE	Radixin (ESP10)
P26231	CTNA1_MOUSE	Catenin alpha-1 (102 kDa cadherin-associated protein) (Alpha E-catenin) (CAP102)
P26443	DHE3_MOUSE	Glutamate dehydrogenase 1, mitochondrial (GDH 1) (EC 1.4.1.3)
P26450	P85A_MOUSE	Phosphatidylinositol 3-kinase regulatory subunit alpha (PI3-kinase regulatory subunit alpha) (PI3K regulatory subunit alpha) (PtdIns-3-kinase regulatory subunit alpha) (Phosphatidylinositol 3-kinase 85 kDa regulatory subunit alpha) (PI3-kinase subunit p85-alpha) (PtdIns-3-kinase regulatory subunit p85-alpha)
P26516	PSMD7_MOUSE	26S proteasome non-ATPase regulatory subunit 7 (26S proteasome regulatory subunit RPN8) (26S proteasome regulatory subunit S12) (Mov34 protein) (Proteasome subunit p40)
P26638	SYSC_MOUSE	Serine--tRNA ligase, cytoplasmic (EC 6.1.1.11) (Seryl-tRNA synthetase) (SerRS) (Seryl-tRNA(Ser/Sec) synthetase)
P26645	MARCS_MOUSE	Myristoylated alanine-rich C-kinase substrate (MARCKS)
P27005	S10A8_MOUSE	Protein S100-A8 (Calgranulin-A) (Chemotactic cytokine CP-10) (Leukocyte L1 complex light chain) (Migration inhibitory factor-related protein 8) (MRP-8) (p8) (Pro-inflammatory S100 cytokine) (S100 calcium-binding protein A8)
P27048	RSMB_MOUSE	Small nuclear ribonucleoprotein-associated protein B (snRNP-B) (snRPB) (Sm protein B) (Sm-B) (SmB)
P27601	GNA13_MOUSE	Guanine nucleotide-binding protein subunit alpha-13 (G alpha-13) (G-protein subunit alpha-13)
P27612	PLAP_MOUSE	Phospholipase A-2-activating protein (PLA2P) (PLAP)
P27659	RL3_MOUSE	60S ribosomal protein L3 (J1 protein)
P27661	H2AX_MOUSE	Histone H2AX (H2a/x) (Histone H2A.X)
P27773	PDIA3_MOUSE	Protein disulfide-isomerase A3 (EC 5.3.4.1) (58 kDa glucose-regulated protein) (58 kDa microsomal protein) (p58) (Disulfide isomerase ER-60) (Endoplasmic reticulum resident protein 57) (ER protein 57) (ERp57) (Endoplasmic reticulum resident protein 60) (ER protein 60) (ERp60)
P28076	PSB9_MOUSE	Proteasome subunit beta type-9 (EC 3.4.25.1) (LMP-2d) (Low molecular mass protein 2) (Macropain chain 7) (Multicatalytic endopeptidase complex chain 7)

		(Proteasome chain 7) (Proteasome subunit beta-1i) (Really interesting new gene 12 protein)
P28271	ACOC_MOUSE	Cytoplasmic aconitate hydratase (Aconitase) (EC 4.2.1.3) (Citrate hydro-lyase) (Iron regulatory protein 1) (IRP1) (Iron-responsive element-binding protein 1) (IRE-BP 1)
P28656	NP1L1_MOUSE	Nucleosome assembly protein 1-like 1 (Brain protein DN38) (NAP-1-related protein)
P28738	KIF5C_MOUSE	Kinesin heavy chain isoform 5C (Kinesin heavy chain neuron-specific 2)
P28843	DPP4_MOUSE	Dipeptidyl peptidase 4 (EC 3.4.14.5) (Dipeptidyl peptidase IV) (DPP IV) (T-cell activation antigen CD26) (Thymocyte-activating molecule) (THAM) (CD antigen CD26) [Cleaved into: Dipeptidyl peptidase 4 membrane form (Dipeptidyl peptidase IV membrane form); Dipeptidyl peptidase 4 soluble form (Dipeptidyl peptidase IV soluble form)]
P28867	KPCD_MOUSE	Protein kinase C delta type (EC 2.7.11.13) (Tyrosine-protein kinase PRKCD) (EC 2.7.10.2) (nPKC-delta) [Cleaved into: Protein kinase C delta type regulatory subunit; Protein kinase C delta type catalytic subunit (Sphingosine-dependent protein kinase-1) (SDK1)]
P29341	PABP1_MOUSE	Polyadenylate-binding protein 1 (PABP-1) (Poly(A)-binding protein 1)
P29351	PTN6_MOUSE	Tyrosine-protein phosphatase non-receptor type 6 (EC 3.1.3.48) (70Z-SHP) (Hematopoietic cell protein-tyrosine phosphatase) (PTPTY-42) (Protein-tyrosine phosphatase 1C) (PTP-1C) (SH-PTP1) (SHP-1)
P29387	GBB4_MOUSE	Guanine nucleotide-binding protein subunit beta-4 (Transducin beta chain 4)
P29391	FRIL1_MOUSE	Ferritin light chain 1 (Ferritin L subunit 1)
P29416	HEXA_MOUSE	Beta-hexosaminidase subunit alpha (EC 3.2.1.52) (Beta-N-acetylhexosaminidase subunit alpha) (Hexosaminidase subunit A) (N-acetyl-beta-glucosaminidase subunit alpha)
P30355	AL5AP_MOUSE	Arachidonate 5-lipoxygenase-activating protein (FLAP) (MK-886-binding protein)
P30416	FKBP4_MOUSE	Peptidyl-prolyl cis-trans isomerase FKBP4 (PPIase FKBP4) (EC 5.2.1.8) (52 kDa FK506-binding protein) (52 kDa FKBP) (FKBP-52) (59 kDa immunophilin) (p59) (FK506-binding protein 4) (FKBP-4) (FKBP59) (HSP-binding immunophilin) (HBI) (Immunophilin FKBP52) (Rotamase) [Cleaved into: Peptidyl-prolyl cis-trans isomerase FKBP4, N-terminally processed]
P30677	GNA14_MOUSE	Guanine nucleotide-binding protein subunit alpha-14 (G alpha-14) (G-protein subunit alpha-14)
P30681	HMGB2_MOUSE	High mobility group protein B2 (High mobility group protein 2) (HMG-2)
P30999	CTND1_MOUSE	Catenin delta-1 (Cadherin-associated Src substrate) (CAS) (p120 catenin) (p120(ctn)) (p120(cas))
P31230	AIMP1_MOUSE	Aminoacyl tRNA synthase complex-interacting multifunctional protein 1 (Multisynthase complex auxiliary

		component p43) [Cleaved into: Endothelial monocyte-activating polypeptide 2 (EMAP-2) (Endothelial monocyte-activating polypeptide II) (EMAP-II) (Small inducible cytokine subfamily E member 1)]
P31725	S10A9_MOUSE	Protein S100-A9 (Calgranulin-B) (Leukocyte L1 complex heavy chain) (Migration inhibitory factor-related protein 14) (MRP-14) (p14) (S100 calcium-binding protein A9)
P31750	AKT1_MOUSE	RAC-alpha serine/threonine-protein kinase (EC 2.7.11.1) (AKT1 kinase) (Protein kinase B) (PKB) (Protein kinase B alpha) (PKB alpha) (Proto-oncogene c-Akt) (RAC-PK-alpha) (Thymoma viral proto-oncogene)
P31786	ACBP_MOUSE	Acyl-CoA-binding protein (ACBP) (Diazepam-binding inhibitor) (DBI) (Endozepine) (EP)
P31809	CEAM1_MOUSE	Carcinoembryonic antigen-related cell adhesion molecule 1 (Biliary glycoprotein 1) (BGP-1) (Biliary glycoprotein D) (MHVR1) (Murine hepatitis virus receptor) (MHV-R) (CD antigen CD66a)
P32067	LA_MOUSE	Lupus La protein homolog (La autoantigen homolog) (La ribonucleoprotein)
P32233	DRG1_MOUSE	Developmentally-regulated GTP-binding protein 1 (DRG-1) (Neural precursor cell expressed developmentally down-regulated protein 3) (NEDD-3) (Translation factor GTPase DRG1) (TRAFAC GTPase DRG1) (EC 3.6.5.-)
P32921	SYWC_MOUSE	Tryptophan--tRNA ligase, cytoplasmic (EC 6.1.1.2) (Tryptophanyl-tRNA synthetase) (TrpRS) [Cleaved into: T1-TrpRS; T2-TrpRS]
P33435	MMP13_MOUSE	Collagenase 3 (EC 3.4.24.-) (Matrix metalloproteinase-13) (MMP-13)
P33622	APOC3_MOUSE	Apolipoprotein C-III (Apo-CIII) (ApoC-III) (Apolipoprotein C3)
P33766	FPR1_MOUSE	fMet-Leu-Phe receptor (fMLP receptor) (N-formyl peptide receptor) (FPR) (N-formylpeptide chemoattractant receptor)
P34884	MIF_MOUSE	Macrophage migration inhibitory factor (MIF) (EC 5.3.2.1) (Delayed early response protein 6) (DER6) (Glycosylation-inhibiting factor) (GIF) (L-dopachrome isomerase) (L-dopachrome tautomerase) (EC 5.3.3.12) (Phenylpyruvate tautomerase)
P35123	UBP4_MOUSE	Ubiquitin carboxyl-terminal hydrolase 4 (EC 3.4.19.12) (Deubiquitinating enzyme 4) (Ubiquitin thioesterase 4) (Ubiquitin-specific-processing protease 4) (Ubiquitous nuclear protein)
P35276	RAB3D_MOUSE	Ras-related protein Rab-3D
P35278	RAB5C_MOUSE	Ras-related protein Rab-5C
P35279	RAB6A_MOUSE	Ras-related protein Rab-6A (Rab-6)
P35282	RAB21_MOUSE	Ras-related protein Rab-21 (Rab-12)
P35293	RAB18_MOUSE	Ras-related protein Rab-18

P35343	CXCR2_MOUSE	C-X-C chemokine receptor type 2 (CXC-R2) (CXCR-2) (GRO/MGSA receptor) (High affinity interleukin-8 receptor B) (IL-8R B) (CD antigen CD182)
P35456	UPAR_MOUSE	Urokinase plasminogen activator surface receptor (U-PAR) (uPAR) (CD antigen CD87)
P35700	PRDX1_MOUSE	Peroxiredoxin-1 (EC 1.11.1.15) (Macrophage 23 kDa stress protein) (Osteoblast-specific factor 3) (OSF-3) (Thioredoxin peroxidase 2) (Thioredoxin-dependent peroxide reductase 2)
P35821	PTN1_MOUSE	Tyrosine-protein phosphatase non-receptor type 1 (EC 3.1.3.48) (Protein-tyrosine phosphatase 1B) (PTP-1B) (Protein-tyrosine phosphatase HA2) (PTP-HA2)
P35979	RL12_MOUSE	60S ribosomal protein L12
P35980	RL18_MOUSE	60S ribosomal protein L18
P36371	TAP2_MOUSE	Antigen peptide transporter 2 (APT2) (ATP-binding cassette sub-family B member 3) (Histocompatibility antigen modifier 2)
P36536	SAR1A_MOUSE	GTP-binding protein SAR1a
P40124	CAP1_MOUSE	Adenylyl cyclase-associated protein 1 (CAP 1)
P40142	TKT_MOUSE	Transketolase (TK) (EC 2.2.1.1) (P68)
P40237	CD82_MOUSE	CD82 antigen (C33 antigen) (IA4) (Inducible membrane protein R2) (Metastasis suppressor Kangai-1 homolog) (CD antigen CD82)
P40336	VP26A_MOUSE	Vacuolar protein sorting-associated protein 26A (H<beta>58 protein) (H beta 58) (Vesicle protein sorting 26A) (mVPS26)
P41233	ABCA1_MOUSE	Phospholipid-transporting ATPase ABCA1 (EC 7.6.2.1) (ATP-binding cassette sub-family A member 1) (ATP-binding cassette transporter 1) (ABC-1) (ATP-binding cassette 1)
P41241	CSK_MOUSE	Tyrosine-protein kinase CSK (EC 2.7.10.2) (C-Src kinase) (Protein-tyrosine kinase MPK-2) (p50CSK)
P41731	CD63_MOUSE	CD63 antigen (CD antigen CD63)
P42082	CD86_MOUSE	T-lymphocyte activation antigen CD86 (Activation B7-2 antigen) (Early T-cell costimulatory molecule 1) (ETC-1) (CD antigen CD86)
P42208	SEPT2_MOUSE	Septin-2 (Neural precursor cell expressed developmentally down-regulated protein 5) (NEDD-5)
P42225	STAT1_MOUSE	Signal transducer and activator of transcription 1
P42227	STAT3_MOUSE	Signal transducer and activator of transcription 3 (Acute-phase response factor)
P42230	STA5A_MOUSE	Signal transducer and activator of transcription 5A (Mammary gland factor)
P42232	STA5B_MOUSE	Signal transducer and activator of transcription 5B
P42567	EPS15_MOUSE	Epidermal growth factor receptor substrate 15 (Protein Eps15) (Protein AF-1p)
P42932	TCPQ_MOUSE	T-complex protein 1 subunit theta (TCP-1-theta) (CCT-theta)

P43274	H14_MOUSE	Histone H1.4 (H1 VAR.2) (H1e)
P43275	H11_MOUSE	Histone H1.1 (H1 VAR.3) (Histone H1a) (H1a)
P43276	H15_MOUSE	Histone H1.5 (H1 VAR.5) (H1b)
P43406	ITAV_MOUSE	Integrin alpha-V (Vitronectin receptor subunit alpha) (CD antigen CD51) [Cleaved into: Integrin alpha-V heavy chain; Integrin alpha-V light chain]
P43488	TNFL4_MOUSE	Tumor necrosis factor ligand superfamily member 4 (OX40 ligand) (OX40L) (CD antigen CD252)
P45376	ALDR_MOUSE	Aldo-keto reductase family 1 member B1 (EC 1.1.1.300) (EC 1.1.1.372) (EC 1.1.1.54) (Aldehyde reductase) (Aldo-keto reductase family 1 member B3) (Aldose reductase) (AR) (EC 1.1.1.21)
P46460	NSF_MOUSE	Vesicle-fusing ATPase (EC 3.6.4.6) (N-ethylmaleimide-sensitive fusion protein) (NEM-sensitive fusion protein) (Suppressor of K(+) transport growth defect 2) (Protein SKD2) (Vesicular-fusion protein NSF)
P46467	VPS4B_MOUSE	Vacuolar protein sorting-associated protein 4B (EC 3.6.4.6) (Suppressor of K(+) transport growth defect 1) (Protein SKD1)
P46471	PRS7_MOUSE	26S proteasome regulatory subunit 7 (26S proteasome AAA-ATPase subunit RPT1) (Proteasome 26S subunit ATPase 2) (Protein MSS1)
P46638	RB11B_MOUSE	Ras-related protein Rab-11B
P47738	ALDH2_MOUSE	Aldehyde dehydrogenase, mitochondrial (EC 1.2.1.3) (AHD-M1) (ALDH class 2) (ALDH-E2) (ALDHI)
P47754	CAZA2_MOUSE	F-actin-capping protein subunit alpha-2 (CapZ alpha-2)
P47757	CAPZB_MOUSE	F-actin-capping protein subunit beta (CapZ beta)
P47774	CCR7_MOUSE	C-C chemokine receptor type 7 (C-C CKR-7) (CC-CKR-7) (CCR-7) (Epstein-Barr virus-induced G-protein coupled receptor 1) (EBI1) (EBV-induced G-protein coupled receptor 1) (MIP-3 beta receptor) (CD antigen CD197)
P47791	GSHR_MOUSE	Glutathione reductase, mitochondrial (GR) (GRase) (EC 1.8.1.7)
P47811	MK14_MOUSE	Mitogen-activated protein kinase 14 (MAP kinase 14) (MAPK 14) (EC 2.7.11.24) (CRK1) (Mitogen-activated protein kinase p38 alpha) (MAP kinase p38 alpha)
P47934	CACP_MOUSE	Carnitine O-acetyltransferase (Carnitine acetylase) (EC 2.3.1.7) (Carnitine acetyltransferase) (CAT) (CrAT)
P47955	RLA1_MOUSE	60S acidic ribosomal protein P1
P47962	RL5_MOUSE	60S ribosomal protein L5
P47963	RL13_MOUSE	60S ribosomal protein L13 (A52)
P48455	PP2BC_MOUSE	Serine/threonine-protein phosphatase 2B catalytic subunit gamma isoform (EC 3.1.3.16) (CAM-PRP catalytic subunit) (Calcineurin, testis-specific catalytic subunit) (Calmodulin-dependent calcineurin A subunit gamma isoform)

P49290	PERE_MOUSE	Eosinophil peroxidase (EPO) (EC 1.11.1.7) [Cleaved into: Eosinophil peroxidase light chain; Eosinophil peroxidase heavy chain]
P49300	CLC10_MOUSE	C-type lectin domain family 10 member A (MMGL) (Macrophage asialoglycoprotein-binding protein 1) (M-ASGP-BP-1) (Macrophage galactose/N-acetylgalactosamine-specific lectin)
P49312	ROA1_MOUSE	Heterogeneous nuclear ribonucleoprotein A1 (hnRNP A1) (HDP-1) (Helix-destabilizing protein) (Single-strand-binding protein) (Topoisomerase-inhibitor suppressed) (hnRNP core protein A1) [Cleaved into: Heterogeneous nuclear ribonucleoprotein A1, N-terminally processed]
P49722	PSA2_MOUSE	Proteasome subunit alpha type-2 (EC 3.4.25.1) (Macropain subunit C3) (Multicatalytic endopeptidase complex subunit C3) (Proteasome component C3)
P49769	PSN1_MOUSE	Presenilin-1 (PS-1) (EC 3.4.23.-) (Protein S182) [Cleaved into: Presenilin-1 NTF subunit; Presenilin-1 CTF subunit; Presenilin-1 CTF12 (PS1-CTF12)]
P50247	SAHH_MOUSE	Adenosylhomocysteinase (AdoHcyase) (EC 3.3.1.1) (CUBP) (Liver copper-binding protein) (S-adenosyl-L-homocysteine hydrolase)
P50396	GDIA_MOUSE	Rab GDP dissociation inhibitor alpha (Rab GDI alpha) (Guanosine diphosphate dissociation inhibitor 1) (GDI-1)
P50516	VATA_MOUSE	V-type proton ATPase catalytic subunit A (V-ATPase subunit A) (EC 7.1.2.2) (V-ATPase 69 kDa subunit) (Vacuolar proton pump subunit alpha)
P50518	VATE1_MOUSE	V-type proton ATPase subunit E 1 (V-ATPase subunit E 1) (V-ATPase 31 kDa subunit) (p31) (Vacuolar proton pump subunit E 1)
P50543	S10AB_MOUSE	Protein S100-A11 (Calgizzarin) (Endothelial monocyte-activating polypeptide) (EMAP) (Protein S100-C) (S100 calcium-binding protein A11)
P50580	PA2G4_MOUSE	Proliferation-associated protein 2G4 (IRES-specific cellular trans-acting factor 45 kDa) (ITAF45) (Mpp1) (Proliferation-associated protein 1) (Protein p38-2G4)
P51150	RAB7A_MOUSE	Ras-related protein Rab-7a
P51410	RL9_MOUSE	60S ribosomal protein L9
P51670	CCL9_MOUSE	C-C motif chemokine 9 (CCF18) (Macrophage inflammatory protein 1-gamma) (MIP-1-gamma) (Macrophage inflammatory protein-related protein 2) (MRP-2) (Small-inducible cytokine A9) [Cleaved into: CCL9(29-101); CCL9(30-101); CCL9(31-101)]
P51885	LUM_MOUSE	Lumican (Keratan sulfate proteoglycan lumican) (KSPG lumican)
P52480	KPYM_MOUSE	Pyruvate kinase PKM (EC 2.7.1.40) (Pyruvate kinase muscle isozyme)
P52624	UPP1_MOUSE	Uridine phosphorylase 1 (UPase 1) (UrdPase 1) (EC 2.4.2.3)

P53026	RL10A_MOUSE	60S ribosomal protein L10a (CSA-19) (Neural precursor cell expressed developmentally down-regulated protein 6) (NEDD-6)
P53762	ARNT_MOUSE	Aryl hydrocarbon receptor nuclear translocator (ARNT protein) (Dioxin receptor, nuclear translocator) (Hypoxia-inducible factor 1-beta) (HIF-1-beta) (HIF1-beta)
P53810	PIPNA_MOUSE	Phosphatidylinositol transfer protein alpha isoform (PI-TP-alpha) (PtdIns transfer protein alpha) (PtdInsTP alpha)
P53994	RAB2A_MOUSE	Ras-related protein Rab-2A
P54071	IDHP_MOUSE	Isocitrate dehydrogenase [NADP], mitochondrial (IDH) (EC 1.1.1.42) (ICD-M) (IDP) (NADP(+)-specific ICDH) (Oxalosuccinate decarboxylase)
P54116	STOM_MOUSE	Erythrocyte band 7 integral membrane protein (Protein 7.2b) (Stomatin)
P54729	NUB1_MOUSE	NEDD8 ultimate buster 1 (Negative regulator of ubiquitin-like proteins 1) (Protein BS4)
P54775	PRS6B_MOUSE	26S proteasome regulatory subunit 6B (26S proteasome AAA-ATPase subunit RPT3) (CIP21) (MB67-interacting protein) (MIP224) (Proteasome 26S subunit ATPase 4) (Tat-binding protein 7) (TBP-7)
P54823	DDX6_MOUSE	Probable ATP-dependent RNA helicase DDX6 (EC 3.6.4.13) (ATP-dependent RNA helicase p54) (DEAD box protein 6) (Oncogene RCK homolog)
P55012	S12A2_MOUSE	Solute carrier family 12 member 2 (Basolateral Na-K-Cl symporter) (Bumetanide-sensitive sodium-(potassium)-chloride cotransporter 2)
P55194	3BP1_MOUSE	SH3 domain-binding protein 1 (3BP-1)
P55258	RAB8A_MOUSE	Ras-related protein Rab-8A (Oncogene c-mel)
P55772	ENTP1_MOUSE	Ectonucleoside triphosphate diphosphohydrolase 1 (NTPDase 1) (EC 3.6.1.5) (Ecto-ATP diphosphohydrolase 1) (Ecto-ATPDase 1) (Ecto-ATPase 1) (Ecto-apyrase) (Lymphoid cell activation antigen) (CD antigen CD39)
P56395	CYB5_MOUSE	Cytochrome b5
P56399	UBP5_MOUSE	Ubiquitin carboxyl-terminal hydrolase 5 (EC 3.4.19.12) (Deubiquitinating enzyme 5) (Isopeptidase T) (Ubiquitin thioesterase 5) (Ubiquitin-specific-processing protease 5)
P56477	IRF5_MOUSE	Interferon regulatory factor 5 (IRF-5)
P56565	S10A1_MOUSE	Protein S100-A1 (S-100 protein alpha chain) (S-100 protein subunit alpha) (S100 calcium-binding protein A1)
P56812	PDCD5_MOUSE	Programmed cell death protein 5 (TF-1 cell apoptosis-related protein 19) (Protein TFAR19)
P57716	NICA_MOUSE	Nicastrin
P57746	VATD_MOUSE	V-type proton ATPase subunit D (V-ATPase subunit D) (V-ATPase 28 kDa accessory protein) (Vacuolar proton pump subunit D)
P57759	ERP29_MOUSE	Endoplasmic reticulum resident protein 29 (ERp29)
P57776	EF1D_MOUSE	Elongation factor 1-delta (EF-1-delta)
P57780	ACTN4_MOUSE	Alpha-actinin-4 (Non-muscle alpha-actinin 4)

P57784	RU2A_MOUSE	U2 small nuclear ribonucleoprotein A' (U2 snRNP A')
P57787	MOT4_MOUSE	Monocarboxylate transporter 4 (MCT 4) (Solute carrier family 16 member 3)
P58021	TM9S2_MOUSE	Transmembrane 9 superfamily member 2
P58242	ASM3B_MOUSE	Acid sphingomyelinase-like phosphodiesterase 3b (ASM-like phosphodiesterase 3b) (EC 3.1.4.-)
P58252	EF2_MOUSE	Elongation factor 2 (EF-2)
P58389	PTPA_MOUSE	Serine/threonine-protein phosphatase 2A activator (EC 5.2.1.8) (PP2A, subunit B', PR53 isoform) (Phosphotyrosyl phosphatase activator) (PTPA) (Serine/threonine-protein phosphatase 2A regulatory subunit 4) (Serine/threonine-protein phosphatase 2A regulatory subunit B')
P59108	CPNE2_MOUSE	Copine-2 (Copine II)
P59325	IF5_MOUSE	Eukaryotic translation initiation factor 5 (eIF-5)
P59383	LRRN4_MOUSE	Leucine-rich repeat neuronal protein 4 (Neuronal leucine-rich repeat protein 4) (NLRR-4)
P59708	SF3B6_MOUSE	Splicing factor 3B subunit 6 (Pre-mRNA branch site protein p14) (SF3b 14 kDa subunit)
P59729	RIN3_MOUSE	Ras and Rab interactor 3 (Ras interaction/interference protein 3)
P59999	ARPC4_MOUSE	Actin-related protein 2/3 complex subunit 4 (Arp2/3 complex 20 kDa subunit) (p20-ARC)
P60122	RUVB1_MOUSE	RuvB-like 1 (EC 3.6.4.12) (49 kDa TATA box-binding protein-interacting protein) (49 kDa TBP-interacting protein) (DNA helicase p50) (Pontin 52) (TIP49a)
P60229	EIF3E_MOUSE	Eukaryotic translation initiation factor 3 subunit E (eIF3e) (Eukaryotic translation initiation factor 3 subunit 6) (MMTV integration site 6) (Mammary tumor-associated protein INT-6) (Viral integration site protein INT-6) (eIF-3 p48)
P60335	PCBP1_MOUSE	Poly(rC)-binding protein 1 (Alpha-CP1) (Heterogeneous nuclear ribonucleoprotein E1) (hnRNP E1)
P60710	ACTB_MOUSE	Actin, cytoplasmic 1 (Beta-actin) [Cleaved into: Actin, cytoplasmic 1, N-terminally processed]
P60766	CDC42_MOUSE	Cell division control protein 42 homolog (EC 3.6.5.2) (G25K GTP-binding protein)
P60843	IF4A1_MOUSE	Eukaryotic initiation factor 4A-I (eIF-4A-I) (eIF4A-I) (EC 3.6.4.13) (ATP-dependent RNA helicase eIF4A-1)
P60904	DNJC5_MOUSE	DnaJ homolog subfamily C member 5 (Cysteine string protein) (CSP)
P61021	RAB5B_MOUSE	Ras-related protein Rab-5B
P61027	RAB10_MOUSE	Ras-related protein Rab-10
P61028	RAB8B_MOUSE	Ras-related protein Rab-8B
P61079	UB2D3_MOUSE	Ubiquitin-conjugating enzyme E2 D3 (EC 2.3.2.23) ((E3-independent) E2 ubiquitin-conjugating enzyme D3) (EC 2.3.2.24) (E2 ubiquitin-conjugating enzyme D3) (Ubiquitin carrier protein D3) (Ubiquitin-conjugating enzyme

		E2(17)KB 3) (Ubiquitin-conjugating enzyme E2-17 kDa 3) (Ubiquitin-protein ligase D3)
P61082	UBC12_MOUSE	NEDD8-conjugating enzyme Ubc12 (EC 2.3.2.-) (NEDD8 carrier protein) (Ubiquitin-conjugating enzyme E2 M)
P61087	UBE2K_MOUSE	Ubiquitin-conjugating enzyme E2 K (EC 2.3.2.23) (E2 ubiquitin-conjugating enzyme K) (Huntingtin-interacting protein 2) (HIP-2) (Ubiquitin carrier protein) (Ubiquitin-conjugating enzyme E2-25 kDa) (Ubiquitin-conjugating enzyme E2(25K)) (Ubiquitin-conjugating enzyme E2-25K) (Ubiquitin-protein ligase)
P61089	UBE2N_MOUSE	Ubiquitin-conjugating enzyme E2 N (EC 2.3.2.23) (Bendless-like ubiquitin-conjugating enzyme) (E2 ubiquitin-conjugating enzyme N) (Ubc13) (Ubiquitin carrier protein N) (Ubiquitin-protein ligase N)
P61161	ARP2_MOUSE	Actin-related protein 2 (Actin-like protein 2)
P61164	ACTZ_MOUSE	Alpha-centractin (Centractin) (ARP1) (Actin-RPV) (Centrosome-associated actin homolog)
P61211	ARL1_MOUSE	ADP-ribosylation factor-like protein 1
P61226	RAP2B_MOUSE	Ras-related protein Rap-2b
P61358	RL27_MOUSE	60S ribosomal protein L27
P61750	ARF4_MOUSE	ADP-ribosylation factor 4
P61759	PFD3_MOUSE	Prefoldin subunit 3 (von Hippel-Lindau-binding protein 1) (VBP-1) (VHL-binding protein 1)
P61961	UFM1_MOUSE	Ubiquitin-fold modifier 1
P61971	NTF2_MOUSE	Nuclear transport factor 2 (NTF-2)
P61979	HNRPK_MOUSE	Heterogeneous nuclear ribonucleoprotein K (hnRNP K)
P61982	1433G_MOUSE	14-3-3 protein gamma [Cleaved into: 14-3-3 protein gamma, N-terminally processed]
P62071	RRAS2_MOUSE	Ras-related protein R-Ras2
P62075	TIM13_MOUSE	Mitochondrial import inner membrane translocase subunit Tim13
P62082	RS7_MOUSE	40S ribosomal protein S7
P62137	PP1A_MOUSE	Serine/threonine-protein phosphatase PP1-alpha catalytic subunit (PP-1A) (EC 3.1.3.16)
P62141	PP1B_MOUSE	Serine/threonine-protein phosphatase PP1-beta catalytic subunit (PP-1B) (EC 3.1.3.16) (EC 3.1.3.53)
P62192	PRS4_MOUSE	26S proteasome regulatory subunit 4 (P26s4) (26S proteasome AAA-ATPase subunit RPT2) (Proteasome 26S subunit ATPase 1)
P62196	PRS8_MOUSE	26S proteasome regulatory subunit 8 (26S proteasome AAA-ATPase subunit RPT6) (Proteasome 26S subunit ATPase 5) (Proteasome subunit p45) (p45/SUG) (mSUG1)
P62242	RS8_MOUSE	40S ribosomal protein S8
P62259	1433E_MOUSE	14-3-3 protein epsilon (14-3-3E)
P62264	RS14_MOUSE	40S ribosomal protein S14
P62267	RS23_MOUSE	40S ribosomal protein S23
P62270	RS18_MOUSE	40S ribosomal protein S18 (Ke-3) (Ke3)

P62281	RS11_MOUSE	40S ribosomal protein S11
P62301	RS13_MOUSE	40S ribosomal protein S13
P62305	RUXE_MOUSE	Small nuclear ribonucleoprotein E (snRNP-E) (Sm protein E) (Sm-E) (SmE)
P62315	SMD1_MOUSE	Small nuclear ribonucleoprotein Sm D1 (Sm-D1) (Sm-D autoantigen) (snRNP core protein D1)
P62320	SMD3_MOUSE	Small nuclear ribonucleoprotein Sm D3 (Sm-D3) (snRNP core protein D3)
P62331	ARF6_MOUSE	ADP-ribosylation factor 6
P62334	PRS10_MOUSE	26S proteasome regulatory subunit 10B (26S proteasome AAA-ATPase subunit RPT4) (Proteasome 26S subunit ATPase 6) (Proteasome subunit p42)
P62702	RS4X_MOUSE	40S ribosomal protein S4, X isoform
P62715	PP2AB_MOUSE	Serine/threonine-protein phosphatase 2A catalytic subunit beta isoform (PP2A-beta) (EC 3.1.3.16)
P62717	RL18A_MOUSE	60S ribosomal protein L18a
P62746	RHOB_MOUSE	Rho-related GTP-binding protein RhoB
P62754	RS6_MOUSE	40S ribosomal protein S6 (Phosphoprotein NP33)
P62774	MTPN_MOUSE	Myotrophin (Granule cell differentiation protein) (Protein V-1)
P62806	H4_MOUSE	Histone H4
P62814	VATB2_MOUSE	V-type proton ATPase subunit B, brain isoform (V-ATPase subunit B 2) (Endomembrane proton pump 58 kDa subunit) (Vacuolar proton pump subunit B 2)
P62821	RAB1A_MOUSE	Ras-related protein Rab-1A (YPT1-related protein)
P62830	RL23_MOUSE	60S ribosomal protein L23
P62849	RS24_MOUSE	40S ribosomal protein S24
P62852	RS25_MOUSE	40S ribosomal protein S25
P62855	RS26_MOUSE	40S ribosomal protein S26
P62858	RS28_MOUSE	40S ribosomal protein S28
P62874	GBB1_MOUSE	Guanine nucleotide-binding protein G(I)/G(S)/G(T) subunit beta-1 (Transducin beta chain 1)
P62880	GBB2_MOUSE	Guanine nucleotide-binding protein G(I)/G(S)/G(T) subunit beta-2 (G protein subunit beta-2) (Transducin beta chain 2)
P62889	RL30_MOUSE	60S ribosomal protein L30
P62900	RL31_MOUSE	60S ribosomal protein L31
P62908	RS3_MOUSE	40S ribosomal protein S3 (EC 4.2.99.18)
P62962	PROF1_MOUSE	Profilin-1 (Profilin I)
P63001	RAC1_MOUSE	Ras-related C3 botulinum toxin substrate 1 (EC 3.6.5.2) (p21-Rac1)
P63005	LIS1_MOUSE	Platelet-activating factor acetylhydrolase IB subunit alpha (Lissencephaly-1 protein) (LIS-1) (PAF acetylhydrolase 45 kDa subunit) (PAF-AH 45 kDa subunit) (PAF-AH alpha) (PAFAH alpha)
P63017	HSP7C_MOUSE	Heat shock cognate 71 kDa protein (Heat shock 70 kDa protein 8)

P63028	TCTP_MOUSE	Translationally-controlled tumor protein (TCTP) (21 kDa polypeptide) (p21) (p23)
P63037	DNJA1_MOUSE	DnaJ homolog subfamily A member 1 (DnaJ protein homolog 2) (Heat shock 40 kDa protein 4) (Heat shock protein J2) (HSJ-2)
P63038	CH60_MOUSE	60 kDa heat shock protein, mitochondrial (EC 5.6.1.7) (60 kDa chaperonin) (Chaperonin 60) (CPN60) (HSP-65) (Heat shock protein 60) (HSP-60) (Hsp60) (Mitochondrial matrix protein P1)
P63085	MK01_MOUSE	Mitogen-activated protein kinase 1 (MAP kinase 1) (MAPK 1) (EC 2.7.11.24) (ERT1) (Extracellular signal-regulated kinase 2) (ERK-2) (MAP kinase isoform p42) (p42-MAPK) (Mitogen-activated protein kinase 2) (MAP kinase 2) (MAPK 2)
P63094	GNAS2_MOUSE	Guanine nucleotide-binding protein G(s) subunit alpha isoforms short (Adenylate cyclase-stimulating G alpha protein)
P63101	1433Z_MOUSE	14-3-3 protein zeta/delta (Protein kinase C inhibitor protein 1) (KCIP-1) (SEZ-2)
P63242	IF5A1_MOUSE	Eukaryotic translation initiation factor 5A-1 (eIF-5A-1) (eIF-5A1) (Eukaryotic initiation factor 5A isoform 1) (eIF-5A) (eIF-4D)
P63276	RS17_MOUSE	40S ribosomal protein S17
P63323	RS12_MOUSE	40S ribosomal protein S12
P63330	PP2AA_MOUSE	Serine/threonine-protein phosphatase 2A catalytic subunit alpha isoform (PP2A-alpha) (EC 3.1.3.16)
P67871	CSK2B_MOUSE	Casein kinase II subunit beta (CK II beta) (Phosvitin)
P67984	RL22_MOUSE	60S ribosomal protein L22 (Heparin-binding protein HBp15)
P68037	UB2L3_MOUSE	Ubiquitin-conjugating enzyme E2 L3 (EC 2.3.2.23) (E2 ubiquitin-conjugating enzyme L3) (UbcM4) (Ubiquitin carrier protein L3) (Ubiquitin-protein ligase L3)
P68040	RACK1_MOUSE	Receptor of activated protein C kinase 1 (12-3) (Guanine nucleotide-binding protein subunit beta-2-like 1) (Receptor for activated C kinase) (Receptor of activated protein kinase C 1) (p205) [Cleaved into: Receptor of activated protein C kinase 1, N-terminally processed (Guanine nucleotide-binding protein subunit beta-2-like 1, N-terminally processed)]
P68134	ACTS_MOUSE	Actin, alpha skeletal muscle (Alpha-actin-1) [Cleaved into: Actin, alpha skeletal muscle, intermediate form]
P68181	KAPCB_MOUSE	cAMP-dependent protein kinase catalytic subunit beta (PKA C-beta) (EC 2.7.11.11)
P68254	1433T_MOUSE	14-3-3 protein theta (14-3-3 protein tau)
P68368	TBA4A_MOUSE	Tubulin alpha-4A chain (Alpha-tubulin 4) (Alpha-tubulin isotype M-alpha-4) (Tubulin alpha-4 chain)
P68369	TBA1A_MOUSE	Tubulin alpha-1A chain (Alpha-tubulin 1) (Alpha-tubulin isotype M-alpha-1) (Tubulin alpha-1 chain) [Cleaved into: Detyrosinated tubulin alpha-1A chain]

P68373	TBA1C_MOUSE	Tubulin alpha-1C chain (Alpha-tubulin 6) (Alpha-tubulin isotype M-alpha-6) (Tubulin alpha-6 chain) [Cleaved into: Detyrosinated tubulin alpha-1C chain]
P68404	KPCB_MOUSE	Protein kinase C beta type (PKC-B) (PKC-beta) (EC 2.7.11.13)
P68433	H31_MOUSE	Histone H3.1
P68510	1433F_MOUSE	14-3-3 protein eta
P70122	SBDS_MOUSE	Ribosome maturation protein SBDS (Protein 22A3) (Shwachman-Bodian-Diamond syndrome protein homolog)
P70168	IMB1_MOUSE	Importin subunit beta-1 (Karyopherin subunit beta-1) (Nuclear factor p97) (Pore targeting complex 97 kDa subunit) (PTAC97) (SCG)
P70195	PSB7_MOUSE	Proteasome subunit beta type-7 (EC 3.4.25.1) (Macropain chain Z) (Multicatalytic endopeptidase complex chain Z) (Proteasome subunit Z)
P70202	LXN_MOUSE	Latexin (Endogenous carboxypeptidase inhibitor) (ECI) (Tissue carboxypeptidase inhibitor) (TCI)
P70248	MYO1F_MOUSE	Unconventional myosin-If
P70261	PALD_MOUSE	Paladin
P70288	HDAC2_MOUSE	Histone deacetylase 2 (HD2) (EC 3.5.1.98) (YY1 transcription factor-binding protein)
P70296	PEBP1_MOUSE	Phosphatidylethanolamine-binding protein 1 (PEBP-1) (HCNPpp) [Cleaved into: Hippocampal cholinergic neurostimulating peptide (HCNP)]
P70349	HINT1_MOUSE	Histidine triad nucleotide-binding protein 1 (EC 3.-.-.-) (Adenosine 5'-monophosphoramidase) (Protein kinase C inhibitor 1) (Protein kinase C-interacting protein 1) (PKCI-1)
P70460	VASP_MOUSE	Vasodilator-stimulated phosphoprotein (VASP)
P70670	NACAM_MOUSE	Nascent polypeptide-associated complex subunit alpha, muscle-specific form (Alpha-NAC, muscle-specific form) (skNAC)
P70677	CASP3_MOUSE	Caspase-3 (CASP-3) (EC 3.4.22.56) (Apopain) (Cysteine protease CPP32) (CPP-32) (LICE) (Protein Yama) (SREBP cleavage activity 1) (SCA-1) [Cleaved into: Caspase-3 subunit p17; Caspase-3 subunit p12]
P70698	PYRG1_MOUSE	CTP synthase 1 (EC 6.3.4.2) (CTP synthetase 1) (UTP--ammonia ligase 1)
P80313	TCPH_MOUSE	T-complex protein 1 subunit eta (TCP-1-eta) (CCT-eta)
P80314	TCPB_MOUSE	T-complex protein 1 subunit beta (TCP-1-beta) (CCT-beta)
P80315	TCPD_MOUSE	T-complex protein 1 subunit delta (TCP-1-delta) (A45) (CCT-delta)
P80316	TCPE_MOUSE	T-complex protein 1 subunit epsilon (TCP-1-epsilon) (CCT-epsilon)
P80317	TCPZ_MOUSE	T-complex protein 1 subunit zeta (TCP-1-zeta) (CCT-zeta-1)
P80318	TCPG_MOUSE	T-complex protein 1 subunit gamma (TCP-1-gamma) (CCT-gamma) (Matricin) (mTRiC-P5)

P84078	ARF1_MOUSE	ADP-ribosylation factor 1
P84084	ARF5_MOUSE	ADP-ribosylation factor 5
P84091	AP2M1_MOUSE	AP-2 complex subunit mu (AP-2 mu chain) (Adaptor protein complex AP-2 subunit mu) (Adaptor-related protein complex 2 subunit mu) (Clathrin assembly protein complex 2 mu medium chain) (Clathrin coat assembly protein AP50) (Clathrin coat-associated protein AP50) (Mu2-adaptin) (Plasma membrane adaptor AP-2 50 kDa protein)
P84096	RHOG_MOUSE	Rho-related GTP-binding protein RhoG (Sid 10750)
P84099	RL19_MOUSE	60S ribosomal protein L19
P84104	SRSF3_MOUSE	Serine/arginine-rich splicing factor 3 (Pre-mRNA-splicing factor SRP20) (Protein X16) (Splicing factor, arginine/serine-rich 3)
P84228	H32_MOUSE	Histone H3.2
P85094	ISC2A_MOUSE	Isochorismatase domain-containing protein 2A
P97315	CSRP1_MOUSE	Cysteine and glycine-rich protein 1 (Cysteine-rich protein 1) (CRP) (CRP1)
P97351	RS3A_MOUSE	40S ribosomal protein S3a (Protein TU-11)
P97370	AT1B3_MOUSE	Sodium/potassium-transporting ATPase subunit beta-3 (Sodium/potassium-dependent ATPase subunit beta-3) (ATPB-3) (CD antigen CD298)
P97371	PSME1_MOUSE	Proteasome activator complex subunit 1 (11S regulator complex subunit alpha) (REG-alpha) (Activator of multicatalytic protease subunit 1) (Proteasome activator 28 subunit alpha) (PA28a) (PA28alpha)
P97372	PSME2_MOUSE	Proteasome activator complex subunit 2 (11S regulator complex subunit beta) (REG-beta) (Activator of multicatalytic protease subunit 2) (Proteasome activator 28 subunit beta) (PA28b) (PA28beta)
P97384	ANX11_MOUSE	Annexin A11 (Annexin XI) (Annexin-11) (Calcyclin-associated annexin 50) (CAP-50)
P97429	ANXA4_MOUSE	Annexin A4 (Annexin IV) (Annexin-4)
P97470	PP4C_MOUSE	Serine/threonine-protein phosphatase 4 catalytic subunit (PP4C) (Pp4) (EC 3.1.3.16) (Protein phosphatase X) (PP-X)
P97484	LIRB3_MOUSE	Leukocyte immunoglobulin-like receptor subfamily B member 3 (LIR-3) (Leukocyte immunoglobulin-like receptor 3) (Cell-surface glycoprotein p91) (Paired immunoglobulin-like receptor B) (PIR-B)
P97492	RGS14_MOUSE	Regulator of G-protein signaling 14 (RGS14) (RAP1/RAP2-interacting protein) (RPIP1)
P97499	TEP1_MOUSE	Telomerase protein component 1 (Telomerase-associated protein 1) (Telomerase protein 1) (p240) (p80 telomerase homolog)
P99024	TBB5_MOUSE	Tubulin beta-5 chain
P99026	PSB4_MOUSE	Proteasome subunit beta type-4 (EC 3.4.25.1) (Low molecular mass protein 3) (Macropain beta chain)

		(Multicatalytic endopeptidase complex beta chain) (Proteasome beta chain) (Proteasome chain 3)
P99029	PRDX5_MOUSE	Peroxiredoxin-5, mitochondrial (EC 1.11.1.15) (Antioxidant enzyme B166) (AOEB166) (Liver tissue 2D-page spot 2D-0014IV) (PLP) (Peroxiredoxin V) (Prx-V) (Peroxisomal antioxidant enzyme) (Thioredoxin peroxidase PMP20)
Q00519	XDH_MOUSE	Xanthine dehydrogenase/oxidase [Includes: Xanthine dehydrogenase (XD) (EC 1.17.1.4); Xanthine oxidase (XO) (EC 1.17.3.2) (Xanthine oxidoreductase) (XOR)]
Q00651	ITA4_MOUSE	Integrin alpha-4 (CD49 antigen-like family member D) (Integrin alpha-IV) (Lymphocyte Peyer patch adhesion molecules subunit alpha) (LPAM subunit alpha) (VLA-4 subunit alpha) (CD antigen CD49d)
Q00PI9	HNRL2_MOUSE	Heterogeneous nuclear ribonucleoprotein U-like protein 2 (MLF1-associated nuclear protein)
Q01405	SC23A_MOUSE	Protein transport protein Sec23A (SEC23-related protein A)
Q01730	RSU1_MOUSE	Ras suppressor protein 1 (RSP-1) (Rsu-1)
Q02053	UBA1_MOUSE	Ubiquitin-like modifier-activating enzyme 1 (EC 6.2.1.45) (Ubiquitin-activating enzyme E1) (Ubiquitin-activating enzyme E1 X) (Ubiquitin-like modifier-activating enzyme 1 X)
Q02566	MYH6_MOUSE	Myosin-6 (Myosin heavy chain 6) (Myosin heavy chain, cardiac muscle alpha isoform) (MyHC-alpha)
Q04447	KCRB_MOUSE	Creatine kinase B-type (EC 2.7.3.2) (B-CK) (Creatine kinase B chain) (Creatine phosphokinase B-type) (CPK-B)
Q04736	YES_MOUSE	Tyrosine-protein kinase Yes (EC 2.7.10.2) (Proto-oncogene c-Yes) (p61-Yes)
Q05144	RAC2_MOUSE	Ras-related C3 botulinum toxin substrate 2 (Protein EN-7) (p21-Rac2)
Q05816	FABP5_MOUSE	Fatty acid-binding protein 5 (Epidermal-type fatty acid-binding protein) (E-FABP) (Fatty acid-binding protein, epidermal) (Keratinocyte lipid-binding protein) (Psoriasis-associated fatty acid-binding protein homolog) (PA-FABP)
Q05D44	IF2P_MOUSE	Eukaryotic translation initiation factor 5B (eIF-5B) (EC 3.6.5.3) (Translation initiation factor IF-2)
Q06138	CAB39_MOUSE	Calcium-binding protein 39 (MO25alpha) (Protein Mo25)
Q08481	PECA1_MOUSE	Platelet endothelial cell adhesion molecule (PECAM-1) (CD antigen CD31)
Q08509	EPS8_MOUSE	Epidermal growth factor receptor kinase substrate 8
Q08943	SSRP1_MOUSE	FACT complex subunit SSRP1 (Facilitates chromatin transcription complex subunit SSRP1) (Recombination signal sequence recognition protein 1) (Structure-specific recognition protein 1) (T160)
Q09014	NCF1_MOUSE	Neutrophil cytosol factor 1 (NCF-1) (47 kDa neutrophil oxidase factor) (NCF-47K) (Neutrophil NADPH oxidase factor 1) (p47-phox)

Q09200	B4GN1_MOUSE	Beta-1,4 N-acetylgalactosaminyltransferase 1 (EC 2.4.1.92) ((N-acetylneuraminyl)-galactosylglucosylceramide) (GM2/GD2 synthase) (GalNAc-T)
Q0EEE2	PTHD3_MOUSE	Patched domain-containing protein 3 (RND-type protein RNDEu-3)
Q10470	MGAT3_MOUSE	Beta-1,4-mannosyl-glycoprotein 4-beta-N-acetylglucosaminyltransferase (EC 2.4.1.144) (N-glycosyl-oligosaccharide-glycoprotein N-acetylglucosaminyltransferase III) (GNT-III) (GlcNAc-T III) (N-acetylglucosaminyltransferase III)
Q11011	PSA_MOUSE	Puromycin-sensitive aminopeptidase (PSA) (EC 3.4.11.14) (Cytosol alanyl aminopeptidase) (AAP-S)
Q149F3	ERF3B_MOUSE	Eukaryotic peptide chain release factor GTP-binding subunit ERF3B (Eukaryotic peptide chain release factor subunit 3b) (eRF3b) (G1 to S phase transition protein 2 homolog)
Q1EG27	MYO3B_MOUSE	Myosin-IIIb (EC 2.7.11.1)
Q2XU92	ACBG2_MOUSE	Long-chain-fatty-acid--CoA ligase ACSBG2 (EC 6.2.1.3) (Acyl-CoA synthetase bubblegum family member 2) (Arachidonate--CoA ligase ACSBG2) (EC 6.2.1.15) (Bubblegum-related protein)
Q32NZ6	TMC5_MOUSE	Transmembrane channel-like protein 5
Q3TB82	PKHF1_MOUSE	Pleckstrin homology domain-containing family F member 1 (PH domain-containing family F member 1) (Lysosome-associated apoptosis-inducing protein containing PH and FYVE domains)
Q3TBD2	HMHA1_MOUSE	Rho GTPase-activating protein 45 (Minor histocompatibility protein HA-1)
Q3TBT3	STING_MOUSE	Stimulator of interferon genes protein (mSTING) (Endoplasmic reticulum interferon stimulator) (ERIS) (Mediator of IRF3 activation) (MMITA) (Transmembrane protein 173)
Q3TIU4	PDE12_MOUSE	2',5'-phosphodiesterase 12 (2'-PDE) (2-PDE) (EC 3.1.4.-) (Mitochondrial deadenylase) (EC 3.1.13.4)
Q3TLI0	TPC10_MOUSE	Trafficking protein particle complex subunit 10 (Trafficking protein particle complex subunit TMEM1) (Transport protein particle subunit TMEM1) (TRAPP subunit TMEM1)
Q3TQQ9	CA112_MOUSE	Uncharacterized protein C1orf112 homolog
Q3TRM8	HXK3_MOUSE	Hexokinase-3 (EC 2.7.1.1) (Hexokinase type III) (HK III) (Hexokinase-C)
Q3TW96	UAP1L_MOUSE	UDP-N-acetylhexosamine pyrophosphorylase-like protein 1 (EC 2.7.7.-)
Q3TXS7	PSMD1_MOUSE	26S proteasome non-ATPase regulatory subunit 1 (26S proteasome regulatory subunit RPN2) (26S proteasome regulatory subunit S1)
Q3U1Z5	GPSM3_MOUSE	G-protein-signaling modulator 3

Q3U2S8	HVCN1_MOUSE	Voltage-gated hydrogen channel 1 (Hydrogen voltage-gated channel 1) (HV1) (Voltage sensor domain-only protein) (mVSOP)
Q3U7R1	ESYT1_MOUSE	Extended synaptotagmin-1 (E-Syt1) (Membrane-bound C2 domain-containing protein)
Q3U898	CSN9_MOUSE	COP9 signalosome complex subunit 9
Q3UDE2	TTL12_MOUSE	Tubulin--tyrosine ligase-like protein 12 (Inactive tubulin--tyrosine ligase-like protein 12)
Q3UH60	DIP2B_MOUSE	Disco-interacting protein 2 homolog B (DIP2 homolog B)
Q3UH93	PLXD1_MOUSE	Plexin-D1
Q3UHU5	MTCL1_MOUSE	Microtubule cross-linking factor 1 (Coiled-coil domain-containing protein 165) (PAR-1-interacting protein) (SOGA family member 2)
Q3UIR3	DTX3L_MOUSE	E3 ubiquitin-protein ligase DTX3L (EC 2.3.2.27) (Protein deltex-3-like) (RING-type E3 ubiquitin transferase DTX3L)
Q3UMY5	EMAL4_MOUSE	Echinoderm microtubule-associated protein-like 4 (EMAP-4)
Q3UPL0	SC31A_MOUSE	Protein transport protein Sec31A (SEC31-like protein 1) (SEC31-related protein A)
Q3UQ44	IQGA2_MOUSE	Ras GTPase-activating-like protein IQGAP2
Q3UVV9	VWA3A_MOUSE	von Willebrand factor A domain-containing protein 3A
Q3UW53	NIBA1_MOUSE	Protein Niban 1 (Protein FAM129A) (Protein Niban)
Q3UX10	TBAL3_MOUSE	Tubulin alpha chain-like 3
Q3UY96	CFA74_MOUSE	Cilia- and flagella-associated protein 74
Q3V1L4	5NTC_MOUSE	Cytosolic purine 5'-nucleotidase (EC 3.1.3.5) (Cytosolic 5'-nucleotidase II)
Q3V3I2	GNAT3_MOUSE	Guanine nucleotide-binding protein G(t) subunit alpha-3 (Gustducin alpha-3 chain)
Q4KML4	ABRAL_MOUSE	Costars family protein ABRACL (ABRA C-terminal-like protein)
Q4QRL3	CC88B_MOUSE	Coiled-coil domain-containing protein 88B (Gipie) (Hook-related protein 3) (HkRP3)
Q4ZJN1	C1QT9_MOUSE	Complement C1q and tumor necrosis factor-related protein 9
Q501J6	DDX17_MOUSE	Probable ATP-dependent RNA helicase DDX17 (EC 3.6.4.13) (DEAD box protein 17)
Q571I9	A16A1_MOUSE	Aldehyde dehydrogenase family 16 member A1
Q5NCX5	NEUL4_MOUSE	Neuralized-like protein 4
Q5QNQ9	CORA1_MOUSE	Collagen alpha-1(XXVII) chain
Q5SS00	ZDBF2_MOUSE	DBF4-type zinc finger-containing protein 2 homolog
Q5SUA5	MYO1G_MOUSE	Unconventional myosin-Ig
Q5SUF2	LC7L3_MOUSE	Luc7-like protein 3 (Cisplatin resistance-associated-overexpressed protein)
Q5SUR0	PUR4_MOUSE	Phosphoribosylformylglycinamidine synthase (FGAM synthase) (FGAMS) (EC 6.3.5.3) (Formylglycinamide ribonucleotide amidotransferase) (FGAR amidotransferase)

		(FGAR-AT) (Formylglycinamide ribotide amidotransferase)
Q5SWU9	ACACA_MOUSE	Acetyl-CoA carboxylase 1 (ACC1) (EC 6.4.1.2) (ACC-alpha) (Acetyl-CoA carboxylase 265)
Q60597	ODO1_MOUSE	2-oxoglutarate dehydrogenase, mitochondrial (EC 1.2.4.2) (2-oxoglutarate dehydrogenase complex component E1) (OGDC-E1) (Alpha-ketoglutarate dehydrogenase)
Q60605	MYL6_MOUSE	Myosin light polypeptide 6 (17 kDa myosin light chain) (LC17) (Myosin light chain 3) (MLC-3) (Myosin light chain alkali 3) (Myosin light chain A3) (Smooth muscle and nonmuscle myosin light chain alkali 6)
Q60631	GRB2_MOUSE	Growth factor receptor-bound protein 2 (Adapter protein GRB2) (SH2/SH3 adapter GRB2)
Q60668	HNRPD_MOUSE	Heterogeneous nuclear ribonucleoprotein D0 (hnRNP D0) (AU-rich element RNA-binding protein 1)
Q60692	PSB6_MOUSE	Proteasome subunit beta type-6 (EC 3.4.25.1) (Low molecular mass protein 19) (Macropain delta chain) (Multicatalytic endopeptidase complex delta chain) (Proteasome delta chain) (Proteasome subunit Y)
Q60710	SAMH1_MOUSE	Deoxynucleoside triphosphate triphosphohydrolase SAMHD1 (dNTPase) (EC 3.1.5.-) (Interferon-gamma-inducible protein Mg11) (SAM domain and HD domain-containing protein 1) (mSAMHD1)
Q60739	BAG1_MOUSE	BAG family molecular chaperone regulator 1 (BAG-1) (Bcl-2-associated athanogene 1)
Q60767	LY75_MOUSE	Lymphocyte antigen 75 (Ly-75) (DEC-205) (CD antigen CD205)
Q60770	STXB3_MOUSE	Syntaxin-binding protein 3 (MUNC-18-3) (Mammalian homolog of Unc-18c) (Munc-18c) (Protein unc-18 homolog 3) (Unc18-3) (Protein unc-18 homolog C) (Unc-18C)
Q60787	LCP2_MOUSE	Lymphocyte cytosolic protein 2 (SH2 domain-containing leukocyte protein of 76 kDa) (SLP-76 tyrosine phosphoprotein) (SLP76)
Q60875	ARHG2_MOUSE	Rho guanine nucleotide exchange factor 2 (Guanine nucleotide exchange factor H1) (GEF-H1) (LBC'S first cousin) (Lymphoid blast crisis-like 1) (Oncogene LFC) (Rhobin)
Q60930	VDAC2_MOUSE	Voltage-dependent anion-selective channel protein 2 (VDAC-2) (mVDAC2) (Outer mitochondrial membrane protein porin 2) (Voltage-dependent anion-selective channel protein 6) (VDAC-6) (mVDAC6)
Q60932	VDAC1_MOUSE	Voltage-dependent anion-selective channel protein 1 (VDAC-1) (mVDAC1) (Outer mitochondrial membrane protein porin 1) (Plasmalemmal porin) (Voltage-dependent anion-selective channel protein 5) (VDAC-5) (mVDAC5)
Q60972	RBBP4_MOUSE	Histone-binding protein RBBP4 (Chromatin assembly factor 1 subunit C) (CAF-1 subunit C) (Chromatin assembly factor I p48 subunit) (CAF-I 48 kDa subunit) (CAF-I p48) (Nucleosome-remodeling factor subunit

		RBAP48) (Retinoblastoma-binding protein 4) (RBBP-4) (Retinoblastoma-binding protein p48)
Q60973	RBBP7_MOUSE	Histone-binding protein RBBP7 (Histone acetyltransferase type B subunit 2) (Nucleosome-remodeling factor subunit RBAP46) (Retinoblastoma-binding protein 7) (RBBP-7) (Retinoblastoma-binding protein p46)
Q61035	SYHC_MOUSE	Histidine--tRNA ligase, cytoplasmic (EC 6.1.1.21) (Histidyl-tRNA synthetase) (HisRS)
Q61081	CDC37_MOUSE	Hsp90 co-chaperone Cdc37 (Hsp90 chaperone protein kinase-targeting subunit) (p50Cdc37) [Cleaved into: Hsp90 co-chaperone Cdc37, N-terminally processed]
Q61151	2A5E_MOUSE	Serine/threonine-protein phosphatase 2A 56 kDa regulatory subunit epsilon isoform (PP2A B subunit isoform B'-epsilon) (PP2A B subunit isoform B56-epsilon) (PP2A B subunit isoform PR61-epsilon) (PP2A B subunit isoform R5-epsilon)
Q61171	PRDX2_MOUSE	Peroxiredoxin-2 (EC 1.11.1.15) (Thiol-specific antioxidant protein) (TSA) (Thioredoxin peroxidase 1) (Thioredoxin-dependent peroxide reductase 1)
Q61176	ARGI1_MOUSE	Arginase-1 (EC 3.5.3.1) (Liver-type arginase) (Type I arginase)
Q61205	PA1B3_MOUSE	Platelet-activating factor acetylhydrolase IB subunit gamma (EC 3.1.1.47) (PAF acetylhydrolase 29 kDa subunit) (PAF-AH 29 kDa subunit) (PAF-AH subunit gamma) (PAFAH subunit gamma)
Q61206	PA1B2_MOUSE	Platelet-activating factor acetylhydrolase IB subunit beta (EC 3.1.1.47) (PAF acetylhydrolase 30 kDa subunit) (PAF-AH 30 kDa subunit) (PAF-AH subunit beta) (PAFAH subunit beta)
Q61210	ARHG1_MOUSE	Rho guanine nucleotide exchange factor 1 (Lbc's second cousin) (Lymphoid blast crisis-like 2)
Q61239	FNTA_MOUSE	Protein farnesyltransferase/geranylgeranyltransferase type-1 subunit alpha (EC 2.5.1.58) (EC 2.5.1.59) (CAAX farnesyltransferase subunit alpha) (FTase-alpha) (Ras proteins prenyltransferase subunit alpha) (Type I protein geranyl-geranyltransferase subunit alpha) (GGTase-I-alpha)
Q61316	HSP74_MOUSE	Heat shock 70 kDa protein 4 (Heat shock 70-related protein APG-2)
Q61390	TCPW_MOUSE	T-complex protein 1 subunit zeta-2 (TCP-1-zeta-2) (CCT-zeta-2) (Cctz-2)
Q61553	FSCN1_MOUSE	Fascin (Singed-like protein)
Q61598	GDIB_MOUSE	Rab GDP dissociation inhibitor beta (Rab GDI beta) (GDI-3) (Guanosine diphosphate dissociation inhibitor 2) (GDI-2)
Q61599	GDIR2_MOUSE	Rho GDP-dissociation inhibitor 2 (Rho GDI 2) (D4) (Rho-GDI beta)
Q61699	HS105_MOUSE	Heat shock protein 105 kDa (42 degrees C-HSP) (Heat shock 110 kDa protein) (Heat shock-related 100 kDa protein E7I) (HSP-E7I)

Q61735	CD47_MOUSE	Leukocyte surface antigen CD47 (Integrin-associated protein) (IAP) (CD antigen CD47)
Q61768	KINH_MOUSE	Kinesin-1 heavy chain (Conventional kinesin heavy chain) (Ubiquitous kinesin heavy chain) (UKHC)
Q61792	LASP1_MOUSE	LIM and SH3 domain protein 1 (LASP-1) (Metastatic lymph node gene 50 protein) (MLN 50)
Q61838	PZP_MOUSE	Pregnancy zone protein (Alpha-2-macroglobulin) (Alpha-2-M) [Cleaved into: Alpha-2-macroglobulin 165 kDa subunit; Alpha-2-macroglobulin 35 kDa subunit]
Q61881	MCM7_MOUSE	DNA replication licensing factor MCM7 (EC 3.6.4.12) (CDC47 homolog)
Q61937	NPM_MOUSE	Nucleophosmin (NPM) (Nucleolar phosphoprotein B23) (Nucleolar protein NO38) (Numatrin)
Q61990	PCBP2_MOUSE	Poly(rC)-binding protein 2 (Alpha-CP2) (CTBP) (CBP) (Putative heterogeneous nuclear ribonucleoprotein X) (hnRNP X)
Q62009	POSTN_MOUSE	Periostin (PN) (Osteoblast-specific factor 2) (OSF-2)
Q62136	PTN21_MOUSE	Tyrosine-protein phosphatase non-receptor type 21 (EC 3.1.3.48) (Protein-tyrosine phosphatase PTP-RL10)
Q62148	AL1A2_MOUSE	Retinal dehydrogenase 2 (RALDH 2) (RalDH2) (EC 1.2.1.36) (Aldehyde dehydrogenase family 1 member A2) (Retinaldehyde-specific dehydrogenase type 2) (RALDH(II))
Q62186	SSRD_MOUSE	Translocon-associated protein subunit delta (TRAP-delta) (Signal sequence receptor subunit delta) (SSR-delta)
Q62261	SPTB2_MOUSE	Spectrin beta chain, non-erythrocytic 1 (Beta-II spectrin) (Embryonic liver fodrin) (Fodrin beta chain)
Q62348	TSN_MOUSE	Translin (EC 3.1.-.-) (Component 3 of promoter of RISC) (C3PO) (Testis/brain RNA-binding protein) (TB-RBP)
Q62351	TFR1_MOUSE	Transferrin receptor protein 1 (TR) (TfR) (TfR1) (Trfr) (CD antigen CD71)
Q62422	OSTF1_MOUSE	Osteoclast-stimulating factor 1 (SH3 domain protein 3)
Q62426	CYTB_MOUSE	Cystatin-B (Stefin-B)
Q62448	IF4G2_MOUSE	Eukaryotic translation initiation factor 4 gamma 2 (eIF-4-gamma 2) (eIF-4G 2) (eIF4G 2) (Novel APOBEC-1 target 1) (Translation repressor NAT1) (p97)
Q62465	VAT1_MOUSE	Synaptic vesicle membrane protein VAT-1 homolog (EC 1.-.-.-)
Q63844	MK03_MOUSE	Mitogen-activated protein kinase 3 (MAP kinase 3) (MAPK 3) (EC 2.7.11.24) (ERT2) (Extracellular signal-regulated kinase 1) (ERK-1) (Insulin-stimulated MAP2 kinase) (MAP kinase isoform p44) (p44-MAPK) (MNK1) (Microtubule-associated protein 2 kinase) (p44-ERK1)
Q64127	TIF1A_MOUSE	Transcription intermediary factor 1-alpha (TIF1-alpha) (EC 2.3.2.27) (E3 ubiquitin-protein ligase Trim24) (RING-type E3 ubiquitin transferase TIF1-alpha) (Tripartite motif-containing protein 24)
Q64253	LY6E_MOUSE	Lymphocyte antigen 6E (Ly-6E) (Stem cell antigen 2) (Thymic shared antigen 1) (TSA-1)

Q64277	BST1_MOUSE	ADP-ribosyl cyclase/cyclic ADP-ribose hydrolase 2 (EC 3.2.2.6) (ADP-ribosyl cyclase 2) (Antigen BP3) (BP-3 alloantigen) (Bone marrow stromal antigen 1) (BST-1) (Cyclic ADP-ribose hydrolase 2) (cADPr hydrolase 2) (Leukocyte antigen 65) (Ly-65) (CD antigen CD157)
Q64310	SURF4_MOUSE	Surfeit locus protein 4
Q64337	SQSTM_MOUSE	Sequestosome-1 (STONE14) (Ubiquitin-binding protein p62)
Q64378	FKBP5_MOUSE	Peptidyl-prolyl cis-trans isomerase FKBP5 (PPIase FKBP5) (EC 5.2.1.8) (51 kDa FK506-binding protein) (51 kDa FKBP) (FKBP-51) (FK506-binding protein 5) (FKBP-5) (Rotamase)
Q64436	ATP4A_MOUSE	Potassium-transporting ATPase alpha chain 1 (EC 7.2.2.19) (Gastric H(+)/K(+) ATPase subunit alpha) (Proton pump)
Q64516	GLPK_MOUSE	Glycerol kinase (GK) (Glycerokinase) (EC 2.7.1.30) (ATP:glycerol 3-phosphotransferase)
Q64518	AT2A3_MOUSE	Sarcoplasmic/endoplasmic reticulum calcium ATPase 3 (SERCA3) (SR Ca(2+)-ATPase 3) (EC 7.2.2.10) (Calcium pump 3)
Q64522	H2A2B_MOUSE	Histone H2A type 2-B (H2a-613A)
Q64674	SPEE_MOUSE	Spermidine synthase (SPDSY) (EC 2.5.1.16) (Putrescine aminopropyltransferase)
Q64727	VINC_MOUSE	Vinculin (Metavinculin)
Q67E05	BPI_MOUSE	Bactericidal permeability-increasing protein (BPI)
Q68FD5	CLH1_MOUSE	Clathrin heavy chain 1
Q68FH4	GALK2_MOUSE	N-acetylgalactosamine kinase (EC 2.7.1.157) (GalNAc kinase) (Galactokinase 2)
Q68FL6	SYMC_MOUSE	Methionine--tRNA ligase, cytoplasmic (EC 6.1.1.10) (Methionyl-tRNA synthetase) (MetRS)
Q69ZN7	MYOF_MOUSE	Myoferlin (Fer-1-like protein 3)
Q6A0D4	RFTN1_MOUSE	Raftlin (Raft-linking protein)
Q6DFW4	NOP58_MOUSE	Nucleolar protein 58 (MSSP) (Nucleolar protein 5) (SIK-similar protein)
Q6GQT1	A2MG_MOUSE	Alpha-2-macroglobulin-P (Alpha-2-macroglobulin)
Q6IRU2	TPM4_MOUSE	Tropomyosin alpha-4 chain (Tropomyosin-4)
Q6NVF4	HELB_MOUSE	DNA helicase B (EC 3.6.4.12)
Q6NXH9	K2C73_MOUSE	Keratin, type II cytoskeletal 73 (Cytokeratin-73) (CK-73) (Keratin-73) (K73) (Type II inner root sheath-specific keratin-K6irs3) (Type-II keratin Kb36)
Q6NZJ6	IF4G1_MOUSE	Eukaryotic translation initiation factor 4 gamma 1 (eIF-4-gamma 1) (eIF-4G 1) (eIF-4G1)
Q6P069	SORCN_MOUSE	Sorcin
Q6P1B1	XPP1_MOUSE	Xaa-Pro aminopeptidase 1 (EC 3.4.11.9) (Aminoacylproline aminopeptidase) (Cytosolic aminopeptidase P) (Soluble aminopeptidase P) (sAmp) (X-Pro aminopeptidase 1) (X-prolyl aminopeptidase 1, soluble)

Q6P4T2	U520_MOUSE	U5 small nuclear ribonucleoprotein 200 kDa helicase (EC 3.6.4.13) (BRR2 homolog) (U5 snRNP-specific 200 kDa protein) (U5-200KD)
Q6P5E4	UGGG1_MOUSE	UDP-glucose:glycoprotein glucosyltransferase 1 (UGT1) (EC 2.4.1.-) (UDP--Glc:glycoprotein glucosyltransferase) (UDP-glucose ceramide glucosyltransferase-like 1)
Q6P5F9	XPO1_MOUSE	Exportin-1 (Exp1) (Chromosome region maintenance 1 protein homolog)
Q6P8X1	SNX6_MOUSE	Sorting nexin-6 [Cleaved into: Sorting nexin-6, N-terminally processed]
Q6PB66	LPPRC_MOUSE	Leucine-rich PPR motif-containing protein, mitochondrial (130 kDa leucine-rich protein) (LRP 130) (mLRP130)
Q6PD03	2A5A_MOUSE	Serine/threonine-protein phosphatase 2A 56 kDa regulatory subunit alpha isoform (PP2A B subunit isoform B'-alpha) (PP2A B subunit isoform B56-alpha) (PP2A B subunit isoform PR61-alpha) (PR61alpha) (PP2A B subunit isoform R5-alpha)
Q6PDM2	SRSF1_MOUSE	Serine/arginine-rich splicing factor 1 (ASF/SF2) (Pre-mRNA-splicing factor SRp30a) (Splicing factor, arginine/serine-rich 1)
Q6PEB6	PHOCN_MOUSE	MOB-like protein phocein (Class II mMOB1) (Mob1 homolog 3) (Mob3) (Mps one binder kinase activator-like 3) (Preimplantation protein 3)
Q6PGF7	EXOC8_MOUSE	Exocyst complex component 8 (Exocyst complex 84 kDa subunit)
Q6PHN9	RAB35_MOUSE	Ras-related protein Rab-35
Q6PHZ2	KCC2D_MOUSE	Calcium/calmodulin-dependent protein kinase type II subunit delta (CaM kinase II subunit delta) (CaMK-II subunit delta) (EC 2.7.11.17)
Q6Q899	DDX58_MOUSE	Antiviral innate immune response receptor RIG-I (DEAD box protein 58) (Probable ATP-dependent RNA helicase DDX58) (EC 3.6.4.13) (RIG-I-like receptor 1) (RLR-1) (Retinoic acid-inducible gene 1 protein) (RIG-1) (Retinoic acid-inducible gene I protein) (RIG-I)
Q6ZQ06	CE162_MOUSE	Centrosomal protein of 162 kDa (Cep162) (Protein QN1 homolog)
Q6ZQ38	CAND1_MOUSE	Cullin-associated NEDD8-dissociated protein 1 (Cullin-associated and neddylation-dissociated protein 1) (p120 CAND1)
Q6ZQA0	NBEL2_MOUSE	Neurobeachin-like protein 2
Q6ZWN5	RS9_MOUSE	40S ribosomal protein S9
Q6ZWR4	2ABB_MOUSE	Serine/threonine-protein phosphatase 2A 55 kDa regulatory subunit B beta isoform (PP2A subunit B isoform B55-beta) (PP2A subunit B isoform PR55-beta) (PP2A subunit B isoform R2-beta) (PP2A subunit B isoform beta)
Q6ZWV3	RL10_MOUSE	60S ribosomal protein L10 (Protein QM homolog) (Ribosomal protein L10)

Q6ZWX6	IF2A_MOUSE	Eukaryotic translation initiation factor 2 subunit 1 (Eukaryotic translation initiation factor 2 subunit alpha) (eIF-2-alpha) (eIF-2A) (eIF-2alpha)
Q70KF4	CMYA5_MOUSE	Cardiomyopathy-associated protein 5 (Heart and skeletal muscle-specific and sprouty domain-containing) (Myospryn) (Stretch-response protein 553) (Stretch-responsive fibronectin protein type 3) (TRIM-like protein)
Q76MZ3	2AAA_MOUSE	Serine/threonine-protein phosphatase 2A 65 kDa regulatory subunit A alpha isoform (PP2A subunit A isoform PR65-alpha) (PP2A subunit A isoform R1-alpha)
Q78ZA7	NP1L4_MOUSE	Nucleosome assembly protein 1-like 4
Q7TMB8	CYFP1_MOUSE	Cytoplasmic FMR1-interacting protein 1 (Specifically Rac1-associated protein 1) (Sra-1)
Q7TMK9	HNRPQ_MOUSE	Heterogeneous nuclear ribonucleoprotein Q (hnRNP Q) (Glycine- and tyrosine-rich RNA-binding protein) (GRY-RBP) (NS1-associated protein 1) (Synaptotagmin-binding, cytoplasmic RNA-interacting protein) (pp68)
Q7TMM9	TBB2A_MOUSE	Tubulin beta-2A chain
Q7TMY8	HUWE1_MOUSE	E3 ubiquitin-protein ligase HUWE1 (EC 2.3.2.26) (E3Histone) (HECT, UBA and WWE domain-containing protein 1) (HECT-type E3 ubiquitin transferase HUWE1) (Upstream regulatory element-binding protein 1) (URE-B1) (URE-binding protein 1)
Q7TPR4	ACTN1_MOUSE	Alpha-actinin-1 (Alpha-actinin cytoskeletal isoform) (F-actin cross-linking protein) (Non-muscle alpha-actinin-1)
Q7TQ65	TMC4_MOUSE	Transmembrane channel-like protein 4
Q7TQH0	ATX2L_MOUSE	Ataxin-2-like protein
Q7TQI3	OTUB1_MOUSE	Ubiquitin thioesterase OTUB1 (EC 3.4.19.12) (Deubiquitinating enzyme OTUB1) (OTU domain-containing ubiquitin aldehyde-binding protein 1) (Otubain-1) (Ubiquitin-specific-processing protease OTUB1)
Q80SU7	GVIN1_MOUSE	Interferon-induced very large GTPase 1 (Very large-inducible GTPase-1) (VLIG-1)
Q80TL7	MON2_MOUSE	Protein MON2 homolog (Protein SF21)
Q80UG5	SEPT9_MOUSE	Septin-9 (SL3-3 integration site 1 protein)
Q80VF6	F181B_MOUSE	Protein FAM181B
Q80VQ0	AL3B1_MOUSE	Aldehyde dehydrogenase family 3 member B1 (EC 1.2.1.28) (EC 1.2.1.5) (EC 1.2.1.7) (Aldehyde dehydrogenase 7)
Q80X90	FLNB_MOUSE	Filamin-B (FLN-B) (ABP-280-like protein) (Actin-binding-like protein) (Beta-filamin)
Q80X95	RRAGA_MOUSE	Ras-related GTP-binding protein A (Rag A) (RagA)
Q80Y84	KDM5B_MOUSE	Lysine-specific demethylase 5B (EC 1.14.11.-) (Histone demethylase JARID1B) (Jumonji/ARID domain-containing protein 1B) (PLU-1)
Q80YP0	CDK3_MOUSE	Cyclin-dependent kinase 3 (EC 2.7.11.22) (Cell division protein kinase 3)
Q80ZJ1	RAP2A_MOUSE	Ras-related protein Rap-2a

Q810B6	ANFY1_MOUSE	Rabankyrin-5 (Rank-5) (Ankyrin repeat and FYVE domain-containing protein 1) (Ankyrin repeats hooked to a zinc finger motif)
Q8BFQ4	WDR82_MOUSE	WD repeat-containing protein 82
Q8BFR5	EFTU_MOUSE	Elongation factor Tu, mitochondrial
Q8BFY6	PEF1_MOUSE	Peflin (PEF protein with a long N-terminal hydrophobic domain) (Penta-EF hand domain-containing protein 1)
Q8BFY9	TNPO1_MOUSE	Transportin-1 (Importin beta-2) (Karyopherin beta-2)
Q8BFZ3	ACTBL_MOUSE	Beta-actin-like protein 2 (Kappa-actin)
Q8BG05	ROA3_MOUSE	Heterogeneous nuclear ribonucleoprotein A3 (hnRNP A3)
Q8BG07	PLD4_MOUSE	5'-3' exonuclease PLD4 (EC 3.1.16.1) (Choline phosphatase 4) (Phosphatidylcholine-hydrolyzing phospholipase D4) (Phospholipase D4) (PLD 4)
Q8BG32	PSD11_MOUSE	26S proteasome non-ATPase regulatory subunit 11 (26S proteasome regulatory subunit RPN6) (26S proteasome regulatory subunit S9) (26S proteasome regulatory subunit p44.5)
Q8BGX0	TRI23_MOUSE	E3 ubiquitin-protein ligase TRIM23 (EC 2.3.2.27) (ADP-ribosylation factor domain-containing protein 1) (GTP-binding protein ARD-1) (RING-type E3 ubiquitin transferase TRIM23) (Tripartite motif-containing protein 23)
Q8BH04	PCKGM_MOUSE	Phosphoenolpyruvate carboxykinase [GTP], mitochondrial (PEPCK-M) (EC 4.1.1.32)
Q8BH15	CNO10_MOUSE	CCR4-NOT transcription complex subunit 10
Q8BH43	WASF2_MOUSE	Wiskott-Aldrich syndrome protein family member 2 (WASP family protein member 2) (Protein WAVE-2)
Q8BHI4	KBTB3_MOUSE	Kelch repeat and BTB domain-containing protein 3 (BTB and kelch domain-containing protein 3)
Q8BHK6	SLAF7_MOUSE	SLAM family member 7 (Leukocyte cell-surface antigen) (Novel Ly9) (CD antigen CD319)
Q8BHL5	ELMO2_MOUSE	Engulfment and cell motility protein 2 (Protein ced-12 homolog A)
Q8BJW6	EIF2A_MOUSE	Eukaryotic translation initiation factor 2A (eIF-2A) [Cleaved into: Eukaryotic translation initiation factor 2A, N-terminally processed]
Q8BJY1	PSMD5_MOUSE	26S proteasome non-ATPase regulatory subunit 5 (26S protease subunit S5 basic) (26S proteasome subunit S5B)
Q8BK67	RCC2_MOUSE	Protein RCC2
Q8BKC5	IPO5_MOUSE	Importin-5 (Imp5) (Importin subunit beta-3) (Karyopherin beta-3) (Ran-binding protein 5) (RanBP5)
Q8BKX6	SMG1_MOUSE	Serine/threonine-protein kinase SMG1 (SMG-1) (EC 2.7.11.1)
Q8BLN6	UNC80_MOUSE	Protein unc-80 homolog (mUNC-80)
Q8BMJ2	SYLC_MOUSE	Leucine--tRNA ligase, cytoplasmic (EC 6.1.1.4) (Leucyl-tRNA synthetase) (LeuRS)
Q8BNA6	FAT3_MOUSE	Protocadherin Fat 3 (FAT tumor suppressor homolog 3)

Q8BP47	SYNC_MOUSE	Asparagine--tRNA ligase, cytoplasmic (EC 6.1.1.22) (Asparaginyl-tRNA synthetase) (AsnRS) (Asparaginyl-tRNA synthetase 1)
Q8BP48	MAP11_MOUSE	Methionine aminopeptidase 1 (MAP 1) (MetAP 1) (EC 3.4.11.18) (Peptidase M 1)
Q8BP67	RL24_MOUSE	60S ribosomal protein L24
Q8BPB0	MOB1B_MOUSE	MOB kinase activator 1B (Mob1 homolog 1A) (Mps one binder kinase activator-like 1A)
Q8BPU7	ELMO1_MOUSE	Engulfment and cell motility protein 1 (Protein ced-12 homolog)
Q8BQC3	IGDC3_MOUSE	Immunoglobulin superfamily DCC subclass member 3 (Putative neuronal cell adhesion molecule)
Q8BTI9	PK3CB_MOUSE	Phosphatidylinositol 4,5-bisphosphate 3-kinase catalytic subunit beta isoform (PI3-kinase subunit beta) (PI3K-beta) (PI3Kbeta) (PtdIns-3-kinase subunit beta) (EC 2.7.1.153) (Phosphatidylinositol 4,5-bisphosphate 3-kinase 110 kDa catalytic subunit beta) (PtdIns-3-kinase subunit p110-beta) (p110beta)
Q8BTM8	FLNA_MOUSE	Filamin-A (FLN-A) (Actin-binding protein 280) (ABP-280) (Alpha-filamin) (Endothelial actin-binding protein) (Filamin-1) (Non-muscle filamin)
Q8BU30	SYIC_MOUSE	Isoleucine--tRNA ligase, cytoplasmic (EC 6.1.1.5) (Isoleucyl-tRNA synthetase) (IRS) (IleRS)
Q8BU31	RAP2C_MOUSE	Ras-related protein Rap-2c
Q8BVE3	VATH_MOUSE	V-type proton ATPase subunit H (V-ATPase subunit H) (Vacuolar proton pump subunit H)
Q8BVF2	PDCL3_MOUSE	Phosducin-like protein 3 (Viral IAP-associated factor 1) (VIAF-1)
Q8BVI4	DHPR_MOUSE	Dihydropteridine reductase (EC 1.5.1.34) (HDHPR) (Quinoid dihydropteridine reductase)
Q8BWM0	PGES2_MOUSE	Prostaglandin E synthase 2 (EC 5.3.99.3) (GATE-binding factor 1) (GBF-1) (Microsomal prostaglandin E synthase 2) (mPGES-2) [Cleaved into: Prostaglandin E synthase 2 truncated form]
Q8BWQ6	VP35L_MOUSE	VPS35 endosomal protein sorting factor-like
Q8BY89	CTL2_MOUSE	Choline transporter-like protein 2 (Solute carrier family 44 member 2)
Q8BYA0	TBCD_MOUSE	Tubulin-specific chaperone D (Beta-tubulin cofactor D) (Tubulin-folding cofactor D)
Q8BYC6	TAOK3_MOUSE	Serine/threonine-protein kinase TAO3 (EC 2.7.11.1) (Thousand and one amino acid protein 3)
Q8BZ98	DYN3_MOUSE	Dynamin-3 (EC 3.6.5.5)
Q8BZN6	DOC10_MOUSE	Dedicator of cytokinesis protein 10 (Zizimin-3)
Q8C0C7	SYFA_MOUSE	Phenylalanine--tRNA ligase alpha subunit (EC 6.1.1.20) (Phenylalanyl-tRNA synthetase alpha subunit) (PheRS)
Q8C0J2	A16L1_MOUSE	Autophagy-related protein 16-1 (APG16-like 1)
Q8C0P5	COR2A_MOUSE	Coronin-2A

Q8C129	LCAP_MOUSE	Leucyl-cystinyl aminopeptidase (Cystinyl aminopeptidase) (EC 3.4.11.3) (Oxytocinase) (OTase)
Q8C147	DOCK8_MOUSE	Dedicator of cytokinesis protein 8
Q8C166	CPNE1_MOUSE	Copine-1 (Copine I)
Q8C196	CPSM_MOUSE	Carbamoyl-phosphate synthase [ammonia], mitochondrial (EC 6.3.4.16) (Carbamoyl-phosphate synthetase I) (CPSase I)
Q8C1A5	THOP1_MOUSE	Thimet oligopeptidase (EC 3.4.24.15)
Q8C1B7	SEP11_MOUSE	Septin-11
Q8C208	IKZF4_MOUSE	Zinc finger protein Eos (Ikaros family zinc finger protein 4)
Q8C2K5	RASL3_MOUSE	RAS protein activator like-3
Q8C3J5	DOCK2_MOUSE	Dedicator of cytokinesis protein 2 (Protein Hch)
Q8C7R4	UBA6_MOUSE	Ubiquitin-like modifier-activating enzyme 6 (Ubiquitin-activating enzyme 6) (EC 6.2.1.45) (Ubiquitin-activating enzyme E1-like protein 2) (E1-L2)
Q8C878	UBA3_MOUSE	NEDD8-activating enzyme E1 catalytic subunit (EC 6.2.1.-) (NEDD8-activating enzyme E1C) (Ubiquitin-activating enzyme E1C) (Ubiquitin-like modifier-activating enzyme 3) (Ubiquitin-activating enzyme 3)
Q8CBE3	WDR37_MOUSE	WD repeat-containing protein 37
Q8CCK0	H2AW_MOUSE	Core histone macro-H2A.2 (Histone macroH2A2) (mH2A2)
Q8CDI7	CC150_MOUSE	Coiled-coil domain-containing protein 150
Q8CDL9	CCD87_MOUSE	Coiled-coil domain-containing protein 87
Q8CDN6	TXNL1_MOUSE	Thioredoxin-like protein 1 (32 kDa thioredoxin-related protein)
Q8CFB4	GBP5_MOUSE	Guanylate-binding protein 5 (EC 3.6.5.-) (GTP-binding protein 5) (GBP-5) (MuGBP-5) (Guanine nucleotide-binding protein 5)
Q8CFK6	DEN1C_MOUSE	DENN domain-containing protein 1C (Connecdenn 3)
Q8CGI1	F193A_MOUSE	Protein FAM193A
Q8CGP6	H2A1H_MOUSE	Histone H2A type 1-H
Q8CHH9	SEPT8_MOUSE	Septin-8
Q8CI51	PDLI5_MOUSE	PDZ and LIM domain protein 5 (Enigma homolog) (Enigma-like PDZ and LIM domains protein)
Q8CI94	PYGB_MOUSE	Glycogen phosphorylase, brain form (EC 2.4.1.1)
Q8CIE6	COPA_MOUSE	Coatomer subunit alpha (Alpha-coat protein) (Alpha-COP) [Cleaved into: Xenin (Xenopsin-related peptide); Proxenin]
Q8CIG8	ANM5_MOUSE	Protein arginine N-methyltransferase 5 (Prmt5) (EC 2.1.1.320) (Histone-arginine N-methyltransferase PRMT5) (Jak-binding protein 1) (Shk1 kinase-binding protein 1 homolog) (SKB1 homolog)
Q8CII2	CD123_MOUSE	Cell division cycle protein 123 homolog
Q8CIN4	PAK2_MOUSE	Serine/threonine-protein kinase PAK 2 (EC 2.7.11.1) (Gamma-PAK) (p21-activated kinase 2) (PAK-2) [Cleaved into: PAK-2p27; PAK-2p34]

Q8CJH3	PLXB1_MOUSE	Plexin-B1
Q8JZQ9	EIF3B_MOUSE	Eukaryotic translation initiation factor 3 subunit B (eIF3b) (Eukaryotic translation initiation factor 3 subunit 9) (eIF-3-eta) (eIF3 p116)
Q8JZR0	ACSL5_MOUSE	Long-chain-fatty-acid--CoA ligase 5 (EC 6.2.1.3) (Arachidonate--CoA ligase) (EC 6.2.1.15) (Long-chain acyl-CoA synthetase 5) (LACS 5)
Q8K0C4	CP51A_MOUSE	Lanosterol 14-alpha demethylase (LDM) (EC 1.14.14.154) (CYPLI) (Cytochrome P450 51A1) (Cytochrome P450-14DM) (Cytochrome P45014DM) (Cytochrome P450LI) (Sterol 14-alpha demethylase)
Q8K183	PDXK_MOUSE	Pyridoxal kinase (EC 2.7.1.35) (Pyridoxine kinase)
Q8K1B8	URP2_MOUSE	Fermitin family homolog 3 (Kindlin-3) (Unc-112-related protein 2)
Q8K1L0	CREB5_MOUSE	Cyclic AMP-responsive element-binding protein 5 (CREB-5) (cAMP-responsive element-binding protein 5) (CRE-BPa)
Q8K1M6	DNM1L_MOUSE	Dynamin-1-like protein (EC 3.6.5.5) (Dynamin family member proline-rich carboxyl-terminal domain less) (Dymple) (Dynamin-related protein 1)
Q8K297	GT251_MOUSE	Procollagen galactosyltransferase 1 (EC 2.4.1.50) (Collagen beta(1-O)galactosyltransferase 1) (Glycosyltransferase 25 family member 1) (Hydroxylysine galactosyltransferase 1)
Q8K2H4	ACAP1_MOUSE	Arf-GAP with coiled-coil, ANK repeat and PH domain-containing protein 1 (Centaurin-beta-1) (Cnt-b1)
Q8K2I9	FBH1_MOUSE	F-box DNA helicase 1 (EC 3.6.4.12) (F-box only protein 18)
Q8K337	I5P2_MOUSE	Type II inositol 1,4,5-trisphosphate 5-phosphatase (EC 3.1.3.36) (Inositol polyphosphate-5-phosphatase B) (Phosphoinositide 5-phosphatase) (5PTase)
Q8K441	ABCA6_MOUSE	ATP-binding cassette sub-family A member 6
Q8QZT1	THIL_MOUSE	Acetyl-CoA acetyltransferase, mitochondrial (EC 2.3.1.9) (Acetoacetyl-CoA thiolase)
Q8QZY1	EIF3L_MOUSE	Eukaryotic translation initiation factor 3 subunit L (eIF3l) (66 kDa tyrosine-rich heat shock protein) (67 kDa polymerase-associated factor) (Eukaryotic translation initiation factor 3 subunit 6-interacting protein) (Eukaryotic translation initiation factor 3 subunit E-interacting protein) (HSP-66Y) (PAF67)
Q8R010	AIMP2_MOUSE	Aminoacyl tRNA synthase complex-interacting multifunctional protein 2 (Multisynthase complex auxiliary component p38) (Protein JTV-1)
Q8R016	BLMH_MOUSE	Bleomycin hydrolase (BH) (BLM hydrolase) (BMH) (EC 3.4.22.40)
Q8R050	ERF3A_MOUSE	Eukaryotic peptide chain release factor GTP-binding subunit ERF3A (Eukaryotic peptide chain release factor subunit 3a) (eRF3a) (G1 to S phase transition protein 1 homolog)

Q8R0W0	EPIPL_MOUSE	Epiplakin
Q8R105	VP37C_MOUSE	Vacuolar protein sorting-associated protein 37C (ESCRT-I complex subunit VPS37C)
Q8R146	APEH_MOUSE	Acylamino-acid-releasing enzyme (AARE) (EC 3.4.19.1) (Acyl-peptide hydrolase) (APH) (Acylaminoacyl-peptidase)
Q8R1A4	DOCK7_MOUSE	Dedicator of cytokinesis protein 7 (Protein moonlight)
Q8R1B4	EIF3C_MOUSE	Eukaryotic translation initiation factor 3 subunit C (eIF3c) (Eukaryotic translation initiation factor 3 subunit 8) (eIF3 p110)
Q8R1Q8	DC1L1_MOUSE	Cytoplasmic dynein 1 light intermediate chain 1 (Dynein light chain A) (DLC-A) (Dynein light intermediate chain 1, cytosolic)
Q8R1S0	COQ6_MOUSE	Ubiquinone biosynthesis monooxygenase COQ6, mitochondrial (EC 1.14.13.-) (Coenzyme Q10 monooxygenase 6)
Q8R1V4	TMED4_MOUSE	Transmembrane emp24 domain-containing protein 4 (Endoplasmic reticulum stress-response protein 25) (ERS25) (p24 family protein alpha-3) (p24alpha3) (p26)
Q8R322	GLE1_MOUSE	Nucleoporin GLE1 (GLE1-like protein)
Q8R366	IGSF8_MOUSE	Immunoglobulin superfamily member 8 (IgSF8) (CD81 partner 3) (Glu-Trp-Ile EWI motif-containing protein 2) (EWI-2) (Keratinocyte-associated transmembrane protein 4) (KCT-4) (Prostaglandin regulatory-like protein) (PGRL) (CD antigen CD316)
Q8R480	NUP85_MOUSE	Nuclear pore complex protein Nup85 (85 kDa nucleoporin) (FROUNT) (Nucleoporin Nup85) (Pericentrin-1)
Q8R550	SH3K1_MOUSE	SH3 domain-containing kinase-binding protein 1 (Regulator of ubiquitous kinase) (Ruk) (SH3-containing, expressed in tumorigenic astrocytes)
Q8R5C5	ACTY_MOUSE	Beta-centractin (Actin-related protein 1B) (ARP1B)
Q8VCW8	ACSF2_MOUSE	Medium-chain acyl-CoA ligase ACSF2, mitochondrial (EC 6.2.1.2)
Q8VD58	EVI2B_MOUSE	Protein EVI2B (Ecotropic viral integration site 2B protein) (EVI-2B) (CD antigen CD361)
Q8VDD5	MYH9_MOUSE	Myosin-9 (Cellular myosin heavy chain, type A) (Myosin heavy chain 9) (Myosin heavy chain, non-muscle IIa) (Non-muscle myosin heavy chain A) (NMMHC-A) (Non-muscle myosin heavy chain IIa) (NMMHC II-a) (NMMHC-IIA)
Q8VDJ3	VIGLN_MOUSE	Vigilin (High density lipoprotein-binding protein) (HDL-binding protein)
Q8VDM4	PSMD2_MOUSE	26S proteasome non-ATPase regulatory subunit 2 (26S proteasome regulatory subunit RPN1) (26S proteasome regulatory subunit S2) (26S proteasome subunit p97)
Q8VDN2	AT1A1_MOUSE	Sodium/potassium-transporting ATPase subunit alpha-1 (Na(+)/K(+) ATPase alpha-1 subunit) (EC 7.2.2.13) (Sodium pump subunit alpha-1)

Q8VDP3	MICA1_MOUSE	[F-actin]-monooxygenase MICAL1 (EC 1.14.13.225) (Molecule interacting with CasL protein 1) (MICAL-1) (mMical1) (NEDD9-interacting protein with calponin homology and LIM domains)
Q8VDW0	DX39A_MOUSE	ATP-dependent RNA helicase DDX39A (EC 3.6.4.13) (DEAD box protein 39)
Q8VE70	PDC10_MOUSE	Programmed cell death protein 10 (TF-1 cell apoptosis-related protein 15)
Q8VED5	K2C79_MOUSE	Keratin, type II cytoskeletal 79 (Cytokeratin-79) (CK-79) (Keratin-79) (K79) (Type-II keratin Kb38)
Q8VEK0	CC50A_MOUSE	Cell cycle control protein 50A (P4-ATPase flippase complex beta subunit TMEM30A) (Transmembrane protein 30A)
Q8VEK3	HNRPU_MOUSE	Heterogeneous nuclear ribonucleoprotein U (hnRNP U) (Scaffold-attachment factor A) (SAF-A)
Q8VEM1	GOLI_MOUSE	E3 ubiquitin-protein ligase RNF130 (EC 2.3.2.27) (G1-related zinc finger protein) (Goliath homolog) (RING finger protein 130) (RING-type E3 ubiquitin transferase RNF130)
Q8VIJ6	SFPQ_MOUSE	Splicing factor, proline- and glutamine-rich (DNA-binding p52/p100 complex, 100 kDa subunit) (Polypyrimidine tract-binding protein-associated-splicing factor) (PSF) (PTB-associated-splicing factor)
Q91V08	CLC2D_MOUSE	C-type lectin domain family 2 member D (C-type lectin-related protein B) (Clr-b) (Lectin-like transmembrane protein) (Osteoclast inhibitory lectin)
Q91V12	BACH_MOUSE	Cytosolic acyl coenzyme A thioester hydrolase (EC 3.1.2.2) (Acyl-CoA thioesterase 7) (Brain acyl-CoA hydrolase) (BACH) (CTE-IIa) (CTE-II) (Long chain acyl-CoA thioester hydrolase)
Q91V41	RAB14_MOUSE	Ras-related protein Rab-14
Q91V64	ISOC1_MOUSE	Isochorismatase domain-containing protein 1
Q91V92	ACLY_MOUSE	ATP-citrate synthase (EC 2.3.3.8) (ATP-citrate (pro-S-)-lyase) (Citrate cleavage enzyme)
Q91VH2	SNX9_MOUSE	Sorting nexin-9
Q91VI7	RINI_MOUSE	Ribonuclease inhibitor (Ribonuclease/angiogenin inhibitor 1)
Q91VK4	ITM2C_MOUSE	Integral membrane protein 2C (Transmembrane protein BRI3) [Cleaved into: CT-BRI3]
Q91VX2	UBAP2_MOUSE	Ubiquitin-associated protein 2 (UBAP-2) (Protein lingerer homolog 1) (mLig-1)
Q91W89	MA2C1_MOUSE	Alpha-mannosidase 2C1 (EC 3.2.1.24) (Alpha-D-mannoside mannohydrolase) (Mannosidase alpha class 2C member 1) (Neutral/cytosolic alpha-mannosidase)
Q91WK2	EIF3H_MOUSE	Eukaryotic translation initiation factor 3 subunit H (eIF3h) (Eukaryotic translation initiation factor 3 subunit 3) (eIF-3-gamma) (eIF3 p40 subunit)

Q91WQ3	SYYC_MOUSE	Tyrosine--tRNA ligase, cytoplasmic (EC 6.1.1.1) (Tyrosyl-tRNA synthetase) (TyrRS) [Cleaved into: Tyrosine--tRNA ligase, cytoplasmic, N-terminally processed]
Q91XV3	BASP1_MOUSE	Brain acid soluble protein 1 (22 kDa neuronal tissue-enriched acidic protein) (Neuronal axonal membrane protein NAP-22)
Q91Y20	PCDAA_MOUSE	Protocadherin alpha-10 (PCDH-alpha-10)
Q91YI0	ARLY_MOUSE	Argininosuccinate lyase (ASAL) (EC 4.3.2.1) (Arginosuccinase)
Q91YI4	ARRB2_MOUSE	Beta-arrestin-2 (Arrestin beta-2)
Q91YQ5	RPN1_MOUSE	Dolichyl-diphosphooligosaccharide--protein glycosyltransferase subunit 1 (Dolichyl-diphosphooligosaccharide--protein glycosyltransferase 67 kDa subunit) (Ribophorin I) (RPN-I) (Ribophorin-1)
Q91YR1	TWF1_MOUSE	Twinfilin-1 (Protein A6)
Q91YR9	PTGR1_MOUSE	Prostaglandin reductase 1 (PRG-1) (EC 1.3.1.-) (15-oxoprostaglandin 13-reductase) (EC 1.3.1.48) (NADP-dependent leukotriene B4 12-hydroxydehydrogenase) (EC 1.3.1.74)
Q91Z67	SRGP2_MOUSE	SLIT-ROBO Rho GTPase-activating protein 2 (srGAP2) (Formin-binding protein 2) (Formin-binding protein 27) (FBP-27)
Q91ZJ5	UGPA_MOUSE	UTP--glucose-1-phosphate uridylyltransferase (EC 2.7.7.9) (UDP-glucose pyrophosphorylase) (UDPGP) (UGPase)
Q91ZR1	RAB4B_MOUSE	Ras-related protein Rab-4B
Q920E5	FPPS_MOUSE	Farnesyl pyrophosphate synthase (FPP synthase) (FPS) (EC 2.5.1.10) ((2E,6E)-farnesyl diphosphate synthase) (Cholesterol-regulated 39 kDa protein) (CR 39) (Dimethylallyltranstransferase) (EC 2.5.1.1) (Farnesyl diphosphate synthase) (Geranyltranstransferase)
Q921F2	TADBP_MOUSE	TAR DNA-binding protein 43 (TDP-43)
Q921H8	THIKA_MOUSE	3-ketoacyl-CoA thiolase A, peroxisomal (EC 2.3.1.16) (Acetyl-CoA acyltransferase A) (Beta-ketothiolase A) (Peroxisomal 3-oxoacyl-CoA thiolase A)
Q921M3	SF3B3_MOUSE	Splicing factor 3B subunit 3 (Pre-mRNA-splicing factor SF3b 130 kDa subunit) (SF3b130) (Spliceosome-associated protein 130) (SAP 130)
Q921M7	FA49B_MOUSE	Protein FAM49B
Q921Z5	TFIP8_MOUSE	Tumor necrosis factor alpha-induced protein 8 (TNF alpha-induced protein 8)
Q922B2	SYDC_MOUSE	Aspartate--tRNA ligase, cytoplasmic (EC 6.1.1.12) (Aspartyl-tRNA synthetase) (AspRS)
Q922F4	TBB6_MOUSE	Tubulin beta-6 chain
Q922R8	PDIA6_MOUSE	Protein disulfide-isomerase A6 (EC 5.3.4.1) (Thioredoxin domain-containing protein 7)
Q922U2	K2C5_MOUSE	Keratin, type II cytoskeletal 5 (Cytokeratin-5) (CK-5) (Keratin-5) (K5) (Type-II keratin Kb5)
Q923D2	BLVRB_MOUSE	Flavin reductase (NADPH) (FR) (EC 1.5.1.30) (Biliverdin reductase B) (BVR-B) (EC 1.3.1.24) (Biliverdin-IX beta-

		reductase) (NADPH-dependent diaphorase) (NADPH-flavin reductase) (FLR)
Q923S6	NEUL1_MOUSE	E3 ubiquitin-protein ligase NEURL1 (EC 2.3.2.27) (Neuralized-like protein 1A) (m-neu1) (m-neuralized 1) (Neuralized1) (RING-type E3 ubiquitin transferase NEURL1)
Q925J9	MED1_MOUSE	Mediator of RNA polymerase II transcription subunit 1 (Mediator complex subunit 1) (Peroxisome proliferator-activated receptor-binding protein) (PBP) (PPAR-binding protein) (Thyroid hormone receptor-associated protein complex 220 kDa component) (Trap220) (Thyroid receptor-interacting protein 2) (TR-interacting protein 2) (TRIP-2)
Q93092	TALDO_MOUSE	Transaldolase (EC 2.2.1.2)
Q99020	ROAA_MOUSE	Heterogeneous nuclear ribonucleoprotein A/B (hnRNP A/B) (CArG-binding factor-A) (CBF-A)
Q99J72	ABEC3_MOUSE	DNA dC->dU-editing enzyme APOBEC-3 (EC 3.5.4.38) (Apolipoprotein B mRNA-editing complex 3) (Arp3) (CEM-15) (CEM15)
Q99J93	IFM2_MOUSE	Interferon-induced transmembrane protein 2 (Dispanin subfamily A member 2c) (DSPA2c) (Fragilis protein 3)
Q99JI4	PSMD6_MOUSE	26S proteasome non-ATPase regulatory subunit 6 (26S proteasome regulatory subunit RPN7) (26S proteasome regulatory subunit S10) (p42A)
Q99JI6	RAP1B_MOUSE	Ras-related protein Rap-1b (GTP-binding protein smg p21B)
Q99JW4	LIMS1_MOUSE	LIM and senescent cell antigen-like-containing domain protein 1 (Particularly interesting new Cys-His protein 1) (PINCH-1)
Q99JX4	EIF3M_MOUSE	Eukaryotic translation initiation factor 3 subunit M (eIF3m) (PCI domain-containing protein 1)
Q99JY9	ARP3_MOUSE	Actin-related protein 3 (Actin-like protein 3)
Q99K70	RRAGC_MOUSE	Ras-related GTP-binding protein C (Rag C) (RagC) (GTPase-interacting protein 2) (TIB929)
Q99KH8	STK24_MOUSE	Serine/threonine-protein kinase 24 (EC 2.7.11.1) (Mammalian STE20-like protein kinase 3) (MST-3) (STE20-like kinase MST3) [Cleaved into: Serine/threonine-protein kinase 24 35 kDa subunit (Mammalian STE20-like protein kinase 3 N-terminal) (MST3/N); Serine/threonine-protein kinase 24 12 kDa subunit (Mammalian STE20-like protein kinase 3 C-terminal) (MST3/C)]
Q99KJ8	DCTN2_MOUSE	Dynactin subunit 2 (50 kDa dynein-associated polypeptide) (Dynactin complex 50 kDa subunit) (DCTN-50) (Growth cone membrane protein 23-48K) (GMP23-48K) (p50 dynamitin)
Q99KK2	NEUA_MOUSE	N-acylneuraminate cytidylyltransferase (EC 2.7.7.43) (CMP-N-acetylneuraminic acid synthase) (CMP-NeuNAc synthase)

Q99KK7	DPP3_MOUSE	Dipeptidyl peptidase 3 (EC 3.4.14.4) (Dipeptidyl aminopeptidase III) (Dipeptidyl arylamidase III) (Dipeptidyl peptidase III) (DPP III) (Enkephalinase B)
Q99KP6	PRP19_MOUSE	Pre-mRNA-processing factor 19 (EC 2.3.2.27) (Nuclear matrix protein 200) (PRP19/PSO4 homolog) (RING-type E3 ubiquitin transferase PRP19) (Senescence evasion factor)
Q99KV1	DJB11_MOUSE	DnaJ homolog subfamily B member 11 (APOBEC1-binding protein 2) (ABBP-2) (ER-associated DNAJ) (ER-associated Hsp40 co-chaperone) (Endoplasmic reticulum DNA J domain-containing protein 3) (ER-resident protein ERdj3) (ERdj3) (ERj3p)
Q99L45	IF2B_MOUSE	Eukaryotic translation initiation factor 2 subunit 2 (Eukaryotic translation initiation factor 2 subunit beta) (eIF-2-beta)
Q99L47	F10A1_MOUSE	Hsc70-interacting protein (Hip) (Protein FAM10A1) (Protein ST13 homolog)
Q99LC5	ETFA_MOUSE	Electron transfer flavoprotein subunit alpha, mitochondrial (Alpha-ETF)
Q99LD4	CSN1_MOUSE	COP9 signalosome complex subunit 1 (SGN1) (Signalosome subunit 1) (G protein pathway suppressor 1) (GPS-1) (JAB1-containing signalosome subunit 1)
Q99LD9	EI2BB_MOUSE	Translation initiation factor eIF-2B subunit beta (eIF-2B GDP-GTP exchange factor subunit beta)
Q99LF4	RTCB_MOUSE	RNA-splicing ligase RtcB homolog (EC 6.5.1.8) (3'-phosphate/5'-hydroxy nucleic acid ligase) (Focal adhesion-associated protein) (FAAP)
Q99LI8	HGS_MOUSE	Hepatocyte growth factor-regulated tyrosine kinase substrate
Q99LT0	DPY30_MOUSE	Protein dpy-30 homolog (Dpy-30-like protein) (Dpy-30L)
Q99LX0	PARK7_MOUSE	Protein/nucleic acid deglycase DJ-1 (EC 3.1.2.-) (EC 3.5.1.-) (EC 3.5.1.124) (Maillard deglycase) (Parkinson disease protein 7 homolog) (Parkinsonism-associated deglycase) (Protein DJ-1) (DJ-1)
Q99LX5	MMTA2_MOUSE	Multiple myeloma tumor-associated protein 2 homolog
Q99M74	KRT82_MOUSE	Keratin, type II cuticular Hb2 (Keratin-82) (K82) (Type II hair keratin Hb2) (Type-II keratin Kb22)
Q99MI1	RB6I2_MOUSE	ELKS/Rab6-interacting/CAST family member 1 (ERC-1) (CAZ-associated structural protein 2) (CAST2) (Rab6-interacting protein 2)
Q99MK8	ARBK1_MOUSE	Beta-adrenergic receptor kinase 1 (Beta-ARK-1) (EC 2.7.11.15) (G-protein-coupled receptor kinase 2)
Q99MN1	SYK_MOUSE	Lysine--tRNA ligase (EC 2.7.7.-) (EC 6.1.1.6) (Lysyl-tRNA synthetase) (LysRS)
Q99P72	RTN4_MOUSE	Reticulon-4 (Neurite outgrowth inhibitor) (Nogo protein)
Q99PV0	PRP8_MOUSE	Pre-mRNA-processing-splicing factor 8 (Splicing factor Prp8)
Q9CPR4	RL17_MOUSE	60S ribosomal protein L17

Q9CPW4	ARPC5_MOUSE	Actin-related protein 2/3 complex subunit 5 (Arp2/3 complex 16 kDa subunit) (p16-ARC)
Q9CPY7	AMPL_MOUSE	Cytosol aminopeptidase (EC 3.4.11.1) (Leucine aminopeptidase 3) (LAP-3) (Leucyl aminopeptidase) (Proline aminopeptidase) (EC 3.4.11.5) (Prolyl aminopeptidase)
Q9CQ22	LTOR1_MOUSE	Ragulator complex protein LAMTOR1 (Late endosomal/lysosomal adaptor and MAPK and MTOR activator 1) (Lipid raft adaptor protein p18)
Q9CQ60	6PGL_MOUSE	6-phosphogluconolactonase (6PGL) (EC 3.1.1.31)
Q9CQ65	MTAP_MOUSE	S-methyl-5'-thioadenosine phosphorylase (EC 2.4.2.28) (5'-methylthioadenosine phosphorylase) (MTA phosphorylase) (MTAP) (MTAPase)
Q9CQ71	RFA3_MOUSE	Replication protein A 14 kDa subunit (RP-A p14) (Replication factor A protein 3) (RF-A protein 3)
Q9CQC6	BZW1_MOUSE	Basic leucine zipper and W2 domain-containing protein 1
Q9CQC8	SPG21_MOUSE	Maspardin (Acid cluster protein 33) (Spastic paraplegia 21 autosomal recessive Mast syndrome protein homolog)
Q9CQC9	SAR1B_MOUSE	GTP-binding protein SAR1b
Q9CQD1	RAB5A_MOUSE	Ras-related protein Rab-5A (EC 3.6.5.2)
Q9CQI6	COTL1_MOUSE	Coactosin-like protein
Q9CQM9	GLRX3_MOUSE	Glutaredoxin-3 (PKC-interacting cousin of thioredoxin) (PICOT) (PKC-theta-interacting protein) (PKCq-interacting protein) (Thioredoxin-like protein 2)
Q9CQN1	TRAP1_MOUSE	Heat shock protein 75 kDa, mitochondrial (HSP 75) (TNFR-associated protein 1) (Tumor necrosis factor type 1 receptor-associated protein) (TRAP-1)
Q9CQQ7	AT5F1_MOUSE	ATP synthase F(0) complex subunit B1, mitochondrial (ATP synthase peripheral stalk-membrane subunit b) (ATP synthase subunit b) (ATPase subunit b)
Q9CQR6	PPP6_MOUSE	Serine/threonine-protein phosphatase 6 catalytic subunit (PP6C) (EC 3.1.3.16)
Q9CQV8	1433B_MOUSE	14-3-3 protein beta/alpha (Protein kinase C inhibitor protein 1) (KCIP-1) [Cleaved into: 14-3-3 protein beta/alpha, N-terminally processed]
Q9CQW1	YKT6_MOUSE	Synaptobrevin homolog YKT6 (EC 2.3.1.-)
Q9CQW2	ARL8B_MOUSE	ADP-ribosylation factor-like protein 8B (ADP-ribosylation factor-like protein 10C) (Novel small G protein indispensable for equal chromosome segregation 1)
Q9CQX2	CYB5B_MOUSE	Cytochrome b5 type B (Cytochrome b5 outer mitochondrial membrane isoform)
Q9CR16	PPID_MOUSE	Peptidyl-prolyl cis-trans isomerase D (PPIase D) (EC 5.2.1.8) (40 kDa peptidyl-prolyl cis-trans isomerase) (Cyclophilin-40) (CYP-40) (Rotamase D)
Q9CR57	RL14_MOUSE	60S ribosomal protein L14
Q9CR64	KISHA_MOUSE	Protein kish-A (Transmembrane protein 167) (Transmembrane protein 167A)

Q9CRB5	PR7C1_MOUSE	Prolactin-7C1 (Placental prolactin-like protein O) (PLP-O) (PRL-like protein O)
Q9CS42	PRPS2_MOUSE	Ribose-phosphate pyrophosphokinase 2 (EC 2.7.6.1) (Phosphoribosyl pyrophosphate synthase II) (PRS-II)
Q9CVB6	ARPC2_MOUSE	Actin-related protein 2/3 complex subunit 2 (Arp2/3 complex 34 kDa subunit) (p34-ARC)
Q9CWJ3	KTBL1_MOUSE	KATNB1-like protein 1 (Katanin p80 subunit B-like 1)
Q9CWJ9	PUR9_MOUSE	Bifunctional purine biosynthesis protein PURH [Includes: Phosphoribosylaminoimidazolecarboxamide formyltransferase (EC 2.1.2.3) (5-aminoimidazole-4-carboxamide ribonucleotide formyltransferase) (AICAR transformylase); IMP cyclohydrolase (EC 3.5.4.10) (ATIC) (IMP synthase) (Inosinicase)]
Q9CWK8	SNX2_MOUSE	Sorting nexin-2
Q9CX00	IST1_MOUSE	IST1 homolog
Q9CY50	SSRA_MOUSE	Translocon-associated protein subunit alpha (TRAP-alpha) (Signal sequence receptor subunit alpha) (SSR-alpha)
Q9CYG7	TOM34_MOUSE	Mitochondrial import receptor subunit TOM34 (Translocase of outer membrane 34 kDa subunit)
Q9CYL5	GAPR1_MOUSE	Golgi-associated plant pathogenesis-related protein 1 (GAPR-1) (Golgi-associated PR-1 protein) (Glioma pathogenesis-related protein 2) (GliPR 2)
Q9CYN9	RENR_MOUSE	Renin receptor (ATPase H(+)-transporting lysosomal accessory protein 2) (ATPase H(+)-transporting lysosomal-interacting protein 2) (Renin/prorenin receptor) [Cleaved into: Renin receptor extracellular fragment; Renin receptor cytoplasmic fragment]
Q9CYZ2	TPD54_MOUSE	Tumor protein D54 (Tumor protein D52-like 2)
Q9CZ44	NSF1C_MOUSE	NSFL1 cofactor p47 (p97 cofactor p47)
Q9CZ52	ANTR1_MOUSE	Anthrax toxin receptor 1 (Tumor endothelial marker 8)
Q9CZD3	GARS_MOUSE	Glycine--tRNA ligase (EC 6.1.1.14) (Diadenosine tetraphosphate synthetase) (Ap4A synthetase) (EC 2.7.7.-) (Glycyl-tRNA synthetase 1) (GlyRS)
Q9CZM2	RL15_MOUSE	60S ribosomal protein L15
Q9CZU6	CISY_MOUSE	Citrate synthase, mitochondrial (EC 2.3.3.1) (Citrate (Si)-synthase)
Q9CZX8	RS19_MOUSE	40S ribosomal protein S19
Q9CZY3	UB2V1_MOUSE	Ubiquitin-conjugating enzyme E2 variant 1 (UEV-1) (CROC-1)
Q9D0F9	PGM1_MOUSE	Phosphoglucomutase-1 (PGM 1) (EC 5.4.2.2) (Glucose phosphomutase 1) (Phosphoglucomutase-2)
Q9D0I9	SYRC_MOUSE	Arginine--tRNA ligase, cytoplasmic (EC 6.1.1.19) (Arginyl-tRNA synthetase) (ArgRS)
Q9D0K2	SCOT1_MOUSE	Succinyl-CoA:3-ketoacid coenzyme A transferase 1, mitochondrial (EC 2.8.3.5) (3-oxoacid CoA-transferase 1) (Somatic-type succinyl-CoA:3-oxoacid CoA-transferase) (SCOT-s)

Q9D0R2	SYTC_MOUSE	Threonine--tRNA ligase 1, cytoplasmic (EC 6.1.1.3) (Threonine--tRNA ligase, cytoplasmic) (Threonyl-tRNA synthetase) (ThrRS) (Threonyl-tRNA synthetase 1)
Q9D0T1	NH2L1_MOUSE	NHP2-like protein 1 (Fertilization antigen 1) (FA-1) (High mobility group-like nuclear protein 2 homolog 1) (Sperm-specific antigen 1) (U4/U6.U5 small nuclear ribonucleoprotein SNU13) (U4/U6.U5 tri-snRNP 15.5 kDa protein) [Cleaved into: NHP2-like protein 1, N-terminally processed]
Q9D1A2	CNDP2_MOUSE	Cytosolic non-specific dipeptidase (EC 3.4.13.18) (CNDP dipeptidase 2) (Glutamate carboxypeptidase-like protein 1)
Q9D1C8	VPS28_MOUSE	Vacuolar protein sorting-associated protein 28 homolog (Caspase-activated DNase inhibitor that interacts with ASK1) (CIIA) (ESCRT-I complex subunit VPS28)
Q9D1D4	TMEDA_MOUSE	Transmembrane emp24 domain-containing protein 10 (21 kDa transmembrane-trafficking protein) (Transmembrane protein Tmp21) (p24 family protein delta-1) (p24delta1)
Q9D1G1	RAB1B_MOUSE	Ras-related protein Rab-1B
Q9D1K2	VATF_MOUSE	V-type proton ATPase subunit F (V-ATPase subunit F) (V-ATPase 14 kDa subunit) (Vacuolar proton pump subunit F)
Q9D1M0	SEC13_MOUSE	Protein SEC13 homolog (GATOR complex protein SEC13) (SEC13-like protein 1) (SEC13-related protein)
Q9D1Q6	ERP44_MOUSE	Endoplasmic reticulum resident protein 44 (ER protein 44) (ERp44) (Thioredoxin domain-containing protein 4)
Q9D2V7	CORO7_MOUSE	Coronin-7 (Crn7) (70 kDa WD repeat tumor rejection antigen homolog)
Q9D358	PPAC_MOUSE	Low molecular weight phosphotyrosine protein phosphatase (LMW-PTP) (LMW-PTPase) (EC 3.1.3.48) (Low molecular weight cytosolic acid phosphatase) (EC 3.1.3.2)
Q9D379	HYEP_MOUSE	Epoxide hydrolase 1 (EC 3.3.2.9) (Epoxide hydratase) (Microsomal epoxide hydrolase) (mEH)
Q9D620	RFIP1_MOUSE	Rab11 family-interacting protein 1 (Rab11-FIP1) (Rab-coupling protein)
Q9D662	SC23B_MOUSE	Protein transport protein Sec23B (SEC23-related protein B)
Q9D6F9	TBB4A_MOUSE	Tubulin beta-4A chain (Tubulin beta-4 chain)
Q9D6Z1	NOP56_MOUSE	Nucleolar protein 56 (Nucleolar protein 5A)
Q9D7V9	NAAA_MOUSE	N-acylethanolamine-hydrolyzing acid amidase (EC 3.5.1.60) (N-acylsphingosine amidohydrolase-like) (ASAH-like protein) [Cleaved into: N-acylethanolamine-hydrolyzing acid amidase subunit alpha; N-acylethanolamine-hydrolyzing acid amidase subunit beta]
Q9D7X3	DUS3_MOUSE	Dual specificity protein phosphatase 3 (EC 3.1.3.16) (EC 3.1.3.48) (T-DSP11) (Vaccinia H1-related phosphatase) (VHR)
Q9D898	ARP5L_MOUSE	Actin-related protein 2/3 complex subunit 5-like protein (Arp2/3 complex 16 kDa subunit 2) (ARC16-2)
Q9D8B3	CHM4B_MOUSE	Charged multivesicular body protein 4b (Chromatin-modifying protein 4b) (CHMP4b)

Q9D8C4	IN35_MOUSE	Interferon-induced 35 kDa protein homolog (IFP 35) (Ifi-35)
Q9D8M3	HRG1_MOUSE	Heme transporter HRG1 (Heme-responsive gene 1 protein homolog) (HRG-1) (Solute carrier family 48 member 1)
Q9D8N0	EF1G_MOUSE	Elongation factor 1-gamma (EF-1-gamma) (eEF-1B gamma)
Q9D8T2	GSDMD_MOUSE	Gasdermin-D (Gasdermin domain-containing protein 1) [Cleaved into: Gasdermin-D, N-terminal (GSDMD-NT); Gasdermin-D, C-terminal (GSDMD-CT)]
Q9D8W5	PSD12_MOUSE	26S proteasome non-ATPase regulatory subunit 12 (26S proteasome regulatory subunit RPN5) (26S proteasome regulatory subunit p55)
Q9D8Y0	EFHD2_MOUSE	EF-hand domain-containing protein D2 (Swiprosin-1)
Q9D906	ATG7_MOUSE	Ubiquitin-like modifier-activating enzyme ATG7 (ATG12-activating enzyme E1 ATG7) (Autophagy-related protein 7) (APG7-like) (mAGP7) (Ubiquitin-activating enzyme E1-like protein)
Q9DAR7	DCPS_MOUSE	m7GpppX diphosphatase (EC 3.6.1.59) (DCS-1) (Decapping scavenger enzyme) (Hint-related 7meGMP-directed hydrolase) (Histidine triad nucleotide-binding protein 5) (Histidine triad protein member 5) (HINT-5) (Scavenger mRNA-decapping enzyme DcpS)
Q9DAS9	GBG12_MOUSE	Guanine nucleotide-binding protein G(I)/G(S)/G(O) subunit gamma-12
Q9DB05	SNAA_MOUSE	Alpha-soluble NSF attachment protein (SNAP-alpha) (N-ethylmaleimide-sensitive factor attachment protein alpha)
Q9DB27	MCTS1_MOUSE	Malignant T-cell-amplified sequence 1 (MCT-1) (Multiple copies T-cell malignancies 1)
Q9DB29	IAH1_MOUSE	Isoamyl acetate-hydrolyzing esterase 1 homolog (EC 3.1.-.-)
Q9DBC7	KAP0_MOUSE	cAMP-dependent protein kinase type I-alpha regulatory subunit [Cleaved into: cAMP-dependent protein kinase type I-alpha regulatory subunit, N-terminally processed]
Q9DBG3	AP2B1_MOUSE	AP-2 complex subunit beta (AP105B) (Adaptor protein complex AP-2 subunit beta) (Adaptor-related protein complex 2 subunit beta) (Beta-2-adaptin) (Beta-adaptin) (Clathrin assembly protein complex 2 beta large chain) (Plasma membrane adaptor HA2/AP2 adaptin beta subunit)
Q9DBG5	PLIN3_MOUSE	Perilipin-3 (Cargo selection protein TIP47) (Mannose-6-phosphate receptor-binding protein 1)
Q9DBJ1	PGAM1_MOUSE	Phosphoglycerate mutase 1 (EC 5.4.2.11) (EC 5.4.2.4) (BPG-dependent PGAM 1) (Phosphoglycerate mutase isozyme B) (PGAM-B)
Q9DBP5	KCY_MOUSE	UMP-CMP kinase (EC 2.7.4.14) (Deoxycytidylate kinase) (CK) (dCMP kinase) (Nucleoside-diphosphate kinase) (EC 2.7.4.6) (Uridine monophosphate/cytidine monophosphate kinase) (UMP/CMP kinase) (UMP/CMPK)
Q9DBR4	APBB2_MOUSE	Amyloid-beta A4 precursor protein-binding family B member 2

Q9DBT5	AMPD2_MOUSE	AMP deaminase 2 (EC 3.5.4.6) (AMP deaminase isoform L)
Q9DBU0	TM9S1_MOUSE	Transmembrane 9 superfamily member 1
Q9DBZ5	EIF3K_MOUSE	Eukaryotic translation initiation factor 3 subunit K (eIF3k) (Eukaryotic translation initiation factor 3 subunit 12) (eIF-3 p25)
Q9DC04	RGS3_MOUSE	Regulator of G-protein signaling 3 (RGS3) (C2PA)
Q9DC51	GNAI3_MOUSE	Guanine nucleotide-binding protein G(i) subunit alpha (G(i) alpha-3)
Q9DC61	MPPA_MOUSE	Mitochondrial-processing peptidase subunit alpha (Alpha-MPP) (Inactive zinc metalloprotease alpha) (P-55)
Q9DCD0	6PGD_MOUSE	6-phosphogluconate dehydrogenase, decarboxylating (EC 1.1.1.44)
Q9DCH4	EIF3F_MOUSE	Eukaryotic translation initiation factor 3 subunit F (eIF3f) (Deubiquitinating enzyme eIF3f) (EC 3.4.19.12) (Eukaryotic translation initiation factor 3 subunit 5) (eIF-3-epsilon) (eIF3 p47)
Q9DCL9	PUR6_MOUSE	Multifunctional protein ADE2 [Includes: Phosphoribosylaminoimidazole-succinocarboxamide synthase (EC 6.3.2.6) (SAICAR synthetase); Phosphoribosylaminoimidazole carboxylase (EC 4.1.1.21) (AIR carboxylase) (AIRC)]
Q9DCN2	NB5R3_MOUSE	NADH-cytochrome b5 reductase 3 (B5R) (Cytochrome b5 reductase) (EC 1.6.2.2) (Diaphorase-1) [Cleaved into: NADH-cytochrome b5 reductase 3 membrane-bound form; NADH-cytochrome b5 reductase 3 soluble form]
Q9DCW4	ETFB_MOUSE	Electron transfer flavoprotein subunit beta (Beta-ETF)
Q9EP69	SAC1_MOUSE	Phosphatidylinositide phosphatase SAC1 (EC 3.1.3.-) (Suppressor of actin mutations 1-like protein)
Q9EP73	PD1L1_MOUSE	Programmed cell death 1 ligand 1 (PD-L1) (PDCD1 ligand 1) (Programmed death ligand 1) (B7 homolog 1) (B7-H1) (CD antigen CD274)
Q9EPB4	ASC_MOUSE	Apoptosis-associated speck-like protein containing a CARD (mASC) (PYD and CARD domain-containing protein)
Q9EPK7	XPO7_MOUSE	Exportin-7 (Exp7) (Ran-binding protein 16)
Q9EPL8	IPO7_MOUSE	Importin-7 (Imp7) (Ran-binding protein 7) (RanBP7)
Q9EPL9	ACOX3_MOUSE	Peroxisomal acyl-coenzyme A oxidase 3 (EC 1.3.3.6) (Branched-chain acyl-CoA oxidase) (BRCACox) (Pristanoyl-CoA oxidase)
Q9EPR4	S23A2_MOUSE	Solute carrier family 23 member 2 (Na(+)/L-ascorbic acid transporter 2) (Sodium-dependent vitamin C transporter 2) (SVCT-2) (mSVCT2) (Yolk sac permease-like molecule 2)
Q9EQK5	MVP_MOUSE	Major vault protein (MVP)
Q9EQP2	EHD4_MOUSE	EH domain-containing protein 4 (PAST homolog 2) (mPAST2)
Q9EQU5	SET_MOUSE	Protein SET (Phosphatase 2A inhibitor I2PP2A) (I-2PP2A) (Template-activating factor I) (TAF-I)
Q9ERD8	PARVG_MOUSE	Gamma-parvin

Q9ERF3	WDR61_MOUSE	WD repeat-containing protein 61 (Meiotic recombination REC14 protein homolog) [Cleaved into: WD repeat-containing protein 61, N-terminally processed]
Q9ERN0	SCAM2_MOUSE	Secretory carrier-associated membrane protein 2 (Secretory carrier membrane protein 2)
Q9ES03	TBX20_MOUSE	T-box transcription factor TBX20 (T-box protein 20)
Q9ES52	SHIP1_MOUSE	Phosphatidylinositol 3,4,5-trisphosphate 5-phosphatase 1 (EC 3.1.3.86) (Inositol polyphosphate-5-phosphatase D) (EC 3.1.3.56) (Inositol polyphosphate-5-phosphatase of 145 kDa) (SIP-145) (Phosphatidylinositol-4,5-bisphosphate 5-phosphatase) (EC 3.1.3.36) (SH2 domain-containing inositol 5'-phosphatase 1) (SH2 domain-containing inositol phosphatase 1) (SHIP-1) (p150Ship)
Q9ES57	MO2R1_MOUSE	Cell surface glycoprotein CD200 receptor 1 (CD200 cell surface glycoprotein receptor) (Cell surface glycoprotein OX2 receptor 1)
Q9ESD6	CKLF7_MOUSE	CKLF-like MARVEL transmembrane domain-containing protein 7 (Chemokine-like factor superfamily member 7) (LNV)
Q9ESD7	DYSF_MOUSE	Dysferlin (Dystrophy-associated fer-1-like protein) (Fer-1-like protein 1)
Q9EST4	PSMG2_MOUSE	Proteasome assembly chaperone 2 (CD40 ligand-activated specific transcript 3) (Tumor necrosis factor superfamily member 5-induced protein 1)
Q9ESW8	PGPI_MOUSE	Pyroglutamyl-peptidase 1 (EC 3.4.19.3) (5-oxoprolyl-peptidase) (Pyroglutamyl aminopeptidase I) (PAP-I) (Pyroglutamyl-peptidase I) (PGP-I) (Pyrrolidone-carboxylate peptidase)
Q9ESX5	DKC1_MOUSE	H/ACA ribonucleoprotein complex subunit DKC1 (EC 5.4.99.-) (Dyskerin) (Nopp140-associated protein of 57 kDa) (Nucleolar protein NAP57) (Nucleolar protein family A member 4) (snoRNP protein DKC1)
Q9JHK5	PLEK_MOUSE	Pleckstrin
Q9JHU4	DYHC1_MOUSE	Cytoplasmic dynein 1 heavy chain 1 (Cytoplasmic dynein heavy chain 1) (Dynein heavy chain, cytosolic)
Q9JI11	STK4_MOUSE	Serine/threonine-protein kinase 4 (EC 2.7.11.1) (Mammalian STE20-like protein kinase 1) (MST-1) (STE20-like kinase MST1) [Cleaved into: Serine/threonine-protein kinase 4 37kDa subunit (MST1/N); Serine/threonine-protein kinase 4 18kDa subunit (MST1/C)]
Q9JIF0	ANM1_MOUSE	Protein arginine N-methyltransferase 1 (EC 2.1.1.319) (Histone-arginine N-methyltransferase PRMT1)
Q9JIF7	COPB_MOUSE	Coatomer subunit beta (Beta-coat protein) (Beta-COP)
Q9JIG7	CCD22_MOUSE	Coiled-coil domain-containing protein 22
Q9JJ00	PLS1_MOUSE	Phospholipid scramblase 1 (PL scramblase 1) (Ca(2+)-dependent phospholipid scramblase 1) (Transplantability-associated protein 1) (NOR1) (TRA1)

Q9JJN5	CBPN_MOUSE	Carboxypeptidase N catalytic chain (CPN) (EC 3.4.17.3) (Carboxypeptidase N polypeptide 1) (Carboxypeptidase N small subunit)
Q9JJU8	SH3L1_MOUSE	SH3 domain-binding glutamic acid-rich-like protein
Q9JJZ2	TBA8_MOUSE	Tubulin alpha-8 chain (Alpha-tubulin 8)
Q9JKF1	IQGA1_MOUSE	Ras GTPase-activating-like protein IQGAP1
Q9JKF4	CLC6A_MOUSE	C-type lectin domain family 6 member A (C-type lectin superfamily member 10) (Dendritic cell-associated C-type lectin 2) (DC-associated C-type lectin 2) (Dectin-2)
Q9JKJ9	CP39A_MOUSE	24-hydroxycholesterol 7-alpha-hydroxylase (EC 1.14.14.26) (Cytochrome P450 39A1) (mCYP39A1) (Oxysterol 7-alpha-hydroxylase)
Q9JKV1	ADRM1_MOUSE	Proteasomal ubiquitin receptor ADRM1 (110 kDa cell membrane glycoprotein) (Gp110) (Adhesion-regulating molecule 1) (ARM-1) (Rpn13 homolog)
Q9JLJ2	AL9A1_MOUSE	4-trimethylaminobutyraldehyde dehydrogenase (TMABA-DH) (TMABADH) (EC 1.2.1.47) (Aldehyde dehydrogenase family 9 member A1) (EC 1.2.1.3)
Q9JLQ2	GIT2_MOUSE	ARF GTPase-activating protein GIT2 (ARF GAP GIT2) (Cool-interacting tyrosine-phosphorylated protein 2) (CAT-2) (CAT2) (G protein-coupled receptor kinase-interactor 2) (GRK-interacting protein 2)
Q9JLV6	PNKP_MOUSE	Bifunctional polynucleotide phosphatase/kinase (DNA 5'-kinase/3'-phosphatase) (Polynucleotide kinase-3'-phosphatase) [Includes: Polynucleotide 3'-phosphatase (EC 3.1.3.32) (2'(3')-polynucleotidase); Polynucleotide 5'-hydroxyl-kinase (EC 2.7.1.78)]
Q9JM76	ARPC3_MOUSE	Actin-related protein 2/3 complex subunit 3 (Arp2/3 complex 21 kDa subunit) (p21-ARC)
Q9JMA1	UBP14_MOUSE	Ubiquitin carboxyl-terminal hydrolase 14 (EC 3.4.19.12) (Deubiquitinating enzyme 14) (Ubiquitin thioesterase 14) (Ubiquitin-specific-processing protease 14)
Q9QUI0	RHOA_MOUSE	Transforming protein RhoA (EC 3.6.5.2)
Q9QUJ7	ACSL4_MOUSE	Long-chain-fatty-acid--CoA ligase 4 (EC 6.2.1.3) (Arachidonate--CoA ligase) (EC 6.2.1.15) (Long-chain acyl-CoA synthetase 4) (LACS 4) (mACS4)
Q9QUM4	SLAF1_MOUSE	Signaling lymphocytic activation molecule (SLAM family member 1) (CD antigen CD150)
Q9QUM9	PSA6_MOUSE	Proteasome subunit alpha type-6 (EC 3.4.25.1) (Macropain iota chain) (Multicatalytic endopeptidase complex iota chain) (Proteasome iota chain)
Q9QUR6	PPCE_MOUSE	Prolyl endopeptidase (PE) (EC 3.4.21.26) (Post-proline cleaving enzyme)
Q9QXB9	DRG2_MOUSE	Developmentally-regulated GTP-binding protein 2 (DRG-2) (Translation factor GTPase DRG2) (TRAFAC GTPase DRG2) (EC 3.6.5.-)
Q9QXK2	RAD18_MOUSE	E3 ubiquitin-protein ligase RAD18 (EC 2.3.2.27) (Postreplication repair protein RAD18) (mRAD18Sc) (RING-type E3 ubiquitin transferase RAD18)

Q9QXK3	COPG2_MOUSE	Coatomer subunit gamma-2 (Gamma-2-coat protein) (Gamma-2-COP)
Q9QXL1	KI21B_MOUSE	Kinesin-like protein KIF21B (Kinesin-like protein KIF6)
Q9QXS1	PLEC_MOUSE	Plectin (PCN) (PLTN) (Plectin-1) (Plectin-6)
Q9QXW9	LAT2_MOUSE	Large neutral amino acids transporter small subunit 2 (L-type amino acid transporter 2) (mLAT2) (Solute carrier family 7 member 8)
Q9QY06	MYO9B_MOUSE	Unconventional myosin-IXb (Unconventional myosin-9b)
Q9QYG0	NDRG2_MOUSE	Protein NDRG2 (N-myc downstream-regulated gene 2 protein) (Protein Ndr2)
Q9QYI3	DNJC7_MOUSE	DnaJ homolog subfamily C member 7 (Cytoplasmic CAR retention protein) (CCRP) (MDj11) (Tetratricopeptide repeat protein 2) (TPR repeat protein 2)
Q9QYJ0	DNJA2_MOUSE	DnaJ homolog subfamily A member 2 (mDj3)
Q9QZ88	VPS29_MOUSE	Vacuolar protein sorting-associated protein 29 (Vesicle protein sorting 29)
Q9QZB7	ARP10_MOUSE	Actin-related protein 10 (Actin-related protein 11)
Q9QZD9	EIF3I_MOUSE	Eukaryotic translation initiation factor 3 subunit I (eIF3i) (Eukaryotic translation initiation factor 3 subunit 2) (TGF-beta receptor-interacting protein 1) (TRIP-1) (eIF-3-beta) (eIF3 p36)
Q9QZE5	COPG1_MOUSE	Coatomer subunit gamma-1 (Gamma-1-coat protein) (Gamma-1-COP)
Q9QZQ8	H2AY_MOUSE	Core histone macro-H2A.1 (Histone macroH2A1) (mH2A1) (H2A.y) (H2A/y)
Q9R0E1	PLOD3_MOUSE	Multifunctional procollagen lysine hydroxylase and glycosyltransferase LH3 [Includes: Procollagen-lysine,2-oxoglutarate 5-dioxygenase 3 (EC 1.14.11.4) (Lysyl hydroxylase 3) (LH3); Procollagen glycosyltransferase (EC 2.4.1.50) (EC 2.4.1.66) (Galactosylhydroxylysine-glucosyltransferase) (Procollagen galactosyltransferase) (Procollagen glucosyltransferase)]
Q9R0K7	AT2B2_MOUSE	Plasma membrane calcium-transporting ATPase 2 (PMCA2) (EC 7.2.2.10) (Plasma membrane calcium ATPase isoform 2) (Plasma membrane calcium pump isoform 2)
Q9R0N0	GALK1_MOUSE	Galactokinase (EC 2.7.1.6) (Galactose kinase)
Q9R0P3	ESTD_MOUSE	S-formylglutathione hydrolase (FGH) (EC 3.1.2.12) (Esterase 10) (Esterase D) (Sid 478)
Q9R0P5	DEST_MOUSE	Destrin (Actin-depolymerizing factor) (ADF) (Sid 23)
Q9R0Q6	ARC1A_MOUSE	Actin-related protein 2/3 complex subunit 1A (SOP2-like protein) (Sid 329)
Q9R0Q7	TEBP_MOUSE	Prostaglandin E synthase 3 (EC 5.3.99.3) (Cytosolic prostaglandin E2 synthase) (cPGES) (Hsp90 co-chaperone) (Progesterone receptor complex p23) (Sid 3177) (Telomerase-binding protein p23)
Q9R111	GUAD_MOUSE	Guanine deaminase (Guanase) (Guanine aminase) (EC 3.5.4.3) (Guanine aminohydrolase) (GAH)

Q9R1K5	FZR1_MOUSE	Fizzy-related protein homolog (Fzr) (Cdh1/Hct1 homolog)
Q9R1P0	PSA4_MOUSE	Proteasome subunit alpha type-4 (EC 3.4.25.1) (Macropain subunit C9) (Multicatalytic endopeptidase complex subunit C9) (Proteasome component C9) (Proteasome subunit L)
Q9R1P4	PSA1_MOUSE	Proteasome subunit alpha type-1 (EC 3.4.25.1) (Macropain subunit C2) (Multicatalytic endopeptidase complex subunit C2) (Proteasome component C2) (Proteasome nu chain)
Q9R1T2	SAE1_MOUSE	SUMO-activating enzyme subunit 1 (Ubiquitin-like 1-activating enzyme E1A) [Cleaved into: SUMO-activating enzyme subunit 1, N-terminally processed]
Q9WTM5	RUVB2_MOUSE	RuvB-like 2 (EC 3.6.4.12) (p47 protein)
Q9WTR1	TRPV2_MOUSE	Transient receptor potential cation channel subfamily V member 2 (TrpV2) (Growth factor-regulated calcium channel) (GRC) (Osm-9-like TRP channel 2) (OTRPC2)
Q9WTS5	TEN2_MOUSE	Teneurin-2 (Ten-2) (Protein Odd Oz/ten-m homolog 2) (Tenascin-M2) (Ten-m2) (Teneurin transmembrane protein 2) [Cleaved into: Ten-2, soluble form; Ten-2 intracellular domain (Ten-2 ICD)]
Q9WTU6	MK09_MOUSE	Mitogen-activated protein kinase 9 (MAP kinase 9) (MAPK 9) (EC 2.7.11.24) (Stress-activated protein kinase JNK2) (c-Jun N-terminal kinase 2)
Q9WU28	PFD5_MOUSE	Prefoldin subunit 5 (EIG-1) (Myc modulator 1) (c-Myc-binding protein Mm-1)
Q9WUA2	SYFB_MOUSE	Phenylalanine--tRNA ligase beta subunit (EC 6.1.1.20) (Phenylalanyl-tRNA synthetase beta subunit) (PheRS)
Q9WUA3	PFKAP_MOUSE	ATP-dependent 6-phosphofructokinase, platelet type (ATP-PFK) (PFK-P) (EC 2.7.1.11) (6-phosphofructokinase type C) (Phosphofructo-1-kinase isozyme C) (PFK-C) (Phosphohexokinase)
Q9WUM4	COR1C_MOUSE	Coronin-1C (Coronin-3)
Q9WV27	AT1A4_MOUSE	Sodium/potassium-transporting ATPase subunit alpha-4 (Na(+)/K(+) ATPase alpha-4 subunit) (EC 7.2.2.13) (Sodium pump subunit alpha-4)
Q9WV32	ARC1B_MOUSE	Actin-related protein 2/3 complex subunit 1B (Arp2/3 complex 41 kDa subunit) (p41-ARC)
Q9WV80	SNX1_MOUSE	Sorting nexin-1
Q9WVA3	BUB3_MOUSE	Mitotic checkpoint protein BUB3 (WD repeat type I transmembrane protein A72.5)
Q9WVA4	TAGL2_MOUSE	Transgelin-2 (SM22-beta)
Q9WVE8	PACN2_MOUSE	Protein kinase C and casein kinase substrate in neurons protein 2 (Syndapin-2) (Syndapin-II) (SdpII)
Q9WVJ2	PSD13_MOUSE	26S proteasome non-ATPase regulatory subunit 13 (26S proteasome regulatory subunit RPN9) (26S proteasome regulatory subunit S11) (26S proteasome regulatory subunit p40.5)
Q9Z0J0	NPC2_MOUSE	NPC intracellular cholesterol transporter 2 (Epididymal secretory protein E1) (mE1) (Niemann Pick type C2 protein homolog)

Q9Z0L8	GGH_MOUSE	Gamma-glutamyl hydrolase (EC 3.4.19.9) (Conjugase) (FGPH) (Folylpolyglutamate hydrolase) (GH) (Gamma-Glu-x carboxypeptidase)
Q9Z0M6	CD97_MOUSE	CD97 antigen (CD antigen CD97) [Cleaved into: CD97 antigen subunit alpha; CD97 antigen subunit beta]
Q9Z0N1	IF2G_MOUSE	Eukaryotic translation initiation factor 2 subunit 3, X-linked (Eukaryotic translation initiation factor 2 subunit gamma, X-linked) (eIF-2-gamma X)
Q9Z0N2	IF2H_MOUSE	Eukaryotic translation initiation factor 2 subunit 3, Y-linked (Eukaryotic translation initiation factor 2 subunit gamma, Y-linked) (eIF-2-gamma Y) (Spermatogonial proliferation factor) (Spy)
Q9Z0P5	TWF2_MOUSE	Twinfilin-2 (A6-related protein) (mA6RP) (Twinfilin-1-like protein)
Q9Z1B7	MK13_MOUSE	Mitogen-activated protein kinase 13 (MAP kinase 13) (MAPK 13) (EC 2.7.11.24) (Mitogen-activated protein kinase p38 delta) (MAP kinase p38 delta) (Stress-activated protein kinase 4)
Q9Z1D1	EIF3G_MOUSE	Eukaryotic translation initiation factor 3 subunit G (eIF3g) (Eukaryotic translation initiation factor 3 RNA-binding subunit) (eIF-3 RNA-binding subunit) (Eukaryotic translation initiation factor 3 subunit 4) (eIF-3-delta) (eIF3 p42) (eIF3 p44)
Q9Z1N5	DX39B_MOUSE	Spliceosome RNA helicase Ddx39b (EC 3.6.4.13) (56 kDa U2AF65-associated protein) (DEAD box protein UAP56) (HLA-B-associated transcript 1 protein)
Q9Z1T1	AP3B1_MOUSE	AP-3 complex subunit beta-1 (Adaptor protein complex AP-3 subunit beta-1) (Adaptor-related protein complex 3 subunit beta-1) (Beta-3A-adaptin) (Clathrin assembly protein complex 3 beta-1 large chain)
Q9Z1Z2	STRAP_MOUSE	Serine-threonine kinase receptor-associated protein (UNR-interacting protein)
Q9Z204	HNRPC_MOUSE	Heterogeneous nuclear ribonucleoproteins C1/C2 (hnRNP C1/C2)
Q9Z2C8	YBOX2_MOUSE	Y-box-binding protein 2 (FRGY2 homolog) (Germ cell-specific Y-box-binding protein)
Q9Z2M6	UBL3_MOUSE	Ubiquitin-like protein 3 (Membrane-anchored ubiquitin-fold protein) (MUB) (MmMUB) (Protein HCG-1)
Q9Z2N8	ACL6A_MOUSE	Actin-like protein 6A (53 kDa BRG1-associated factor A) (Actin-related protein Baf53a) (BRG1-associated factor 53A) (BAF53A)
Q9Z2U0	PSA7_MOUSE	Proteasome subunit alpha type-7 (EC 3.4.25.1) (Proteasome subunit RC6-1)
Q9Z2W0	DNPEP_MOUSE	Aspartyl aminopeptidase (EC 3.4.11.21)
Q9Z2X1	HNRPF_MOUSE	Heterogeneous nuclear ribonucleoprotein F (hnRNP F) [Cleaved into: Heterogeneous nuclear ribonucleoprotein F, N-terminally processed]

Table 5. Differentially expressed proteins

Accesion	Entry name	regDCs EXO	iDCs EXO	stimDCs EXO	Protein names
P99027	RLA2_MOUSE	0.000401	0.0004846	0.00076945	60S acidic ribosomal protein P2
P48036	ANXA5_MOUSE	0.003121	0.0062391	0.0045662	Annexin A5 (Anchorin CII) (Annexin V) (Annexin-5) (Calphobindin I) (CBP-I) (Endonexin II) (Lipocortin V) (Placental anticoagulant protein 4) (PP4) (Placental anticoagulant protein I) (PAP-I) (Thromboplastin inhibitor) (Vascular anticoagulant-alpha) (VAC-alpha)
Q61233	PLSL_MOUSE	0.005398	0.0082766	0.00604266	Plastin-2 (65 kDa macrophage protein) (L-plastin) (Lymphocyte cytosolic protein 1) (LCP-1) (pp65)
Q9WVK4	EHD1_MOUSE	0.003418	0.0022469	0.00322076	EH domain-containing protein 1 (PAST homolog 1) (mPAST1)
Q01853	TERA_MOUSE	0.003543	0.0053561	0.00468531	Transitional endoplasmic reticulum ATPase (TER ATPase) (EC 3.6.4.6) (15S Mg(2+)-ATPase p97 subunit) (Valosin-containing protein) (VCP)
Q9QYB1	CLIC4_MOUSE	0.002292	0.0012486	0.00275543	Chloride intracellular channel protein 4 (mc3s5/mtCLIC)
Q9Z1Q5	CLIC1_MOUSE	0.00413	0.0026806	0.00206993	Chloride intracellular channel protein 1 (Nuclear chloride ion channel 27) (NCC27)
P11352	GPX1_MOUSE	0.001085	0.0005799	0.00114964	Glutathione peroxidase 1 (GPx-1) (GSHPx-1) (EC 1.11.1.9) (Cellular glutathione peroxidase) (Selenium-dependent glutathione peroxidase 1)
Q9D819	IPYR_MOUSE	0.000294	4.698E-05	0.00059992	Inorganic pyrophosphatase (EC 3.6.1.1) (Pyrophosphate phospho-hydrolase) (PPase)
P07356	ANXA2_MOUSE	0.006683	0.0106378	0.00770791	Annexin A2 (Annexin II) (Annexin-2) (Calpactin I heavy chain) (Calpactin-1 heavy chain) (Chromobindin-8) (Lipocortin II) (Placental anticoagulant protein IV) (PAP-IV) (Protein I) (p36)
P12815	PDCD6_MOUSE	0.000457	0.0006539	0.00033445	Programmed cell death protein 6 (ALG-257) (Apoptosis-linked

					gene 2 protein) (ALG-2) (PMP41)
Q01768	NDKB_MOUSE	0.000638	0.0013181	0.00106445	Nucleoside diphosphate kinase B (NDK B) (NDP kinase B) (EC 2.7.4.6) (Histidine protein kinase NDKB) (EC 2.7.13.3) (P18) (nm23-M2)
Q9JM14	NT5C_MOUSE	0.000173	9.655E-05	0.0002834	5'(3')-deoxyribonucleotidase, cytosolic type (EC 3.1.3.-) (Cytosolic 5',3'-pyrimidine nucleotidase) (Deoxy-5'-nucleotidase 1) (dNT-1)
Q3THE2	ML12B_MOUSE	0.000716	0.0003398	0.00075315	Myosin regulatory light chain 12B (Myosin regulatory light chain 2-B, smooth muscle isoform) (Myosin regulatory light chain 20 kDa) (MLC20) (Myosin regulatory light chain MRLC2)
P28667	MRP_MOUSE	0.0000744	5.019E-05	0.00026734	MARCKS-related protein (Brain protein F52) (MARCKS-like protein 1) (Macrophage myristoylated alanine-rich C kinase substrate) (Mac-MARCKS) (MacMARCKS)
P47753	CAZA1_MOUSE	0.000835	0.001127	0.00065097	F-actin-capping protein subunit alpha-1 (CapZ alpha-1)
O08997	ATOX1_MOUSE	0.0000204	0.0001204	0.0001002	Copper transport protein ATOX1 (Metal transport protein ATX1)
O35744	CHIL3_MOUSE	0.005243	0.0228313	0.00303876	Chitinase-like protein 3 (EC 3.2.1.52) (Beta-N-acetylhexosaminidase Ym1) (Chitinase-3-like protein 3) (ECF-L) (Eosinophil chemotactic cytokine) (Secreted protein Ym1)
P56480	ATPB_MOUSE	0.001727	0.0014836	0.00075078	ATP synthase subunit beta, mitochondrial (EC 7.1.2.2) (ATP synthase F1 subunit beta)
O70370	CATS_MOUSE	0.000398	0.0024071	0.00055035	Cathepsin S (EC 3.4.22.27)
P0DP26	CALM1_MOUSE	0.000889	0.0013658	0.00153757	Calmodulin-1
Q99KN1	ARRD1_MOUSE	0.000271	0.001257	0.00010115	Arrestin domain-containing protein 1 (Alpha-arrestin 1)
P62835	RAP1A_MOUSE	0.001876	0.0025236	0.00153698	Ras-related protein Rap-1A (Ras-related protein Krev-1)
Q9R1P1	PSB3_MOUSE	0.000454	0.0011547	0.00090137	Proteasome subunit beta type-3 (EC 3.4.25.1) (Proteasome chain 13) (Proteasome component C10-II) (Proteasome theta chain)

P10126	EF1A1_MOUSE	0.005463	0.0043816	0.00672864	Elongation factor 1-alpha 1 (EF-1-alpha-1) (Elongation factor Tu) (EF-Tu) (Eukaryotic elongation factor 1 A-1) (eEF1A-1)
P27784	CCL6_MOUSE	0.000312	0.000482	0.00013441	C-C motif chemokine 6 (Protein C10) (Small-inducible cytokine A6) [Cleaved into: CCL6(22-95); CCL6(23-95)]
P11499	HS90B_MOUSE	0.004698	0.0043947	0.00770712	Heat shock protein HSP 90-beta (Heat shock 84 kDa) (HSP 84) (HSP84) (Tumor-specific transplantation 84 kDa antigen) (TSTA)
P29477	NOS2_MOUSE	0.0000363	0.0003849	0.00440807	Nitric oxide synthase, inducible (EC 1.14.13.39) (Inducible NO synthase) (Inducible NOS) (iNOS) (Macrophage NOS) (MAC-NOS) (NOS type II) (Peptidyl-cysteine S-nitrosylase NOS2)
P34960	MMP12_MOUSE	0.009638	0.0163367	0.00398931	Macrophage metalloelastase (MME) (EC 3.4.24.65) (Matrix metalloproteinase-12) (MMP-12)
P28474	ADHX_MOUSE	0.000655	0.0002723	0.0007683	Alcohol dehydrogenase class-3 (EC 1.1.1.1) (Alcohol dehydrogenase 2) (Alcohol dehydrogenase 5) (Alcohol dehydrogenase B2) (ADH-B2) (Alcohol dehydrogenase class-III) (Glutathione-dependent formaldehyde dehydrogenase) (FALDH) (FDH) (GSH-FDH) (EC 1.1.1.-) (S-(hydroxymethyl)glutathione dehydrogenase) (EC 1.1.1.284)
P10605	CATB_MOUSE	0.000861	0.0032714	0.00087018	Cathepsin B (EC 3.4.22.1) (Cathepsin B1) [Cleaved into: Cathepsin B light chain; Cathepsin B heavy chain]
Q99PT1	GDIR1_MOUSE	0.000749	0.0011675	0.00112029	Rho GDP-dissociation inhibitor 1 (Rho GDI 1) (GDI-1) (Rho-GDI alpha)
Q9WU78	PDC6I_MOUSE	0.003605	0.0048618	0.00227039	Programmed cell death 6-interacting protein (ALG-2-interacting protein 1) (ALG-2-interacting protein X) (E2F1-inducible protein) (Eig2)
Q62159	RHOC_MOUSE	0.000867	0.0005406	0.00058548	Rho-related GTP-binding protein RhoC (Silica-induced gene 61 protein) (SIG-61)

Q3UU41	SCIMP_MOUSE	0.00036	0.0007369	0.00018377	SLP adapter and CSK-interacting membrane protein
P11835	ITB2_MOUSE	0.005301	0.0069837	0.00370523	Integrin beta-2 (Cell surface adhesion glycoproteins LFA-1/CR3/p150,95 subunit beta) (Complement receptor C3 subunit beta) (CD antigen CD18)
Q9Z2U1	PSA5_MOUSE	0.000335	0.0005812	0.00058527	Proteasome subunit alpha type-5 (EC 3.4.25.1) (Macropain zeta chain) (Multicatalytic endopeptidase complex zeta chain) (Proteasome zeta chain)
P18242	CATD_MOUSE	0.000607	0.0016186	0.00061616	Cathepsin D (EC 3.4.23.5)
Q07797	LG3BP_MOUSE	0.001838	0.0058664	0.0022212	Galectin-3-binding protein (Cyp-C-associated protein) (CyCAP) (Lectin galactoside-binding soluble 3-binding protein) (Protein MAMA)
O08992	SDCB1_MOUSE	0.00144	0.0020087	0.00075223	Syntenin-1 (Scaffold protein Pbp1) (Syndecan-binding protein 1)
P51863	VA0D1_MOUSE	0.000908	0.0005374	0.00053485	V-type proton ATPase subunit d 1 (V-ATPase subunit d 1) (P39) (Physophilin) (V-ATPase 40 kDa accessory protein) (V-ATPase AC39 subunit) (Vacuolar proton pump subunit d 1)
P17879	HS71B_MOUSE	0.001105	0.0008335	0.00167216	Heat shock 70 kDa protein 1B (Heat shock 70 kDa protein 1) (HSP70.1)
P05555	ITAM_MOUSE	0.003861	0.004726	0.00300325	Integrin alpha-M (CD11 antigen-like family member B) (CR-3 alpha chain) (Cell surface glycoprotein MAC-1 subunit alpha) (Leukocyte adhesion receptor MO1) (CD antigen CD11b)
Q61696	HS71A_MOUSE	0.001126	0.0008335	0.00162206	Heat shock 70 kDa protein 1A (Heat shock 70 kDa protein 3) (HSP70.3) (Hsp68)
Q00612	G6PD1_MOUSE	0.001009	0.000874	0.00150484	Glucose-6-phosphate 1-dehydrogenase X (G6PD) (EC 1.1.1.49)
Q9WUU7	CATZ_MOUSE	0.0000752	0.000486	0.00020022	Cathepsin Z (EC 3.4.18.1)
P20152	VIME_MOUSE	0.002159	0.0034838	0.00449668	Vimentin
Q61753	SERA_MOUSE	0.001413	0.0014482	0.0023535	D-3-phosphoglycerate dehydrogenase (3-PGDH) (EC 1.1.1.95) (A10)
Q64444	CAH4_MOUSE	0.00078	0.0001995	0.00028319	Carbonic anhydrase 4 (EC 4.2.1.1) (Carbonate dehydratase

					IV) (Carbonic anhydrase IV) (CA-IV)
P09103	PDIA1_MOUSE	0.001223	0.0020286	0.00150375	Protein disulfide-isomerase (PDI) (EC 5.3.4.1) (Cellular thyroid hormone-binding protein) (Endoplasmic reticulum resident protein 59) (ER protein 59) (ERp59) (Prolyl 4-hydroxylase subunit beta) (p55)
Q9EQH3	VPS35_MOUSE	0.001627	0.0010324	0.00158862	Vacuolar protein sorting-associated protein 35 (Maternal-embryonic 3) (Vesicle protein sorting 35)
Q99KQ4	NAMPT_MOUSE	0.000228	0.0001261	0.00066682	Nicotinamide phosphoribosyltransferase (NAmPRTase) (Nampt) (EC 2.4.2.12) (Pre-B-cell colony-enhancing factor 1 homolog) (PBEF) (Visfatin)
Q8K124	PKHO2_MOUSE	0.000589	0.0006552	0.00033463	Pleckstrin homology domain-containing family O member 2 (PH domain-containing family O member 2) (Pleckstrin homology domain-containing family Q member 1) (PH domain-containing family Q member 1)
Q9ET01	PYGL_MOUSE	0.001847	0.0008386	0.00266863	Glycogen phosphorylase, liver form (EC 2.4.1.1)
Q99KC8	VMA5A_MOUSE	0.001022	0.0016045	0.00160166	von Willebrand factor A domain-containing protein 5A (Loss of heterozygosity 11 chromosomal region 2 gene A protein homolog)
O08709	PRDX6_MOUSE	0.000569	0.0001493	0.00048327	Peroxiredoxin-6 (EC 1.11.1.15) (1-Cys peroxiredoxin) (1-Cys PRX) (Acidic calcium-independent phospholipase A2) (aiPLA2) (EC 3.1.1.4) (Antioxidant protein 2) (Non-selenium glutathione peroxidase) (NSGPx)
Q9QXH4	ITAX_MOUSE	0.002733	0.0030694	0.00093431	Integrin alpha-X (CD11 antigen-like family member C) (Leukocyte adhesion glycoprotein p150,95 alpha chain) (Leukocyte adhesion receptor p150,95) (CD antigen CD11c)
Q99K85	SERC_MOUSE	0.000227	9.976E-05	0.00058554	Phosphoserine aminotransferase (PSAT) (EC 2.6.1.52) (Endometrial

					progesterone-induced protein) (EPIP) (Phosphohydroxythreonine aminotransferase)
Q9Z1E4	GYS1_MOUSE	0.000211	4.698E-05	0.00103528	Glycogen [starch] synthase, muscle (EC 2.4.1.11)
P97449	AMPN_MOUSE	0.004186	0.0054894	0.00258201	Aminopeptidase N (AP-N) (mAPN) (EC 3.4.11.2) (Alanyl aminopeptidase) (Aminopeptidase M) (AP-M) (Membrane protein p161) (Microsomal aminopeptidase) (CD antigen CD13)
Q61207	SAP_MOUSE	0.001428	0.0041912	0.00074965	Prosaposin (Sulfated glycoprotein 1) (SGP-1) [Cleaved into: Saposin-A; Saposin-B-Val; Saposin-B; Saposin-C; Saposin-D]
Q03265	ATPA_MOUSE	0.001264	0.0004834	0.00033276	ATP synthase subunit alpha, mitochondrial (ATP synthase F1 subunit alpha)
P12382	PFKAL_MOUSE	0.001213	0.000997	0.00205223	ATP-dependent 6-phosphofructokinase, liver type (ATP-PFK) (PFK-L) (EC 2.7.1.11) (6-phosphofructokinase type B) (Phosphofructo-1-kinase isozyme B) (PFK-B) (Phosphohexokinase)
P21956	MFGM_MOUSE	0.001617	0.0022127	0.00029968	Lactadherin (MFGM) (Milk fat globule-EGF factor 8) (MFG-E8) (SED1) (Sperm surface protein SP47) (MP47)
P00920	CAH2_MOUSE	0.0000548	0.0003926	0.00036637	Carbonic anhydrase 2 (EC 4.2.1.1) (Carbonate dehydratase II) (Carbonic anhydrase II) (CA-II)
Q9DBG6	RPN2_MOUSE	0.000432	9.397E-05	0.00020135	Dolichyl-diphosphooligosaccharide--protein glycosyltransferase subunit 2 (Dolichyl-diphosphooligosaccharide--protein glycosyltransferase 63 kDa subunit) (Ribophorin II) (RPN-II) (Ribophorin-2)
P97369	NCF4_MOUSE	0.000208	7.337E-05	0.00020156	Neutrophil cytosol factor 4 (NCF-4) (Neutrophil NADPH oxidase factor 4) (p40-phox) (p40phox)
P16110	LEG3_MOUSE	0.001763	0.0029103	0.00183421	Galectin-3 (Gal-3) (35 kDa lectin) (Carbohydrate-binding protein 35) (CBP 35) (Galactose-specific lectin 3)

					(IgE-binding protein) (L-34 galactoside-binding lectin) (Laminin-binding protein) (Lectin L-29) (Mac-2 antigen)
Q64105	SPRE_MOUSE	0.000238	4.636E-05	0.00029985	Sepiapterin reductase (SPR) (EC 1.1.1.153)
P51881	ADT2_MOUSE	0.000605	0.0004402	0.00021685	ADP/ATP translocase 2 (ADP,ATP carrier protein 2) (Adenine nucleotide translocator 2) (ANT 2) (Solute carrier family 25 member 5) [Cleaved into: ADP/ATP translocase 2, N-terminally processed]
P12970	RL7A_MOUSE	0.000926	0.0008515	0.00048531	60S ribosomal protein L7a (Surfeit locus protein 3)
Q9Z1Q9	SYVC_MOUSE	0.00133	0.0008206	0.00157178	Valine--tRNA ligase (EC 6.1.1.9) (Protein G7a) (Valyl-tRNA synthetase) (ValRS)
P63321	RALA_MOUSE	0.000264	0.0001236	0.00026656	Ras-related protein Ral-A
P35441	TSP1_MOUSE	0.000328	0.0020667	0.00165218	Thrombospondin-1 (Glycoprotein G)
Q9D7G0	PRPS1_MOUSE	0.000212	4.957E-05	0.0002004	Ribose-phosphate pyrophosphokinase 1 (EC 2.7.6.1) (Phosphoribosyl pyrophosphate synthase I) (PRS-I)
Q9JIW9	RALB_MOUSE	0.000211	7.596E-05	0.00030021	Ras-related protein Ral-B
Q9JII6	AK1A1_MOUSE	0.00031	0.0008071	0.00098366	Aldo-keto reductase family 1 member A1 (EC 1.1.1.2) (EC 1.1.1.33) (EC 1.1.1.372) (EC 1.1.1.54) (Alcohol dehydrogenase [NADP(+)]) (Aldehyde reductase) (Glucuronate reductase) (EC 1.1.1.19) (Glucuronolactone reductase) (EC 1.1.1.20)
Q8BGQ7	SYAC_MOUSE	0.001176	0.000811	0.00158485	Alanine--tRNA ligase, cytoplasmic (EC 6.1.1.7) (Alanyl-tRNA synthetase) (AlaRS) (Protein sticky) (Sti)
Q08857	CD36_MOUSE	0.002037	0.0032128	0.0012868	Platelet glycoprotein 4 (Glycoprotein IIIb) (GPIIIB) (PAS IV) (PAS-4) (Platelet glycoprotein IV) (GPIV) (CD antigen CD36)
Q9WTI7	MYO1C_MOUSE	0.001363	0.0007801	0.00123398	Unconventional myosin-Ic (Myosin I beta) (MMI-beta) (MMIb)
O35639	ANXA3_MOUSE	0.000621	0.0010974	0.00068532	Annexin A3 (35-alpha calcimedin) (Annexin III) (Annexin-3) (Lipocortin III)

					(Placental anticoagulant protein III) (PAP-III)
P39054	DYN2_MOUSE	0.000712	0.0004152	0.00091771	Dynamin-2 (EC 3.6.5.5) (Dynamin UDNM)
P62983	RS27A_MOUSE	0.001332	0.0016727	0.00075258	Ubiquitin-40S ribosomal protein S27a (Ubiquitin carboxyl extension protein 80) [Cleaved into: Ubiquitin; 40S ribosomal protein S27a]
Q3THS6	METK2_MOUSE	0.000244	0.0001204	0.00031761	S-adenosylmethionine synthase isoform type-2 (AdoMet synthase 2) (EC 2.5.1.6) (Methionine adenosyltransferase 2) (MAT 2)
P19973	LSP1_MOUSE	0.000265	0.0005168	0.00058456	Lymphocyte-specific protein 1 (52 kDa phosphoprotein) (pp52) (Lymphocyte-specific antigen WP34) (S37 protein)
P35762	CD81_MOUSE	0.000454	0.000634	0.00034957	CD81 antigen (26 kDa cell surface protein TAPA-1) (Target of the antiproliferative antibody 1) (CD antigen CD81)
Q8BT60	CPNE3_MOUSE	0.000843	0.0004318	0.00063315	Copine-3 (Copine III)
Q91Z98	CHIL4_MOUSE	0.002071	0.005536	0.00121877	Chitinase-like protein 4 (Chitinase-3-like protein 4) (Secreted protein Ym2)
Q91YP3	DEOC_MOUSE	0.000155	0.0002929	8.3955E-05	Deoxyribose-phosphate aldolase (DERA) (EC 4.1.2.4) (2-deoxy-D-ribose 5-phosphate aldolase) (Phosphodeoxyriboaldolase) (Deoxyriboaldolase)
P42669	PURA_MOUSE	0.000284	2.318E-05	0.00011665	Transcriptional activator protein Pur-alpha (Purine-rich single-stranded DNA-binding protein alpha)
Q9WTP6	KAD2_MOUSE	0.0000903	0.0004106	0.00039988	Adenylate kinase 2, mitochondrial (AK 2) (EC 2.7.4.3) (ATP-AMP transphosphorylase 2) (ATP:AMP phosphotransferase) (Adenylate monophosphate kinase)
P10649	GSTM1_MOUSE	0.000131	4.698E-05	0.00011647	Glutathione S-transferase Mu 1 (EC 2.5.1.18) (GST 1-1) (GST class-mu 1) (Glutathione S-transferase GT8.7) (pmGT10)
P05063	ALDOC_MOUSE	0.000527	0.0003411	0.00085017	Fructose-bisphosphate aldolase C (EC 4.1.2.13) (Aldolase 3) (Brain-type aldolase) (Scrapie-

					responsive protein 2) (Zebrin II)
Q9WUL5	PD1L2_MOUSE	0.000518	0.0009236	0.00033445	Programmed cell death 1 ligand 2 (PD-1 ligand 2) (PD-L2) (PDCD1 ligand 2) (Programmed death ligand 2) (Butyrophilin B7-DC) (B7-DC) (CD antigen CD273)
Q8R180	ERO1A_MOUSE	0.000212	0.0001467	0.00048418	ERO1-like protein alpha (ERO1-L) (ERO1-L-alpha) (EC 1.8.4.-) (Endoplasmic reticulum oxidoreductase alpha) (Endoplasmic reticulum oxidoreductin-1-like protein) (Oxidoreductin-1-L-alpha)
Q9CQW9	IFM3_MOUSE	0.000155	0.0008129	0.00051783	Interferon-induced transmembrane protein 3 (Dispanin subfamily A member 2b) (DSPA2b) (Fragilis protein) (Interferon-inducible protein 15) (Mouse ifitm-like protein 1) (Mil-1)
Q64324	STXB2_MOUSE	0.000448	0.000168	0.00049972	Syntaxin-binding protein 2 (MUSEC1) (Protein unc-18 homolog 2) (Munc18-2) (Unc18-2) (Protein unc-18 homolog B) (Unc-18B)
P07724	ALBU_MOUSE	0.000623	0.0016998	0.00044775	Serum albumin
Q61093	CY24B_MOUSE	0.000945	0.0004898	0.00101861	Cytochrome b-245 heavy chain (EC 1.-.-.-) (CGD91-phox) (Cytochrome b(558) subunit beta) (Cytochrome b558 subunit beta) (Heme-binding membrane glycoprotein gp91phox) (Neutrophil cytochrome b 91 kDa polypeptide) (gp91-1) (gp91-phox) (p22 phagocyte B-cytochrome)
Q9JKR6	HYOU1_MOUSE	0.000796	0.00041	0.00083174	Hypoxia up-regulated protein 1 (GRP-170) (140 kDa Ca(2+)-binding protein) (CBP-140)
Q78PY7	SND1_MOUSE	0.000791	0.0002477	0.00093772	Staphylococcal nuclease domain-containing protein 1 (EC 3.1.31.1) (100 kDa coactivator) (p100 co-activator)
P01831	THY1_MOUSE	0.000716	0.0010034	0.00048398	Thy-1 membrane glycoprotein (Thy-1 antigen) (CD antigen CD90)
O09043	NAPSA_MOUSE	0.000173	0.0008753	0.00013346	Napsin-A (EC 3.4.23.-) (KDAP-1) (Kidney-derived

					aspartic protease-like protein) (KAP)
Q6WVG3	KCD12_MOUSE	0.000191	7.275E-05	0.00028322	BTB/POZ domain-containing protein KCTD12 (Pfetin) (Predominantly fetal expressed T1 domain)
O88958	GNPI1_MOUSE	0.0000204	0.0001731	6.7116E-05	Glucosamine-6-phosphate isomerase 1 (EC 3.5.99.6) (Glucosamine-6-phosphate deaminase 1) (GNPDA 1) (GlcN6P deaminase 1) (Oscillin)
Q8R1F1	NIBA2_MOUSE	0.000634	7.916E-05	0.0006175	Protein Niban 2 (Meg-3) (Niban-like protein 1) (Protein FAM129B)
P06745	G6PI_MOUSE	0.000768	0.0011656	0.00131678	Glucose-6-phosphate isomerase (GPI) (EC 5.3.1.9) (Autocrine motility factor) (AMF) (Neuroleukin) (NLK) (Phosphoglucose isomerase) (PGI) (Phosphohexose isomerase) (PHI)
P62827	RAN_MOUSE	0.000711	0.0004428	0.00046809	GTP-binding nuclear protein Ran (GTPase Ran) (Ras-like protein TC4) (Ras-related nuclear protein)
Q9WUM3	COR1B_MOUSE	0.000694	0.0003894	0.00078454	Coronin-1B (Coronin-2)
Q8VCT3	AMPB_MOUSE	0.000645	0.0001931	0.00056497	Aminopeptidase B (AP-B) (EC 3.4.11.6) (Arginine aminopeptidase) (Arginyl aminopeptidase) (Cytosol aminopeptidase IV)
Q99KI0	ACON_MOUSE	0.000548	0.0001976	0.00016583	Aconitate hydratase, mitochondrial (Aconitase) (EC 4.2.1.3) (Citrate hydro-lyase)
P01942	HBA_MOUSE	0.000657	0.0006584	0.00034974	Hemoglobin subunit alpha (Alpha-globin) (Hemoglobin alpha chain)
P63325	RS10_MOUSE	0.000545	0.0001229	0.00048454	40S ribosomal protein S10
P11152	LIPL_MOUSE	0.001417	0.0033113	0.00088572	Lipoprotein lipase (LPL) (EC 3.1.1.34)
Q9JMH6	TRXR1_MOUSE	0.000152	9.717E-05	0.00041781	Thioredoxin reductase 1, cytoplasmic (TR) (EC 1.8.1.9) (Thioredoxin reductase TR1)
P28063	PSB8_MOUSE	0.000225	0.0005374	0.0002834	Proteasome subunit beta type-8 (EC 3.4.25.1) (Low molecular mass protein 7) (Macropain subunit C13) (Multicatalytic endopeptidase complex subunit C13) (Proteasome component C13) (Proteasome subunit beta-5i)

Q64737	PUR2_MOUSE	0.000438	0.0001963	0.00053372	Trifunctional purine biosynthetic protein adenosine-3 [Includes: Phosphoribosylamine--glycine ligase (EC 6.3.4.13) (Glycinamide ribonucleotide synthetase) (GARS) (Phosphoribosylglycinamide synthetase); Phosphoribosylformylglycinamidine cyclo-ligase (EC 6.3.3.1) (AIR synthase) (AIRS) (Phosphoribosyl-aminoimidazole synthetase); Phosphoribosylglycinamide formyltransferase (EC 2.1.2.2) (5'-phosphoribosylglycinamide transformylase) (GAR transformylase) (GART)]
P01027	CO3_MOUSE	0.000602	0.0036248	0.00049895	Complement C3 (HSE-MSF) [Cleaved into: Complement C3 beta chain; C3-beta-c (C3bc); Complement C3 alpha chain; C3a anaphylatoxin; Acylation stimulating protein (ASP) (C3adesArg); Complement C3b alpha' chain; Complement C3c alpha' chain fragment 1; Complement C3dg fragment; Complement C3g fragment; Complement C3d fragment; Complement C3f fragment; Complement C3c alpha' chain fragment 2]
P63158	HMGB1_MOUSE	0.000606	0.0001474	0.0001832	High mobility group protein B1 (High mobility group protein 1) (HMG-1)
P17225	PTBP1_MOUSE	0.000252	9.593E-05	0.00035034	Polypyrimidine tract-binding protein 1 (PTB) (Heterogeneous nuclear ribonucleoprotein I) (hnRNP I)
O70194	EIF3D_MOUSE	0.000438	0.0001229	0.00031574	Eukaryotic translation initiation factor 3 subunit D (eIF3d) (Eukaryotic translation initiation factor 3 subunit 7) (eIF-3-zeta) (eIF3 p66)
Q9R1P3	PSB2_MOUSE	0.000211	0.000585	0.00023348	Proteasome subunit beta type-2 (EC 3.4.25.1) (Macropain subunit C7-I) (Multicatalytic endopeptidase complex subunit C7-I) (Proteasome component C7-I)
Q9JJ28	FLII_MOUSE	0.000582	0.00039	0.00060101	Protein flightless-1 homolog

Q8CGC7	SYEP_MOUSE	0.00078	0.0005207	0.00118371	Bifunctional glutamate/proline--tRNA ligase (Bifunctional aminoacyl-tRNA synthetase) [Includes: Glutamate--tRNA ligase (EC 6.1.1.17) (Glutamyl-tRNA synthetase) (GluRS); Proline--tRNA ligase (EC 6.1.1.15) (Prolyl-tRNA synthetase) (ProRS)]
Q9R1T4	SEPT6_MOUSE	0.000169	4.698E-05	0.00025014	Septin-6
Q9Z1F9	SAE2_MOUSE	0.000135	0.0001429	0.00041707	SUMO-activating enzyme subunit 2 (EC 2.3.2.-) (Anthracycline-associated resistance ARX) (Ubiquitin-like 1-activating enzyme E1B) (Ubiquitin-like modifier-activating enzyme 2)
P48025	KSYK_MOUSE	0.000267	0.0002465	0.00038434	Tyrosine-protein kinase SYK (EC 2.7.10.2) (Spleen tyrosine kinase)
Q9R233	TPSN_MOUSE	0.000268	0.0001229	0.0004163	Tapasin (TPN) (TPSN) (TAP-associated protein) (TAP-binding protein)
P28650	PURA1_MOUSE	0.000397	4.636E-05	0.0002524	Adenylosuccinate synthetase isozyme 1 (AMPSase 1) (AdSS 1) (EC 6.3.4.4) (Adenylosuccinate synthetase like 1) (Adenylosuccinate synthetase, basic isozyme) (Adenylosuccinate synthetase, muscle isozyme) (M-type adenylosuccinate synthetase) (IMP--aspartate ligase 1)
P55065	PLTP_MOUSE	0.000405	0.0015684	3.3469E-05	Phospholipid transfer protein (Lipid transfer protein II)
P47226	TES_MOUSE	0.000188	7.337E-05	0.00030137	Testin (TES1/TES2)
Q9CXW4	RL11_MOUSE	0.000414	0.000417	0.00025088	60S ribosomal protein L11
Q07076	ANXA7_MOUSE	0.00042	0.0008347	0.00051519	Annexin A7 (Annexin VII) (Annexin-7) (Synexin)
Q61490	CD166_MOUSE	0.000299	7.337E-05	0.0003849	CD166 antigen (Activated leukocyte cell adhesion molecule) (BEN) (Protein DM-GRASP) (CD antigen CD166)
Q91XB0	TREX1_MOUSE	0.0000389	7.275E-05	0.00030042	Three-prime repair exonuclease 1 (EC 3.1.11.2) (3'-5' exonuclease TREX1)
P17809	GTR1_MOUSE	0.000531	0.0001461	0.00084887	Solute carrier family 2, facilitated glucose transporter member 1 (Glucose transporter type 1, erythrocyte/brain) (GLUT-1) (GT1)

O35598	ADA10_MOUSE	0.000534	0.0005046	4.9922E-05	Disintegrin and metalloproteinase domain-containing protein 10 (ADAM 10) (EC 3.4.24.81) (Kuzbanian protein homolog) (Mammalian disintegrin-metalloprotease) (CD antigen CD156c)
Q61024	ASNS_MOUSE	0.000551	0.0004628	0.0010005	Asparagine synthetase [glutamine-hydrolyzing] (EC 6.3.5.4) (Glutamine-dependent asparagine synthetase)
P10404	ENV1_MOUSE	0.000679	0.0008328	0.00046773	MLV-related proviral Env polyprotein [Cleaved into: Surface protein (SU); Transmembrane protein (TM)]
Q9CZ30	OLA1_MOUSE	0.000264	0.0001989	0.0004664	Obg-like ATPase 1 (GTP-binding protein 9)
Q9D8E6	RL4_MOUSE	0.000916	0.0004892	0.00083446	60S ribosomal protein L4
P62482	KCAB2_MOUSE	0.000173	9.717E-05	0.00018394	Voltage-gated potassium channel subunit beta-2 (EC 1.1.1.-) (K(+) channel subunit beta-2) (Kv-beta-2) (Neuroimmune protein F5)
P10853	H2B1F_MOUSE	0.016227	0.0107589	0.00784009	Histone H2B type 1-F/J/L (H2B 291A)
Q3UM45	PP1R7_MOUSE	0.000391	6.954E-05	0.00025029	Protein phosphatase 1 regulatory subunit 7 (Protein phosphatase 1 regulatory subunit 22)
Q9QZ08	NAGK_MOUSE	0.0000548	4.698E-05	0.00025088	N-acetyl-D-glucosamine kinase (N-acetylglucosamine kinase) (EC 2.7.1.59) (GlcNAc kinase)
Q9ERK4	XPO2_MOUSE	0.00263	0.0009396	0.00100424	Exportin-2 (Exp2) (Chromosome segregation 1-like protein) (Importin-alpha re-exporter)
P26369	U2AF2_MOUSE	0.000194	0.000121	0.00030042	Splicing factor U2AF 65 kDa subunit (U2 auxiliary factor 65 kDa subunit) (U2 snRNP auxiliary factor large subunit)
P61290	PSME3_MOUSE	0.000132	4.698E-05	0.00011721	Proteasome activator complex subunit 3 (11S regulator complex subunit gamma) (REG-gamma) (Activator of multicatalytic protease subunit 3) (Ki nuclear autoantigen) (Proteasome activator 28 subunit gamma) (PA28g) (PA28gamma)
Q9WTK5	NFKB2_MOUSE	0.0000592	5.278E-05	0.00028322	Nuclear factor NF-kappa-B p100 subunit (DNA-binding factor KBF2) (Nuclear factor of

					kappa light polypeptide gene enhancer in B-cells 2) [Cleaved into: Nuclear factor NF-kappa-B p52 subunit]
P08207	S10AA_MOUSE	0.000159	7.016E-05	0.0002834	Protein S100-A10 (Calpactin I light chain) (Calpactin-1 light chain) (Cellular ligand of annexin II) (S100 calcium-binding protein A10) (p10 protein) (p11)
P38647	GRP75_MOUSE	0.000375	5.019E-05	8.2827E-05	Stress-70 protein, mitochondrial (75 kDa glucose-regulated protein) (GRP-75) (Heat shock 70 kDa protein 9) (Mortalin) (Peptide-binding protein 74) (PBP74) (p66 MOT)
Q6SJQ7	CLM1_MOUSE	0.000355	2.639E-05	0.00011644	CMRF35-like molecule 1 (CLM-1) (CD300 antigen-like family member F) (Leukocyte mono-Ig-like receptor 3) (Myeloid-associated immunoglobulin-like receptor 5) (MAIR-5) (MAIR-V) (CD antigen CD300f)
Q80WQ2	VAC14_MOUSE	0.000374	0.0001204	0.00033481	Protein VAC14 homolog
Q9Z1G3	VATC1_MOUSE	0.000172	2.318E-05	0.00010041	V-type proton ATPase subunit C 1 (V-ATPase subunit C 1) (Vacuolar proton pump subunit C 1)
P46664	PURA2_MOUSE	0.000173	0.0002201	0.0004182	Adenylosuccinate synthetase isozyme 2 (AMPSase 2) (AdSS 2) (EC 6.3.4.4) (Adenylosuccinate synthetase, acidic isozyme) (Adenylosuccinate synthetase, liver isozyme) (L-type adenylosuccinate synthetase) (IMP--aspartate ligase 2)
Q61187	TS101_MOUSE	0.000132	0.0003662	5.0485E-05	Tumor susceptibility gene 101 protein (ESCRT-I complex subunit TSG101)
Q91VW3	SH3L3_MOUSE	0.000243	0.000466	0.00023327	SH3 domain-binding glutamic acid-rich-like protein 3
Q64514	TPP2_MOUSE	0.000772	0.0008277	0.00035052	Tripeptidyl-peptidase 2 (TPP-2) (EC 3.4.14.10) (Tripeptidyl aminopeptidase) (Tripeptidyl-peptidase II) (TPP-II)
P62748	HPCL1_MOUSE	0.000115	2.318E-05	0.00020057	Hippocalcin-like protein 1 (Neural visinin-like protein 3) (NVL-3) (NVP-3) (Visinin-like protein 3) (VILIP-3)

O88456	CPNS1_MOUSE	0.000127	2.318E-05	0.00015086	Calpain small subunit 1 (CSS1) (Calcium-activated neutral proteinase small subunit) (CANP small subunit) (Calcium-dependent protease small subunit) (CDPS) (Calcium-dependent protease small subunit 1) (Calpain regulatory subunit)
Q8CBW3	ABI1_MOUSE	0.000176	2.639E-05	0.00031669	Abl interactor 1 (Abelson interactor 1) (Abi-1) (Ablphilin-1) (Eps8 SH3 domain-binding protein) (Eps8-binding protein) (Spectrin SH3 domain-binding protein 1) (e3B1)
Q9Z0E6	GBP2_MOUSE	0.000248	0.0007833	0.00120137	Guanylate-binding protein 2 (EC 3.6.5.-) (GTP-binding protein 2) (GBP-2) (mGBP-2) (mGBP2) (Guanine nucleotide-binding protein 2) (Interferon-induced guanylate-binding protein 2)
Q9D1J1	NECP2_MOUSE	0.000151	4.698E-05	0.00020078	Adaptin ear-binding coat-associated protein 2 (NECAP endocytosis-associated protein 2) (NECAP-2)
P18581	CTR2_MOUSE	0.002676	0.0006983	0.00108626	Cationic amino acid transporter 2 (CAT-2) (CAT2) (20.5) (Low affinity cationic amino acid transporter 2) (Solute carrier family 7 member 2) (T-cell early activation protein) (TEA)
Q6P1F6	2ABA_MOUSE	0.000316	0.0002935	0.0005012	Serine/threonine-protein phosphatase 2A 55 kDa regulatory subunit B alpha isoform (PP2A subunit B isoform B55-alpha) (PP2A subunit B isoform PR55-alpha) (PP2A subunit B isoform R2-alpha) (PP2A subunit B isoform alpha)
Q62418	DBNL_MOUSE	0.000379	9.976E-05	0.00031705	Drebrin-like protein (Actin-binding protein 1) (SH3 domain-containing protein 7)
Q922H4	GMPPA_MOUSE	0.000148	0.0001467	0.00031761	Mannose-1-phosphate guanyltransferase alpha (GDP-mannose pyrophosphorylase A) (GTP-mannose-1-phosphate guanylyltransferase alpha)
Q02248	CTNB1_MOUSE	0.00034	0.0001461	8.3923E-05	Catenin beta-1 (Beta-catenin)

Q922D8	C1TC_MOUSE	0.000248	7.016E-05	0.00024955	C-1-tetrahydrofolate synthase, cytoplasmic (C1-THF synthase) [Cleaved into: C-1-tetrahydrofolate synthase, cytoplasmic, N-terminally processed] [Includes: Methylenetetrahydrofolate dehydrogenase (EC 1.5.1.5); Methenyltetrahydrofolate cyclohydrolase (EC 3.5.4.9); Formyltetrahydrofolate synthetase (EC 6.3.4.3)]
Q62095	DDX3Y_MOUSE	0.000327	9.976E-05	0.00030098	ATP-dependent RNA helicase DDX3Y (EC 3.6.4.13) (D1Pas1-related sequence 1) (DEAD box protein 3, Y-chromosomal) (DEAD-box RNA helicase DEAD2) (mDEAD2)
Q9JL26	FMNL1_MOUSE	0.000538	7.079E-05	0.00038342	Formin-like protein 1 (Formin-related protein)
Q6PIC6	AT1A3_MOUSE	0.001373	0.0014127	0.00086716	Sodium/potassium-transporting ATPase subunit alpha-3 (Na(+)/K(+) ATPase alpha-3 subunit) (EC 7.2.2.13) (Na(+)/K(+) ATPase alpha(III) subunit) (Sodium pump subunit alpha-3)
Q62167	DDX3X_MOUSE	0.000437	0.0001467	0.00043409	ATP-dependent RNA helicase DDX3X (EC 3.6.4.13) (D1Pas1-related sequence 2) (DEAD box RNA helicase DEAD3) (mDEAD3) (DEAD box protein 3, X-chromosomal) (Embryonic RNA helicase)
O70145	NCF2_MOUSE	0.000247	7.275E-05	0.00016714	Neutrophil cytosol factor 2 (NCF-2) (67 kDa neutrophil oxidase factor) (NADPH oxidase activator 2) (Neutrophil NADPH oxidase factor 2) (p67-phox)
Q925E7	2ABD_MOUSE	0.000281	0.0002909	0.00048475	Serine/threonine-protein phosphatase 2A 55 kDa regulatory subunit B delta isoform (PP2A subunit B isoform B55-delta) (PP2A subunit B isoform PR55-delta) (PP2A subunit B isoform R2-delta) (PP2A subunit B isoform delta)
Q64281	LIRB4_MOUSE	0.000265	0.0001699	0.0002172	Leukocyte immunoglobulin-like receptor subfamily B member 4 (Mast cell surface

					glycoprotein Gp49B) (CD antigen CD85k)
P97855	G3BP1_MOUSE	0.000188	0.0001229	0.00024993	Ras GTPase-activating protein-binding protein 1 (G3BP-1) (EC 3.6.4.12) (EC 3.6.4.13) (ATP-dependent DNA helicase VIII) (GAP SH3 domain-binding protein 1) (HDH-VIII)
Q5XJY5	COPD_MOUSE	0.000187	7.016E-05	0.0004337	Coatomer subunit delta (Archain) (Delta-coat protein) (Delta-COP)
P11276	FINC_MOUSE	0.001764	0.0011012	9.9844E-05	Fibronectin (FN) [Cleaved into: Anastellin]
P47911	RL6_MOUSE	0.001052	0.0006899	0.00086606	60S ribosomal protein L6 (TAX-responsive enhancer element-binding protein 107) (TAXREB107)
O09046	OXLA_MOUSE	0.0000796	0.0003926	0.00010076	L-amino-acid oxidase (LAAO) (LAO) (EC 1.4.3.2) (Interleukin-4-induced protein 1) (IL4-induced protein 1) (Protein Fig-1) (mFIG1)
P48999	LOX5_MOUSE	0.000531	0.0001673	5.1049E-05	Arachidonate 5-lipoxygenase (5-LO) (5-lipoxygenase) (EC 1.13.11.34)
Q9Z1G4	VPP1_MOUSE	0.000285	5.019E-05	0.00013367	V-type proton ATPase 116 kDa subunit a isoform 1 (V-ATPase 116 kDa isoform a1) (Clathrin-coated vesicle/synaptic vesicle proton pump 116 kDa subunit) (Vacuolar adenosine triphosphatase subunit Ac116) (Vacuolar proton pump subunit 1) (Vacuolar proton translocating ATPase 116 kDa subunit a isoform 1)
Q3THK7	GUAA_MOUSE	0.000133	7.016E-05	0.00026808	GMP synthase [glutamine-hydrolyzing] (EC 6.3.5.2) (GMP synthetase) (Glutamine amidotransferase)
Q60864	STIP1_MOUSE	0.000264	0.0001435	0.00041689	Stress-induced-phosphoprotein 1 (STI1) (mSTI1) (Hsc70/Hsp90-organizing protein) (Hop)
O70131	NINJ1_MOUSE	0.0000541	2.318E-05	0.0001503	Ninjurin-1 (Nerve injury-induced protein 1)
P70452	STX4_MOUSE	0.0000929	0.0002677	0.00011609	Syntaxin-4
P11688	ITA5_MOUSE	0.00032	0.0001435	0.00043483	Integrin alpha-5 (CD49 antigen-like family member E) (Fibronectin receptor subunit alpha) (Integrin alpha-F) (VLA-5) (CD antigen CD49e)

					[Cleaved into: Integrin alpha-5 heavy chain; Integrin alpha-5 light chain]
Q8BMS1	ECHA_MOUSE	0.000188	2.639E-05	4.9922E-05	Trifunctional enzyme subunit alpha, mitochondrial (Monolysocardiolipin acyltransferase) (EC 2.3.1.-) (TP-alpha) [Includes: Long-chain enoyl-CoA hydratase (EC 4.2.1.17); Long chain 3-hydroxyacyl-CoA dehydrogenase (EC 1.1.1.211)]
Q04750	TOP1_MOUSE	0.000474	0.0002903	0.00011591	DNA topoisomerase 1 (EC 5.6.2.1) (DNA topoisomerase I)
P51912	AAAT_MOUSE	0.000634	0.0006127	5.0663E-05	Neutral amino acid transporter B(0) (ATB(0)) (ASC-like Na(+)-dependent neutral amino acid transporter ASCT2) (Insulin-activated amino acid transporter) (Sodium-dependent neutral amino acid transporter type 2) (Solute carrier family 1 member 5)
P97821	CATC_MOUSE	0.000281	0.0004113	0.00013479	Dipeptidyl peptidase 1 (EC 3.4.14.1) (Cathepsin C) (Cathepsin J) (Dipeptidyl peptidase I) (DPP-I) (DPPI) (Dipeptidyl transferase) [Cleaved into: Dipeptidyl peptidase 1 exclusion domain chain (Dipeptidyl peptidase I exclusion domain chain); Dipeptidyl peptidase 1 heavy chain (Dipeptidyl peptidase I heavy chain); Dipeptidyl peptidase 1 light chain (Dipeptidyl peptidase I light chain)]
Q6PDI5	ECM29_MOUSE	0.000283	7.275E-05	0.00033427	Proteasome adapter and scaffold protein ECM29 (Proteasome-associated protein ECM29 homolog)
P62918	RL8_MOUSE	0.0000567	2.318E-05	0.00011665	60S ribosomal protein L8
Q8R3D1	TBC13_MOUSE	0.000113	4.957E-05	0.00016731	TBC1 domain family member 13
Q9EST5	AN32B_MOUSE	0.0000967	2.318E-05	0.00019983	Acidic leucine-rich nuclear phosphoprotein 32 family member B (Proliferation-related acidic leucine-rich protein PAL31)
Q9Z130	HNRDL_MOUSE	0.000225	0.0001731	0.00040062	Heterogeneous nuclear ribonucleoprotein D-like

					(hnRNP D-like) (hnRNP DL) (JKT41-binding protein)
P40240	CD9_MOUSE	0.001379	0.0015208	0.00098402	CD9 antigen (CD antigen CD9)
P60670	NPL4_MOUSE	0.0000592	2.318E-05	0.00018377	Nuclear protein localization protein 4 homolog (Protein NPL4)
Q60931	VDAC3_MOUSE	0.000211	0.0001931	1.7016E-05	Voltage-dependent anion-selective channel protein 3 (VDAC-3) (mVDAC3) (Outer mitochondrial membrane protein porin 3)
Q8CIH5	PLCG2_MOUSE	0.000247	9.655E-05	0.00030042	1-phosphatidylinositol 4,5-bisphosphate phosphodiesterase gamma-2 (EC 3.1.4.11) (Phosphoinositide phospholipase C-gamma-2) (Phospholipase C-gamma-2) (PLC-gamma-2)
P60867	RS20_MOUSE	0.000262	2.318E-05	0.00030003	40S ribosomal protein S20
Q9JIZ9	PLS3_MOUSE	0.000244	0.0002401	1.663E-05	Phospholipid scramblase 3 (PL scramblase 3) (Ca(2+)-dependent phospholipid scramblase 3)
P04441	HG2A_MOUSE	0.000208	0.0002439	0.00011665	H-2 class II histocompatibility antigen gamma chain (Ia antigen-associated invariant chain) (Ii) (MHC class II-associated invariant chain) (CD antigen CD74)
Q99NB9	SF3B1_MOUSE	0.000617	0.0003643	0.0003006	Splicing factor 3B subunit 1 (Pre-mRNA-splicing factor SF3b 155 kDa subunit) (SF3b155) (Spliceosome-associated protein 155) (SAP 155)
Q9CY58	PAIRB_MOUSE	0.000171	0.0001204	0.00021685	Plasminogen activator inhibitor 1 RNA-binding protein (PAI1 RNA-binding protein 1) (PAI-RBP1) (SERPINE1 mRNA-binding protein 1)
P62751	RL23A_MOUSE	0.0000185	2.318E-05	0.00011665	60S ribosomal protein L23a
P70206	PLXA1_MOUSE	0.000283	9.397E-05	0.00015068	Plexin-A1 (Plex 1) (Plexin-1)
Q61703	ITIH2_MOUSE	0.000158	0.0006861	0.000198	Inter-alpha-trypsin inhibitor heavy chain H2 (ITI heavy chain H2) (ITI-HC2) (Inter-alpha-inhibitor heavy chain 2)
O35604	NPC1_MOUSE	0.000211	2.639E-05	4.9713E-05	NPC intracellular cholesterol transporter 1 (Niemann-Pick C1 protein)
Q69ZK0	PREX1_MOUSE	0.000155	4.698E-05	0.00023422	Phosphatidylinositol 3,4,5-trisphosphate-dependent Rac exchanger 1 protein (P-Rex1)

					(PtdIns(3,4,5)-dependent Rac exchanger 1)
P70268	PKN1_MOUSE	0.0000911	4.957E-05	0.0002172	Serine/threonine-protein kinase N1 (EC 2.7.11.13) (Protein kinase C-like 1) (Protein kinase C-like PKN) (Protein-kinase C-related kinase 1) (Serine-threonine protein kinase N)
P29452	CASP1_MOUSE	0.0000548	2.318E-05	0.00011721	Caspase-1 (CASP-1) (EC 3.4.22.36) (Interleukin-1 beta convertase) (IL-1BC) (Interleukin-1 beta-converting enzyme) (ICE) (IL-1 beta-converting enzyme) (p45) [Cleaved into: Caspase-1 subunit p20; Caspase-1 subunit p10]
Q71LX4	TLN2_MOUSE	0.000287	4.636E-05	0.00033315	Talin-2
Q99104	MYO5A_MOUSE	0.000339	9.334E-05	9.9635E-05	Unconventional myosin-Va (Dilute myosin heavy chain, non-muscle)
Q9QUM0	ITA2B_MOUSE	0.00025	0.0002407	3.3469E-05	Integrin alpha-IIb (GPalpha IIb) (GPIIb) (Platelet membrane glycoprotein IIb) (CD antigen CD41) [Cleaved into: Integrin alpha-IIb heavy chain; Integrin alpha-IIb light chain]
Q8VEN2	PLET1_MOUSE	0.000353	0.0004878	0.00028302	Placenta-expressed transcript 1 protein (Antigen mAgK114)
Q9R1Q9	VAS1_MOUSE	0.000206	0.0002941	0.00011683	V-type proton ATPase subunit S1 (V-ATPase subunit S1) (Protein C7-1) (V-ATPase Ac45 subunit) (V-ATPase S1 accessory protein) (Vacuolar proton pump subunit S1)
P27512	TNR5_MOUSE	0.0000744	2.639E-05	0.0001503	Tumor necrosis factor receptor superfamily member 5 (B-cell surface antigen CD40) (Bp50) (CD40L receptor) (CD antigen CD40)
Q9WVL2	STAT2_MOUSE	0.0000955	9.976E-05	0.0002505	Signal transducer and activator of transcription 2
Q07139	ECT2_MOUSE	0.0000185	0.0003147	1.7016E-05	Protein ECT2 (Epithelial cell-transforming sequence 2 oncogene)
Q8BVG4	DPP9_MOUSE	0.000186	4.698E-05	0.00016675	Dipeptidyl peptidase 9 (DP9) (EC 3.4.14.5) (Dipeptidyl peptidase IX) (DPP IX) (Dipeptidyl peptidase-like protein 9) (DPLP9)

P70398	USP9X_MOUSE	0.0000363	2.38E-05	0.00014973	Probable ubiquitin carboxyl-terminal hydrolase FAF-X (EC 3.4.19.12) (Deubiquitinating enzyme FAF-X) (Fat facets homolog) (Fat facets protein-related, X-linked) (Ubiquitin carboxyl-terminal hydrolase FAM) (Ubiquitin thioesterase FAF-X) (Ubiquitin-specific protease 9, X chromosome) (Ubiquitin-specific-processing protease FAF-X)
Q9CW03	SMC3_MOUSE	0.0000178	0.0008553	0.00010153	Structural maintenance of chromosomes protein 3 (SMC protein 3) (SMC-3) (Basement membrane-associated chondroitin proteoglycan) (Bamacan) (Chondroitin sulfate proteoglycan 6) (Chromosome segregation protein SmcD) (Mad member-interacting protein 1)
P26011	ITB7_MOUSE	0.00032	0.000513	0.0002165	Integrin beta-7 (Integrin beta-P) (M290 IEL antigen)
Q0VE82	CPNE7_MOUSE	0.000299	0.0001937	0.00026713	Copine-7 (Copine VII)
Q8R4U7	LUZP1_MOUSE	0.000184	0.0003907	0.00015068	Leucine zipper protein 1 (Leucine zipper motif-containing protein)
Q6URW6	MYH14_MOUSE	0.000887	0.0004428	0.00065203	Myosin-14 (Myosin heavy chain 14) (Myosin heavy chain, non-muscle IIc) (Non-muscle myosin heavy chain IIc) (NMHC II-C)
Q9Z1M7	LARG1_MOUSE	0.000108	0.0001204	3.3646E-05	LARGE xylosyl- and glucuronyltransferase 1 (EC 2.4.-.-) (Acetylglucosaminyltransferase-like 1A) (Glycosyltransferase-like protein) [Includes: Xylosyltransferase LARGE (EC 2.4.2.-); Beta-1,3-glucuronyltransferase LARGE (EC 2.4.1.-)]
A2AF47	DOC11_MOUSE	0.0000185	0.0001204	0.0001157	Dedicator of cytokinesis protein 11 (Activated Cdc42-associated guanine nucleotide exchange factor) (ACG) (Zizimin-2)
Q9JHU9	INO1_MOUSE	0.0000363	0.0001963	8.3359E-05	Inositol-3-phosphate synthase 1 (IPS 1) (EC 5.5.1.4) (Myo-inositol 1-phosphate synthase) (MI-1-P synthase) (MIP synthase)

Q6PDN3	MYLK_MOUSE	0.0000944	0.0002169	8.3182E-05	Myosin light chain kinase, smooth muscle (MLCK) (smMLCK) (EC 2.7.11.18) (Kinase-related protein) (KRP) (Telokin) [Cleaved into: Myosin light chain kinase, smooth muscle, deglutamylated form]
Q9JHE3	ASAH2_MOUSE	0.0000185	0.0001731	1.6453E-05	Neutral ceramidase (N-CDase) (NCDase) (EC 3.5.1.-) (EC 3.5.1.23) (Acylsphingosine deacylase 2) (N-acylsphingosine amidohydrolase 2) [Cleaved into: Neutral ceramidase soluble form]
Q9Z127	LAT1_MOUSE	0.000304	0.000464	0.00018394	Large neutral amino acids transporter small subunit 1 (4F2 light chain) (4F2 LC) (4F2LC) (L-type amino acid transporter 1) (LAT1) (Solute carrier family 7 member 5)
Q69Z38	PEAK1_MOUSE	0.000302	0.0003643	0.00014956	Inactive tyrosine-protein kinase PEAK1 (Pseudopodium-enriched atypical kinase 1) (Sugen kinase 269) (Tyrosine-protein kinase SgK269)
