# Supplementary material for: Proteomic Characterization, Biodistribution, and Functional Studies of Immune-Therapeutic Exosomes: Implications for Inflammatory Lung Diseases
Source: Front Immunol. 2021 Mar 25;12:636222. doi: 10.3389/fimmu.2021.636222 (PMC8027247; doi:10.3389/fimmu.2021.636222)
Supplement: Supplementary file 2 [file DataSheet_2.pdf]

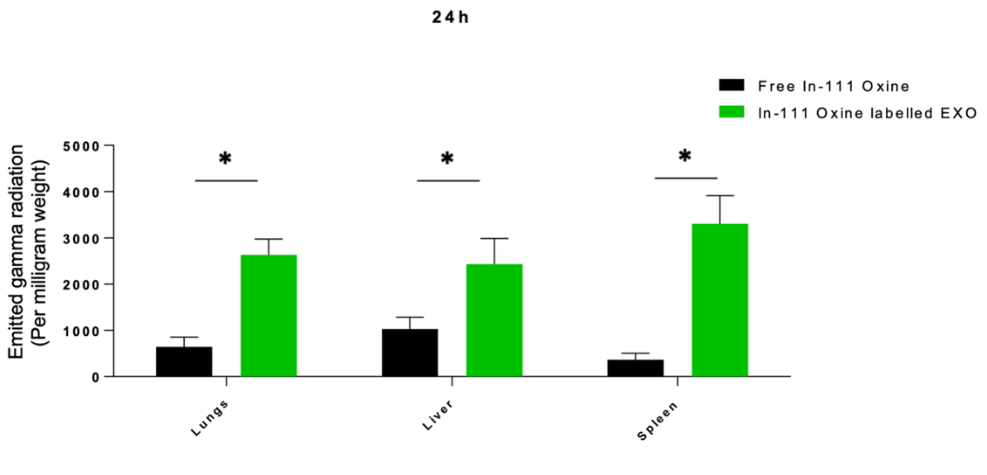

S Figure 1. *Ex vivo* measurement of Gamma Radioactivity in post-mortem isolated tissue, when free radiolabel or bound to DCs EXO, determined by gamma counter after 24 hrs of administration.
